# An extended case study on the phenomenology of sequence-space synesthesia

**DOI:** 10.3389/fnhum.2014.00433

**Published:** 2014-07-03

**Authors:** Cassandra Gould, Tom Froese, Adam B. Barrett, Jamie Ward, Anil K. Seth

**Affiliations:** ^1^Sackler Centre for Consciousness Science, University of SussexBrighton, UK; ^2^Department of Informatics, University of SussexBrighton, UK; ^3^Clinical Imaging Sciences Centre, Brighton and Sussex Medical SchoolBrighton, UK; ^4^Departamento de Ciencias de la Computación, Instituto de Investigaciones en Matemáticas Aplicadas y en Sistemas, Universidad Nacional Autónoma de MéxicoMexico, Mexico; ^5^Centro de Ciencias de la Complejidad, Universidad Nacional Autónoma de MéxicoMexico, Mexico; ^6^School of Psychology, University of SussexBrighton, UK

**Keywords:** sequence-space synesthesia, phenomenology, elicitation interview, conscious experience, case study

## Abstract

Investigation of synesthesia phenomenology in adults is needed to constrain accounts of developmental trajectories of this trait. We report an extended phenomenological investigation of sequence-space synesthesia in a single case (AB). We used the Elicitation Interview (EI) method to facilitate repeated exploration of AB's synesthetic experience. During an EI the subject's attention is selectively guided by the interviewer in order to reveal precise details about the experience. Detailed analysis of the resulting 9 h of interview transcripts provided a comprehensive description of AB's synesthetic experience, including several novel observations. For example, we describe a specific spatial reference frame (a “mental room”) in which AB's concurrents occur, and which overlays his perception of the real world (the “physical room”). AB is able to switch his attention voluntarily between this mental room and the physical room. Exemplifying the EI method, some of our observations were previously unknown even to AB. For example, AB initially reported to experience concurrents following visual presentation, yet we determined that in the majority of cases the concurrent followed an internal verbalization of the inducer, indicating an auditory component to sequence-space synesthesia. This finding is congruent with typical rehearsal of inducer sequences during development, implicating cross-modal interactions between auditory and visual systems in the genesis of this synesthetic form. To our knowledge, this paper describes the first application of an EI to synesthesia, and the first systematic longitudinal investigation of the first-person experience of synesthesia since the re-emergence of interest in this topic in the 1980's. These descriptions move beyond rudimentary graphical or spatial representations of the synesthetic spatial form, thereby providing new targets for neurobehavioral analysis.

## Introduction

**Figure 1:**
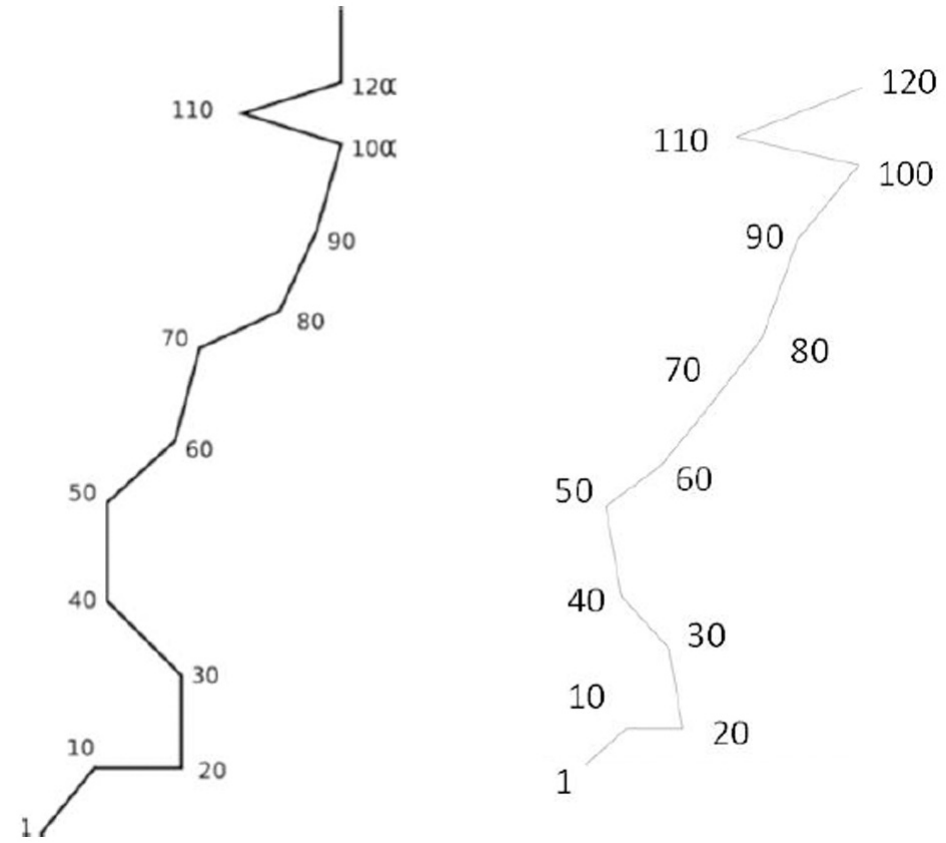
**AB's number form as produced at the start of the investigation (left) and 22 months later (right)**.

In sequence-space synesthesia (SSS) (also known as spatial-form synesthesia), ordinal sequences such as numbers, letters and calendar months are experienced as occupying precise locations in extended areas of space (Price, 2009; Ward, 2013). Despite affecting up to 20% of the population (Sagiv et al., 2006; Mann et al., 2009), our understanding of the visual phenomenology of this experience is largely limited to schematic spatial representations of concurrents spatial-forms (see for example Figure 1).

**Figure 2:**
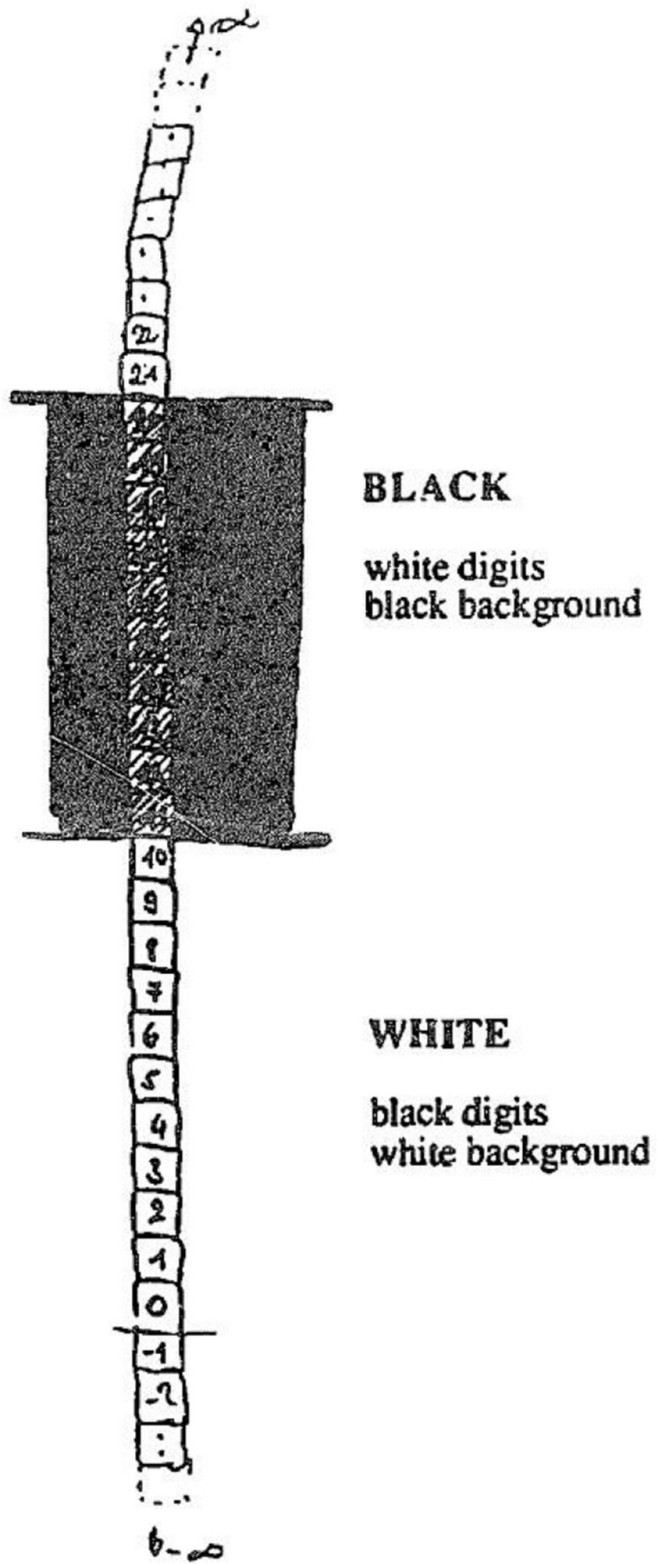
**Number form for subject JB reported in Seron et al. (1992).** Note the features of defined background color, similar to the context reported by our subject, and the high contrast between digit and background color, which we propose to be a feature of the context, enabling the concurrent to be readily visible. Reproduced with permission from Seron et al. (1992), with permission from Elsevier Limited. License Number: 3364950446639.

Some of the most complete introspective reports of SSS were collected from 26 SS synesthetes, via a questionnaire with both open-ended and “yes/no/maybe” questions (Seron et al., 1992). This investigation of Seron et al. uncovered a number of features afforded little attention in subsequent research. For example, one subject indicated that certain sections of their number form had a black background and white digits, whilst other sections had a white background and black digits (see Figure 2). Nineteen subjects reported that once “activated,” a concurrent could not be moved (Seron et al., 1992, p. 174). Subjects also reported being better able to “make use” of their representation where they had a free “visual field,” e.g., when their eyes were closed, and that the “vividness” of the number concurrent increases when the subjects concentrate on it (Seron et al., 1992). One subject reported automatically experiencing the concurrents immediately surrounding the induced item, although these adjacent numbers were “a bit less salient” (Seron et al., 1992, p. 180). Seron et al. re-ignited interest in the exploration of SSS, but concluded that the “genuineness of the number representations” remains the primary open question (Seron et al., 1992, p. 185). Twenty years later, the majority of published research on SSS continues to be concerned with establishing the reliability and behavioral impact of SSS. For example, SSS has been shown to reduce reaction times to items presented in positions congruent to a concurrent spatial location (e.g., Jarick et al., 2009a). However, there has been little regard for the phenomenal aspects which may shed light on other processes known to be modulated by SSS, e.g., visuospatial working memory, visuospatial imagery, sequence representation and memory for personal or historical dates (see Price and Mattingley, 2013 for a review).

The Elicitation Interview (EI, also known as the Explicitation Interview) technique is derived from the phenomenological tradition of Husserl and the study of phenomenological content of conscious experience (Vermersch, 1994). The EI was formulated specifically for the purposes of obtaining descriptions of pre-reflective experience (that is, aspects of our experience which we are not aware of, prior to active reflection upon them), whereas a semi-structured interview is more generally applied to gather information on the reflective experience of the subject. In performing an EI, the trained interviewer guides the introspection of the subject by directing their attention to precise aspects of their phenomenological experience (Vermersch, 1994). This model of pre-reflective access is based on the premise that guided introspection facilitates the transformation of implicit to explicit. Commentary has been provided elsewhere on whether EI itself changes the experience, rather than enabling increased reporting access to the experience (see Froese et al., 2011a). This debate is primarily a philosophical issue of deep versus shallow concepts of lived experience and is outside the scope of this paper.

EI has previously been applied to increase awareness of epileptic prodrome features (Petitmengin et al., 2007), to understand the adaptation strategies of elderly drivers (Cahour et al., 2010), to provide introspective access to unconscious decision making processes (Petitmengin et al., 2013), and to develop and identify distinct “attentional states” before the onset of a cognitive task (Lutz et al., 2002) [see also Maurel (2009) for other applications and Froese et al. (2011a) for a review of recent research using second-person methods]. A full and detailed method for the conduct of an EI has been presented elsewhere (e.g., Petitmengin, 2006). Below we provide a brief overview of the assumptions and priorities in EI data collection, to highlight the unique properties of this method and how it may be applied beneficially in the investigation of synesthesia phenomenology.

During the EI, we aim for the subject to reach and maintain an “evocation state” through interviewer-led direction of attention. Evocation is a deep introspective state in which the subject is in “direct contact” with their experience, and re-living it as they speak. A positive evocation state is suggested by slow and considered speech, avoidance of generalizations, a distant or unfocused gaze and often the use of language in the present tense (the subject may say, “I can see …” rather than “I remember seeing …”). There is some similarity between the induction of the evocation state and that of a hypnotic state. The interviewer may use “Ericksonian” language to refocus the subject and gain access to pre-reflective experience, for example when the participant responds, “I don't know …,” the interviewer may ask, “How do you know that you don't know?” (Petitmengin, 2006). Although the original event may only have lasted a second, through EI we are able to prolong and extend the experience of that event during the evocation state, through subtle use of language and cues. A potential risk in the collection of such first-person data of this kind is the unintentional retrospective transformation of events, as demonstrated by experimental false memory induction (e.g., Loftus and Pickrell, 1995) and false recollection (Greenberg, 2004). Petitmengin et al. (2013) have recently demonstrated that the process of becoming aware of previously pre-reflective aspects of experience does not necessarily lead to confabulation. The specific language used by participants in reporting false memories will, however, allow a skilled interviewer to recognize infidelity in the report and exclude the relevant section from analysis (Froese et al., 2011a). Other bias effects are reduced in the normal practice of EI by monitoring and maintaining the evocation state of the subject, to ensure they are reliably verbalizing the experience and not their beliefs about the experience. Active and open questioning, with rephrasing of the subjects own statements, serves to draw attention to specific aspects which may generally be overlooked in normal discourse. Examples of good practice in bracketing to reduce bias effects and maintaining evocation are provided in the Materials and Methods section.

We present here an extended phenomenological description of SSS in a single case. We apply the EI technique, and demonstrate that qualitative second-person techniques can be used to gather first-person information about a perceptual experience of SSS, which may be used to guide future qualitative and quantitative investigations. The assumptions of EI make it particularly suited to dissecting processes such as SSS, which appear to occur automatically or with little conscious effort, but which also retain a cognitive “anchor” or event upon which to ground the interview. Steady-state experiences such as synesthesia may also engender high fidelity reports, as the event can be reported immediately as it unfolds, rather than retrospectively. The results of the EI were analyzed following Petitmengin (2006), involving successive iterations of aggregation and abstraction of themes, schemas or structures of the experience. This process is similar to the more widely practiced Grounded Theory analysis of qualitative data (Glaser and Strauss, 1967). The themes uncovered via this method have been collated and organized into a detailed and expandable html mind map, which we have made available to readers via the Supplementary Materials. This paper, supported by the Supplementary Materials, presents converging evidence on the reproducibility of the Seron et al. phenomenological investigations. Importantly, we extend the level of phenomenological detail acquired and describe novel aspects of the SSS experience. We demonstrate that a more complete understanding of SSS phenomenology may shed light on features of the experience such as the spatial reference frame in which the concurrents exist, along with issues relating to the apparent automaticity of concurrents and vividness of spatial-forms, which in turn may speak to the recent re-framing of SSS as relating to enhanced visual imagery (Price and Mattingley, 2013). We also demonstrate that greater detail in our understanding of the phenomenological experience of SSS can reveal subtle idiosyncratic aspects of the SSS experience, which may contribute to our understanding of the development of this trait.

## Materials and methods

The subject (AB) is a 30 year old, right handed male with no medical history of psychological or neurological trauma. AB reported to have experienced SSS for numbers, days of the week and months, for as long as he could remember. Prior to interview, no facet of AB's SSS was identified as particularly remarkable or out of the ordinary. To test the consistency of his spatial-forms, AB was asked to reproduce his number forms at the start of data collection and again 22 months post interview. These two schematics are sufficiently similar to confirm the genuineness of AB's synesthesia (see Figure 1).

### Conduct of the interviews

Interviews were conducted by a trained and experienced EI practitioner. Nine hours of interview were conducted over nine non-consecutive sessions spanning 2 months. The interviews were conducted in a private room and recorded following the informed consent of the subject. The interviews were exploratory and involved a fluid examination of topics of interest as they arose. Inducers were presented audibly, for example the interviewer would ask “If I say, five … What do you see?” (20100215_79-81)^1^. A visually presented inducer was used on three occasions (20100408_3, 20100408_65, 20100415_19), with the inducer drawn on a page of the interviewer's notebook. The nature and purpose of the EI method was defined for the participant at the beginning of the first interview session. The interviewer highlighted the requirement for the subject to describe the experience in detail, and reminded the participant of the interviewer's role in guiding and maintaining the focus of attention, suggesting that these particular skills may develop as the series of interviews progress (20100215_2). EI is more commonly applied in the exploration of past events, either naturally occurring or triggered for the purposes of the interview. In the present investigation, experiences were triggered by the interviewer, during the interview itself, and then immediately described and re-evoked. This deviation from the standard EI practice enabled the free exploration of the SSS experience, rather than limiting the interview content to a single event. Each interview session began by revisiting the features of interest in the previous interview, or addressing any topics which had been raised informally between interviews.

#### Maintaining the subject's focus

The interviewer used active questioning and reformulation of the subject's own words in order to maintain attention on a single instant or aspect of the experience, as exemplified in the questioning style demonstrated below.

AB: *Ahm … I'm looking at it … at the moment I'm projecting it onto the screen in front of me and just … It's all on that screen.* INTERVIEWER: *So if you look at that screen …* (20100215_45-46).

Here the subject refers to concepts of “projecting” and “screen,” both of which have not been explored at this stage. In order to gain a description of what the subject means by the use of these words, the interviewer simply asks AB to look at the screen and describe what he sees.

AB: *So, so you can sort of see a floor underneath the five, and then there's kind of a wall … ahm* (20100222_20, 20100222_22). INTERVIEWER: *And if you focus on the wall and the floor, and try to keep your focus there, how does the periphery look?* (20100222_32).

Here the interviewer uses the terms “wall” and “floor” as the subject did. This reuse of the subjects own words serves to maintain and focus his attention on these specific features.

#### Bracketing for bias reduction

“Bracketing” interpretations of events is conducted during the interview and during analysis, to minimize the interpretive bias. Data were removed from analysis if the interviewer's questioning was deemed to be leading. Examples of bracketed or excluded data are given below for clarity (emphasis added).

1. INTERVIEWER: *Can you say something of where this is being presented to you?* AB: *Yeah, **I think I'm projecting a mental image** in front of me*. INTERVIEWER: *Okay, well maybe we'll come back to that later* (20100215_16-23).
2. INTERVIEWER: *But interestingly enough, the number line, when I asked you to have one behind you, **latched** onto the floor* (20100329_206).

In (1) the interviewer identified a “belief” or theory that the subject held about his experience. The subject says, “I think I'm …,” demonstrating that he is trying to infer or interpret what he is doing, rather than simply describing the experience. He interprets this action as “projecting.” “Projecting” is a term often used in the context of synesthesia to refer to the relative location of concurrents, in external or peripersonal space, and as such may be used by the participant as an analogy or “short-cut” to describe a projector-like experience. The interviewer chose to immediately divert from this description and return to the topic at a later time^2^. In (2) the interviewer allowed their own beliefs about the experience to enter the discourse. The term “latching” is not used by the subject himself at any point in the interview. The line of questioning suggests that the interviewer formed a belief regarding attachment between the concurrent and another object in earlier interview sessions, however, this is the only time in which the belief is imposed upon the subject. This section of interview and all related discourse were removed from analysis.

### Analysis

**Figure 3:**
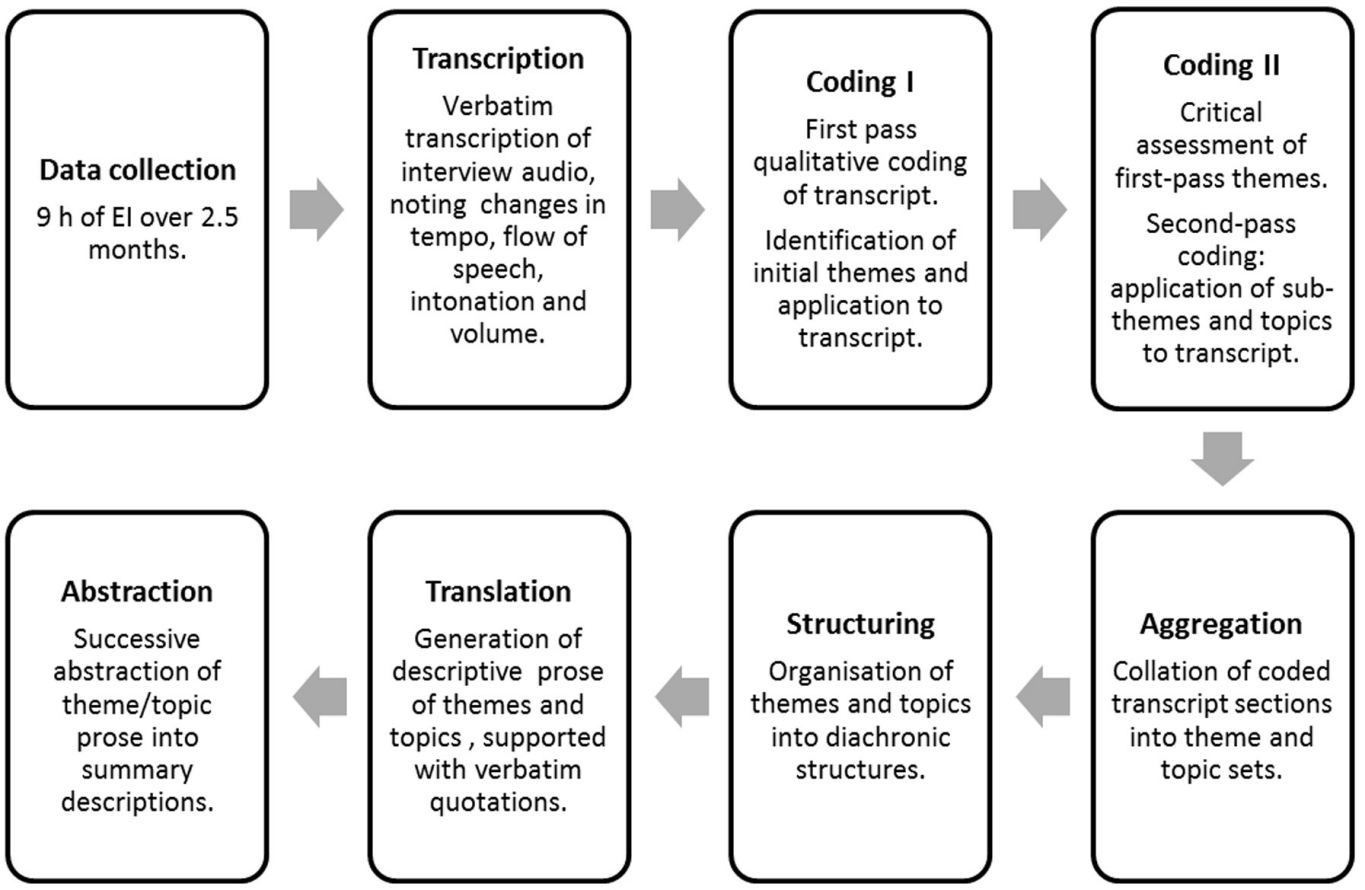
**Schematic of data collection and analysis process**.

Interview data were transcribed and then processed through successive rounds of coding, aggregation and abstraction, as illustrated in Figure 3. Data analysis was conducted blind (that is, not by the interviewer), by a researcher trained in the EI method with no prior theoretical knowledge of SSS, as suggested in Froese et al. (2011b). Blind analysis enabled the independent observation of any interviewer bias and ensured that the themes devised were are only those which are present in the data.

Interview audio files were transcribed verbatim by contracted professionals, including stammers and interjections. Transcribers noted changes in pace, intonation or volume of speech, where specific emphasis is placed on words, and significant or lengthy pauses, to assist in the monitoring of evocation state. Transcribers were blind to the nature and purpose of the interviews. Fidelity of transcription was checked against a random sample of audio files. Transcribed data were assessed for validity according to the principles of EI, by confirming that the report related to the experience as it happened and did not contain comments or thoughts about the experience on the part of the participant or interviewer. Reports were also examined for consistency over the interview sessions. Any reports found to be incompatible with the EI requirements or inconsistent across sessions were excluded from analysis.

The complete set of interview transcripts were read through by the analyst, to identify initial themes or topics. Themes were then summarized into short statements and a code devised to represent the theme. The code was subsequently attached to relevant sections of the transcript using QSR NVivo 9 software (QSR International, 2010). Once all data relevant to a particular theme had been coded, the theme summary was revised and new sub-theme codes were devised. A second round of coding was then conducted using sub-themes.

The coded transcript was exported with paragraph references into Freemind mind mapping software (FreeMind Project, 2010), clustered by themes and sub-themes. Individual excerpts were systematically arranged into appropriate headings (with further subheadings generated where necessary) to ensure all references to a topic were collated. The tree-like representation of the mind-mapping software enabled a systematic analysis of the temporal development of events through vertical arrangement of branches (diachronic structure), and aggregation of simultaneously occurring aspects of the experience (synchronic structures) under a single heading. Ordered clusters were summarized into short sections of prose describing the content and context of the cluster. Abstractions were made for every level of the map from the lowest to the highest level of the theme (for example, the description of the concurrent contexts includes 4 levels of abstraction, over 77 separate sub-headings—see Supplementary Material).

## Results

The EI sessions provide comprehensive descriptions of the first-person experience of SSS for subject AB, including aspects of which AB was previously unaware. Analysis of EI data identified several themes of potential importance to SSS research. Full detail of each of these themes are provided in an html expandable mind map via the Supplementary Material, with the bottom-most level of each branch containing all transcript references which were collated in the generation of the theme. The themes and descriptions are not exhaustive of the SSS phenomenology for this participant, rather they reflect the developing discourse of the interview sessions.

### Discourse content

Table 1 outlines the coverage of individual codes in the transcripts. The majority of the discussion was related to AB's number form (55.8% of all discourse) as opposed to his calendar or alphabet forms (15.5% and 10.3%, respectively). Some of the most informative phenomena were only covered briefly during interview (for example only 1.4% of the transcripts are related to interference from the physical room, see section Mental room), however, when these brief statements are collated and examined in terms of their interactions in the data as a whole, we find them to be relevant to much of the synesthetic experience.

**Table 1:** **Coding summary report**.

**Theme**	**Coverage (%)**	**No. occurrences**
**Forms**	**101.2**	**49**
*Number*	*55.8*	*32*
*Alphabet*	*10.3*	*6*
*Age*	*6.1*	*1*
*Calendars*	*15.5*	*6*
**Physical-mental room**	**0.02**	**33**
*Interference from physical room*	*1.4*	*29*
**Contexts**	**27.38**	**361**
*White Page*	*0.08*	*99*
*Corridor*	*0.08*	*122*
*Free Space*	*0.03*	*17*
*Line*	*0.14*	*156*
*Cave*	*0.02*	*17*
*Felt presence*	*0.02*	*28*
*Context transitions*	*0.02*	*32*
**Inducer-concurrent relationships**	**11.3**	**56**
*Multiple representations*	*7.7*	*50*
**Perspective**	**5.1**	**69**
**Stability**	**6.1**	**60**
*Attention*	*3.2*	*31*
*Knowledge*	*1.5*	*22*
**Voluntarily controllable acts**	**6.4**	**56**

Major themes are given in bold font, with sub-themes in italics. Coverage represents the percentage of all interview data which were coded to a particular theme or sub-theme. A reference may be coded to two or more items when a particular section of discourse is relevant to a number of different themes, thus the total coded coverage does not sum to 100%. Number of occurrences gives the number of separate references to a theme or sub-theme within the interview data, with each instance being variable in length from a single line to an extended discussion. As the interviews were exploratory, the coverage does not necessarily reflect the content of general SSS experience, rather it relates to the content of the interview.

### Basic form descriptions

The forms examined include a spatial number form, mathematical concepts such as pi, an alphabet form, a form for age, a calendar form for years, a form for the months of year and multiple forms for time. These data provide a comprehensive description of the visual synesthetic experience and on the whole are consistent with descriptions obtained by less rigorous methods. This continuity demonstrates that in terms of basic phenomenology, our participant's experience may be considered “normal” for SSS. Brief descriptions of the spatial forms are provided below, with further detail available in the Supplementary Materials.

#### Number form and mathematical concepts

Number concurrents generally appear as black Arabic symbols in an Arial-like type face. Certain numbers (notably five and fifty six) are described as having “metallic” (20100222_247) or “velvety” textures (20100222_321) or surface patterns such as “cross hatching” (20100408_67) or pockmarks, “as if the ink has come away in lots of places” (20100320a_246). The texture or patterning may move around and change although the number itself remains static.

Both fractions and decimals are viewed in the correct position relative to their surrounding numbers. Different notations of fractions can be generated intentionally (e.g., 1.5 may be represented as 3/2 or 6/4)and appear as though superimposed on top of each other. Large numbers (in the thousands) appear with the comma separator.

When discussing mathematical concepts, multiple defining equations and graphs are involuntarily experienced following a related inducer. For example, when discussing Planck's constant the subject reports, “I got e equals H bar times F …. And just underneath I've got H bar times a Greek nu as well […] I've got H bar equals H over 2 pi.” (20100320a_219-224). These concurrents include visual experiences of letters and numbers to multiple decimal places, and associated graph forms. For example, INTERVIEWER: “What about e? AB: So I've got a flash of the exponential curve and then I saw the letter e, it took a while for the equal sign and numbers to appear but then they are there.” (20100320a_71-72). “INTERVIEWER: If you go back to e, do you see the two graphs that you mentioned? AB: Yeah, kind of one over here and one over there.” (20100320a_137-138).

Numbers such as pi are experienced in their correct position in relation to the integer on the number line. A black area or “black hole” (20100229_200) is experienced for numbers with infinite decimal places, with the decimal numbers trailing toward the black hole, “sucking up all those infinite digits … just sort of get them out of the way” (20100229_200). The black hole is flat and AB can change his perspective to view it from behind (20100229_122).

#### Alphabet form

AB's synesthetic form for the alphabet appears as capital letters (20100329_215), in an Arial-like type face (20100329_337-339) with a “ghost-like” translucency (20100408_313) and a reproducible spatial relationship between alphabet items. Specific patterns or textures are generally not reported with the letter form, however, following inspection of the letter B it is described as though “it's made of ribbons attached to each other” (20100408_329).

#### Ages

AB experiences concurrents similar to his number form when exploring concepts of age. AB reports, however, that the experiences of age and number concurrents are differentiated because numbers (such as 28) are more often used in the context of age rather than mathematics (20100320b_90).

The concurrents elicited by inducer concepts of prenatal ages (conception to birth) are accompanied by static visual scenes of embryonic development to a hospital scene with a woman in labor representing birth (20100320b_103-138). The description of the scene for birth (age zero) contains doctors and nurses in a hospital room, medical implements on a tray, storage drawers and a work surface, a woman lying on a bed, a window which looks onto the hospital corridor and electrical equipment over the bed (20100320b_153). AB reports that he can hear the sound of a baby crying from somewhere outside the scene but no other sounds from the hospital room itself (20100320b_139-149). When AB is asked if there is anything “before” that scene (equivalent to negative numbers) he reports a “vague sense of something in the womb” (20100320b_110) but with an unknown or indistinct location. When the interviewer induces “6 months pregnancy” AB is able to identify the location of this age and reports “a sort of fairly black womb with a sort of like fetus in it” (20100320b_112). AB also describes other scenes in separate spatial locations, including conception “at the very top” as a “magnified picture of an egg with loads of sperm” (20100320b_127), then a fertilized egg and a “clump of cells” (20100320b_114) followed by further cell divisions in the embryonic development. Zero and one years of age are separated in space to accommodate “all those first months in the first year of life” (20100320b_182). When directed to the zero time point, AB reports images of a new-born baby “wrapped up in white blankets”, and at 6 months old AB reports hearing a baby cry (20100320b_192).

The interviewer proposes that the first year might contain images of a baby “crawling around on the floor, or something like that” (20100320b_195). At this stage AB remarks that he is beginning to experience “a few more general images” (20100320b_196), including the memory of an occasion where he saw a friends child at one year old. AB spontaneously offers that these images are quite distinct from those described in relation to prenatal age, as he is positioned within the scene and it is surrounding him (20100320b_196), where as in the pictorial representations of age AB was not himself a feature of the scene.

#### Calendars

AB reports a spatial form for calendar aspects such as years, months and time. His year form is similar to his number form, but there is no comma separator. The inducer “one thousand, nine hundred and ninety five” leads to the experience of a number concurrent, whereas the inducer “nineteen ninety five” leads to a year concurrent (20100320a_237). AB is able to describe and draw his spatial form for the more abstract year inducer of 4000 BC, with an inflection point between BC and AD.

AB's spatial form for months includes the experience of digits and a line arranged circularly, with August at the top and subsequent months arranged clockwise, with February at the bottom. Within the months themselves each 10 day segment follows a specific direction on the line such that AB can identify a date not only by the position of that month in relation to the others but also by the direction of the numbers on the line surrounding the date.

AB reports a clock concurrent induced by the time of day. His synesthetic clock takes multiple forms: (1) an analog clock face with hour and minute hands, minute markers and larger hour markers; (2) an orange digital clock with a flashing semicolon for seconds; (3) a separate digital clock with a different type-face. In directing his attention to aspects of the analog clock, AB is able to induce the experience of digits on the clock face where there were none before. AB reports the apparent motion of a second hand as it flashes “in and out of existence” (20100320b_16-18) each time his attention returns to it.

### Themes within the SSS experience

**Figure 4:**
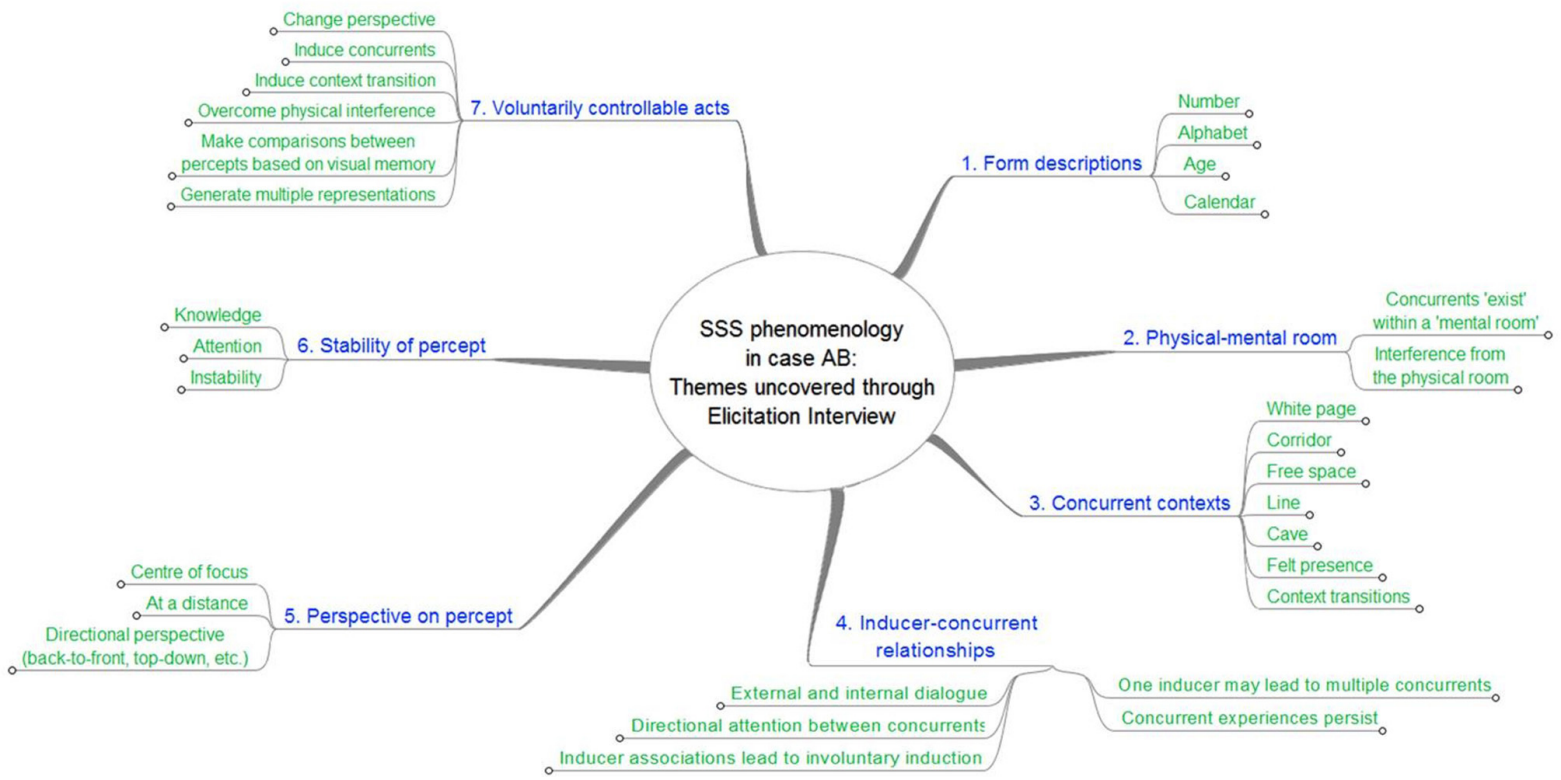
**Mind map of content, themes and sub-themes derived from the EI data with case AB.** This map is reproduced in the Supplementary Material, where summary descriptions are provided at each level, and the collated transcript references supporting our interpretation are provided at the bottom-most level for each theme.

The themes identified in the EI go beyond basic form descriptions of as they address precise aspects of the experience and how it unfolds. Six main themes were identified from the interviews, as illustrated in Figure 4.

#### Mental room

AB describes his synesthetic percepts as existing in a “mental room” (20100320a_108) as opposed to the physical room, which refers to the room in which the interview was conducted and which supports the normal (non-synesthetic) visual experiences (20100408_193). The percepts in the mental room are “superimposed” on the physical room (20100408_137); they have the same physical location as items in the mental room, but the mental and physical exist as if in two “different dimensions” (20100408_163, 20100408_165, 20100408_189).

*I'm sort of seeing the [physical] room behind [the concurrent]. But then I sort of feel the [concurrent] letters are in a different kind of space as well* (20100329_235).

*It's almost like you have this whole room and then there's like a copy of this whole room (20100408_191). And then the pen stays in this [physical] room. But all the [synesthetic] numbers and things are in the copy of this room [the mental room]* (20100408_193).

AB reports that he can switch his attention between the physical and mental rooms by forced effort (20100320a_108) or defocusing his eyes and “looking into space” (20100408_33). AB retains an awareness of the physical room whilst looking at the synesthetic percepts (20100320a_112) although the physical room appears “out of focus” (20100320a_114), similar to a normal experience of an item in peripheral vision (20100320a_114).

*Erm, well I can just alternate my attention between the physical room and the mental room I've got* (20100320a_108).

*I guess all of the physical room is like erm, like what's in my periphery normally. So the focus of my eyes is somewhere about here, because there's nothing in the physical room here. The whole physical room is out of focus* (20100320a_114).

*INTERVIEWER: So if you focus your attention on the [mental room] … your visual awareness of the [physical] room fades out …? AB: Yeah. It never leaves me completely, the physical room* (20100320a_111-112).

Concurrents tend to be located in areas of space where there is little interference from physical objects, such as the top of an empty table (20100329_89) or in an area of clear space (20100329_107, 20100415_238).

*If my eyes are kind of focused on some point in space in which there is no physical object, then it's easier [for the concurrent to appear]* (20100408_31).

*If there's an object in this [physical] room, in the same place as one of the numbers are in the other [mental] room, then I see the object rather than the number* (20100408 195).

*The floor in this [physical] room is obscuring the floor in that space [the mental room]. I can't really see down* (20100415_278).

The interference of visual percepts in the physical room upon synesthetic percepts in the mental is also reported in the visual presentation of an inducer, where AB reports having to look away from the “distracting piece of paper” (20100408_13) in order to experience the synesthetic form.

*INTERVIEWER: What do you see? [Shows AB a letter H drawn on a notepad] AB: I see the letter H on the page. INTERVIEWER: So what do you need to do in order to see the alphabet line? AB: Erm, have to look away from the image. I can see them here* (20100408_3-5).

*When I was focused on the paper there was nothing else except what was physically there (20100408_15). It's almost like the actual physical H can't be part of the mental alphabet* (20100408_23).

*So I see the 4 [drawn in Interviewer's notebook] and then there's nothing really stable coming into view. It's like the number line doesn't know which direction to go in. It's like there are various things down on the table and something over here … Nothing very precise came in. Just very different from when you are telling me to think of the number 4* (20100408_65-67).

#### Concurrent contexts

AB's concurrents appear in a range of “contexts” which describe the perceptual synesthetic experience around the concurrent in the mental room.

**Figure 5:**
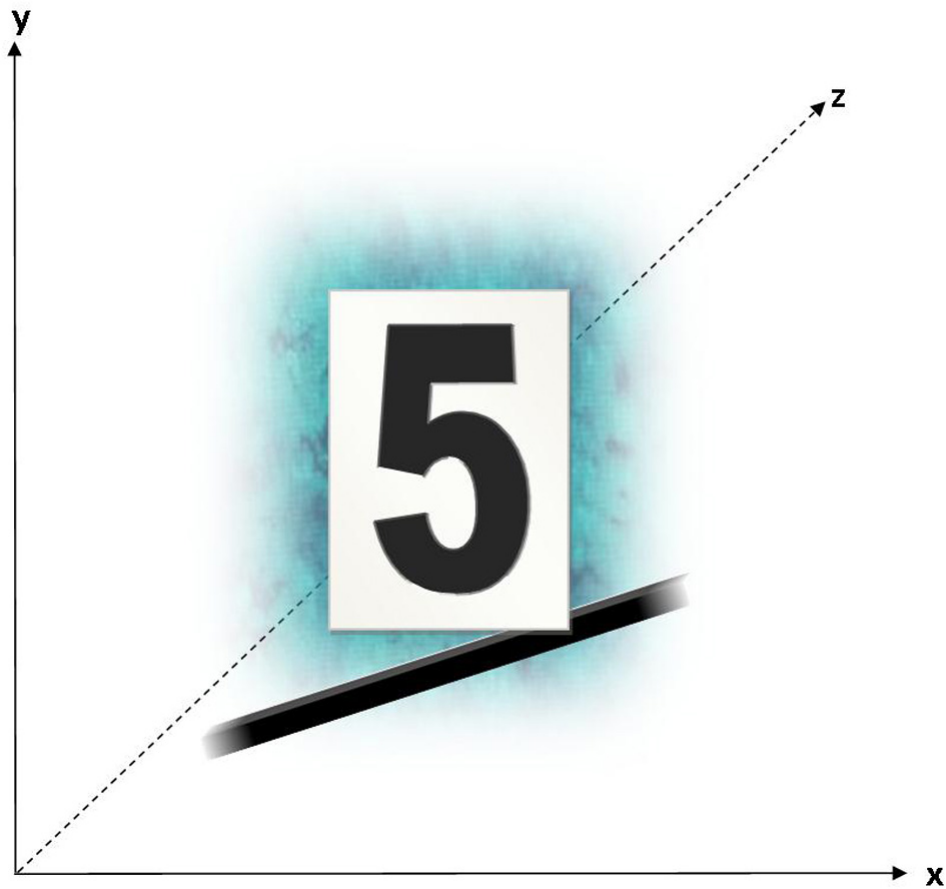
**The “white page” context.** Number concurrents, when viewed in isolation, appear against a white, A4 sized background, with the “line” below, and a purple/green coloring surrounding the page.

***White page*.** The “white page” context is described as an A4 piece of paper, presented vertically in front of AB, forward and below his eye line (see Figure 5). The area around the white page has “a purple layer here, a green layer here and some black.” (20100329_395). Concurrents appear centered on the page in a font size which would fill a “good proportion of the paper … as if there's only room for that one digit [on the page] with a good margin around the edge” (20100215_146-148). The white page provides contrast for the concurrents to be viewed against the background of the physical room, as AB reports that he has difficulty identifying the concurrents when they haven't got the white page behind them:“It's almost better to have the white page there, because it helps me to see the A better” (20100329_399, see also 20100415_43).

**Figure 6:**
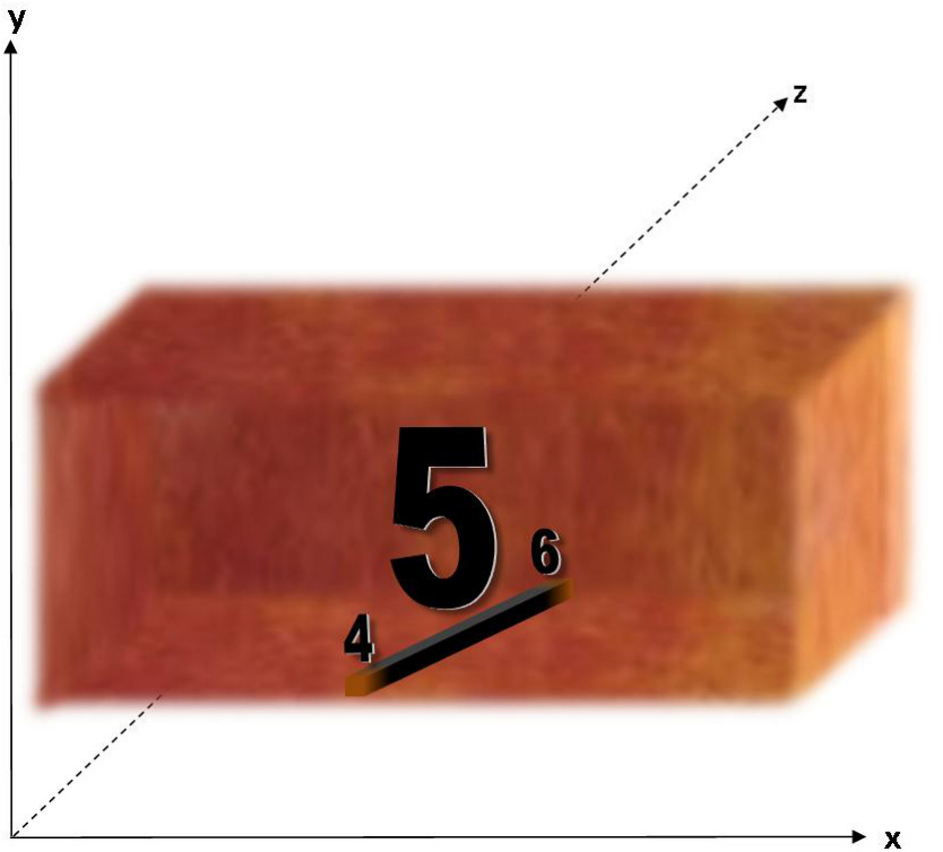
**The “corridor” context, which appears when concurrents are viewed as part of a sequence.** The corridor has a 3D structure, with walls, a floor and ceiling, and red-orange coloring.

***Corridor*.** When viewing a section of the number line (as opposed to individual concurrents), concurrents appear in a “corridor” (see Figure 6). The walls of the corridor are “quite opaque (20100329_42) although “not like a hard surface like the table, if you put anything behind [the wall of the corridor] it would go into the fog and you wouldn't be able to see it” (20100408_349). These walls are described relatively consistently as a “orangey, reddy, greeney color” (20100222_148) elsewhere, “orangey, browney, greeney color” (20100222_103) and “greenish, reddish, orangey” (20100229_108). The floor of the corridor has similar opaque characteristic as the walls (20100222_31, 20100222_186). The corridor has a ceiling (e.g., 20100222_117) and on two occasions the ceiling is referred to with a source of light, with “little circles of light going down the corridor” (20100222_159) and opening up into a “Sun roof” (20100222_326). The corridor itself has a “kind of a yellowish light kind of permeating it” (20100222_146).

The induced concurrent is reported to “float” in the middle of the corridor (20100320a_47) whilst the concurrents immediately surrounding it are against the wall or just outside the corridor. Concurrents with multiple decimal places are experienced with the decimals trailing down the length of the corridor. The size of the corridor appears to vary. On one occasion the corridor is reported to be not much bigger than AB's head (20100222_126), on another it is as wide as AB is tall (20100229_182). The corridor appears to expand width ways to encompass more numbers in the form: “It's kind of like the corridor wants to enlarge and have the 7 over here” (20100329_107).

***Free space*.** The free space context is identified by reference to a specific coloring of the background and a lack of white page or corridor features. AB describes the free space background as “unstable,” “sometimes it's purplish and sometimes it's greenish.” (20100329_13). The background “doesn't make the [physical] room colored. It's just like another world [mental room] that I can focus my attention on, or I can bring my attention back to the [physical] room” (20100415_55).

***Line*.** AB reports a “line” feature in his number and calendar forms. The line is not present in the alphabet form as, “[the alphabet] has no continuous values” (20100329_306). The line is seen both in the free space context, and in the corridor of the number form. The line is black and has a 3D shape like a “very long rectangle” (20100222_58). The line is below the white page in the vertical plane such that AB has to look down from the white page to see the line (20100215_153). AB suggests that he can see the page and the line at the same time, although his language use is slightly ambiguous and may suggest that in switching his attention between the line or page contexts, his perception of the other is diminished:

*I can definitely see it being kind of located below where the page **was*** (20100215_155) [emphasis added].

INTERVIEWER: *Can you see the number line at the same time [as the white page]? AB: Yeah, **when** I see the number line it's going like this and below* (20100229_49-51) [emphasis added].

In the corridor context, the base of the line is “resting on the floor [of the corridor]” (20100222_58), bisecting the corridors width, “going into the wall of the corridor” (20100229_165-166), and follows the gradient of the number form. In the free space context, the number concurrents “hover” just above the line (20100215_172) or the line appears with a gap into which the concurrent would fit if viewed in the corridor (20100222_95), with the gap acting as a “marker for where the [number] should go.” (20100222_109). There is a suggestion that the line functions as a reference for directing AB's gaze or attention to other concurrents, as AB follows it's gradient toward where he “knows” another concurrent to be located (e.g., 20100320a_298).

***Cave*.** The “cave” is the usual context of AB's alphabet form. The cave is described with a “dampness” (20100329_441) or “muddy” atmosphere (20100408_315), a dark background (20100329_426) with depth, like a “a dark hole” (20100329_365). Letter percepts within the cave are described as having “translucent” (20100408_315) or “ghost-like” qualities (20100408_315) rather than a solid form. AB describes his head as being in one wall of the cave, allowing him to look into the depth or toward the entrance. When the entrance of the cave is explored, AB describes it as positioned in the side of a mountain (20100329_459) with a naturalistic scene outside and a “sense of an expansive landscape” (20100329_457), “sky and clouds” (20100329_461).

***Felt presence*.** AB occasionally reports that he can sense the presence of a concurrent but not see it directly. These percepts are often described as “vague,” for example, “these are bit more vague these ones. They are just kind of more like the feeling or the presence of the three there” (20100415_27), “I've kind of got like the sense of the six and the seven sort of somewhere around here. Not really quite sure where they are” (20100329_67).

***Context transitions*.** There are dynamic transitions between the contexts. These transitions generally occur automatically and in a predictable manner, although AB is able to trigger them of his own volition to a limited degree. We present the following brief diachronic description of context transitions for a number concurrent (for a more detailed description of each stage in the transition, see the Supplementary Material):

Initially a new number concurrent is presented on the white page. Surrounding the white page is a color which is similar to the free space context. Directed by a change in AB's focus of attention to the area below the page, the white page “melts” from the bottom to the top. This melting is described as, “[if] the page was made out of ice and there was fire underneath it, and it's kind of melting gradually and kind of in a fairly homogeneous way” (20100329_375). The shift in focus of attention from the page to the line causes a new concurrent to appear above the line. Once the new concurrent is stable, the line and corridor come into view.

#### Internal and external dialog

Auditory presentation appears to be the most effective induction method for AB's synesthetic percepts, as it is not associated with direct physical interference of a visually presented item. The audibly induced item is often accompanied by those items surrounding it in sequence (e.g., the induced experience of a two will be accompanied by a one and three in the correct positions). “Thinking about” an inducer will also lead to the experience of a concurrent (20100305_142), with the thought process often involving inner speech (20100408_48-51). On other occasions AB reports to visualize a concurrent to induce a synesthetic experience (20100222_261-263).

*You say 15, I'm actually seeing more digits now. I sort of see 14 and 16 and 17* (20100215_61).

*So I'm saying M over and over again in my head. But I've got a glimpse of an L at one point. And I've got a glimpse of N O P. It's hard to just look at the M* (20100329_249).

*INTERVIEWER: So if you look at the M, how many letters are you aware of at the same time? AB: Just depends where I focus my attention. If I focus just on the M it's quite hard. It's almost like I have to utter the names of the other letters in my head in order to see them. But as soon as I think, you know, looking at the M and then thinking about what else I can see, need to start uttering M or L and then they come into being* (20100329_244-245).

*INTERVIEWER: So what if you pick a random one, just choose it yourself, how would you go to it? AB: Yes, then I would have to say it in my head. So I think Q was kind of here. … I kind of just thought of the letter Q by saying it in my head, then I kind of looked for it* (20100408_48-51).

#### Directional attention between concurrents

If AB allows his eyes to move freely along the number or alphabet form, he can see as many or as few concurrents as he chooses (20100329_251). Directing attention may also involve inner speech, for example AB reports in one instance that during this act of “looking for a concurrent” he says to himself: “right I'm going to look for the 4 now!” (20100408_138).

*INTERVIEWER: if you let your gaze move a little bit more freely, how easy is it for the other letters to appear? AB: It's pretty easy for H I J K L M N O P Q to appear. R S T. All I have to do is move my attention along the thing. I can kind of see the whole alphabet if I want to* (20100329_250-251).

*INTERVIEWER: What about … the other end of the alphabet, Z? AB: Just have to re-look where it is. INTERVIEWER: It's just a movement of the eyes? AB: Yeah. INTERVIEWER: You don't have to say anything. AB: No, I think you saying it is enough* (20100408_42-47).

AB also has a strong prior knowledge of where each concurrent should be located in relation to another in the same form. Redirection of attention based on prior knowledge also involves inner speech.

*Erm, normally it's like I said to myself, “OK, it's a three so down to the left of three there should be a one and two.” And then, well there they are* (20100408_93).

#### Single inducer leads to multiple concurrents

A single inducer may lead to the experience of multiple representations of the same concurrent, particularly if AB is directed to perform some act upon that concurrent. For example, when AB is asked to view a four from a different angle, he reports to experience two fours, one in the normal, face-on view and one back-to-front (20100408_125-129). New or alternate representations of the same concurrent occupy different locations in the mental room.

*There's a four and a six kind of here. … INTERVIEWER: If you moved your head from side to side like this, how does your perception of the number change? AB: Erm, sometimes it tilts a little bit so it's kind of bi-stable. … It's like there's two here. So there's one where the four is just like that, and then is another one where it's kind of [back to front]. INTERVIEWER: I've noticed that at the moment you are actually standing still. Can you see both of the different ones even though you are not moving? AB: Yeah, there's kind of one like this and there's one kind of like that. INTERVIEWER: So you have two different forms there? AB: Yeah* (20100408_125-129).

#### Associations lead to involuntary inductions

Concurrents may be experienced involuntarily if they are related to the induced concurrent through normal associative mechanisms, for example when “a physical constant […] like the speed of light” (20100320a_195-196) is presented as an inducer, AB reported the involuntary experience of five concurrents associated with other physical constants (20100320a_195-224). Similarly, when AB is asked to transform a letter into an italic font, a bold font (an associated type-face) is perceived (20100329_340-345).

*INTERVIEWER: What about a physical constant … Like the speed of light (20100320a_195-196). AB: Yeah, what else came into view was Planck's constant, like the H bar. I just saw like an H bar and then over here there's an equals and a C squared came up (20100320a_202). INTERVIEWER: So how come the Plank's constant appeared? … Did you think of the Plank's constant as well? AB: Yeah, I guess because it's another fundamental constant of the universe. It's like C [the speed of light]* (20100320a_211-214).

*INTERVIEWER: Do you think you could get them in italics? AB: Erm, saw the C though going to italics very briefly, the capital C. … The lower case C is kind of being mixed up. It's kind of in bold font. <laughter>*(20100329_340-345).

#### Concurrent experiences persist

Once induced, the experience of the concurrent was found to persist without the need for it to be directly referred to (20100229_329, 20100408_40-41). Similarly AB reports that a percept may remain stable even if it is taken out of view or attention is directed elsewhere; once attention is returned, the concurrent is described as if it existed independently of AB's attention (20100305_131-132, 20100229_388).

*60 has rooted now because we've been talking about it for quite a bit* (20100229_329).

*INTERVIEWER: When you look at the … alphabet [form], are you aware of having to say H in order to keep it in focus? AB: No, I think once it's there I don't have to say H* (20100408_40-41).

*INTERVIEWER: What if you … take them out of view completely so if you look behind you so immediately you don't see either of them and then you turn around again. Do they remain exactly where they were before? AB: Yeah still five, six [56], 57* (20100305_131-132).

After several concurrents have been induced through the interview process, AB's experience contains multiple independent concurrent items, for example, INTERVIEWER: “Do you have any number lines ready that we could do that with? AB: Erm, I've got all sorts of number lines” (20100408_386-387). This persistence in the visual representation and spatial location of a concurrent caused experimental difficulties where AB found there were too may concurrents present to concentrate on a single item, causing him to feel “bogged up with loads of numbers” (20100229_384). This experience of multiple concurrents was not explored further.

#### Perspective on percept

Concurrents which are directly induced are generally presented in front of AB in the correct perspective, i.e., “facing,” although there is one report of a concurrent being turned away slightly (20100415_185-187).

*I'm kind of seeing the five right in front of me* (20100215_170).

*So I've sort of got the number five to the right of my focus now, and kind of in front of my focus* (20100222_33).

New concurrents may also appear at some distance particularly if they are a new representation of the original concurrent.

*INTERVIEWER: So the zoomed out version of the alphabet disappeared when you switched your attention to the A? AB: … it's kind of in the distance. It's probably further away from me than I can see* (20100329_280-281).

AB is able to view concurrents from different perspectives, e.g., if they are placed behind him or if he physically moves round or above them.

*So this one is directly facing me and that one is kind of facing that way. So I'm kind of slightly sideways on. … if you were in the chair one to your right, it would face you* (20100415_185-187).

*INTERVIEWER: Can you [physically move round to look at the percept from different angles]? AB: I can see the back of the four. Yeah I can kind of see the four backwards. Sometimes it sort of spins around* (20100408_104-105).

#### Stability of percept

AB often reports “instability” in his percepts, as a flashing or brief appearance of the concurrent. This instability is reported when AB attempts to modify a feature of the concurrent from its preferred or “default” form. For example, attempting to change the type-face (20100329_340-345) or perspective of the alphabet form (20100415_97-113) results in an experience of a percept flickering between the default and requested view/feature.

*[From an upper case A] I can get the lower case A to flash [into an upper case A], but then [it] doesn't stay there* (20100329_323).

[AB is asked to see what happens if he looks at a concurrent whilst he changes his viewing angle by standing up] So it's kind of hard to stand up slowly enough … INTERVIEWER: … you're trying to stand up slowly in order to see what? AB: … where some kind of change happens. … it seems to flicker (20100415_97-101). It just came into view when I sort of reached there at a certain angle.

*INTERVIEWER: Is it not on the table right now? AB: It's sort of flickering* (20100415_107-109).

Where AB's knowledge of a number sequence is limited (e.g., in the decimal places of pi), he only experiences stable concurrents for those numbers of which he is certain (20100229_132).

*So now I've got 3.14159 dot dot dot eight, six, five, six, and then a whole pool, a whole load of numbers just kind of like jumbled up, like kind of just there, not really knowing where to go* (20100229_147).

*So it's unstable after 3.141527986. Seems pretty unstable after the third or fourth decimal point. … it's not really holding still it's kind of changing because of other numbers trying to get in from above. … So it's 3.14 and one and a five, and that's where I start getting difficulties … there's a nine above the two. INTERVIEWER: How is it above the two? AB: Just directly above on the page. INTERVIEWER: Is it stable up there? AB: I just see a jumble of numbers* (20100229_71-83).

*INTERVIEWER: What do you see after the five? AB: It's kind of dot dot dot, and then there's kind of some rather blurry numbers to the right in a crowd* (20100320a_14-15).

Selectively directing AB's attention to specific features of a concurrent appears to improve the stability of the percepts and AB's ability to describe it, for example he often uses phrases such as, “if I think a little bit more then I see …” (20100222_5) or “if I really try and focus …” (20100229_398) before going on to describe a feature which was previously “unstable.”

*[AB was asked to inspect the texture of the 5 concurrent in detail] Yeah. Erm but nothing that stays really too stable. **If I really try and focus** I don't know, I see a little white so it's kind of mainly black top bit of it. Erm, then I am seeing a little white circles … [there is a] white circle missing from top corner as though someone has punched a hole out of it from the top corner* (20100229_398) [emphasis added].

#### Voluntarily controllable acts

AB is able to perform a number of acts to modify his synesthetic experience. These include:

1. Changing perspective on a concurrent, either by physically moving around the room or adopting a different mental perceptual position such that he can view the concurrent from a different angle. *INTERVIEWER: … So how does it change when you stand up for example? AB: … looks like I am kind of above it, kind of like this. It's kind of got some depth to it this time* (20100415_68-69).
2. Self-induce concurrents by thinking about them, looking for them in relation to other concurrents or through the internal recitation of the concurrent (inner speech). *INTERVIEWER: Can you create a different number line … Maybe over there for example, if you say 55 over in that direction … AB: I just faced this direction and then thought of 55* (20100305_135-142).
3. Overcome physical interference in the mental room through effortful “willing” of the concurrent to appear. *AB: Well it just appears first away from the [physically presented paper] page. I have to will an alphabet on the page in order to get it to happen* (20100408_75).
4. Report a visual memory of a concurrent without evoking the concurrent itself. *INTERVIEWER: Did you notice any numbers when you came into this room? AB: No, erm maybe I can remember there was something here. … Hard to tell if it's just a memory or if it's really there. INTERVIEWER: Well have a look at the wall. AB: I remember something being over there as well. Can't remember what number it was whether it was a 55 or a 56. Yes, so there's obviously nothing there that's sort of firm there. Kind of got a bit of an image of a big 55 there then a big 56 sort of stuck on the wall there. Nothing really that stable* (20100320a_2-9).

Although AB's reports would suggest that he can in some cases modify a concurrent itself, attempts to do so lead to the generation of a new concurrent with the desired characteristic, whilst the original remains unchanged. AB's ability to exert some level of control over his synesthetic experience demonstrate the dynamic and elaborative nature of the visual experience.

## Discussion

In this paper we used the EI technique to provide a detailed phenomenological analysis of SSS in a single case. The conclusions drawn are appropriate to our participant and further work will be required to test their applicability to a wider population. Some external validation of the themes derived can none the less be achieved, by reference to the existing (limited) descriptions of phenomenology. Where there is continuity between the reports of our participant and those provided elsewhere, we may assume that these particular themes are generalizable to a wider population. As our participant reports common phenomenological details in the basic form descriptions, it is likely that his experience in general is also typical of a wider SSS population. Therefore, where other more novel aspects of phenomenology are derived for this participant, it is possible that these too may be generalizable.

Our results show that AB's synesthetic concurrents exist in what he terms a “mental room” and that he is able to selectively shift his attention to either the mental room of the concurrents or the physical room in which the interviews were conducted. Although the mental and physical rooms co-exist, percepts in the physical room appear to interfere with synesthetic concurrents in the mental room. This interference effect was also found to be a feature of visually presented inducers such that AB had to divert his gaze from the physical inducer in order to perceive the concurrent. The generation of a concurrent frequently followed auditory induction, for example AB uses internal verbilisation of the inducer name in order to perceive the concurrent, even when the inducer was presented visually. We also found that concurrents are automatically experienced by association to the inducer, for example the discussion of the physical constant for the speed of light causes the automatic perception of related physical constants. AB's concurrents are not limited to simple letters/numbers, but also include equations and graph notations. The induced item is often perceived with adjacent items on the number line, for example the induction of a two may lead to the perception of a one and a three. The stability of synesthetic percepts is found to increase with focused attention on the concurrent percept. AB is also able to voluntarily modulate the features of a concurrent, although they appear to have a default type-face and/or perspective.

As this is a case study, it remains to be seen to what extent the precise nature and content of all themes of AB's SSS experience generalize to a wider population. For example, the experience of a “black hole” representative of infinite decimal places may be highly idiosyncratic and unique to AB in his conceptualization of infinity. Other idiosyncratic features may include AB's ability to move around the spatial form whilst it remains static, and the precise nature and content of the concurrent contexts of the white page, corridor and cave. The unique and artificial experience of the EI may have drawn out details of AB's experience which are not a common feature of the every-day SSS experience. For example, on many occasions AB only perceives the attended concurrent, and a few surrounding items. This may be due to the high level of focus placed on individual concurrents for the purposes of guided exploration. Further investigation may determine whether the process of EI can reveal similar themes for other participants. Three themes in particular may be relevant in developing our understanding of SSS, namely, (1) the description of the physical and mental room; (2) the effect of internal and external dialog; (3) the apparent persistence of concurrents after induction.

### Physical-mental room

AB's description of the mental-room can be considered in light of the projector and associator distinctions in grapheme-color synesthesia (Dixon et al., 2004). AB reported in the first interview that he is “projecting a mental image” when he perceives a synesthetic concurrent (20100215_22). AB's description of the mental room as “another dimension” (20100408_185) is interesting, but of itself says little about the experience. More telling is the apparent relationships between the physical and mental rooms such that the physical can obscure or distract from the mental and make it difficult to visualize or fully induce a concurrent. This effect may be informative in providing a basis for the exploration of first-person experience of projector-associator effects, however, the projector-associator distinction is an assumption of sequence-color synesthesia, and not SSS; although SSS experiences have been described as occupying “distinct loci in imaginal, peripersonal or extrapersonal space” (Price and Pearson, 2013, p. 1166), projector-like and associator-like traits have not, to the best of our knowledge, been formalized in SSS. Any direct comparison between the experience of our subject and the current understanding of the projector-associator experience may therefore be premature. The observation that AB can readily distinguish between the physical and mental rooms (even when they interfere) underlines that his synesthetic experiences, while at times perceptually real, are not subjectively veridical—that is, they are not experienced as being part of the external, real world (Seth, 2014).

### Internal and external dialog

AB internally verbalized the name of the inducer each time he self-induced a concurrent (i.e., when the inducer was not presented by the interviewer). This included occasions when he redirected his attention from one concurrent to another, or when he freely chose a concurrent to explore. When he was visually presented with an inducer, he needed to say the name of that inducer internally before the concurrent was experienced, and repeated the name of the inducer to stabilize his visual experience and maintain his focus of attention. This use of inner speech may not be surprising, given the inter-relation of orthographic and phonemic representations in neural processing, however, it was uniquely identifiable by the process of EI and has not been previously demonstrated in SSS; other methods may have accepted AB's initial descriptions (e.g., of the redirection of spatial attention) without probing further into the acts involved in the generation of a concurrent. This is an example of EI enabling reflective access to an aspect of subjective experience which was previously pre-reflective. This also exemplifies the in-depth exploration of diachronic structures of experience promoted by EI.

External auditory instruction was the most direct and effective route to induction of a concurrent for AB. The primacy of auditory induction may account for AB's primary use of inner speech during all other inductions. Auditory-visual synesthesia is commonly considered to include only more overt forms such as the experience of colors with music (e.g., Goller et al., 2009). As such, SSS and other forms (e.g., grapheme-color synesthesia) have typically been assumed to result from visual-visual interactions. There are, however, investigations of visual-visual types which have used auditory presentation of inducers and found results comparable to visually presented inducers (Paulesu et al., 1995; Nunn et al., 2002; Steven et al., 2006). In SSS, a case has also been described in which auditory and visual presentation of an inducer lead to different perspectives of the form being perceived (Jarick et al., 2009b). These findings suggest that auditory induction is sufficient to cause the synesthetic experience, and the evidence here from EI suggests that it is in most cases necessary in the case of AB, even when items are presented visually.

Assuming there is some overlap between the neural mechanisms involved in processing internal versus external speech, the processes of concurrent induction via inner speech may be similar to the processes involved in concurrent induction via external auditory presentation. As all methods of induction engaged inner speech for this subject, the experience of SSS might be re-framed as primarily auditory-visual process for AB and potentially in other SS synesthetes. Compatible with this hypothesis, children in most English-speaking countries learn the ordinal sequence of the alphabet phonemically through song before they are able to associate the visual grapheme and letter name (Ehri, 2009). Seron et al. found that the SS synesthetes they investigated were less efficient in verbal strategies according to Paivio's degree of imagery questionnaire (Paivio and Harshman, 1983) and suggest that the visual spatial form may have developed as a compensatory strategy to reinforce the ordinality of audibly presented items (Seron et al., 1992). This suggestion may have particular implications for the development of SSS, particularly if it is framed as an auditory-visual association, rather than a visual-visual or visual-spatial.

This formalism of SSS as a trait relevant to the development of visuospatial representations has been re-introduced by Price and Pearson (2013), who argued that the apparent automaticity of spatial forms may in fact represent deliberate recall of the specific visuospatial representation. Indeed, Price and Pearson (2013) suggest that the development of the spatial form may be closely related to the development of the phonological loop during rehearsal, where the dual encoding of visuospatial and auditory representations may be beneficial to learning. Our data support the central intuitions of Price and Pearson (2013) and Price and Mattingley (2013), in the suggested involvement of the phonological loop in concurrent generation and by demonstrating that the apparent automaticity of our participant's experience is graded, or only has the appearance of an involuntary feel.

### Visuospatial memory and persistence of concurrents

Once induced, AB will continue to experience a concurrent even when direct attention is drawn away from it. Similarly, Jarick reported that her subject (L) experienced a persistence of induced concurrents until they were “not needed any more” for the purposes of mental viewpoint navigation (Jarick, 2010, p. 88). Although this finding of persistence has also been identified via more commonplace first-person methods (e.g., semi-structured interviews), the level of detail made available by EI is unparalleled thus far. Specifically, whilst Jarick refers to persistence until concurrents “are not needed” (Jarick, 2010), our data suggest that the development of this persistence is based on sustained exposure and focus on the concurrent, which may have implications for the development or encoding of spatial forms in terms of visuospatial imagery and memory. We also find that AB experiences a sense of “presence” when he cannot directly see a concurrent but knows where it should be in relation to others. These two features suggest involvement of visuo-spatial memory processes in SSS, where the memory of a concurrent position is sufficient to hold the synesthetic experience in active working memory, either as a visual experience or as non-spatial visual content. This feature may be relevant to the development of SSS in individuals, as it may enable the maintenance of a visual representation when ordinal sequences are being taught. Brang et al. (2010) and Simner et al. (2009) have reported superior visuo-spatial memory abilities in SS synesthetes, as demonstrated in their ability to learn new spatial forms (Brang et al., 2010) and perform above average in assessments of visuo-spatial recall such as the Visual Patterns Test (Della Sala, 1997; Simner et al., 2009).

### Blind analysis extends previous introspective reports

Our results extend standard introspective reports collected elsewhere. For example, 19 of the Seron et al. subjects report that, once activated, a concurrent could not be moved, supporting our findings on the persistence of concurrents and AB's necessity to generate a new representation of the concurrent rather than modifying an original. Subjects also reported being better able to “make use” of their representation where they had a free “visual field,” e.g., when their eyes were closed. This statement is compatible with our findings of physical interference, where AB's concurrents were placed in areas of an uninterrupted visual scene in the mental room, so as to avoid conflict with items in the physical room which hold more salience than the mental room percepts. As with our subject, Seron et al. also report that for his subjects the “vividness” of the number concurrent increases when the subjects concentrate on it (Seron et al., 1992, p. 174). Through the application and analysis of the EI data collected here we have been able to identify that this concentration is experienced by the subject in many cases as internal recitation of the inducer name, highlighting the importance of inner speech in the induction process. One of the Seron et al. subjects also reports to automatically experience the concurrents immediately surrounding the induced item, as with our subject.

### New insights may be used to direct future qualitative and quantitative investigations

The present findings suggest new opportunities for future investigations of SSS and associated synesthesia phenomenology. For example, quantitative investigations should consider the interference of physical objects in the perception of synesthetic concurrents and associated limitations of visually presented inducers.

Early self-reports from SS synesthetes described the experience as occurring involuntarily and automatically (Cytowic, 1989). However, our subject reports that he is not continually “swamped” with concurrents on a daily basis, despite continually reading and seeing numbers and letters in his external environment. Indeed, a pilot investigation with our participant, using the Descriptive Experience Sampling method (Hurlburt, 2009; Hurlburt et al., 2009), captured a moment when AB was actively engaged with letters and numbers on a computer screen, but he reported no awareness of any synesthetic experience. As such, the perception of a concurrent is not entirely automatic for AB, in line with evidence that automaticity of concurrents can vary in grapheme-color synesthesia (Rothen et al., 2013). We have also noted specific instances of where AB does retain voluntary control over his synesthetic experience. These examples support the challenge to the assumption of automaticity in SSS, as suggested by Price and Pearson (2013), and the issue of automaticity in SSS may be a topic usefully explored with EI across multiple subjects. For our subject, the concurrent was generated through internal verbalization; it remains to be seen, with the further application of EI or other procedures, how much detail can be obtained regarding the apparent automaticity of concurrent perception. The role of visual imagery in SSS could also be investigated further (Price and Pearson, 2013). Specifically, the experience of “unstable” concurrents could be investigated in synesthetic participants, and compared qualitatively with descriptions of “unstable” visual imagery in non-synesthetic participants^3^.

The phenomenology presented here is by no means exhaustive. A number of unresolved issues are immediately identifiable, such as the role of inner speech and focused attention in stabilizing a concurrent. We may ask how inner speech impacts mental imagery, and whether non-synesthetes employ similar methods of inner speech as observed here. We also demonstrate that the apparent automaticity of SSS can be investigated with this method in terms of phenomenological experience. Price and Mattingley (2013) have recently argued that SSS does not meet the necessary behavioral criteria to be termed truly automatic. Indeed, we have determined here that although synesthetic concurrents were initially described to arise automatically, in the majority of cases, our subject went through a process of internal verbalization of the inducer name before the concurrent was perceived. This may relate to the task-specific strategies suggested by Price and Mattingley (2013) to mediate the cueing effects of SSS. Further exploration of the diachronic and synchronic structure of the spatial cueing effect may help determine the causal relationship between the appearance of the concurrent and the shift in spatial attention.

## Conclusions

Phenomenological descriptions of SSS have so far been largely confined to descriptions of spatial locations pertaining to specific inducers (e.g., numbers, letters, calendar date). Here, employing a second-person method designed to increase the reporting access to pre-reflective aspects of experience, we have provided a comprehensive description of our subject's spatial form for a range of ordinal sequences including number, calendars, alphabets and ages, and extend the depth and breadth of relevant phenomenological information which has been acquired for SSS, or indeed any other form of visual synesthesia. We also introduced new associated synesthetic experiences such as concurrents for graphs, equations and mathematical concepts. Using the formalism of Grounded Theory analysis, we identified seven main themes in AB's SSS experience, as below.

1. Form descriptions
2. Physical and mental rooms
3. Concurrent contexts
4. Inducer-concurrent relationships
5. Perspective taking on the concurrent
6. Stability of the percept
7. Voluntarily controllable acts

Some of these themes have been described elsewhere (e.g., Seron et al., 1992; Jarick, 2010), however, these sources provide only limited information the first-person experience of the subject. As such, the present results provide a unique level of detail which may be beneficial in devising future investigations.

We report the first application of the EI method to SSS, and indeed to synesthesia more widely. We demonstrate that it is possible to gain informative second-person descriptions of synesthetic phenomenology from a naïve subject, through rigorous data collection and analysis, and demonstrate the value of the EI method in enabling access to subtle aspects of the experience. We gained unprecedented detail regarding the precise synesthetic phenomenology of this subject, which may be generalizable to a wider population. We also provide access to interview data as raw transcripts, alongside the structured and analyzed form in the Supplementary Material. This is a rich data source and we invite the interested reader to explore avenues for further connections between the themes uncovered here and other aspects of SSS, or synesthesia more generally. Appreciation for the precise phenomenological experience in synesthesia should focus future research endeavors, as we seek to understand the extent and limitations of the experience from the perspective of the participant. Such work may transform consciousness science by bringing objectivity to experiential phenomenology.

## Author contributions

Cassandra Gould, Tom Froese, and Anil K. Seth designed the research. Tom Froese performed interviews with participant Adam B. Barrett and Cassandra Gould analyzed data and wrote the paper. Anil K. Seth and Jamie Ward supervised the project. All authors contributed to the final version of the paper.

### Conflict of interest statement

The authors declare that the research was conducted in the absence of any commercial or financial relationships that could be construed as a potential conflict of interest.

## Footnotes

^1^Throughout the Materials and Methods and Results sections, we have made use of direct quotations from interview transcripts, in order to convey the essence of the interview conduct and mode of questioning. Quotations are referenced with the interview date in the format YYYYMMDD and the paragraph number in the transcript.

^2^Note that term “projecting” was also bracketed in the earlier example (1. Maintaining the subject's focus) as the interviewer pursued a line of questioning relating to the known object of the “screen.”

^3^EI could also be usefully applied in elaborating the phenomenological experience of “vividness” of visual imagery, which is central in ratings of visual imagery, e.g., VVIQ (Marks, 1973).

## Supplementary material: HTML expandable mind map

Online supplementary material contains the full set of raw interview transcripts and descriptions of all themes uncovered during EI with this subject. Each theme can be explored by clicking the expand (+) button, revealing a summary description and further sub-themes. The bottom-most level contains verbatim excerpts from the in EI sessions, along with a interview date and paragraph reference.
