# Supplementary material for: An extended case study on the phenomenology of sequence-space synesthesia
Source: Front Hum Neurosci. 2014 Jul 3;8:433. doi: 10.3389/fnhum.2014.00433 (PMC4080762; doi:10.3389/fnhum.2014.00433)
Supplement: Supplementary file 1 [file Presentation1.ZIP › legends Supp Mat.pdf]

Supplementary material: Html expandable mind map

Online supplementary material contains the full description of all themes uncovered during EI with this subject. Each theme can be explored by clicking the expand (+) button, revealing a summary description and further sub-themes. The bottom-most level contains verbatim excerpts from the in EI sessions, along with a interview date and paragraph reference.
