# Supplementary material for: An extended case study on the phenomenology of sequence-space synesthesia
Source: Front Hum Neurosci. 2014 Jul 3;8:433. doi: 10.3389/fnhum.2014.00433 (PMC4080762; doi:10.3389/fnhum.2014.00433)
Supplement: Supplementary file 1 [file Presentation1.ZIP › Supp Mat 1.ZIP!/ExtendedCaseStudyOnThePhenomenologyOfSpatialFormSynaesthesia-SupplementaryMaterial.html]

Extended case study on the phenomenology    of spatial sequence
synaesthesia:
   Supplementary results and discussion


All +
All -


Extended case study on the phenomenology

of spatial sequence
synaesthesia:

Supplementary results and discussion

- + -


  Perspective on percept

  - + -


    Concurrents which are directly induced are generally presented in front of AB in the correct perspective, i.e. 'facing' although there is one report of a concurrent being turned away slightly (20100415\_185-187). New concurrents may also appear at some distance particularly if they are a new representation of the original concurrent. AB is able to view concurrents from different perspectives if for example he moves around them, although the change in perspective is not continuous.

    - + -


      Centre of focus

      - + -


        The concurrent of interest is usually presented directly in front of AB. From this perspective he is able to focus directly on different areas of the percept.

        - + -


          fractionated

          - I wasn't really aware of the whole number five at, at the same time. So, I sort of look at the top of it... and I look at the bottom (20100222\_255)


            I can focus on the individual lines of the B. I mean if I really focus on it. Now it's like it's made of ribbons attached to each other. (20100408\_329)

          so now I'm kind of seeing the five right in front of me but the line is as I said it's kind of, erm, yeah it's below, I have to look down, down in front of me to see the line, the number five is right in front of me. (20100215\_170)


          So this time I'm still very close to the five and now I've kind of turned my whole head to look at it. INTERVIEWER: So if you look at it from this new vantage point, how far would you say it is? AB: So it's right here. INTERVIEWER: Okay so, maybe half an arm. (20100222\_209-212)


          it's hard to see from where I am now, because I've got this... got this big back five sort of looming right there. (20100222\_170)


          so I've sort of got the number five to the right of my focus now, and kind of in front of my focus. (20100222\_33)


          it's kind of like, I'm kind of in the four. (20100222\_315)


          So I still caught a glimpse of 55 and 56 kind of fairly small over there, so I've got to see if I can bring it right in front of me. I can see it on a page.  (20100229\_392)


          ...while I was talking about it [56] the 1 9 is a bit more in the background and the 5 6 is a bit more here closer to me. (20100320a\_242)


          INTERVIEWER: ...What if you position yourself at the 0. Can you do that, can you see the 0? What numbers do you see next to it? AB: So there's a 1 here and a -1 there. INTERVIEWER: ...So can you look at the 0 coming from the positive numbers? AB: Looking here, so kind of 1, 0. INTERVIEWER: And what about from the negative numbers? AB: Yeah, 0, -1, -2, 0 is there. INTERVIEWER: So it's equally easy to go both ways?... AB: Yeah, I mean I have to kind of move. Sometimes I can do it with just sitting still, I can shift perspective. (20100408\_396-403)
      + -


      At a distance

      - + -


        New concurrents can also appear at some distance from AB.

        - Well it seems like I only have two perspectives, one was like... it's very hard to get in between the two, two positions. The five's all the way over there now. (20100222\_176)

          + -


          The five has got smaller now, it's kind of in the distance. (20100222\_150)

          - I can't see behind the five. (20100222\_152)
          + -


          INTERVIEWER: So the zoomed out version of the alphabet disappeared when you switched your attention to the A? AB: ...it's kind of in the distance. It's probably further away from me than I can see. (20100329\_280-281)

          - INTERVIEWER: What do you see when you look at the letter A? ... AB: ...it's kind of in the position of where the A is in that alphabet that is far way. (20100329\_272-285)
      + -


      Directional perspective (e.g. back to front, top view)

      - + -


        AB is able to view concurrents from different perspectives, e.g. if they are placed behind him (20100329\_179-183) or if he physically moves round or above them (e.g. 20100408\_113) . On one occasion AB reports that the concurrent is not directly facing him but is turned at an angle away (20100415\_185-187).

        - Still like a five, but, still thinking of it as five, but then I suddenly realise that five doesn't go that way round. ... And then it kind of went back. (20100222\_257-259).


          And from my perspective now, four's kind of the wrong way round. Well I mean yeah, it's kind of sort of so it would be the right way round if I was standing sort of over there, but the four's kind of there and I'm here, so... see what I mean.  (20100222\_303)


          sometimes the bottom of that five is kind of the wrong way, err the curve goes the wrong way.  (20100222\_321)

          + -


          I can sense a 3 behind me. INTERVIEWER: Can you describe that sense? AB: It's almost like I have an eye at the back of my head and I can see the 3 there. So it's as if there's an eye here and the 3 is down there. ... AB: So, it's in the corridor on a brown floor. And from my perspective a 3 kind of looks back to front. So if you were looking at it from...(20100329\_179-183)

          - Now it's all kind of spinning around, more confused. Yeah, I feel it's a back to front 3 from my perspective. INTERVIEWER: So what happened there, were you trying to arrange it differently? What was confusing you? AB: I was a bit confused as for a moment I was imagining being in front of the 3, then it started spinning around. I was like, "hey, that looks like a back to front E" and then I was like, "but that is a 3". (20100329\_189-191)


            I can kind of see the 2 and the 1 as well. Yeah, it's really like I got eyes in the back of my head. (20100329\_193)
          + -


          ...I kind of can't see it, can see it only side on. Erm, sometimes it tilts a little bit so it's kind of bi-stable. (20100408\_103)

          - INTERVIEWER: Can you [physically move round to look at the percept from different angles] do that to the number? AB: I can see the back of the 4. Yeah I can kind of see the 4 backwards. Sometimes it sort of spins around.  (20100408\_104-105).


            So it's always kind of bi-stable, but yeah there's kind of one where it's kind of back to front. Because I see it orientated with the front, this side. (20100408\_107).

          I can see a bit more of the 4 than I should, like if I'm right above I should just see it all black and not see the other bit in the middle at all. I can see more of it than I would expect. INTERVIEWER: So you can still see that's it's a 4, even though from the top it should be just a black line really. <AB agrees> AB: But it does kind of open up wider when I come in front. INTERVIEWER: ...How did it open up a little bit wider?  
          AB: It's like the hole is bigger. (20100408\_113-117)


          INTERVIEWER: ...So if you look at it from the top and keep looking at it when you sit down again? AB: ...it doesn't really seem to change continuously. (20100408\_122-123)


          INTERVIEWER: So it seems like there are some preferred angles from which it can be viewed. Is that an accurate description? AB: Yeah. So it kind of moves a bit, in order to give me that angle. It's almost like the 4 just bends back there. (20100408\_130-131)


          INTERVIEWER: ...[during the hypnosis session where you were floating above the line] did the numbers look as if they were [being viewed] from the top or were they presenting themselves as if you were looking at them from the front? AB: ...I was looking at them from the front. But they kind of turned around for me. (20100408\_414-415).

          + -


          INTERVIEWER: ...So how does it change when you stand up for example? AB: ...looks like I am kind of above it, kind of like this. It's kind of got some depth to it this time.  (20100415\_68-69)

          - Seems to be pretty much vertical. I mean if anything, I just sort of see a thick line there where 2 should be. (20100415\_79)


            I am also seeing a 2 like that [flat, horizontal, looking at it from above] as well simultaneously.  INTERVIEWER: Oh, like the same spot, as if it was lying on the table? AB: Yeah. INTERVIEWER: So when did that one appear? AB: Just now. And I was trying to work out what I was seeing. It gets a bit fuzzy when I'm standing directly above it. (20100415\_81-85)


            It's sort of different images coming into my mind. One is just of erm the top of the 2. Another is it with it slightly angled. Then another is the 2 just flat on the table. INTERVIEWER: Right. So at least three different kinds of fitting here. AB: Yeah. That's just sort of less stable. (20100415\_87-89)


            So, I've got a 2 flat on the table now. Then I've got the other one is kind of there standing up and flicking between the two. (20100415\_91)


            INTERVIEWER: ...If you just focus on the one that's standing up and then just rise up while looking at it. What happens to it? AB: Yeah, it seemed to jump for a moment like flat on the table. And then sort of comes back and is standing up. (20100415\_96-97)


            but then it kind always come back up again.  (20100415\_103)


            INTERVIEWER: ...was the one flat on the table present the whole time... when you were standing up and down?  AB: No, it just came into view when I sort of reached there at a certain angle. (20100415\_106-107)


            INTERVIEWER: When you were ...focusing on the vertical [2], did it [the vertical 2] at any time disappear and then the other [flat 2] one [appeared]? AB: Yeah, when I got to a certain height. INTERVIEWER: And then when you were at the top basically stood up, the standing up one came back. AB: Yeah, yeah and then continued to alternate with the... INTERVIEWER: And then both of them . AB: Yeah. (20100415\_114-119)

          So this one is directly facing me and that one is kind of facing that way. So I'm kind of slightly sideways on. ...  if you are in the chair one to your right would face you. (20100415\_185-187)
  + -


  Voluntarily controllable acts

  - + -


    AB is able to perform a number of acts to modify his synaesthetic experience under his own control or volition. These include:

    1. Changing perspective of a concurrent either by physically moving around the room or adopting a different mental perceptual position.
    2. Induce concurrents by thinking about them, looking for them in relation to other concurrents or through the internal recitation of the concurrent in his head (inner speech).
    3. Perform a context transition to the number line by directing his focus of attention downwards towards where the number line should be.
    4. Overcome physical interference in the mental room through effortful 'willing' of the concurrent to appear.
    5. Evoke a visual memory of a concurrent without evoking the concurrent itself.
    6. Manipulate the corridor context to change the lighting through the addition of a "sunroof".

    We also find that although AB's reports would suggest that he can in some cases modify a concurrent itself, attempts to do so lead to the generation of a new concurrent with the desired characteristic whilst the original remains unchanged.

    - + -


      Change perspective

      - + -


        AB is able to voluntarily change his perspective of a concurrent, either by physically moving his own position in space, e.g. by standing up or looking around a concurrent, or conceptually adopting a different perceptual position. In most cases, physical movement appears to assist the change in perspective, which is otherwise described as effortful. AB also reports an ability to move within the corridor context with such ease that it is instantaneous (20100222\_154) and 'zoom' in or out to direct his attention towards a detail or larger portion of the sequence.

        - + -


          Move within the corridor

          - + -


            AB is able to move himself (20100222\_140) or his head (20100222\_209) within the corridor to view a concurrent from a different perspective. AB describes this movement as "instantaneous" (20100222\_154) and is not able to describe how he performs the movement.

            - + -


              I can bring myself along the corridor (20100222\_140).

              - instantanious (20100222\_154, 160)


                wiggled (20100222\_154)


                moved my eyes (20100222\_160)

              turn whole head within corridor (20100222\_209)


              Yeah I can bring myself along the corridor (20100222\_140)


              So I've kind of moved along the corridor and the five is now to my left.(20100222\_142)


              INTERVIEWER: Okay so just coming back to the movement, can you move back again into the original position? AB: Yes, it's a fairly sort of instantaneous movement. I don't really know how I've got there. Just kind of wiggled it I guess. (20100222\_153-154)


              INTERVIEWER: Once again, let's try it again. So if I say to you, “move to your right”, what happens? AB: Yes I've already done it before you said that. (20100222\_155-156)


              Ahm, it's kind of hard, erm, I come along, ahm, it's kind of stuck against the wall there. (20100222\_303)


              INTERVIEWER: So you can move in relationship to these metallic numbers, but you have difficulty of moving them around? (20100222\_310)
          + -


          See things from a different directional perspective

          - + -


            AB can manipulate his view of the concurrent for a different perspectives. For example, he reports to "rotate the image" of a section of the number line in order to "look back from one hundred" (20100229\_342-345). AB is initially unsure how to achieve these manipulations, suggesting this may not be an action which he regularly undertakes, but is ultimately successful.  
              
            AB is able to move an interact with concurrents as if they are externally fixed objects in space. On an occasion where AB is directed to turn away from the concurrents and then look back at them, he reports that the concurrents continue to occupy a constant position in the physical room, independent of his directed gaze (20100305\_131-132). When AB stands to view a concurrent from above he initially perceives the concurrent from a 'birds-eye' perspective (20100305\_101-103). AB reports that this physical movement makes it easier to achieve the change in perspective of the concurrent rather than attempting a mental rotation (20100408\_403-405). These interactions may suggest that the concurrents are dissociated objects in space and exist somewhat independently of the subject, that is they are automatic rather than consciously generated.

            - still a certain amount of autonomy as percept reverts to dominant perspective (20100222\_259)


              can do it from sitting still, but physical movement helps (20100408\_403-405)


              INTERVIEWER: ...if you take them out of view completely so if you look behind you so immediately you don't see either of them and then you turn around again. Do they remain exactly where they were before? AB: Yeah still 5 6 57. I sense a 54 here, but weaker this one though. (20100305\_131-132)


              [If I] Stand up. INTERVIEWER: Has their location changed at all? AB: Yeah, I'm kind of more above them now. (20100305\_101-103)


              Yeah, I mean I have to kind of move. Sometimes I can do it with just sitting still, I can shift perspective. INTERVIEWER: But sometimes movement helps? AB: Yeah, movement is definitely easier. (20100408\_403-405)


              (20100222\_257)


              So this time I'm still very close to the five and now I've kind of turned my whole head to look at it.(20100222\_209)


              INTERVIEWER: So if you change your perspective so you can see the 56 a little bit closer. Maybe you can put it in front of you.  AB: So I don't know how to do that <quiet> <Laughter> Maybe I can start again. ... Yeah by the time you start with a clean slate and then you get bogged up with loads of numbers! (20100229\_377-384)


              INTERVIEWER: ...So when you want to look at the 6, you actually switch perspectives. AB: Yeah.  (20100329\_14-15).


              INTERVIEWER: Ok, so what if I said can you please look at it from the other side, I mean looking from the 100 onwards down the number line. Can you switch your perspective? AB: So look back from 100. It's tricky, please stabilise...<quiet> I'm kind of imagining behind the line. So the line is kind of like that  <quiet> INTERVIEWER: So what did you have to do in order to change your perspective? AB: Erm, kind of rotate the image in front of me. So it's kind of over there and I kind of feel a 100 here and 101 there. Erm, yeah all the other numbers are kind of going down.<pause> 90 is over there. And erm, I can kind of see this line that sort of zig zags around. (20100229\_342-345)
          + -


          Change focus of attention

          - + -


            AB is able to voluntarily direct his attention to different concurrents or individual features of a single concurrent. AB often refers to this a 'zooming' where he brings a concurrent into view. Zooming appears to effect the size of the concurrent (zooming out makes the concurrent appear smaller) whilst the context such as the white page remains the same size.

            - + -


              look at different parts/areas of the percepts in  detail

              - also described as "zooming" (20100229\_41)


                (20100222\_225)

              INTERVIEWER: So can you easily switch your attention between the 2 in front of you and the 2 over there? AB: Yeah. (20100415\_182-183).

              + -


              ...I was kind of focusing on the five, when you were s..., when you said four, so I kind of erm, erm, kind of made me shift my attention, down the line, down, down and to the left down the line... the number four, appearing sort of at the bottom left of my field of vision there it's just sort of on the edge, and it's like. It's like when I'm looking at the external world and I'm trying to paying attention to something that's erm that's sort of just on the edge of my visual field. The four - you can only just see it but there's a black, you can see there's a number four there, there's a black four quite thick ink again, and again just kind of above the line, above the... the... the number line. (20100215\_178)

              - It's kind of moved a little bit now, now erm cause we're talking more about four than five, so it's kind of I feel it... My, my perspective has changed a little bit and it's kind of, I've got four and five are fitting a bit more clearly into the frame now. (20100215\_200)
              + -


              zooming

              - + -


                zooming in changes focus of attention

                - If I zoom in close enough, if I pay attention (20100320b\_42).
                + -


                zooming out makes the numbers smaller whilst the context stays the same size

                - + -


                  making more space for number to fit into the frame (20100215\_213).

                  - the numbers became smaller the frame stayed the same size (20100215\_215)

                  keeps zooming out, doesn't ever reach the edge of the page (20100215\_220)


                  (20100215\_205)
      + -


      Induce concurrents

      - + -


        AB is able to voluntarily induce concurrents by thinking about them, looking for them in relation to another concurrent or saying the name of the inducer using inner speech\*\*. there is a suggestion thar both 'thinking' about and 'looking for' a concurrent also involve inner speech in saying the name of the inducer e.g.  "I kind of just thought of the letter Q by saying it in my head, then I kind of looked for it" (20100408\_51).

        - + -


          Look for concurrent by changing direction of gaze

          - + -


            AB is able to directly induce concurrents by changing his direction of gaze and looking towards where a specific concurrent 'should' be. AB suggests that the concurrent appears as a result of his directed gaze, for example, "I was... immediately aware of the H... where I put my gaze" (20100408\_43) or "[I make] little eye movements across to read the other numbers" (20100320a\_82).

            - little eye movements accross to read the other numbers (20100320a\_82)


              I move my attention down, so literally have to move my eyes (20100320a\_92).


              Z? Just have to relook where it is (20100408\_43)


              number - I guess I kind of knew to look for them there (20100408\_91)


              I was kind of immediately aware of the H, just kind of where I put my gaze (20100408\_13).


              letter (20100408\_43)


              number (20100215\_220)
          + -


          Self verbalizations

          - + -


            AB is able to induce concurrents by saying the name of the inducer to himself using inner speech. It is probable that this inner speech proceeds the act of looking for a concurrent on more than one occasion, e.g. "I kind of just thought of the letter Q by saying it in my head, then I kind of looked for it" (20100408\_51) and "[I] just say "right I'm going to look for the 4 now." (20100408\_139). Further interviewing on this specific feature would be required to confirm the diachronic structure conclusively.

            - I kind of just thought of the letter Q by saying it in my head, then I kind of looked for it (20100408\_51)


              No, I think you saying it is enough. (20100408\_47)


              Just say "right I'm going to look for the 4 now" (20100408\_139). There's like a decision (20100408\_141). an intention sort of before (20100408\_143).
          + -


          Thinking

          - + -


            AB is able to induce concurrents by simply 'thinking' about the inducer, for example, "I just faced this direction and then thought of 55" (20100305\_135-142). This 'thought' presumably involved inner speech.

            - INTERVIEWER: [can you] create a different number line... Maybe over there for example, if you say 55 over in that direction... AB: I just faced this direction and then thought of 55 (20100305\_135-142)
      + -


      Induce context transition

      - + -


        AB is able to voluntarily induce a context transition (e.g. from the white page to the number line or corridor) by directing his focus of attention to the number line or looking down to where the number line should be. AB reports that during this process he is thinking about where the concurrent sits in "relation to the other numbers" (20100305\_21), suggesting that the context transition involves a change in directional attention.

        - + -


          Eye movements direct focus of attention & enable context transition

          - + -


            AB is able to voluntarily induce a context transition by directing his focus of attention. For example, to perform a context transition from a white page to the corridor, AB simply has to look at the number line (20100329\_108-109). As AB reports to "shift my perspective down [from a white page five]..." we find that the context transition to the corridor has already occured, "...[now] I just have the four, five, six, much more normal" (20100329\_161).

            - + -


              let it melt (20100305\_21)

              - look down...it kind of wants to be behind a white background. But I can kind of make it pretty much go away.  (20100305\_75)

              When I shift my perspective down then I just have the 4 5 and 6 much more normal (20100329\_161).


              INTERVIEWER: ...eventually you can drop down the 6 into this sequence. <AB agreeing throughout> I was just interested, how do you do it? How can you make the two connect? Is there anything that you notice? AB: By looking at the number line. (20100329\_108-109)
          + -


          "Will" a transition

          - + -


            On other occasions ABs directing of gaze or attention does not appear to play such an obvious role in inducing a context transition, for example on request he is able to 'will' a transition (20100222\_218-219), 'make the number line appear' (20100229\_453-454) or "engineer [a context transition quite straightforwardly" (20100329\_89). However this may involve visualisation or directional attention e.g. where AB suggests that he can cause the white page to melt by starting to "think about where the fifty six goes in relation to the other numbers" (20100305\_21).

            - INTERVIEWER: Okay, so if you will it to come down, what happens? AB: Kind of crept down the wall, and there's like a small number six against the wall, and there's a line, joining the five to the six. Well it's not touching the five or the six but err.. (20100222\_218-219)


              INTERVIEWER: Can you make a number line appear? AB: Erm, yeah I can get flashes of it in between the numbers. There's a broken line and gaps for each of the digits. (20100229\_453-454)


              I can kind of let it melt away a bit from the bottom. Start think about where the 56 goes in relation to the other numbers. (20100305\_21)


              I can get a small 56 appearing briefly on the ground [with other numbers in the line] (20100305\_45)


              Erm, it seems like it's easier to appear on a surface. But then I kind of want them to appear in a space. I can kind of engineer that quite straightforwardly. <laughter>  (20100329\_89).


              INTERVIEWER: Can you make a number line appear? AB: Erm, yeah I can get flashes of it in between the numbers. There's a broken line and gaps for each of the ... numbers  (54-57) (20100229\_452-458).


              I can get it to kind to flicker in and out. Segments of the line joining the 5 to the 6 and 6 to the 7 (20100320b\_49).
      + -


      Overcome physical interference

      - + -


        If AB is directed to induce a concurrent where there is interference from an object in the physical room, he appears on occaision to be able to overcome that physical interference exerting cognitive effort which he describes as 'willing' a concurrent to appear. For example, when viewing a letter drawn on a page AB reports, "Well it just appears first away from the page. I have to will an alphabet on the page in order to get it to happen." (20100408\_75). This concept of 'willing' is not investigated further, but we may speculate that it involves processes of induction, such as a focused attention achieved by inner speech.

        - + -


          'willing' the concurrent to appear.

          - [whilst viewing a letter drawn on a page] I have to will an alphabet to appear (20100408\_75)


            Well it just appears first away from the page. I have to will an alphabet on the page in order to get it to happen. (20100408\_75)
      + -


      Make comparisons between percepts based on visual memory

      - + -


        When requested to compare features of different concurrents, AB is able to recall the details without having to (or automatically experiencing) the concurrent itself. Instead, he reports to have a visual memory of the concurrent, as a distinct experience from the concurrent itself (20100415\_203-211).

        - INTERVIEWER: When you said that just now to me [describing the patterning of a replicated 2], you didn't actually look there did you? Were you able to somehow recall it while looking at me? AB: Yeah. INTERVIEWER: How did you do that? AB: Oh just recalling what I saw over there. INTERVIEWER: Oh ok. So it's not like you had a visualisation while you were talking to me? AB: ...I was just remember what was going on over there. INTERVIEWER: Ok, but it was a visual memory? AB: Yeah. <Pause> INTERVIEWER: Ok, because I noticed that several times today you were able to describe in quite precise detail the textures and colours and the different comparisons between the numbers. And I expected you to actually look at one of them and look at the other back and forth to make the comparison, but most of the time you were looking at me directly and still you know giving me quite precise details. But you didn't make any eye movements so it wasn't like there was like an imaginative comparison, I guess. <AB agrees throughout>. (20100415\_203-211)
      + -


      Change lighting

      - + -


        AB is able to change the lighting conditions within the corridor by introducing a "sunroof" (20100415\_326) opening spanning the width of the ceiling. This sunroof appeared when AB was attempting to describe the patterning or texture of a concurrent and he reports that, "I thought perhaps if you shone a really bright light on it then it might have a dark sort of, dark, shiny sort of metallic lustre to it" (20100415\_247). This thought process alone appears to have initiated the introduction of the sunroof.

        - + -


          introducing a light source

          - + -


            AB is able to change the lighting conditions of the concurrent and context for the purposes of examining the percept. For example in a section of the interview AB is attempting to describe the patterning of a concurrent. To do so he imagines another concurrent (of the same number) next to it. In comparing the two concurrents he finds that on might be shiny, "[I] thought perhaps if you shone a really bright light on it then it might have a dark sort of, dark, shiny sort of metallic sort of lustre to it" (20100415\_247). To explore this further he changes the lighting in the the corridor such that a square "sunroof" (20100415\_326) opens above the concurrent of interest to make it "much better illuminated" (20100415\_267) and appear as "kind of getting a bit of light on the top" (20100415\_322). The 'sunroof' itself is described as a square hole, in the ceiling of the corridor, going from one wall to the other as though "the roof has melted away" (20100415\_330). AB refers to this change as "we've introduced this light up here" (20100415\_322). Thus is appears that the context may be modified to a certain degree by AB's intentions rather than being purely automatic.

            - Erm, kind of like a sort of sun roof has opened up in the corridor. (20100222\_326)


              Ahm, it's just like a white hole. (20100222\_328)


              No it's just err, it's like a square hole? It goes from one wall to the other. Ahm The whole corridor's becoming a bit less stable now, it's kind of err, the roof from this point onwards has just kind of err melted away. (20100222\_330)


              we've introduced this light up here. (20100222\_322)


              yeah, it's black, it's black. I thought perhaps if you shone a really bright light on it then it might have a dark sort of, dark, shiny sort of metallic sort of lustre to it, but erm... it's quite sort of vague... INTERVIEWER: Dark, metallic and shiny? ...how do you know that it has this quality? AB: Just from imagining the light shining on it from above. AB: Erm, I think I was just envisioning another number five, that was kind of here. And it had a kind of dark grey colour to it, just like kind of metallic... Yeah when I compared the two, a light kind of started shining on the actual five that was down here... Ahm, it's kind of different because the one that appeared here was much better illuminated... The black at the top is kind of much darker, so there's a little bit of white light on the top now, because we've introduced this light up here. So.. So it's kind of shining, kind of getting a bit of light on top...INTERVIEWER: So the light that's shining on that five actually has a source? AB: Yes, it's up here. INTERVIEWER: Is it in the corridor, or... AB: Erm, kind of like a sort of sun roof has opened up in the corridor. INTERVIEWER: So, what does it look like, the point where the light crosses into the corridor? AB: Ahm, it's just like a white hole. INTERVIEWER: A round hole? AB: No it's just err, it's like a square hole? It goes from one wall to the other. Ahm The whole corridor's becoming a bit less stable now, it's kind of err, the roof from this point onwards has just kind of err melted away... Erm, kind of like a sort of sun roof has opened up in the corridor. (20100415\_247-330)
      + -


      Generate multiple representations

      - + -


        AB's reports suggest that he is able to manipulate concurrents e.g. to change the representation of a fraction, change the case of letters, move the concurrents to change his perspective or change the context of the concurrent. When the details of this manipulation are investigated further, we find that rather than changing the concurrent itself, AB generates a new concurrent with the desired characteristic. This generation of multiple representations of a concurrent might suggest that AB has a preferred or default form which they are initially presented in and once apparent the features of this form cannot be modified.

        - + -


          Attempt to change between common and decimal fraction representations leads to two seperate concurrents

          - + -


            When investigating concurrents for fractions, AB is asked if he can "change the representation of the fraction" 1.5 (20100229\_22). Here reports the experience of the fraction in the common form of a numerator and denominator suggesting he is able to change the representation of a concurrent at will. Note however that the new representation appears in a different (although overlapping) space and the original decimal representation remains rather than being 'morphed' into the common notation.

            - INTERVIEWER: Can you change the representation of the fraction? AB: Yeah I can see there is 3 over 2 (20100229\_22-23)
          + -


          Attempt to change case of letter forms leads to two seperate concurrents

          - + -


            When investigating ABs alphabet form, he is asked if he can change the font of the letters from upper case (his usual form) into lower case (20100329\_322). AB reports that he can "get the lower case A to flash" (20100329\_323) in the same place as the upper case A, but the lower case A is initially not stable "[it] doesn't stay there" (ibid). The lower case form later stabilises such that each uppercase letter has a lower case version next to it (20100329\_329-333). Here again that rather than modifying the original concurrent, AB has generated a new form sequence with the requested features.

            - INTERVIEWER: how easy is it to change the big A into a lower case A? AB: I can get the lower case A to flash, but then doesn't stay there. INTERVIEWER: Above it or it's replacing? AB: Same place.<pause> (20100329\_322-325). ... black capital A and then a lower case A to the right of it (20100329\_331).


              Now I've got the B next to it, down and to the right of the two A's. Capital B, small B. INTERVIEWER: Oh so it's a different kind of sequence, its capital letters, lower case letters, capital letters, lower case letters (20100329\_333-334). AB: So there's the A with the erm, capital A and the lower case A next to each other were kind of on a white paper with gridlines on it (20100329\_347)
          + -


          Attempt to change directional perspective leads to two seperate concurrents

          - + -


            AB is able to view concurrents from different perspectives or perceptual positions. When he attempts to do so under his own volition, it is often evident that rather than moving the concurrent itself, AB spontaneously generates a new concurrent upon which he has the desired perspectival view. For example, AB reports that he can "bring [the numbers 54-58] over here" (20100305\_109) with the intention of wanting "them to be a bit closer" (20100305\_133). Subsequently we find that there is now a new set of numbers in the desired location and the previous set remain as they were (20100305\_117-119).

            - The numbers [54-58] are over there. I can bring them over here. INTERVIEWER: Ok, can you do that?  
              AB: So I've just brought them over there. INTERVIEWER: What did you do in order to do that?  
              AB: Erm, dunno. Just wanted them to be a bit closer <laughter> (20100305\_109-113)


              So I've got one lot over here and one lot over there. INTERVIEWER: Are there still remains over here? AB: Yeah. But look over there, there's some numbers over there. (20100305\_117-119)
          + -


          Attempt to perform a context transition leads to two seperate concurrents

          - + -


            When AB reports to perform a context transition, e.g. 'moving' a free space concurrent (6) into the correct position in the number line (between a 5 and 7) (20100329\_17), we find that the concurrent is "kind of in both places at once" (20100329\_19). It appears that rather than the same concurrent 'moving' to a new location a new representation of the same concurrent is generated in the desired context and the concurrent in the free space context remains at least partially visible (20100329\_19).

            - No, it's [the 6 is in] some kind of floating space [free space(20100329\_13)], then I can bring it down here [into the correct position between the 5 and 7 on the number line]. Erm. INTERVIEWER: Is it down there now? AB: Yeah, it's kind of in both places at once. It's kind of getting more down here than up there now. For a while it was in a position between the two. (20100329\_17-19).
  + -


  Stability of percept

  - Some concurrents appear to be generated with certainty and little
    effort, particularly those which AB is familiar with. He is able to
    induce these concurrents with ease by directing attention to the
    location in the mental room which the concurrent should be located, with
    respect to existing concurrents. Directing attention to a concurrent
    through changing gaze direction or verbal reporting improves the
    stability of the percept and AB's ability to provide detail on the
    experience. Focused attention and suggestion may also lead to the *de
    novo*\*\* induction of a concurrent. The ability of attention to
    modulate the conscious visual experience of the subject should be
    investigated further.

    'Instability' is often reported where AB is attempts to modify a feature
    of the concurrent or context, resulting in a brief appearance or
    flashing of the percept. These unstable percepts generally stabilise as
    AB continues to direct attention towards them. Interestingly, patterns
    on the concurrent and background colours do not appear to to stabilise
    into static images and a consistently described with a sense of change.

    - + -


      Knowledge

      - + -


        AB has a concrete knowledge of where in space his concurrents are located (the defining feature of spatial sequence synaesthesia). This knowledge enables him to locate one concurrent with respect to another by directing his focus of attention to that space. Where ABs knowledge of a number sequence is limited (e.g. in the decimal places of pi), we find that he only experiences stable concurrents for those numbers of which he is certain.

        - + -


          "I know where to look" to locate a concurrent with respect to another

          - + -


            We may assume that through a life time of exposure to his synaesthetic forms, AB has gained a sophisticated knowledge of where in space each should exist in relation to another. This enables AB to locate a concurrent with ease simply by directing his attention to the proper spatial locations.  "INTERVIEWER: But you said they only appear when you look in their direction. AB: I just know they are there. I know where to look" (20100329\_254-255).

            - INTERVIEWER: Well I mean, if you let your gaze move a little bit more freely, how easy is it for the other letters to appear? AB: It's pretty easy for H I J K L M N O P Q to appear. R S T. All I have to do is move my attention along the thing. I can kind of see the whole alphabet if I want to.  INTERVIEWER: But how do you know where to look, if there's no line connecting them? AB: Because the letters are just there. INTERVIEWER: But you said they only appear when you look in their direction. AB: I just know they are there. I know where to look (20100329\_250-255).
          + -


          Insufficient knowledge of a sequence leads to an unstable percept

          - + -


            It appears that AB's direct knowledge of a numerical sequence curtails his ability to perceive the correct concurrent. After five decimal places of pi, the concurrents become unstable and he reports that any further numbers which he can make out with effort exist as concurrents but are random and not related to the decimals of pi (20100229\_132).

            - + -


              pi

              - So now I've got 3.14159 dot dot dot 8656 and then a whole pool a whole load of numbers just kind of like jumbled up like kind of just there not really knowing where to go. (20100229\_147)


                2, 7. I'm not even sure if these are right now but I am seeing 2796 too. And then just some kind of vague numbers just kind of going off and I can't. I'm not really sure what they are because they are sort of an 8 and then another 6 2. Erm yeah, seems a bit unstable  ... So it's unstable after 3.141527986. Seems pretty unstable after the third or fourth decimal point. ... INTERVIEWER: Ok, so just curious. What does unstable look like? AB: Erm <long pause to think>  So the focus on one number. The one to right is kind of just changed. Because it's not really holding still it's kind of changing because of other numbers trying to get in from above. ... So it's 3.14 and 1 and a 5 and that's where I start getting difficulties. Next to the 5 there's like I say a 2 to the right of the 5 but there's a 9 above the 2. INTERVIEWER: How is it above the 2? AB: Just directly above on the page. INTERVIEWER: Is it stable up there? AB: I see a 1 a 2 and a 9. I see a 7 to the right of the 2 and the 9, slightly above the 2. I just see a jumble of numbers. When I am focusing on that region now the pihas gone.  I've just brought that back by saying that <pause> pi.uals 3.1415926. Yeah and the 9 to the side of me, just kind of still there. Just kind of not really working themselves out into anything coherent. ... Yeah so as I focus on it, it becomes stable. So it's like, so it's almost like I have to fill it in with something. But they just seem to be random numbers. (20100229\_71-83)


                Sort of floating there. I'm sort of seeing a dot, dot, dot and then sort of. There seems to be a rotation where it's starting to go off in that direction away from me.  (20100229\_99)


                I can see 3 and then I can see pi.uals 3.1415 9. Now the 2 is above the 9. They are still not stable <laughter and cross-talking>. And then there's some numbers kind of on the floor in front of the blackness which is covering the wall on the 9 and just kind of going off. (20100229\_116)


                They're just potentially numbers that could go into the sequence but they don't know where to go (20100229\_118)


                So there's just kind of lots of numbers but I can't really read them. I mean I can focus on erm individual numbers I can just see a 4 there and a 9. Well now I'm seeing a sequence 4 9 3 2 8 6 but it's like I see sequences of random numbers. Erm, well I just can't relate those to the beginning of 5. (20100229\_132)


                So I looked to the left of those random numbers I've just said and then I saw 3.14159 again. Then I seem to not be able to get back to the same numbers as I approach it again from the left hand side. (20100229\_136)


                And then I see dot dot dot . INTERVIEWER: So you actually see 3 dots in the corridor? AB: Yeah  (20100229\_139-141)


                Think the 9 has gone into place as I now know for sure that's the next digit and not the 2 that was kind of jostling for position. INTERVIEWER: Oh really! So what is the one after the 9 this time? AB: Don't know as it's not very clear. (20100305\_7-9)


                I think I'm just seeing kind of dot dot dot. And then there's, I've got 3.14159 and then I've got this kind of a gap and then there's kind of a dot dot dot kind of going slightly behind the numbers going towards the black hole. And then there's sort of over here, sort of some digits just on the floor. (20100305\_11)

                + -


                I'm not even sure if these are right now[digits of pi. but I am seeing 2, 7, 9, 6, 2. And then just some kind of vague numbers just kind of going off and I can't. I'm not really sure what they are because they are sort of an 8 and then another 6 2. Erm yeah, seems a bit unstable  (20100229\_71)

                - So the focus on one number. The one to right is kind of just changed. Because it's not really holding still it's kind of changing because of other numbers trying to get in from above. (20100229\_75)

                So it's 3.14 and 1 and a 5 and that's where I start getting difficulties. Next to the 5 there's like I say a 2 to the right of the 5 but there's a 9 above the 2. INTERVIEWER: How is it above the 2? AB: Just directly above on the page (20100229\_77-79).


                Erm <long pause to think>  So the focus on one number. The one to right is kind of just changed. Because it's not really holding still it's kind of changing because of other numbers trying to get in from above (20100229\_75).


                I see a 1 a 2 and a 9. I see a 7 to the right of the 2 and the 9, slightly above the 2. I just see a jumble of numbers. When I am focusing on that region now the pihas gone.  I've just brought that back by saying that <pause> pi.uals 3.1415926. Yeah and the 9 to the side of me <unclear> just kind of still there. Just kind of not really working themselves out into anything coherent. (20100229\_81).


                Yeah so as I focus on it, it becomes stable. So it's like, so it's almost like I have to fill it in with something. But they just seem to be random numbers (20100229\_83)


                So it's almost like that's kind of a new, so I was kind of looking over there towards you and there was, so I get this kind of page but then I'm kind of feel my eyes drawn back to this corridor which I'm now in. There's number 3 and then we've got pi equals 3.14159, 2 floating above this. It's because all of the digits have gone smaller  (20100229\_128).


                Erm, yes I'm kind of looking down now piis kind of over here and it's just got much smaller. So there's just kind of lots of numbers but I can't really read them. I mean I can focus on erm individual numbers I can just see a 4 there and a 9. Well now I'm seeing a sequence 4 9 3 2 8 6 but it's like I see sequences of random numbers. Erm, well I just can't relate those to the beginning of pi. (20100229\_132)


                Yeah. I think that's what's going on. So I looked to the left of those random numbers I've just said and then I saw 3.14159 again. Then I seem to not be able to get back to the same numbers as I approach it again from the left hand side. (20100229\_136).


                INTERVIEWER: So you actually see 3 dots in the corridor? AB: Yeah  (20100229\_140-141)


                So now I've got 3.14159 dot dot dot 8656 and then a whole pool a whole load of numbers just kind of like jumbled up like kind of just there not really knowing where to go. .. Er yeah kind to the side and behind the main sequence. Kind of just lying on the floor (20100229\_147-149).


                INTERVIEWER: Ok so you don't notice anything different from last week or? AB: Think the 9 has gone into place as I now know for sure that's the next digit and not the 2 that was kind of jostling for position. (20100305\_6-7)
              + -


              root2

              - So I saw a briefly flashing the square root of 2 equals 1.41 and then like there's a whole load of other numbers to the right, which are not really settling down. Just seem really random, there's a like kind of a 2 8 7 6 5 4, but that's not the correct expression of it. (20100320a\_11)


                And then 1.41. I am seeing 2 8 7 6 5 now. Not sure if those are correct numbers. (20100320a\_13)


                So I've got root 2 equals 1.4128765. I saw a 4 and then a 2 above the 4 and I see dot dot dot. Then I've got a crowd of numbers, some 6's and 5's in this crowd. And then there's kind of like a black hole to the right of that crowd. (20100320a\_31)


                Erm, yeah it's kind of like a black sludge. They are all kind of overlapping the numbers. I'm sort of aware of the presence of mainly a couple of 6's and a couple of 5's. (20100320a\_11)

                + -


                So I saw a briefly flashing the square root of 2 equals 1.41 and then like there's a whole load of other numbers to the right, which are not really settling down. Just seem really random, there's a like kind of a 2 8 7 6 5 4, but that's not the correct expression of it. (20100320a\_11).

                - The square root of 2 is kind of floating above [1 and 2 in their respective positions either side of root 2 decimal] in the middle of the room [corridor]. ... It's kind of dropped a little. It's kind of fluctuated between being up here and down there. I saw it for a moment dropped. INTERVIEWER: But it's still once in a while going up back again? AB: Seems to be more down now. ... Just appeared at the bottom as I was focusing on the number 1 and number 2. (20100320a\_47-59)

                And then 1.41. I am seeing 2 8 7 6 5 now. Not sure if those are correct numbers. (20100320a\_13).


                INTERVIEWER: What do you see after the 5? AB: It's kind of dot dot dot, and then there's kind of some rather blurry numbers to the right in a crowd. (20100320a\_14-15).


                So I've got root 2 equals 1.4128765. I saw a 4 and then a 2 above the 4 and I see dot dot dot. Then I've got a crowd of numbers, some 6's and 5's in this crowd. And then there's kind of like a black hole to the right of that crowd. (20100320a\_31).


                AB: No movement. INTERVIEWER: So the crowd is still? (20100320a\_37-38).


                AB: Erm, yeah it's kind of like a black sludge. They are all kind of overlapping the numbers. <Sneezes> I'm sort of aware of the presence of mainly a couple of 6's and a couple of 5's. (20100320a\_39)
      + -


      Attention

      - + -


        Selectively directing AB's attention to specific features of a concurrent appears to both improve the stability of the percepts along with AB's ability to describe it (as is an assumption of the EI method). However, it may not be possible to disentangle this finding\*\* from the possibility that directed attention is changing the content of the experience rather than simply increasing the subjects access to the content. causing the concurrent to visually appear and improve the stability of the percept. In either case, it appears that attention is able to selectively modulate the conscious visual content of the subjects' experience and this facet of synaesthesia should be bared into consideration during experimental design.  
          
        AB is aware of concurrents in his periphery (or those surrounding the concurrent in question) and is able to direct his attention towards them without looking directly at them (in a manor akin to normal peripheral vision). The allocation of attention through directed gaze also appears to cause the visual appearance of a concurrent, although it may also invoke inner speech and thus an 'auditory' induction.  
          
        The final position of a concurrent in the corridor appeare to be modulated by the direction of AB's gaze and attention.  
          
        Under normal circumstances, the physical room appears to hold more salience than the mental room in which the concurrents are experienced.

        - + -


          Attention focused on a percept increases its stability - more detail in description or   
                                                                                         appearance of additional features

          - + -


            AB's ability to describe specific properties of a percept (e.g. any patterning or colouration) seems to improve as he directs his attention towards it. For example, he often uses phrases such as "if I think a little bit more then I see... " (20100222\_5), "if I really try and focus..." (20100229\_398), before continuing to describe a feature which was initially 'unstable'. Note that this a primary assumption of the EI technique; subjects initially report having little knowledge of the experience or a difficulty in describing it, but through guided attention go on to produce detailed reports. In this instance, the subject does not appear to have a specific difficulty in describing the experience (compared with the difficulty one might face in describing the physical feeling associated with a specific emotion, for example), the subject appears to report difficulty in perceiving or visualising the experience itself.

            Verbalising the perceptual experience also appears to maintain AB's attention on a feature, resulting in improved stability of the percept or additional detail in the experience. For example, when AB is attempting to perform a context transition from the corridor to the white page, he initially reports that "it's hard to bring it backwards" (20100222\_66). Noting that the usual direction of the context transition is from the white page to the corridor, we might assume that this tasks requires some cognitive effort. AB then continues to describe his visual experience with "little bits of it" (ibid) coming back. As he says this the white page begins to return more fully. We may hypothesise that attempting to describe his visual experience serves to anchor his attention, making the percept more visible.\*\*  
              
            Indirect induction from the interviewer may also focus attention and/or\*\* lead to the appearance of a concurrent, for example when describing his experience of Plank's constant, AB is asked "But you didn't see any digits appear with it?" (20100320a\_215), to which he responds affirmatively and then continues with, "they are sort of coming along now." (20100320a\_216). In this example, the interviewers reference to 'digits' may have directed AB's attention to that part of the concurrent, causing digits to appear.

            Additional support for the assumption that the appearance of additional features in a percept or its description  are not the result of a hypnotic-like suggestion is evident when AB is describing his experience of pi: "When I am focusing on that region now [the decimal digits] the pi has gone.  I've just brought that back by saying that." (20100229\_81). Here AB's own reference to a feature causes it to enter his experience. A similar series of events occurs in AB's discussion of his synaesthetic time form, where he initially reports that "there are no numbers on the clock" (20100320b\_5), and then with continued attention he reports "Well I kind of get vague ones coming in along, round the outside of the clock actually. Yeah, if I can focus my attention on it." (ibid\_7). Similarly the second hand on the clock appears to "flicker" in and out of existance with each time AB brings his attention to it (20100320b\_16-22, 31-34).  
              
            There remains a possibility that this focusing of attention changes the percept itself rather than simply improving the subject ability to report or perceive the inherent detail of the concurrent. On at least one occasion AB states that, "...really staring it seems like it changes what it's made of a bit." (20100408\_333). What remains apparent however is that the conscious experience of the subject is modulated by the degree of attention afforded to the concurrent. This property of attentional modulation may be a feature of other cases and varients of synaesthesia and should be considered in future investigations.

            - ... and if I think a little bit more then I see... it hovering above a line that's sort of below me like that, sort of where I'm pointing, so looking at an angle away from me, going away from me and from left to right. (20100222\_5)


              INTERVIEWER: But you didn't see any digits appear with it? AB: No, they are sort of coming along now. (20100320a\_215-216)


              Yeah. Erm but nothing that stays really too stable. If I really try and focus I don't know, I see a little white so it's kind of mainly black top bit of it. Erm, then I am seeing a little white circles so the 5 this is. The white circle missing from top corner as though someone has punched a hole out of it from the top corner. (20100229\_398)


              ...I just see a jumble of numbers. When I am focusing on that region now the pihas gone.  I've just brought that back by saying that (20100229\_81)


              Yeah, it's hard to bring it backwards, it's hard to recreate the initial, th-the full white, background with the five. I can get little bits of it, maybe in the top left, but to totally bring back the whole number five... well now it's kind of coming back. But erm, as soon as I bring it back it kind of gets stripped away again and the five is floating above the line. (20100222\_66)

              + -


              so we talked about four and five being sort of centred in the frame... four in the lower left and five in upper right, but then... whilst I was talking about that... my attention was wondering “ well where's three and two, and six and seven” and when I started thinking about that then that caused the motion of the thing to zoom out and so then two appeared in the lower left and it went as far as erm... like every instant there's a different set of numbers in the frame, so one minute there's two, three, four, five, six, seven, they're all going in the same, the direction's staying the same. (20100215\_209).

              - INTERVIEWER: And as your attention drifted towards these extra numbers, the experience was of the frame with the numbers zooming out, and therefore making more space for the numbers... ? AB: Yeah making more space for more numbers to fit in the frame. (20100215\_212-213)
              + -


              ...There's no numbers on the clock. INTERVIEWER: No numbers? AB: No. Well I kind of get vague ones coming in along, round the outside of the clock actually. Yeah, If I can focus my attention on it. (20100320b\_5-7)

              - + -


                Second hand kind of flickers in and out of existence. I wasn't aware of that at all. ...It's kind of going up towards somewhere between 5 and 10 seconds. 9 10 11 12....That line goes around as I focus on it (20100320b\_16-20)

                - So I don't see it like every second it's just whenever my attention comes back it's kind of moved on. There's a 55 now, 60. So yeah, it's just kind of moving around. (20100320b\_22)

                  + -


                  No, I wasn't paying attention... so I was aware of those other hands, only vaguely aware, but I didn't know exactly where it was. (20100320b\_26)

                  - I wasn't paying any attention to the minute hand, but then when I drew my attention back to it, it was pointing at 3.21. (20100320b\_30)

                  INTERVIEWER: But in terms of the second [hand], you described the movement not as... not continually going around. AB: No, it's kind of like appearing, its kind of flashing  INTERVIEWER: But it's not doing it automatically, you have to look at it? AB: Yeah, pay attention to it. (20100320b\_31-34)

              I mean really staring it seems like it changes what it's made of a bit. (20100408\_333)
          + -


          Attention to peripheral concurrents

          - + -


            In most cases, AB reports that even though his attention is focused on a single concurrent, he retains an awareness of the surrounding concurrents in the appropriate sequence. He describes the experience of these surrounding as concurrents similar to 'peripheral vision': "I can see what they [peripheral numbers] are like if I focus my attention on them. So it's just in a way paying attention to what's in my visual periphery in the physical world. I can pay attention to stuff that isn't in the same direction as my gaze." (20100329\_177).   
              
            The shifting of attention towards one of these peripheral concurrents appears to increase their detail or intensity of experience or indeed their visibility. For example, "I've centred it [my perspective] on number five and so there's not really room for the number four there. ... But [now] I'm focusing my attention on it [the number four] and so it's kind of, kind of appearing." (20100215\_190-192). The shift of attention or gaze toward a peripheral concurrent may occur through auditory induction, ""I was kind of focusing on the five... when you said four, so [it]... kind of made me shift my attention, down the line" (20100215\_178), or alternatively through visual salience, "seems very hard [to be aware of the peripheral numbers] without moving my eyes. Like I'm focusing my eyes on the e. Seems like I am [making] little eye movements across to read the other numbers" (20100320a\_82).

            - I can see what they are like if I focus my attention on them. So it's just in a way paying attention to what's in my visual periphery in the physical world. I can pay attention to stuff that isn't in the same direction as my gaze. (20100329\_177)

              + -


              so if I focus my attention on E... Well I'm just aware of numbers but I can't really say what numbers. I'm only seeing the 2 and maybe the 7 but after, as soon as I focus on them then they kind of, loose my attention (20100320a\_80)

              - Erm, seems very hard without moving my eyes. Like I'm focusing my eyes on the E. Seems like I am little eye movements across to read the other numbers (20100320a\_82)
              + -


              Umm, umm, I've centred it on number five and so there's not really room for the number four there. ... But I'm focusing my attention on it [4] and so it's kind of, kind of appearing. But the number five has kind of moved off (20100215\_190-192)

              - It's kind of moved a little bit now, now erm cause we're talking more about four than five... my perspective has changed a little bit and... I've got four and five are fitting a bit more clearly into the frame now. (20100215\_200)

              I'm saying M over and over again in my head. But I've got a glimpse of an L at one point. And I've got a glimpse of N O P. It's hard to just look at the M (20100329\_249).


              I was kind of focusing on the five... when you said four, so [it]... kind of made me shift my attention, down the line, down, down and to the left down the line, and... sort of got a, getting a... flashing of the number four, appearing sort of at the bottom left of my field of vision there it's just sort of on the edge, and it's like. It's like when I'm looking at the external world and I'm trying to paying attention to something that's erm that's sort of just on the edge of my visual field. The four - you can only just see it but there's a black, you can see there's a number four there, there's a black four quite thick ink again, and again just kind of above... the number line. (20100215\_178)
          + -


          Attention induced context transition

          - + -


            The final position of a concurrent in the corridor appeares to be modulated by the direction of AB's attention (20100229\_315)

            - It seems to depend on where I focus my attention where it ends up. I am thinking more about the numbers below 60 or the numbers above 60. (20100229\_315)
          + -


          Drawn to the physical room - physical interference

          - + -


            Allocation of attention to the subject visual experience may be related the salience of its content (see for example \cite{ref}). Under this assumption, instances of physical interference may demonstrate that the physical room is inherently more salient that the mental room and attention is automatically drawn to the physical if there are competing percepts (physical and synaesthetic).

            AB confirms that he can retain an awareness of the mental room despite his attention being drawn to the physical in the following interaction: "[The percept (4) is in the mental room,] kind of superimposed [on the physical room]. Soon as my attention [is drawn to the physical room by my moving around in it, the percept] ... disappears a bit ... I'm aware of the 4 being there, but I can't really keep focused on it." (20100408\_136-137)

            - [the percept (4) is in the mental room and .:] kind of superimposed [on the physical room]. Soon as my attention [is drawn to the physical room by my moving around in it, the percept "disappears a bit"], but I'm aware of the 4 being there, but I can't really keep focused on it. (20100408\_136-137)


              Very hard to keep my attention totally focused. There's kind of a greeny, orangey colour. I don't see any details, just kind of pure kind of background. (20100320a\_100)
          + -


          Directed attention enables concurrent to be located

          - + -


            Although AB has an inherent sense of where in space a concurrent should be perceived, his direction of attention and gaze appear to 'bring about' the visual experience of the concurrent.

            *INTERVIEWER: If you let your gaze move a little bit more freely, how easy is it for the other letters to appear? AB: It's pretty easy for H I J K L M N O P Q to appear. R S T. All I have to do is move my attention along the thing. I can kind of see the whole alphabet if I want to.  INTERVIEWER: But how do you know where to look, if there's no line connecting them? AB: Because the letters are just there. INTERVIEWER: But you said they only appear when you look in their direction. AB: I just know they are there. I know where to look (20100329\_250-255)*  

            *INTERVIEWER: Do you already see a bit of the other month as well or is it just focused on a couple of days? AB: Erm, it depends on what I draw my attention to. I can draw my attention to whatever I like really. September is here, October, November, December, January, February. (20100320a\_335-336)*  

            *I have to look over to see the next letter. (20100329\_261)*

            Whilst directing his gaze towards a specific concurrent, AB is also reports engaging in inner speech on some occasions.

            *INTERVIEWER: ...how many letters are you aware of at the same time? Just depends where I focus my attention. If I focus just on the M it's quite hard [to be aware of periperal percepts]. It's almost like I have to utter the names of the other letters in my head in order to see them. But as soon as I think, you know, looking at the M and then thinking about what else I can see, need to start uttering M or L and then they come into being. (20100329\_244-245)* 

            *15 comes into place first, and then 14 and then 16, erm 17 is pretty vague. Unless I let my mind wander up the line. But if I sort of, if I... if I... centering my attention on 15 then I get fleeting images of the 14, 16, maybe 17. Even slighter, 13. ... ... I guess it depends on how long I let my attention wander as to how far up and down the line I can ‘grip'. So just, the process of talking about 13, 17, 18 popped up, err, for a moment, and as 19 just popped up and 20 there. (20100215\_101-103)*  

            - INTERVIEWER: So can you easily switch your attention between the 2 in front of you and the 2 over there? AB: Yeah. (20100415\_182-183)


              INTERVIEWER: ...how many letters are you aware of at the same time? Just depends where I focus my attention. If I focus just on the M it's quite hard [to be aware of periperal percepts]. It's almost like I have to utter the names of the other letters in my head in order to see them. But as soon as I think, you know, looking at the M and then thinking about what else I can see, need to start uttering M or L and then they come into being. (20100329\_244-245)


              INTERVIEWER: Do you already see a bit of the other month as well or is it just focused on a couple of days? AB: Erm, it depends on what I draw my attention to. I can draw my attention to whatever I like really. September is here, October, November, December, January, February. (20100320a\_335-336)


              INTERVIEWER: So, when I told you to think of the number E, the first thing you noticed was flash of the exponential graph and then the E and then it took a while for the equals sign to appear, and then the numbers appeared or did they appear the same time as the equals sign? AB: Erm, as I just kind of moved my attention from left to right I became sort of aware of them and could see them. (20100320a\_75-76)


              15 comes into place first, and then 14 and then 16, erm 17 is pretty vague. Unless I let my mind wander up the line. But if I sort of, if I... if I... centering my attention on 15 then I get fleeting images of the 14, 16, maybe 17. Even slighter, 13. ... ... I guess it depends on how long I let my attention wander as to how far up and down the line I can ‘grip'. So just, the process of talking about 13, 17, 18 popped up, err, for a moment, and as 19 just popped up and 20 there. But if I bring my attention back to 15, it won't go any further. (20100215\_101-103)


              INTERVIEWER: Well I mean, if you let your gaze move a little bit more freely, how easy is it for the other letters to appear? AB: It's pretty easy for H I J K L M N O P Q to appear. R S T. All I have to do is move my attention along the thing. I can kind of see the whole alphabet if I want to.  INTERVIEWER: But how do you know where to look, if there's no line connecting them? AB: Because the letters are just there. INTERVIEWER: But you said they only appear when you look in their direction. AB: I just know they are there. I know where to look (20100329\_250-255).


              I'm just aware of the whole presence of the whole alphabet. INTERVIEWER: But visually present is only one letter at a time? AB: Erm, usually the one before and after the one I'm looking at. I'm looking at K now, I can see the J and L next to it. Yeah, I think the one before and the one after are kind of quite clearly there. INTERVIEWER: And then it just becomes blurry? AB: Yeah I guess. I have to look over to see the next letter. (20100329\_257-261)
      + -


      Instability

      - + -


        AB often reports 'instability' in his percepts, as a flashing or brief appearance of the concurrent. This instability is reported when AB attempts to modify a feature of the concurrent from is preferred or 'default' form. For example, attempting to change the typeface of his alphabet form (20100329\_340-345), the context of the concurrent (20100329\_49) or AB's perspective of it (20100415\_97-113) all result in a experience of a percept flickering between the default and requested view/feature. Generally an unstable percept becomes fixed with focused attention and a certain amount of cognitive effort. In one case however, the form appears to automatically stabilise with a feature which opposes the direct instruction of the interviewer (20100415\_128-137).  
          
        AB also describes instability in the patterning or texture described with some concurrents such that the patterns themselves appear to move or change although the digit is itself stable. Similarly the background colours of the corridor and free space context are describe with an instability which results in a difficulty in describing them as though the exact composition of the colour is not static. These features of patterning and background colour are not common to AB's every day synaesthetic experience, therefore we may speculate that he has no 'default' experience on which the percept will stabilise.

        - + -


          Unstable induction

          - + -


            AB describes a concurrent as 'unstable' if it appears only briefly in a certain position or orientation, sometimes described as "flashing" (20100215\_188-190), "jumping" (20100215\_89) or "popping" (ibid), where the concurrent appears then disappears (we are not able to comment on the temporal scale of these appearances, but the terms used suggest rapidity). A concurrent is may be unstable if example there is interference from the physical room (20100329\_63-65) or if there are already a number of concurrents present and 'confusion' in the perceptual scene(20100415\_128-129; 20100305\_63-65).

            When AB is attempting to induce multiple concurrents for the number two he reports, "It took a while to stabilise so I sort of got repeats of the two" (20100415\_128). These repeated then suggest twenty two: "I think I'm a bit confused about whether its a two [and another] two or a twenty two" (20100415\_135). Although the instruction was to induce a single two, the concurrents have appeared to resemble twenty two. This suggestion of twenty two appears to automatically trigger the induction of the relevant surrounding concurrents, although with instability: "So I can kind of see a twenty three here and a twenty four there, but I'm also getting like a twenty one here and a twenty three above it" (20100415\_137). This instability may be due to AB's uncertainty in the directly induced number (two or twenty two). It is interesting however to note that this repeated two form tended towards twenty two rather than two, two., suggesting perhaps that twenty two would be the preferred form\*\* \cite{inJarick2011}.

            - + -


              INTERVIEWER: ...So I wonder if you try to imagine the 2... AB: It took a while to stabilise so I got sort of repeats of the 2 that had been there for a while on either side (20100415\_128-129)

              - think I am a bit confused about whether it's a 2, 2 or a 22 (20100415\_135)


                it's bit unstable. So I can kind of see a 23 here and a 24 there, but I'm also getting like a 21 here and a 23 above it. (20100415\_137)
              + -


              4000 BC... So first... struggling to find it on the number line. (20100320a\_294)

              - ...so that's 0, like 10 AD, 10 BC, 20 BC and then I was kind of here and it was kind of going off here. Then I was kind of losing touch with it and then 4000 BC just in numbers and the capital B and capital C here. (20100320a\_296)

              this is a two dimensional corner. So the line coming in... sort of from the left... It's not really stabilised, the angle of the turn isn't stabilising. (20100215\_41).


              I feel the 6 may be a bit blocked by the table now. Not sure exactly where it is. INTERVIEWER: Can you see the 5? AB: Erm, I have a sense of the 5 being somewhere over here. Can't see it very clear, well it's coming into view. There's an 8 over there. (20100329\_63-65)


              INTERVIEWER: Close your eyes please and now think about...number 5. Do you see it? AB: Takes a little while. Sense a 5 just over here. It's a little bit, it's a bit of an unstable image at the moment. INTERVIEWER: Can you try to concentrate on it? AB: Yeah. This time it's kind of gone a little bit. A bit distorted the shape. ... it's not so well formed... the top is sort of normal, so the neck going down is a bit short and then curve is kind of elongated. There's a bit of purple and red on it. It's oscillating a bit. I'm kind of getting glimpses of a better formed 5. (20100305\_180-185).


              So look back from 100. It's tricky, please stabilise...<quiet> (20100229\_343)


              INTERVIEWER: So what if I said think of a different representation once more about these fraction. I mean we could say for example 6 over 4? What do you see? AB: Quite fleeting. It's kind of appearing in the same place. And likes to go back to 3 over 2 (20100229\_54-55)

              + -


              Yeah, not really stabilising, kind of flashing a little bit. INTERVIEWER: What did the flashes look like? AB: Just the number four disappearing, it seems like there's a clash, like the number four, like there's not really room for it... I've centred it [the frame] on number five and so there's not really room for the number four there [inside the frame]. (20100215\_188-190)

              - I've still got a bit of a “shower”, still flicking backwards and forwards between the two, err, so I've got a little, glim...glimmer of the previous image of four kind of not fitting in the frame, but erm... Four's becoming a little more centred now so I had a, I had one glimmer of four and five fitting in the frame, now I'm kind of getting three, four and five fitting in the frame. (20100215\_201)


                so there's a line now... a framed line... not really stabilising. You're getting a... different number of digits appearing in it... a bit of motion there... I get different images popping up so one moment there's two three, four, five, six, seven in the frame and the next... I've got number one appearing and I've the whole number line shrinking as I zoom out, and and just seeing a straight line going from the bottom left, to the, top right. (20100215\_205)

              INTERVIEWER: Okay, so if I say, 25, do you see all the figures now? AB: Now the line appeared, so I saw 25, didn't see any other digits very clearly, hard to stabilise... but the line is going in a different direction now. (20100215\_73-74)


              [INTERVIEWER: what's the difference between the angle of the line from percept 5 to the angle of the line if I say, "count from one to three"?] I'm having trouble stabilising the digits. <animated> I keep seeing the number six the wrong, wrong side of number five. Although now it's kind of, err, so it's kind of jumping around a bit, the actual digits. Erm, number five is staying still, somehow the six is erm, popping back to where I kind of know four should be but. And with seven, the other side but then, but then it's kind of moving around a bit. (20100215\_89).
          + -


          Unstable context transition

          - + -


            A context transition may be unstable, for example with sets of concurrents existing in both contexts simultaneously (20100305\_53) or with the number line "flicker[ing] in  and out" (20100329\_49).

            - it's kind of oscillating a bit between having a line between 55 and 57 and having a little 56 in there and 56 appears as the line kind of goes away. (20100305\_53)


              INTERVIEWER: Can you make the number line appear? AB: Yeah, I can get it to kind to flicker in and out. Segments of the line joining the 5 to the 6 and 6 to the 7. (20100329\_48-49)
          + -


          Unstable feature change

          - + -


            A specific feature of a concurrent may be unstable, particularly when AB attempts to modify it. For example, when changing the case or formatting of a letter form, the requested type face is said to flash (20100329\_323) or be "mixed up" (20100329\_340-345).

            - + -


              I can get the lower case A to flash, but then doesn't stay there. (20100329\_323)

              - The lower case C is kind of being mixed up. It's kind of in bold font. (20100329\_245)

              [from normal font]  INTERVIEWER: Do you think you could get them in italics? AB: Erm, saw the C though going to italics very briefly, the capital C. ... The lower case C is kind of being mixed up. It's kind of in bold font (20100329\_340-345).
          + -


          Unstable perspective change

          - + -


            Instability in changing perspective also appears as a 'flickering' image, e.g. when AB stands up in an attempt to view a concurrent from the top it is seen to flicker between to two view points (20100415\_97-101). AB initially suggests that standing up induces a continuous change in viewing angle however the concurrent is only stable at set points e.g. when he is directly in front or above it. The achievement of a vertical (birds-eye\*\*) view may also be assisted by the interviewers instruction to focus on this perspective of the concurrent (20100415\_112-113).

            - + -


              So it's kind of hard to stand up slowly enough... INTERVIEWER: ...you're trying to stand up slowly in order to see what? AB: ...where some kind of change happens. ... it seems to flicker. (20100415\_97-101)

              - it just came into view when I sort of reached there at a certain angle. INTERVIEWER: Is it not on the table right now? AB: It's sort of flickering. (20100415\_107-109)


                INTERVIEWER: ...it stabilises [vertical]?  AB: Well that was what happened last time. Erm, because you told me to focus on the vertical 2. (20100415\_112-113)
              + -


              So I'm having trouble stabilising that [the angle of the line in relation to the corridor], not sure whether I'm thinking of 1 to 10 or 60's but erm but I've got one line kind of going more like this. (20100229\_271)

              - So, that's a bit more difficult. That's when I have to focus just on 60, rather than 60 and 61. I was having more trouble I think because I was thinking of 60 and 61 and then trying to put them down on the [line]. (20100229\_299)


                It must have fallen down at some point because it's starts off up here and then it kind of sinks more there. It's a bit unstable, it kind of yeah doesn't really, takes a while to really settle into one place. It seems to depend on where I focus my attention where it ends up. I am thinking more about the numbers below 60 or the numbers above 60. (20100229\_315)


                it's almost like... 60 has rooted now because we've been talking about it for quite a bit (20100229\_329)
          + -


          Unstable patterning

          - + -


            AB's descriptions of patterning or textures on concurrents are often described as 'unstable' in that the patterning changes, for example, "It's kind of black for a bit then it's kind of hollow, just the outline in black ink." (20100320a\_248), or "It's almost like I see for a moment some stripes around here." (20100229\_402-404). Thus although the digit itself may be stable, the patterning upon it may initially "Just seems to just be moving around." (20100229\_424). The patterning described during these interviews is not a regular feature of AB's synaesthetic forms, thus we might suggest he is 'unfamiliar' with these features and does not have a 'default' form.

            - Not very stable. The five itself isn't very stable, and in terms of err, colour, and shading of it... it seems like there's always kind of a... a black, sort of black outline of the number five ... Yeah it's not staying... stable black (20100222\_14-16).

              + -


              It's quite hard to draw. It's not as fat as that. ... <pauses to think> all kinds of marking, but they are kind of not really stable. It's almost like I see for a moment some stripes around here. (20100229\_402-404)

              - So it's black at the top and there's kind of white bits <pauses> Not really stabilising very well, I mean sometimes I see bits of like stripes and it's hard to draw precisely. (20100229\_412).


                So that's kind of black up to there. And then curvy bit and a few stripes around here. And the bottom is just white. There it's a bit unclear. (20100229\_420)


                Have to really kind of try and focus on one bit of the number or another (20100229\_422)


                Just seems to just be moving around. ... Not the digit itself but the markings on it. (20100229\_424-426)


                INTERVIEWER: Is it shifting around or is it becoming more stable? AB: Becoming more stable. Yeah, the black splodge is a bit of a mess really. (20100229\_433-434)

              It's kind of looking more black now. It's not really stabilising that easily. It's kind of black for a bit then it's kind of hollow, just for the outline in black in. That kind of orangey, greeny background, you can see through the outline in ink. (20100320a\_248)


              I can focus on the individual lines of the B. I mean if I really focus on it. Now it's like it's made of ribbons attached to each other.  ... They're not very stable.  (20100408\_329-331)
          + -


          Unstable background colours

          - + -


            The background colours of the corridor and free space are consistently described as unstable, with descriptions such as "grereny, greeny-brown, orange" for the corridor (20100222\_27). AB asserts that the instability here is not in the vagueness of the visual experience but more in the difficulty of describing the colours (20100305\_59-65), although there is a suggestion that the colours themselves may change, e.g. "sometimes it's purpleish, sometimes it's greenish" for free space (20100329\_13).

            - + -


              corridor

              - There's kind of a greeny, green-brown, orange, sort of somewhere in there, it's not... it's not that stable. (20100222\_27).


                There's this big black mass there. Just concentrating on the black then it kind of flickers for a bit and the corridor just kind of comes back for a second (20100229\_124).
              + -


              backgrounds

              - I didn't really get one stable colour ... It is one of the ones that was kind of in that mix. ... INTERVIEWER: Ok, so the difficulty is in describing the colours, not that the colour itself is somehow undefined or unstable. AB: No, the colour seems pretty stable. (20100305\_59-65)


                The [free space] background is a bit unstable, sometimes it's purplish and sometimes it's greenish (20100329\_13)
  + -


  Inducer-concurrent relationships

  - + -


    During the interviews most inducers were presented audibly, e.g. in a question form the interviewer, "If I say, five...What do you see?" (20100215\_79-81). AB also appears to 'auto-induce' a concurrent through his own inner speech (e.g. reciting the name of the concurrent in his head) and we find that where AB is directing his attention to 'where a concurrent should be', he is also employing inner speech, e.g. "right I'm going to look for the 4 now!" (20100408\_138). We not however that this spatial redirection of attention via inner speech may not happen on the time scale of normal conscious inner speech, rather it is likely experienced as instantaneous thoughts, which become realised as inner speech through the EI process.

    Concurrents may also be automatically generated if they are related to the induced item by memory associations, such as a series of related mathematical equations (20100320a\_195-224) . We also find that the concurrent of a single induced item may be repeated or have multiple representations, e.g. when AB attempts to view a concurrent from a certain perspective, a new concurrent is generated in the required view and the default concurrent remains. Similarly AB may create multiple representations of the same fraction but in different notations (e.g. decimal or common fraction). Here the multiple representations of the same concurrent are experienced as 'superimposed' on top of on another (20100229\_24-25). We also find that once an item is induced the concurrents continue to exist in AB's experience even if his attention is directed elsewhere for an extended amount of time (20100229\_384).

    - + -


       External and internal dialogue

      - + -


        Auditory presentation appears to be the most effective induction method for AB's synaesthetic percepts (note that visual presentation is discussed in 'Physical interference'')\*\*. We find that the directly induced item is accompanied by those surrounding it in sequence and repeated mention of an inducer or appears to increase its stability (20100408\_41). We speculate that when AB reports to be 'thinking about' a concurrent, he may often be employing in inner speech and that this process of induction may be similar in mechanism to auditory induction from an external source. On other occasions he reports to engaged in visualisation of a concurrent to induce it (e.g. 20100222\_261-263). We also find that reference to a memory of a concurrent my induce it, however with a degree of vaugeness and uncertainty which may distinguish it from other 'unstable' concurrents (20100320a\_2-9).

        - + -


          Auditory induction

          - + -


            For the majority if the interviews synaesthetic inducers were presented to AB audibly. That is, the interviewer would ask "If I say, five...What do you see?" (20100215\_79-81). Direct induction of a single concurrent is also accompanied by the concurrents surrounding the induced item, for example when the interviewer presented the verbal inducer fifteen, AB reports that he is "actually seeing more digits now. I sort of see 14 and 16 and 17" (20100215\_61) (see also 20100329\_249, 20100329\_258-259, 20100408\_37).  Repeated mention of an inducer or appears to increase its stability, for example AB reports that once a letter concurrent has stabilised, he does not have to continue to say the letter (20100408\_41). Similarly the interviewer's mention of an inducer serves to stabilise it, for example a two concurrent stabilses in a non-defualt perspective "because you  [the interviewer] told me to focus on the vertical two" (20100415\_112-113). AB's percepts appear to be highly compliant with the interviewer's instructions, for example AB becomes aware of a five concurrent and directly attributes it's appearance to the interviewer's suggestion: "I'm being aware of it now you said, "is there a 5 there?"" (20100408\_95).

            - It seems to get triggered by you saying it (20100222\_62)


              INTERVIEWER: So when I say five, do you immediately see the five? AB: It seems to take a little while. (20100222\_71-72)


              neither was it immediate when you just say numbers and letters to me (20100408\_61)


              You say 15, I'm actually seeing more digits now. I sort of see 14 and 16 and 17 (20100215\_61).

              + -


              INTERVIEWER: So if you look at the M, how many letters are you aware of at the same time? (20100329\_244) AB: so I'm saying M over and over again in my head. But I've got a glimpse of an L at one point. And I've got a glimpse of N O P. It's hard to just look at the M (20100329\_249).

              - INTERVIEWER: But visually present is only one letter at a time? AB: Erm, usually the one before and after the one I'm looking at. I'm looking at K now, I can see the J and L next to it. Yeah, I think the one before and the one after are kind of quite clearly there. (20100329\_258-259).


                Erm, after internally kind of say the letter that I am looking at that I mean I can have, I am going to be aware of the sequence with several letters in between A and H. (20100408\_37)

              It's just kind of a haze. I kind of got a Z appearing up and to the left of the A for a moment there. Smaller Z. <laughter> Only after a bit you said "Is there anything there?" (20100329\_297).


              I'm being aware of it now you said, "is there a 5 there?" (20100408\_95)


              INTERVIEWER: ...it stabilises?  AB: ...because you told me to focus on the vertical 2. (20100415\_112-113).


              INTERVIEWER: What about if you transfer it to the other end of the alphabet Z? AB: Just have to relook where it is. INTERVIEWER: It's just a movement of the eyes? AB: Yeah. INTERVIEWER: You don't have to say anything. AB: No, I think you saying it is enough (20100408\_42-46).


              INTERVIEWER: Ok so last time you said that in order to keep it there you had to internally always repeat the letter. Are you doing something like that this time? AB: Erm, after internally kind of say the letter that I am looking at that I mean I can have, I am going to be aware of the sequence with several letters in between A and H (20100408\_36-37). I think once it's there I don't have to say H (20100408\_41).


              INTERVIEWER: I'm going to say M. What do you see? AB: Yeah, I sort of see M here and then I sort of see an L to the left and then an N. I see a J K L M and then a P there. (20100329\_212-213).


              INTERVIEWER: So what if you pick a random one, just choose it yourself, how would you go to it?  AB: Yes, then I would have to say it in my head. So I think Q was kind of here. ... I kind of just thought of the letter Q by saying it in my head, then I kind of looked for it (20100408\_48-51).
          + -


          'Thinking' and inner speech

          - + -


            It is not within the scope of this project to determine the processes or acts involved during 'thinking' per se, but one such act may likely involve inner-speech and as such lead to similar results as auditory induction. For example, AB is asked to chose an alphabet inducer at random and then describe his synaesthetic experience. It is found that AB thought of the letter and said it in his head simultaneously and then looked for where it would be in relation to other stable alphabet concurrents in his experience at the time (20100408\_48-51). Similarly when AB is asked to focus on a single concurrent M, he resorts to "saying M over and over again in my head" (20100329\_249).

            - INTERVIEWER: Why are there two now? AB: Just because thinking of 60.<laughter> (20100229\_354-355).


              I just faced this direction and then thought of 55 (20100305\_142)


              INTERVIEWER: Close your eyes please and now think about favourite number, number 5. Do you see it? AB: Takes a little while. Sense a 5 just over here. It's a little bit, it's a bit of an unstable image at the moment. (20100305\_180-181).


              INTERVIEWER: So what if you pick a random one, just choose it yourself, how would you go to it?  AB: Yes, then I would have to say it in my head. So I think Q was kind of here. ... I kind of just thought of the letter Q by saying it in my head, then I kind of looked for it. (20100408\_48-51).
          + -


          Imagining and visualisation

          - + -


            AB also appears to be able to induce a concurrent at will by imagining or visualising it (20100415\_122-123). AB reports that this new concurrent "took a while to stabilise" (20100415\_129). We may speculate that during this time AB was employing inner-speech and attentional re-direction processes to stabilise the concurrent. On other occasions the induction appears more rapid: "I was just envisioning another number five, that was kind of here... just kind of appeared." (20100222\_261-263).

            - + -


              INTERVIEWER: what if I ask you to say imagine or visualise a 2 next to it. How would that look different to the 2 that's already there? AB: Erm, just kind of like a duplicated, number 22 right now (20100415\_122-123).

              - + -


                It took a while to stabilise so I got sort of repeats of the 2 that had been there for a while on either side and then in the end the new one was sort of thinner and blacker. (20100415\_129).

                - so the instruction to imagine a 2 is not quite as strong, took longer to stabilise... specualtive.

              Erm, I think I was just envisioning another number five, that was kind of here. And it had a kind of dark grey colour to it, just like kind of metallic. INTERVIEWER: And how was that five related to the other five? AB: So... just kind of appeared, it wasn't in the same room.  (20100222\_261-263)
          + -


          Memories of concurrents

          - + -


            As distinct from visual imagery or inner-speech, AB is able to discern a visual memory of a concurrent. At the opening of the session 20100320a the interviewer enquires as to wether there are any concurrents present in the room (addressing a feature raised previously that a concurrent may continue to 'exist' when AB's attention is not directed towards it). AB reports, "maybe I can remember there was something here. ... Hard to tell if it's just a memory or if it's really there... Can't remember what number it was whether it was a 55 or a 56. Yes, so there's obviously nothing there that's sort of firm there. Kind of got a bit of an image of a big 55 there then a big 56 sort of stuck on the wall there. Nothing really that stable." (20100320a\_2-9). Here we see that the simple memory and discussion of a concurrent serves to induce an unstable percept, but the vagueness of the description may differentiate it from more classically\*\* unstable percept which tends to 'flicker'.

            - INTERVIEWER: Ok to begin this session... Did you notice any numbers when you came into this room? AB: No, erm maybe I can remember there was something here. ... Hard to tell if it's just a memory or if it's really there. INTERVIEWER: Well have a look at the wall. AB: I remember something being over there as well. Can't remember what number it was whether it was a 55 or a 56. Yes, so there's obviously nothing there that's sort of firm there. Kind of got a bit of an image of a big 55 there then a big 56 sort of stuck on the wall there. Nothing really that stable. (20100320a\_2-9)
      + -


      Directional attention between concurrents

      - + -


        Directional attention plays a role in AB's ability to perceive a concurrent, for example AB reports that if he allows his gaze to move freely along the form of the number or alphabet line he can see as many or as few concurrents as he chooses (20100329\_251). Directing gaze or attention may of itself involve inner speech, for example AB reports in one instance that during this act of 'looking for a concurrent' he says to himself: "right I'm going to look for the 4 now!" (20100408\_138). Thus it may not be the act of placing attention in a specific location which causes the concurrent to appear (suggesting that the spatial location is itself the inducer), rather the act of changing the focus of direction involves auditory induction through inner speech.

        AB also has a strong prior knowledge of where each concurrent should be located in relation to another in the same form, such that in directing his attention he often reports that he knows where to look (e.g. 20100329\_255). This act may again involve inner speech auditory induction, for example AB reports in a generalisation "OK, it's a 3 so down to the left of 3 there should be a 1 and 2.” And then, well there they are." (20100408\_93).

        - + -


          Attention and eye movements

          - + -


            As is expected with a spatial-form synaesthesia, AB's experience of a concurrent is related to his direction of gaze or spatial focus of attention. There are slight inconsistencies in AB's description, where on some occasions he suggests that the number appears "where I focus my attention" (20100329\_245, see also 20100408\_42-47 and 20100408\_75-77), that is, the spatial location is the inducer and the number is the concurrent. AB reports that if he allows his gaze to move freely along the form of the number or alphabet line he can see as many or as few concurrents as he chooses (20100329\_251). Similarly in locating a concurrent on is age-form, AB reports that he is initially "more aware of the position" (20100320b\_100) of this point, relative to the other months, rather than the age/number itself. However as he soon as he focuses on this position he is able to induce the digits "one, four" in the correct position (20100320b\_100).

            However, it is also suggested that the act of re-directing spatial attention itself entails a conscious decision to locate a concurrent and involves inner speech in the utterance\*\* of the number name, suggesting that the inducer is the number and the concurrent is both the number AND a spatial location. For example, "INTERVIEWER: So what do you do in order to impose it [the concurrent 4] onto the physical room? AB: Just say, "right I'm going to look for the 4 now!" INTERVIEWER: Do you actually have an internal dialogue going on when you tried that?  AB: Erm, a voice. There's like a decision. INTERVIEWER: Like an intention? AB: Yeah, an intention sort of before." (20100408\_138-143).

            - INTERVIEWER: So if you look at the M, how many letters are you aware of at the same time?  AB: Just depends where I focus my attention. If I focus just on the M it's quite hard. It's almost like I have to utter the names of the other letters in my head in order to see them. But as soon as I think, you know, looking at the M and then thinking about what else I can see, need to start uttering M or L and then they come into being. (20100329\_244-245).


              INTERVIEWER: if you let your gaze move a little bit more freely, how easy is it for the other letters to appear? AB: It's pretty easy for H I J K L M N O P Q to appear. R S T. All I have to do is move my attention along the thing. I can kind of see the whole alphabet if I want to (20100329\_250-251).


              I was kind of immediately aware of the H, just kind of where I put my gaze. Away from the distracting pi.e of paper (20100408\_13)


              INTERVIEWER: What about... the other end of the alphabet, Z? AB: Just have to relook where it is. INTERVIEWER: It's just a movement of the eyes? AB: Yeah. INTERVIEWER: You don't have to say anything. AB: No, I think you saying it is enough (20100408\_42-47).


              I have to will an alphabet on the page in order to get it to happen ... I just look around the H for sign of other letters. (20100408\_75-77).


              INTERVIEWER: So what do you do in order to impose it onto the physical room? AB: Just say, "right I'm going to look for the 4 now!" INTERVIEWER: Do you actually have an internal dialogue going on when you tried that?  AB: Erm, a voice. There's like a decision. INTERVIEWER: Like an intention? AB: Yeah, an intention sort of before. (20100408\_138-143)

              INTERVIEWER: What if it's 1 year and 2 months, what happens then? (200100320b\_95) AB: Saw it as kind of... What did come into view? I was more aware of position [relative to the markers for 12months, 18months] than of particular number. Well I can do a 14 there now. 1 4 (20100320b\_100).
          + -


          Knowledge

          - + -


            AB appears to have a strong knowledge of where in space one concurrent should appear in relationship to another. This enables him to locate a concurrent in space without an external inducer:  "INTERVIEWER: But how do you know where to look? AB: Because the letters are just there. INTERVIEWER: But you said they only appear when you look in their direction. AB: I just know they are there. I know where to look." (20100329\_252-255).  This process involves spatial direction of attention and it also appears that in some cases this directing of attention may involve inner speech and hence direct auditory induction:  "Erm, normally it's like I said to myself, “OK, it's a 3 so down to the left of 3 there should be a 1 and 2.” And then, well there they are." (20100408\_93).

            - INTERVIEWER: But how do you know where to look? AB: Because the letters are just there. INTERVIEWER: But you said they only appear when you look in their direction. AB: I just know they are there. I know where to look. (20100329\_252-255).


              Erm, normally it's like I said to myself, “OK, it's a 3 so down to the left of 3 there should be a 1 and 2.” And then, well there they are (20100408\_93).
      + -


      Inducer associations lead to involuntary inductions

      - + -


        On some occasions, currents are also perceived if they are related to the induced concurrent through normal associative mechanisms, for example the induction of the physical constant 'c' also caused the rapid and automatic experience of five other physical constants (20100320a\_195-224). Similarly, when AB is asked to transform a letter into an italic font, instead an associated bold font is perceived.

        - + -


          associations (other than surrounding letters/numbers)

          - + -


            On a number of occasions AB experiences a concurrent which is neither directly induced nor is it immediately adjacent in sequence to the induced item. In these situations, the concurrents experienced appear to be related to the inducers through normal associative mechanisms.  In the explorations of physical constants the interviewer inadvertently suggests "physical constant[s]" as an area to be explored, to which AB responds with the exemplar "the speed of light" (20100320a\_195-196). AB describes the speed of light concurrent but also reports the automatic experience of concurrents for five other physical constants and their varying expressions (20100320a\_195-224). The diachronic examination of this experience suggests that they may have been present in AB's experience prior to the concurrent for the speed of light. Thus it appears that the broad category "physical constant" lead to the rapid generation of numerous representations of associated items before the selectively induced item. On another occasion, the interviewer requests that AB attempt to modify an alphabet concurrent: "INTERVIEWER: Do you think you could get them in italics?" (20100329\_340). while the requested form is stabilising, the concurrent adopts a bold type face rather than italic (20100329\_345). This bold varient is not explicitly mentioned, however we may speculate that it appears due to it's associations with the requested italics.

            - + -


              INTERVIEWER: What about a physical constant? AB: Like the speed of light (20100320a\_195-196).

              - + -


                Yeah, what else came into view was Planck's constant, like the H bar. I just saw like a H bar and then over here there's an equals and a C square came up (20100320a\_202).

                - + -


                  INTERVIEWER: Did it happen before you saw the equation C equals 3 times 10 to the 8? AB: Yeah, it happened before the equation (20100320a\_203-2-4).

                  - physical constant (Planck's) came first as inducer 1, then c equation as inducer 2
                  + -


                  INTERVIEWER: Do you see any equations or graphs? AB: Yeah, I got E equals H bar times F.... And just underneath I've got H bar times a Greek nu as well (20100320a\_219-222).

                  - Well those are the same thing, the F and nu, the same equation and a different notation. I've got H bar equals H over 2 pi. Can't even remember if these are correct or not (20100320a\_224)

                INTERVIEWER: So how come the Plank's constant appeared? ... Did you think of the Plank's constant as well? AB: Yeah, I guess because it's another fundamental constant of the universe. It's like C (20100320a\_211-214).

              INTERVIEWER: Do you think you could get them in italics? AB: Erm, saw the C though going to italics very briefly, the capital C. ... The lower case C is kind of being mixed up. It's kind of in bold font. <laughter> (20100329\_340-345).
      + -


      One inducer may lead to multiple concurrents

      - + -


        One inducer may be perceived as multiple representations of the same concurrents, that is a letter or number may be directly induced but then the same concurrent may simultaneously exist elsewhere in AB's experience, particularly if AB is directed to perform some act upon that concurrent. For example, when AB is asked to view a 'four' from a different angle, he reports experiencing two 'fours', one in the normal view and one back-to-front (20100408\_125-129). Alternatively in some context transitions AB reports experiencing simultaneously a 'big' concurrent in the free space context and a 'smaller' one in the correct position on the line. In both of these situations the new representation of a concurrent occupi. a different physical location in the mental room than the original. However, AB also reports to experience multiple representations in different forms of fractions (e.g. 1.5 and 1/2), in which case these tend to occupy the same space with the new representation being 'superimposed'  on the old.

        - + -


          multiple representations

          - + -


            Under certain circumstances BA may experience multiple representations of the same concurrent following a single inducer. This multiple representations are experienced for example when the inducer can be expressed in different notations (such as in fractions or equations), when AB is asked to view the concurrent from a different perspective or with a different feature such as a type face, induce a context transition or when the concurrent percept is unstable due to physical interference for example. In some cases the patterning and form of the multiple representations are equivalent, however in other cases they have distinct properties and appearances, such as different physical locations in the mental room. In all cases however, it appears that the the original (induced) concurrent remains fixed and stable in his experience.  
              
            Where these multiple representations are of equivalent meaning (for example 1.5 and 1/2) the concurrents appear to occupy the same space or be roughly "superimposed" on one another (e.g. 20100229\_25). When AB performs a voluntary context transition from the white page to the corridor, he appears to experience both a larger concurrent in the white page/free space and another  smaller representation in the line/corridor (e.g. 20100329\_119-163). Unstable percepts or those which AB describes as a bit 'confused' sometimes appear to stabilise into two seperate representations (e.g. 20100415\_135-138). Where physical interference is reported, a new representation of the same concurrent may be spontaneously generated in a different area of space.

            - + -


               differnt patterns/properties

              - + -


                Multiple representations of the same concurrent do not necessarily have the same physical features such as size or patterning. "INTERVIEWER: ...a different one? AB: Err, it was much err thinner, so one in front of me, that was like had a sort of er width to it, like a depth, ahm, but this one's kind of much flatter" (20100222\_270-271).

                - INTERVIEWER: ...a different one? AB: Err, it was much err thinner, so one in front of me, that was like had a sort of er width to it, like a depth, ahm, but this one's kind of much flatter (20100222\_270-271).


                  So there's a number line going this way, through the big one there, and this one... if I look over there there's a little six, so there's like two number lines now. But erm... I can only see one at a time  (20100222\_275).


                  It's in the corridor now. INTERVIEWER: The same corridor, or a different corridor? AB: It's... all in the same corridor (20100222\_277-279)

                  + -


                  I did get a flickering 1 3 here linking up with the second [visual imagery exercise] 2. ,,, INTERVIEWER: ...If you look at both of them [the original and the visual imagery exercise 2] now, do they have the same texture? AB: No, so the one on the left [original] is kind of more shiny and sort of metallic, the other one is just kind of black. Kind of soft texture. ... the newer smaller one is black. (20100415\_141-157)

                  - INTERVIEWER: What if we try to get another 2 where you try to get the metallic one which was the first one right? Try to imagine the metallic one in a different location. AB: Yeah, I got one over there on the far desk. INTERVIEWER: Right. Ok, and what does this one look like? AB: It's pretty similar [to the original 2], with a silver outline, kind of black in the middle. (20100415\_166-169)

                    + -


                    INTERVIEWER: One more try [at visualising and generating another 2], what about in that direction? AB: Yeah, it's kind of more similar [to the original] now. It's got the silver metally thing and the sort of outline and sort of purpley thing on the inside. INTERVIEWER: So it's not as black anymore? AB: No. INTERVIEWER: Is it getting more shiny? AB: Yeah, so I can see a bit of side of the 2 and it's kind of shiny. INTERVIEWER: Is it the same kind of metallic shininess than the 2 in front of you? AB: Yeah. ... Both [this and the original] kind of sitting on the tables. So this one is directly facing me and that one is kind of facing that way. So I'm kind of slightly sideways on. (20100415\_174-185)

                    - INTERVIEWER: So do you think you can imagine it [the newer one] would vary, like the purple bit in the middle bit to disappear or be replaced by the metallic outer rim? AB: Erm, it's harder than with the elephant [exercise]. You get a bit of green at the bottom. Hard to get it fully metallic. ... INTERVIEWER:So when you say it's harder, what does that mean? Does that mean when you try something unexpected happens or nothing happens? AB: Nothing happened for a while and then I got a bit of green at the bottom. INTERVIEWER: So even though you said ok I want to imagine it, I want to change it to be metallic in the middle, it didn't happen for a while. AB: Kind of metallic one appearing briefly over there in the middle of the space, and that one won't really change. (20100415\_188-196)

                    INTERVIEWER: ...So the fact of the matter is, it was easier to create a new 2 then to change the one that was already there in the left corner. But it's still not quite the same right? AB: No. (20100415\_211-212)
              + -


              Different concurrents for equivlent inducers, e.g. fractions

              - + -


                There are occasions where AB is presented an inducer which could be expressed in multiple notations, e.g. 0.5 could also be expressed as 1/2. With these inducers, it appears that AB initially experiences both/multiple expressions occupying the same space, however with continued attention they may stabilise onto seperate locations.  "INTERVIEWER: ... So if we take between say 1 and 2, what do you see? AB: I see 1, 1.5 and 2 (20100229\_8-9). INTERVIEWER: Can you change the representation of the fraction? AB: Yeah I can see there is 3 over 2 (20100229\_22-23). INTERVIEWER: Can you see any difference between 3 over 2 or 1.5? AB: See them as super imposed, one on top of the other (20100229\_24-25). ...I can kind of see just like a juxtaposition which one is on top. I can make the 1.5 fade away or I can make the 3 over 2 fade away  (20100229\_27) ...just as if you've written them on the same bit of paper, one on top of the other (20100229\_29). INTERVIEWER: So what if I said think of a different representation once more about these fractions. I mean we could say for example 6 over 4? What do you see? AB: <pause> Quite fleeting. It's kind of appearing in the same place. And likes to go back to 3 over 2 (20100229\_54-55). So now I've got 6 over 4 as being slightly up and to the right of the 3 over 2 .... It's still overlapping slightly. ... so the 6 is kind of unimpeded but then the 4 is getting in the way of the 3 (20100229\_59-63). INTERVIEWER: Ok. But if you like there was a tendency towards the 3 over 2. Could you describe that? AB: But now I've got both, they're not in the same place" (20100229\_64-65).  This is similar to the pattern of experiences when AB is describing different expressions in mathematical formulae. Although different notations of the same mathematical formula may have memory associations with a single inducer, here we see that alternate notations initially occupy the same or a close spatial location as each other whereas normal memory associated items automatically occupi. different locations  (20100320a\_220-224). For example, "I got E equals H bar times F. ... And just underneath I've got H bar times a Greek nu as well. ... Well those are the same thing, the F and nu, the same equation and a different notation." (20100320a\_220-224).  One final example can be drawn from AB's extended description of a visual scene representing the birth of a child whilst exploring AB's age form. Here AB describes a highly detailed (static) auditory and visual hospital scene in which a child is being born; it appears as if the scene represents 0 years old (the moment of birth), and both the scene and the number 0 occupy the same physical space, presumably because they bothe represent the same thing.  "INTERVIEWER: ... So we had this whole scene with the bed and the drawers and work surface, where was the zero in all of this? AB: Kind of superimposed by that scene at the hospital." (20100320b\_177-178).

                - INTERVIEWER: ... So we had this whole scene with the bed and the drawers and work surface. Where was the zero in all of this? AB: kind of superimposed by that scene at the hospital. (20100320b\_177-178).

                  + -


                  fractions

                  - INTERVIEWER: Can you see any difference between 3 over 2 or 1.5? AB: See them as super imposed, one on top of the other (20100229\_24-25).


                    I can kind of see just like a juxtaposition which one is on top. I can make the 1.5 fade away or I can make the 3 over 2 fade away  (20100229\_27)


                    That just as if you've written them on the same bit of paper, one on top of the other (20100229\_29)


                    No, they are easy to recognise (20100229\_31)

                    + -


                    So the 3 over 2 occupi. more vertical space. So all of the numbers I think are the same size, like all the actual digits. So you got the 3 over 2 and then you got the 1.5 and that (20100229\_33)

                    - Sticking out. ... they're not totally covering each other up (20100229\_35-37)

                    Yeah they're just flat on each other. I'm just seeing them on a page basically (20100229\_45)


                    So now I've got 6 over 4 as being slightly up and to the right of the 3 over 2 (20100229\_59)


                    But now I've got both, they're not in the same place (20100229\_65)


                    so sort of 0.3 slightly to the left of the 1 over 3 and then there's a, then they get smaller and end up very close to the 1 of the 1 over 3 and then they are going off sort of diagonally to the right and behind of the 1, into the distance (20100305\_173).
                  + -


                  graphs

                  - Seems to be occupying different spaces (20100320a\_158).


                    I got E equals H bar times F. INTERVIEWER: It's also sitting on top? AB: Yeah. And just underneath I've got H bar times a Greek nu as well. ... Well those are the same thing, the F and nu, the same equation and a different notation. I've got H bar equals H over 2 pi(20100320a\_220-224)

                  I have all kinds of clocks (20100320b\_44)

                  + -


                  different forms for same inducer

                  - + -


                    When investigating the properties of larger numbers (in the thousands) there is a moment of confusion where AB is instructed to consider the number "nineteen ninety five". This inducer leads to a concurrent from to AB's year spatial form, rather than his number form. When investigating this further, it appears that AB can experience simultaneously the concurrent for the year for 'nineteen ninety five' and the number form 'one thousand nine hundred and ninety five'. The inducers for both forms represent the same digits, however the sematic associations of the different different manners of expressing the numbers appear to cause them to induce different concurrents. The concurrents themselves also differ in a manner which slightly reduces the ambiguity of the inducer through the inclusion of a thousand delimiting comma: "INTERVIEWER: So if I tell you what about one thousand, nine hundred, ninety five? AB: Up here. One, comma, nine nine five. INTERVIEWER: Ok what if I tell you now that say Francisco Varela published a nice paper in nineteen ninety five? AB: Yeah, that's over here; One nine nine five. INTERVIEWER: Do you have both of them in view, the one comma and? AB: Yes, there's one here and one there. There's one comma nine nine five and it's kind of thinner letters, thinner digits. And this one's got thicker digits one nine nine five, no comma." (20100320a\_286-291).

                    - INTERVIEWER: So if I tell you what about one thousand, nine hundred, ninety five? AB: Up here. 1 comma 9 9 5. INTERVIEWER: Ok what if I tell you now that say Francisco Varela published a nice paper in nineteen fifty five? AB: Yeah, that's over here; 1 9 9 5 INTERVIEWER: Do you have both of them in view, the one comma and? AB: Yes, there's one here and one there. There's 1 comma 9 9 5 and it's kind of thinner letters, thinner digits. And this one's got thicker digits 1 9 9 5, no comma (20100320a\_286-291).
              + -


              Multiple representationsfrom different perspectives

              - + -


                AB is able to direct his attention to different areas of a single concurrent to view them from different angles or perspectives. It generally appears that when AB attempts to look from a different angle a new concurrent is generated with the required perspective whilst the original concurrent remains fixed.

                In the exerts below, AB first attempts to view a four from the back by physically moving around behind it in the room. In the second exert he attempts to view a two from above by standing up and looking down on it.

                "...there's a 4 and a 6 kind of here. ... INTERVIEWER: If you moved your head from side to side like this, how does your perception of the number change? AB: Erm, sometimes it tilts a little bit so it's kind of bi-stable. ... I can see the back of the 4 [if i move round it and look at the back]. Yeah I can kind of see the 4 backwards. Sometimes it sort of spins around.  ... So it's always kind of bi-stable, but yeah there's kind of one where it's kind of back to front."(20100408\_99-107). ... it's like there's two here. So there's one where the 4 is just like that and then is another one where it's kind of [back to front]. INTERVIEWER: I've noticed that at the moment you are actually standing still. Can you see both of the different ones even though you are not moving? AB: Yeah, there's kind of one like this and there's one kind of like that. INTERVIEWER: So you have two different forms there? AB: Yeah." (20100408\_125-129).

                "So I've got a 2 here. ...  INTERVIEWER: ...So how does it change when you stand up for example? AB: ... looks like I am kind of above it ... INTERVIEWER: ...if you go exactly above it. AB: Kind of disappeared now. ... Seems to be pretty much vertical. I mean if anything, I just sort of see a thick line there where 2 should be. ... I am also seeing a 2 like that as well simultaneously. INTERVIEWER: Oh, like the same spot, as if it was lying on the table? AB: Yeah. INTERVIEWER: So when did that one appear? AB: Just now. And I was trying to work out what I was seeing. It gets a bit fuzzy when I'm standing directly above it. ... It's sort of different images coming into my mind. One is just of erm the top of the 2. Another is it with it slightly angled. Then another is the 2 just flat on the table. INTERVIEWER: Right. So at least three different kinds of fitting here. AB: Yeah. That's just sort of less stable. INTERVIEWER: If you sit down, what happens with them? AB: So, I've got a 2 flat on the table now. Then I've got the other one is kind of there standing up and flicking between the two. ... INTERVIEWER: Ok, shall we try it again? If you just focus on the one that's standing up and then just rise up while looking at it. What happens to it? AB: ...it seemed to jump for a moment like flat on the table. And then sort of comes back and is standing up. So it's kind of hard to stand up slowly enough ... it seems to flicker. I'm getting images of a 2 going just flat. ... INTERVIEWER: But was the one flat on the table present the whole time... when you were standing up and down? AB: No, it just came into view when I sort of reached there at a certain angle. INTERVIEWER: Is it not on the table right now[whilst you're sitting down] ? AB: It's sort of flickering. INTERVIEWER: And then when you start moving at some point it ... stabilises? AB: Well that was what happened last time. Erm, because you told me to focus on the vertical 2. INTERVIEWER: ...When you were ...focusing on the vertical [2], did it at any time disappear and then the other one [appeared]? AB: Yeah, when I got to a certain height. INTERVIEWER: And then when you were at the top basically stood up, the standing up one came back. AB: Yeah, yeah and then continued to alternate with the [flat one]." (20100415\_65-119).

                In this next example, AB is asked to "zoom out to view the whole alphabet". Doing so appears to cause the generation of two alphabet lines/forms; one distant and smaller and one closer and larger.

                "INTERVIEWER: Can you zoom out and have a look at the whole alphabet? AB: <pause> Erm yeah ... just kind of appears as that chart I drew. ... INTERVIEWER: What do you see when you look at the letter A? AB: Just the letter A. B down and the right of it. ... INTERVIEWER: So the zoomed out version of the alphabet disappeared when you switched your attention to the A? AB: ...it's kind of in the distance. It's probably further away from me than I can see. ... Big A up there. ... INTERVIEWER: Is that [big] A connected to the alphabet? ... AB: Yeah, it's kind of in the position of where the A is in that alphabet that is far way. ... this [big, closer] alphabet is kind of here A B C.... INTERVIEWER: So the zoomed out one has moved into the distance and now you have a new one that's appeared with the bigger letters." (20100329\_160-190)

                - I was looking at them from the front. But they kind of turned around for me. (20100408\_415).


                  INTERVIEWER: So in some sense switching perspective is necessary in order to look at one or the other. You don't have both of them in view at the same time.? AB: No.  (20100329\_112-113).


                  I'm just aware of the whole presence of the whole alphabet. INTERVIEWER: But visually present is only one letter at a time? AB: Erm, usually the one before and after the one I'm looking at. I'm looking at K now, I can see the J and L next to it. Yeah, I think the one before and the one after are kind of quite clearly there. ... INTERVIEWER: Can you zoom out and have a look at the whole alphabet? AB: <pause> Erm yeah ... just kind of appears as that chart I drew. ... INTERVIEWER: What do you see when you look at the letter A? AB: Just the letter A. B down and the right of it. ... INTERVIEWER: So the zoomed out version of the alphabet disappeared when you switched your attention to the A? AB: ...it's kind of in the distance. It's probably further away from me than I can see. ... Big A up there. ... INTERVIEWER: Is that [big] A connected to the alphabet? ... AB: Yeah, it's kind of in the position of where the A is in that alphabet that is far way. ... this [big, closer] alphabet is kind of here A B C.  
                  ... INTERVIEWER: So the zoomed out one has moved into the distance and now you have a new one that's appeared with the bigger letters (20100329\_157-190)


                  ...there's a 4 and a 6 kind of here. ... INTERVIEWER: If you moved your head from side to side like this, how does your perception of the number change? AB: Only it's still kind of like this, so I kind of can't see it, can see it only side on. Erm, sometimes it tilts a little bit so it's kind of bi-stable. ... I can see the back of the 4 [if i move round it and look at the back]. Yeah I can kind of see the 4 backwards. Sometimes it sort of spins around.  ... So it's always kind of bi-stable, but yeah there's kind of one where it's kind of back to front. Because I see it orientated with the front, this side. (20100408\_99-107) ... it's like there's two here. So there's one where the 4 is just like that and then is another one where it's kind of. INTERVIEWER: I've noticed that at the moment you are actually standing still. Can you see both of the different ones even though you are not moving? AB: Yeah, there's kind of one like this and there's one kind of like that. INTERVIEWER: So you have two different forms there? AB: Yeah. (20100408\_125-129)


                  So I've got a 2 here. ...  INTERVIEWER: ...So how does it change when you stand up for example? AB: ... looks like I am kind of above it ... INTERVIEWER: ...if you go exactly above it. AB: Kind of disappeared now. ... Seems to be pretty much vertical. I mean if anything, I just sort of see a thick line there where 2 should be. ... I am also seeing a 2 like that as well simultaneously. INTERVIEWER: Oh, like the same spot, as if it was lying on the table? AB: Yeah. INTERVIEWER: So when did that one appear? AB: Just now. And I was trying to work out what I was seeing. It gets a bit fuzzy when I'm standing directly above it. ... It's sort of different images coming into my mind. One is just of erm the top of the 2. Another is it with it slightly angled. Then another is the 2 just flat on the table. INTERVIEWER: Right. So at least three different kinds of fitting here. AB: Yeah. That's just sort of less stable. INTERVIEWER: If you sit down, what happens with them? AB: So, I've got a 2 flat on the table now. Then I've got the other one is kind of there standing up and flicking between the two. ... INTERVIEWER: Ok, shall we try it again? If you just focus on the one that's standing up and then just rise up while looking at it. What happens to it? AB: ...it seemed to jump for a moment like flat on the table. And then sort of comes back and is standing up. So it's kind of hard to stand up slowly enough ... it seems to flicker. I'm getting images of a 2 going just flat. ... INTERVIEWER: but was the one flat on the table present the whole time... when you were standing up and down? AB: No, it just came into view when I sort of reached there at a certain angle. INTERVIEWER: Is it not on the table right now[whilst you're sitting down] ? AB: It's sort of flickering. INTERVIEWER: And then when you start moving at some point it ... stabilises? AB: Well that was what happened last time. Erm, because you told me to focus on the vertical 2. INTERVIEWER: ...When you were ...focusing on the vertical [2], did it at any time disappear and then the other one [appeared]? AB: Yeah, when I got to a certain height. INTERVIEWER: And then when you were at the top basically stood up, the standing up one came back. AB: Yeah, yeah and then continued to alternate with the [flat one] (20100415\_65-119)

                  + -


                  INTERVIEWER: Ok and can you move them to be in the middle of the space...? So if I said for example can you put a 55 [here]? AB: So I've got one lot over here and one lot over there. (20100305\_116-117).

                  - There's still a number line ["lot"] over here and a number line ["lot"] over there. (20100305\_124)
              + -


              Newinducersappearing during a context transition

              - + -


                When AB attempts to voluntarily alter the context of a concurrent, it appears that a new concurrent is generated in the new context rather than the concurrent moving or the background changing. Multiple representations in a context transition occurs most frequently when the context transition is induced voluntarily or is requested by the interviewer. It may be that under these conditions AB is affording more attention or mental effort to the experience and thus can report more detail on the experience and that the context transition itself is the same wether voluntary or automatic. If this is the case then we may have to revisit our discussions on the section \section{context transition} and include the generation of multiple representations as part of the process\*\*. \*This has not been shown but is suggested for follow-up\*.

                In the context transition to the number line, the primary concurrent initially appears 'large' and is then followed by 'smaller' surrounding sequence numbers (+/-1 item) . The large number is then replicated in the correct position between the smaller numbers.

                "Background [of a white page 56] is melting away. There's a 55 and a 57 appeared first and then a 56 here. But I think there's still a 56 up there. INTERVIEWER: So, does this [new] 56 look different than the one? AB: Much smaller." (20100329\_119-121).

                "[5] Above and in the middle of the 4 and 6. INTERVIEWER: So what happens when you want it to be between the 4 and 6 rather than floating above? AB: I can see a smaller 5 trying to edge its way in, but the big 5 is kind of in its way. So the big 5 wants to be the one that's going to in there, but it's not moving down. ... So as long as I see it from here. Because I look down now and got 4 5 6. Then the smaller 5 has gone now, but there's still the big 5 right here. ... The one down there is a very plain small 5, but this one is kind of big and patterned. All kinds of markings on it (20100329\_141-151). INTERVIEWER: What happened to the small 5? AB: Erm, kind of there if I look down. INTERVIEWER: ...Is it less apparent the small 5? AB: Yeah. When I look up slightly I am well aware of the 4 and the 6 over here. And big 5 is kind of blocking out the smaller one. When I shift my perspective down then I just have the 4 5 and 6 much more normal. INTERVIEWER: And when you look down at the 4 5 6 do you still have the [big] pattern 5 in view? AB: Yeah, I'm still aware of it as floating above." (20100329\_158-163).

                "INTERVIEWER: You said the line connects 55 to 57. AB: 55 to 57. 56 is kind of hovering above [the line]. ... INTERVIEWER: And the 56 is not on the ground [of the corridor]? AB: No. I can get a small 56 appearing briefly on the ground [in the correct position between 55 and 57]. ... but there's still a big 56 hovering above as well (20100305\_40-47). The big 56. Erm, so it's almost like the small one is being magnified. Don't know." (20100305\_51).

                "as soon as I look at the number line, I'm not seeing the [white page or free space] 6 anymore. I'm kind of aware of its presence. But it's not the same 6 dropping. It's like a new 6 gets produced there (20100329\_111).  INTERVIEWER: previously you used the word I think, “dropping down” but now you are saying actually it's more about recreation at the bottom. Which one of the two would you say is more accurately a description of your experience? AB: Well, today I had a 6 down there and a 6 up here for a while." (20100329\_114-115).

                - + -


                  three 6's

                  - + -


                    first 6

                    - INTERVIEWER: So let's say number 6. Can you see the number 6? AB: Yeah, just about here. (20100329\_2-3)


                      I sensed a little bit patterned. Kind of appeared like that, going around like that (20100329\_5)


                      Sort of drawing itself yeah. It's kind of mainly hollow at the beginning but then it towards the end it was kind of filled in, more black (20100329\_7).


                      It was just the blackness was there at the same time the contour kind of appeared. Bit hard to say, it happens rather fast (20100329\_9).


                      INTERVIEWER: So is it still hovering up here, in the air?  <AB agrees> (20100329\_10)


                      5 and 7 down here [on the line] (20100329\_11)


                      The background is a bit unstable, sometimes it's purplish and sometimes it's greenish.  (20100329\_13).


                      it's some kind of floating space, then I can bring it down here. ... Yeah, it's kind of in both places at once. It's kind of getting more down here than up there now. For a while it was in a position between the two (20100329\_17-19)
                    + -


                    second 6

                    - They are kind of standing up on the table, 5 6 7. (20100329\_23).


                      I'm kind of seeing them on the table now. But sometimes I don't see the table at all and just only aware of them as being inside this corridor.(20100329\_25).


                      I can just see the table behind them. (20100329\_39)


                      Just the numbers themselves [no line] (20100329\_45)
                    + -


                    third 6

                    - I'm getting glimpses of another set of numbers over here that really are in the corridor. 5 6 7 <laughter> (20100329\_29)


                      5 and another 7 (20100329\_33)


                      It's the third 6 now (20100329\_35)


                      It's kind of in the corridor. The floor's brown. (20100329\_41)


                      I don't see the table in that at all (20100329\_43)


                      Just the numbers themselves [no line] (20100329\_45)
                    + -


                    (forth 6)

                    - INTERVIEWER: Can you bring in another 6? AB: Yeah, there's one right here. ... got a 5 6 and 7. (20100329\_52-55)

                  as soon as I look at the number line, I'm not seeing the [white page or free space] 6 anymore. I'm kind of aware of its presence. But it's not the same 6 dropping. It's like a new 6 gets produced there [in the line/corridor] (20100329\_111)


                  INTERVIEWER: Previously you used the word I think, “dropping down” but now you are saying actually it's more about recreation at the bottom. Which one of the two would you say is more accurately a description of your experience? AB: Well, today I had a 6 down there and a 6 up here for a while (20100329\_114-115).


                  Background [of a white page 56] is melting away. There's a 55 and a 57 appeared first and then a 56 here. But I think there's still a 56 up there. INTERVIEWER: So, does this 56 look different than the one? AB: Much smaller. (20100329\_119-121).

                  + -


                  [5 "floating"]... Above and in the middle of the 4 and 6 [5 appeared first, 4 and 6 later]. INTERVIEWER: So what happens when you want it to be between the 4 and 6 rather than floating above? AB: I can see a smaller 5 trying to edge its way in, but the big 5 is kind of in its way. So the big 5 wants to be the one that's going to in there, but it's not moving down. ... So as long as I see it from here. Because I look down now and got 4 5 6. Then the smaller 5 has gone now, but there's still the big 5 right here. ... The one down there is a very plain small 5, but this one is kind of big and patterned. All kinds of markings on it.(20100329\_141-151)

                  - INTERVIEWER: What happened to the small 5? AB: Erm, kind of there if I look down. INTERVIEWER: ...Is it less apparent the small 5? AB: Yeah. When I look up slightly I am well aware of the 4 and the 6 over here. And big 5 is kind of blocking out the smaller one. When I shift my perspective down then I just have the 4 5 and 6 much more normal. INTERVIEWER: And when you look down at the 4 5 6 do you still have the [big] pattern 5 in view? AB: Yeah, I'm still aware of it as floating above. (20100329\_158-163)
                  + -


                  INTERVIEWER: You said the line connects 55 to 57. AB: 55 to 57. 56 is kind of hovering above [the line, in free space]. ... INTERVIEWER: And the 56 is not on the ground? AB: No. I can get a small 56 appearing briefly on the ground [in the correct position between 55, 57]. ... but there's still a big 56 hovering above as well (20100305\_40-47).

                  - The big 56. Erm, so it's almost like the small one is being magnified. Don't know. The small one doesn't really have the markings on it (20100305\_51).
              + -


              Unstable concurrents stabilising into multiple representations

              - + -


                In some cases where AB describes and unstable percept, a second concurrent or new representation of the first appears to be generated as the visual experience stabilises. The stabilisation is not immediate and we might speculate regarding the cognitive activities taking place during this time, to include potentially inner speech and focused attention. "...think I am a bit confused about whether it's a 2 2 or a 22 INTERVIEWER: Ok well assuming that it's a 22, if you let your gaze drift towards the right you should encounter the 23 if it is a number line. AB: Yeah, it's bit unstable. So I can kind of see a 23 here and a 24 there, but I'm also getting like a 21 here and a 23 above it. INTERVIEWER: Oh there's two different [representations of 22]! A bit of a confusion going on between the different number lines then?" (20100415\_135-138).

                There is a similar occurence when AB attempting to modify the font of an alphabet representation. Here it appears that the attempted manipulation of the alphabet form lead to the generation of a new percept which stabilised into a uppercase-lowercase sequence. "INTERVIEWER: ...how easy is it to change the big A into a lower case A? AB: I can get the lower case A to flash, but then doesn't stay there. ... On here, black capital A and then a lower case A to the right of it. INTERVIEWER: Oh but it went to a different sequence. AB: Yeah. Now I've got the B next to it, down and to the right of the two A's. Capital B, small B. INTERVIEWER: Oh so it's a different kind of sequence, its capital letters, lower case letters, capital letters, lower case letters. AB: Yeah. So the two A's are next to each other and then the B's are kind of down to the right. INTERVIEWER: So it's more like a step now, like a line. Like a stair with the two next to each other." (20100329\_322-336).

                In another example AB generates a new representation of his alphabet form when exploring the space surrounding the usual concurrent. "INTERVIEWER: ...do you have any sense that there is something to the left of the A? ... AB: ... I kind of got a Z appearing up and to the left of the A for a moment there. Smaller Z. Only after a bit you said is there anything there? ... Now I've got a whole cloud of letters kind of going over here. ... One's towards the end of the alphabet. ... Then I can view X Y Z. ... Can feel them all around me now, there's R S T behind me. ... I've kind of got it curving around now. ... INTERVIEWER: How far are the A and Z apart? AB: Right next to each other. ... the Z is smaller than the A. ... There's two Y's next to the Z though, on top of each other. ... There's like a small Y and then a capital Y... Lower case Y ... it's just like an ordinary Y, lower case Y in black ink. ... so it's stabilised above the upper case Y. (20100329\_290-329).

                - INTERVIEWER: ...do you have any sense that there is something to the left of the A? ... AB: ... I kind of got a Z appearing up and to the left of the A for a moment there. Smaller Z. Only after a bit you said is there anything there? ... Now I've got a whole cloud of letters kind of going over here. ... One's towards the end of the alphabet. ... Then I can view X Y Z. ... Can feel them all around me now, there's R S T behind me. ... I've kind of got it curving around now. ... INTERVIEWER: How far are the A and Z apart? AB: Right next to each other. ... the Z is smaller than the A. ... There's two Y's next to the Z though, on top of each other. ... There's like a small Y and then a capital Y... Lower case Y ... it's just like an ordinary Y, lower case Y in black ink. ... so it's stabilised above the upper case Y. (20100329\_290-329)


                  INTERVIEWER: ...how easy is it to change the big A into a lower case A? AB: I can get the lower case A to flash, but then doesn't stay there. INTERVIEWER: Above it or it's replacing? AB: Same place  ... INTERVIEWER: But if you wanted to do a small A, let's say with a big A. It wouldn't do that or? AB: On here, black capital A and then a lower case A to the right of it. INTERVIEWER: Oh but it went to a different sequence. AB: Yeah. Now I've got the B next to it, down and to the right of the two A's. Capital B, small B. INTERVIEWER: Oh so it's a different kind of sequence, its capital letters, lower case letters, capital letters, lower case letters. AB: Yeah. So the two A's are next to each other and then the B's are kind of down to the right. INTERVIEWER: So it's more like a step now, like a line. Like a stair with the two next to each other. (20100329\_322-336)


                  No, think I am a bit confused about whether it's a 2 2 or a 22 INTERVIEWER: Ok well assuming that it's a 22, if you let your gaze drift towards the right you should encounter the 23 if it is a number line. AB: Yeah, it's bit unstable. So I can kind of see a 23 here and a 24 there, but I'm also getting like a 21 here and a 23 above it. INTERVIEWER: Oh there's two different! A bit of a confusion going on between the different number lines then? (20100415\_135-138)
              + -


               Physical interference causes placement of a new concurrent

              - + -


                Where AB experiences interference from the physical room, a new representation of the weak (interfered) percept generated in a more stable physical location.  "INTERVIEWER: Can you change [free space to corridor]? Is that something that is under your control? AB: Erm, seems hard with the corridor down there. I can have the corridor up here. Got a 7 here and 8 here (20100329\_102-105)."

                - + -


                  INTERVIEWER: Can you change [free space to corridor]? Is that something that is under your control? AB: Erm, seems hard with the corridor down there. I can have the corridor up here. Got a 7 here and 8 here (20100329\_102-105).

                  - INTERVIEWER: Can you still see the one on the floor? Is it still there AB: In a vague sense, the 6 and 7 kind of floating around somewhere over there. I've got a 6 here now... AB: Yeah, there's an 8 somewhere over there on the floor (20100329\_94-97).
      + -


      Concurrent experiences persist

      - + -


         Once a concurrent has been induced, it was found that the experience of the concurrent persisted or was sustained in AB's experience for an unknown length of time without the need for it to be directly refered to. This supports reports of synaesthetic concurrents existing independently of the subject. In this experimental setting this lead to AB's experience being "bogged up with loads of numbers" (20100229\_384) where new concurrents being continually introduced.

        - + -


          sustained

          - + -


            Following induction, individual concurrent percepts generally appear to be 'sustained' in AB's experience. For example, AB reports that a concurrent of "60 has rooted now because we've been talking about it for quite a bit" (20100229\_329). Similarly AB reports that a percept may remain stable even if it is taken out of view or attention is directed elsewhere; once the focus is returned, the same concurrent is described almost as if it existed independently of AB's attention: "My view it went back to the 3. That's all still there as well. (20100229\_388). INTERVIEWER: What if you... take them out of view completely...  Do they remain exactly where they were before? AB: Yeah still 5, 6, 57." (20100305\_131-132). This leads on situation where AB's experience contains multiple independent concurrents, for example, "INTERVIEWER: Do you have any number line ready that we could do that with? AB: Erm, I've got all sorts of number lines." (20100408\_386-387). On occasion, this persistence of concurrents lead to confusion not only in descriptions of the experience (deducing which concurrents where being refered to) but also where AB found there were too may concurrents present to concentrate on a single item, causing him to feel "bogged up with loads of numbers" (20100229\_384).

            - there's almost like that 60 has rooted now because we've been talking about it for quite a bit  (20100229\_329).


              INTERVIEWER: When you look at the... alphabet line, are you aware of having to say H in order to keep it in focus? AB: No, I think once it's there I don't have to say H (20100408\_40-41).


              My view it went back to the 3. That's all still there as well. (20100229\_388)


              INTERVIEWER: What if you... take them out of view completely so if you look behind you so immediately you don't see either of them and then you turn around again. Do they remain exactly where they were before? AB: Yeah still 5 6 57 (20100305\_131-132).


              INTERVIEWER: Do you have any number line ready that we could do that with? AB: Erm, I've got all sorts of number lines (20100408\_386-387).
  + -


  Concurrent contexts

  - + -


    AB's concurrents consistently appear in a range of 'contexts' which describe the perceptual experience around the concurrent in the mental room. In the number form AB generally reports the concurrents as initially appearing against a "white page" and then when viewing a section of the number line, concurrents appear in a "corridor" with the black line cross through the width (see fig\*\*). Where no white page or corridor is reported, there is a consistent description of an illuminating blue-green background, a context which we have termed 'free space'. For the alphabet form, AB describes a similar free space context, the same white page and a "cave" context which has a distinctly "muddy" atmosphere. We have also identified a tentative grouping of concurrents which have a 'presence' based on reference to spatial relationships to seen concurrents, but are not directly seen themselves.

    - + -


      Free space

      - + -


        The free space context consists of a distinctly coloured background (light green-purple for numbers and darker purple for letters) which is further away from AB than the concurrent itself. This background colour appears to provide some contrast against which the concurrents can be viewed.

        - + -


          free space

          - + -


            The free space context is identifiable by a lack of reference to surrounding numbers/letters or any other specific context. The free space context also has a distinctly coloured background of light purple-green for numbers and a darker purple for letters. This background appears to itself provide a some contrast for the number/letter concurrents.

            - + -


              number free space features

              - + -


                The free space context is identified by reference to specific colouring of the background and a lack of reference to features of the white page or corridor context.AB describes the free space background as 'unstable', "sometimes it's purplish and sometimes it's greenish." (20100329\_13), "a more purple background." (20100329\_37), "little sparks of sort of green and umm... purple I think." (20100215\_117), "It's not really smooth; it's kind of a bit lumpy sort of texture" (20100415\_31). AB reports that this background "doesn't make the [physical] room coloured. It's just like another world [mental room] that I can focus my attention on, or I can bring my attention back to the [physical] room (20100415\_55)".

                - there's a little bit of err, like a green background to the line, when it first appears (20100215\_134)

                  + -


                  green (20100215\_134)

                  - *blurry (20100215\_142)*

                  unstable background, like almost sort of like psychedelic. (20100215\_166)


                  When I see the number line that's always against the more kind of coloured background (20100415\_61)


                  a bit unstable, sometimes it's purplish and sometimes it's greenish (20100329\_13)


                  a more purple background (20100329\_37)


                  purplish background (20100415\_31)


                  it's kind of like a dunno sort of a turquoisey background. Not really sure what colours exactly (20100415\_49)


                  Oh no it doesn't make the room coloured. It's just like another world that I can focus my attention on or I can bring my attention back to the room (20100415\_55)


                  There's a little bit of colour in there as well (20100215\_116) As kind of, erm, sort of, like little sparks of sort of green and umm... purple I think (20100215\_117)


                  There's no line with the digits now, so 54 5 56 57. They're all just standing out (20100229\_452)


                  It's not really smooth; it's kind of a bit lumpy sort of texture (20100415\_31)
              + -


              alphabet free space features

              - + -


                 A free space context is also described for AB's alphabet form, although with a darker background: "It's kind of against a dark sort of purplish background." (20100415\_244). This background itself provides contrast to the letter concurrents and may be a source of light: "But the purplish background seems kind of luminescent itself as if it's giving off its own light over there." (20100415\_268).

                - It's kind of against a dark sort of purplish background (20100415\_244)


                  it's a bit kind of translucent, a bit like the texture of the iris of an eye (20100415\_262)


                  purplish background (20100415\_266)


                  But the purplish background seems kind of luminescent itself as if it's giving off its own light over there (20100415\_268)


                  it just kind of goes beyond the eye can see. So there's like a vanishing point and there's not enough light to illuminate it beyond a certain point (20100415\_284). eventually you can't see any further (20100415\_286)


                  seems kind of ghost like, like you could actually go through it (20100415\_288-290).
          + -


          backgrounds

          - + -


            Where AB refers to the 'background' of a context, this is described as being "further away" from AB than the concurrent (20100222\_18), such that the background is physically behind the concurrent.

            - The background's further away. (20100222\_18)
      + -


      White page

      - + -


        The white page is often the first context upon which a concurrent (number and alphabet) is presented. It is described as an A4 portrait sheet of paper, presented vertically in front of AB's eye line. The concurrents appear in a font size to approximately fill the white page, where numbers with more than one digit presented in a smaller font to accomodate the fixed size of the page. It is suggested that the white page provides contrast against which the black concurrents can be easily viewed.

        - + -


          white page

          - + -


            The white page is reported in AB's number, alphabet and time forms. It is described as similar in size as an an A4 pi.e of paper presented vertically in front of him, forward and below his eye line. The area around the white page is described with features and colours similar to the free space percept. Concurrents themselves appear centred on the page in a 'font size' which would fill a "good proportion of the paper... as if there's only room for that one digit... with a good margin around the edge" (20100215\_146-148). The A4 size of the white page appears fixed but the font size of the concurrents appears variable such that a larger number with more digits will appear with a smaller font size such that all digits fit on one single page.

            The line is below the white page and there is a suggestion that AB does not experience both the line and the page simultaneously (although this may be an effect of attention switching between the two). It is suggested that the white page provides contrast against which the concurrents can be seen in the mental room. AB alludes to this in reporting that it is easier to see the letter concurrents in the cave against the white page (20100329\_399), and in a short experiment where AB has his eyes closed and reports that the white page is more "stubborn" (20100305\_75).

            - + -


              present in forms:

              - + -


                The white is described consistently in association with AB's number, alphabet and time forms. It is also reported to form the backdrop to the graph and equation percepts, however in this context is is described as more of a "whiteboard" which AB can "scribble on" (20100320a\_210).

                - + -


                  alphabet form

                  - letters can appear against the white page (20100329\_227)


                    multiple letters on the white page (20100329\_241)

                    + -


                    white paper with gridlines on it (20100329\_347)

                    - requires more forceful removal (20100329\_343)

                    A4 proportions (20100329\_389).


                    INTERVIEWER: it's still rectangular. There's no background showing around the sides. AB: Yeah, it's like the same type of background that is revealed.  INTERVIEWER: Oh it's already around it? ... AB: Kind of like a purple layer here, a green layer here and some black (20100329\_392-395)
                  + -


                  time

                  - one of the clocks appeared on a white page (20100320b\_46)
                  + -


                  graphs

                  - whiteboard (20100320a\_120)


                    Just a white page with the formula (20100415\_370)


                    It's almost like there's a white board I can scribble on (20100320a\_210).
              + -


              perspective

              - + -


                The white page is described as forward and below AB's horizontal eyeline. In this position, the page itself is described as in a portrait orientation and roughly the size of an A4 pi.e of paper.  \*\*fig. white page against x-y-z axis

                - So now I'm kind of more looking down on the page (20100215\_26)


                  I'm looking, I'm looking erm, directly at the page. The page is horizontal in front of me  (20100215\_153)


                  the page is err, sort of upright, portrait, err orientation (20100222\_74)


                  I'm looking erm, directly at the page. The page is horizontal in front of me but then the number line is kind of down. (20100215\_153).
              + -


              background (around the edges) similar free space

              - + -


                The area outside of the white page is described with similar colours as the free space context ("a purple layer here, a green layer here and some black. (20100329\_395)"), although not in exactly the same manner. It is worth noting that this specific feature was not explored in depth thus any similarities between the area surrounding the white page and the free space context may be coincidental at this stage.

                - Kind of like a purple layer here, a green layer here and some black. (20100329\_395).
              + -


              relation to line

              - + -


                The 'line' is described as being below the page, "The page is horizontal in front of me but then the number line is kind of down." (20100215\_153)  \*\*fig. line and page on x-y-z axis. AB suggests that he can see the page and the line at the same time, although his language use is slightly ambiguous and may suggest that in switching his attention between these two context his perception of the other is diminished:  "I can definitely see it being kind of located below where the page *was*." (20100215\_155).  INTERVIEWER: "Can you see the number line at the same time [as the white page]? AB: Yeah, *when* I see the number line it's going like this and below." (20100229\_49-51).

                - The page is horizontal in front of me but then the number line is kind of down. (20100215\_153)


                  I can definitely see it being kind of located below where the page was (20100215\_155)


                  so at one point it hits the margin ... I then look for where the number 20 is it keeps zooming out, and it doesn't ever reach the edge of the page (20100215\_220).


                  INTERVIEWER:  Can you see the number line at the same time [as the white page]? AB: Yeah, when I see the number line it's going like this and below (20100229\_49-51).
              + -


              relation to percept

              - + -


                The concurrents themselves are experienced in isolation on the white page. They appear as centered and "filling good proportion of the paper. Like as if there's only room for that one digit...  With a good margin around the edge, but no room for anything else." (20100215\_146-148).

                - Just directly above on the page (20100229\_79)


                  the number one... is, ahm, sort of, sort of appearing in the middle of the page (20100215\_11)


                  So the first thing is the digits ... against a kind of white background (20100215\_105)


                  there's a white background and the number revealed itself from the bottom up (20100215\_111)


                  It starts off right in front of me as a... as like on a white background, kind of, like on a rectangular sheet of paper, and filling good proportion of the paper. Like as if there's only room for that one digit. Sort of centred ...  With a good margin around the edge, but no room for anything else (20100215\_146-148).
              + -


              percepts scale to fit on page

              - + -


                The size of the white page is fixed in relation to the concurrents, that is, the font size of the concurrent 'scales' to fit on the white page: "so you have to scale down the width [of the concurrent] in order for them to fit where erm, into the width of the page, and in so doing it seems like that the height has been scaled proportionately, thus giving you more room at the top and the bottom so they're kind of in the middle of the page." (20100222\_88).  "if it [the font size of 56] had been as big as the number five, they wouldn't have fitted [on the white page], there wouldn't have been enough err width on the page for them." (20100222\_82). "So they're scaled down in order to fit the two characters on the page." (20100222\_86). "It seems like the 56 is err actually shorter aswell, so there's more, more white at the top. But that makes sense doesn't it, because ahm, if... the five and the six in 56 had been as tall as the... single number five, then... there wouldn't have been room for it.... So they're scaled down in order to fit the two characters on the page." (20100222\_86).

                - so it's got to be in a smaller font now, to fit on this, err page (20100222\_74)


                  so there's sort of more white at the top and bottom now because the five and six occupi. the whole width of the page (20100222\_74) (20100222\_76). there's equal amounts [of white] at the top and the bottom (20100222\_78)


                  if it had been as big as the number five, they wouldn't have fitted, there wouldn't have been enough err width on the page for them (20100222\_82)


                  if the five ... and the six in 56 had been as tall as the ... the single number five, then ... there wouldn't have been room for it. So they're scaled down in order to fit the two characters on the page (20100222\_86)


                  so you have to scale down the width in order for them to fit where erm, into the width of the page, and in so doing it seems like that the height has been scaled proportionately, thus giving you more room at the top and the bottom so they're kind of in the middle of the page (20100222\_88).


                  they are all kind of on the page [root2 decimals], kind of all crowding around each other, much sort of closer together (20100320a\_19)


                  INTERVIEWER: So when I said 56, you said the font was smaller, how do you know that? AB: Because if the... if it had been as big as the number five, they wouldn't have fitted, there wouldn't have been enough err width on the page for them. (20100222\_81-82).

                  + -


                  It seems like the 56 is err actually shorter aswell, so there's more, more white at the top. But that makes sense doesn't it, because ahm, if... the five and the six in 56 had been as tall as the... single number five, then... there wouldn't have been room for it.... So they're scaled down in order to fit the two characters on the page. (20100222\_86).

                  - but they're scaled down so, so you have to scale down the width in order for them to fit where erm, into the width of the page, and in so doing it seems like that the height has been scaled proportionately, thus giving you more room at the top and the bottom so they're kind of in the middle of the page. (20100222\_88).
              + -


              provides contrast

              - + -


                We speculate that the white page provides contrast for the concurrents to be viewed against the colouring of the physical room (it should be noted that the concurrents are most often black). This is supported by AB's report that he has difficulty identifying the concurrents when they haven't got the white page behind them (20100415\_43) and also when he is also suggested when AB is exploring a letter concurrent in the cave: "[the cave] it's quite kind of dark. It's almost better to have the white page there, because it helps me to see the A." better (20100329\_399). In a short experiment, AB explores a concurrent percept with his eyes closed. In this inherently darker scene, he describes the white page as being "more stubborn", as if the concurrent "wants to be behind a white background." (20100305\_75).

                - [the cave] it's quite kind of dark. It's almost better to have the white page there, because it helps me to see the A better (20100329\_399).


                  staying more on a white background 56 perhaps ... White background is kind of melting away again from the bottom ... Little bit more stubborn in the top left corner, it kind of wants to be behind a white background (20100305\_75).


                  Erm, they are a bit more vague. They haven't got the background behind them, so I can't see them as well (20100415\_43)
              + -


              interaction with percept patterns

              - + -


                The patterning or texture which is described on some percepts occasionally appears to "spill" onto the white page (e.g. 20100229\_436)

                - patterning can spill onto the page (20100229\_436)


                  So I can get kind of get white lines going across the numbers [56] so it's kind of black, mainly black but sometimes you get whiteness fills the bottom bit of the number, kind of goes up to the top and then goes black again. So a shimmering effect (20100229\_466)2
      + -


      Corridor

      - + -


        When viewing a sequence of numbers in the form, percepts are reported to exist in the context of a 'corridor'. The corridor is described with walls, a floor and a ceiling all in a reddy-orange colour. AB's perspective on the corridor is as if his head is in one of the walls. Concurrents appear in the middle of the corridor with the line bisecting the width such that the surrounding concurrents are 'outside' the corridor, beyond the walls or above the ceiling (depending on the gradient of the line).

        - + -


          corridor

          - + -


            The corridor is the usual context for AB's number form when viewing a sequence of numbers rather than isolated ones. The number line bisects the width of the corridor at the angle of AB's overall number form. The corridor has distinct walls, floor and ceiling of an opaque or dense orangy-red colour. The ceiling is described on two occasions with reference to a source of light (20100222\_159 and 20100222\_326). The far end of the corridor is well lit and may also provide a light source or contrast for the concurrents, whilst the entrance to the corridor has a more naturalistic feel with sky and clouds (20100404\_297-299). AB is generally positioned with his head in the one wall of the corridor. The induced concurrent is normally reported as floating in the middle of the corridor (20100320a\_47) whilst those immediately surrounding it are against the wall or just outside the corridor. Concurrents with multiple decimal places are experience with the decimals trailing down the length of the corridor. The size or reporting of the size of the corridor varies; on one occasion it is not much bigger than AB's head (20100222\_126), on another it is as wide as AB is tall (20100229\_182). It may particularly expand width ways to encompass more numbers in the form, e.g.  "It's kind of like the corridor wants to enlarge and have the 7 over here (20100329\_107)."

            - + -


              features

              - + -


                The corridor context was initially described as a 'room', with the concurrents 'floating in the middle' (20100222\_25; 2010022\_35; 20100222\_64). This description later transformed into a 'corridor' with walls, a ceiling and a floor (20100222\_117).   The walls of the corridor are "quite opaque (20100329\_42)" although "not like a hard surface like the table, if you put anything behind [the wall] it would go into the fog and you wouldn't be able to see it (20100408\_349)." AB reports a "slightly ghostlike quality of the wall of the corridor where it... doesn't feel hard but, feels like you couldn't penetrate it very far (20100408\_369)." These walls are described relatively consistently as a "orangey reddy greeney colour (20100222\_148)"  elsewhere, "orangey browney, greeney colour (20100222\_103)", "greenish, reddish orangey (20100229\_108)" or "orangey, greeny colour (20100408\_225)", however it is clear that AB has some difficulty in describing the exact colour; "this colour that I can't quite make out exactly what colour it is (20100222\_49)." The floor of the corridor is "Kind of the same as the wall (20100222\_31)" and appears to have a similar opaque characteristic: "The five's kind of disappeared now completely, into the floor! (20100222\_186)."

                The corridor is repeatedly referred to as having a ceiling (e.g. 20100222\_117) and on two occasions with reference to a source of light; with "little circles of light going down the corridor" (20100222\_159) and opening up into a "Sun roof" (20100222\_326). When this sun roof is opened it reveals a "kind of white space. It's not as pure a white, there's kind of a creamy colour in there and sort of, some very soft blues and yellows and things (20100222\_337). It's quite nice to look at. Almost like a cream cake (20100222\_338)". AB also reports that the corridor itself has a "kind of a yellowish light kind of permeating it (20100222\_146)."

                The size of the corridor appears to vary. On one occision the corridor is reports to be not much bigger than AB's head (20100222\_126), on another it is as wide as AB is tall (20100229\_182). The orientation of the line and form suggest that the corridor may expand width ways to encompass more numbers in the form, e.g.  "It's kind of like the corridor wants to enlarge and have the 7 over here (20100329\_107)."

                - kind of in the middle of the room  (20100222\_25)


                  And then the five is kind of floating in this room (2010022\_35)


                  sometimes it's sort of in that room that I was describing (20100222\_64)


                  the room that it's in. This is more like a corridor (20100222\_117)

                  + -


                  walls

                  - + -


                    colour

                    - I think it mainly is green, and there's appearing a kind of a wall (20100222\_20)


                      There's kind of a greeny, green-brown, orange, sort of somewhere in there, it's not... it's not that stable. (20100222\_27).


                      this colour that I can't quite make out exactly what colour it is (20100222\_49).


                      orangey reddy greeney colour (20100222\_148)


                      Kind of an orangey browney, greeney colour (20100222\_103).


                      There's kind of a green-like quality to it (20100222\_164)


                      greenish, reddish orangey (20100229\_108)


                      this colour I'm not quite sure what it is. This greeny orangey colour (20100305\_55)


                      orangey, greeny colour (20100408\_225)
                    + -


                    properties

                    - quite opaque (20100329\_42)


                      not that dark (20100329\_415). Provides sufficient contrast to see black numbers.


                      a bit fuzzy, so it's not like a hard surface like the table, if you put anything (physical) behind it would go into the fog and you wouldn't be able to see it (20100408\_349).


                      The wall of the corridor is a bit kind of best defined as hard (20100408\_359).


                      There's again all that slightly ghostlike quality of the wall of the corridor where it's kind of, doesn't feel hard but, feels like you couldn't penetrate it very far (20100408\_369).
                  + -


                  floor

                  - you can sort of see a floor underneath (20100222\_22)


                    Kind of the same as the wall (20100222\_31)


                    the floor is not so stable I can't tell if it's kind of wooden, or... exactly what colour it is... (20100222\_97)


                    extends as awareness expands (20100222\_49)


                    The five's kind of disappeared now completely, into the floor! (20100222\_186)
                  + -


                  ceiling

                  - it's kind of like touching the ceiling of the... pretty much touching the ceiling of the room that it's in (20100222\_117)


                    Cause if I look slightly away from the five, then I can see, see the top of the wall. So it's kind of a wall here and then there's a ceiling coming this way, ahm... I think looking up, the five isn't quite touching the ceiling (20100222\_121)


                    Erm, kind of like a sort of sun roof has opened up in the corridor (20100222\_326). Ahm, it's just like a white hole (20100222\_328) it's just err, it's like a square hole. It goes from one wall to the other. Ahm The whole corridor's becoming a bit less stable now, it's kind of err, the roof from this point onwards has just kind of err melted away (20100222\_330). kind of white space. It's not as pure a white, there's kind of a creamy colour in there and sort of, some very soft blues and yellows and things (20100222\_337). it's quite nice to look at. Almost like a cream cake kind of thing (20100222\_338)


                    All you get is little light overhead lights. It's like a ceiling with little electrical lights. Little circles of light going down the corridor (20100222\_159). I haven't seen any numbers go in to those white lights ((20100222\_165)
                  + -


                  lighting

                  - there's kind of a yellowish light kind of permeating it (20100222\_146). But I still can't tell what colour the walls and things are... kind of orangey reddy greeney colour (20100222\_148)
                  + -


                  size

                  - maybe a little less wide than I am high (20100229\_180)


                    Seems to change a bit by trying to imagine myself lying across it. It's kind of, seems like there is room for me to lie down. So maybe a bit wider than I am  (20100229\_182)


                    It's kind of like the corridor wants to enlarge and have the 7 over here. <laughter> So that table isn't in the way (20100329\_107)


                    Yeah, got a big corridor today (20100329\_407)


                    Ahm, I feel a bit crushed, like there's not really room for me to stand there, and there's just, somehow I've sort of just got my head in there (20100222\_132)


                    So I kind of feel like my head's sort of sticking up through the floor of this corridor and there's not really much room in there for much more than my head (20100222\_136). So I kind of see across the width of the corridor (20100222\_146).
              + -


              entrance and exit

              - + -


                The far end of the corridor is described as white and possibly a providing a source of light (20100222\_186; 20100222\_202; 20100229\_155; 20100408\_281). The entrance is reported as having with a naturalistic feel, with references to outdoors (20100408\_285), sky (20100404\_297) and clouds (20100408\_299).

                - + -


                  exit

                  - It seems like there's a source of light either end (20100222\_186)


                    I see the end now, just white... it's very long (20100222\_202)


                    I look down that end and there's a white light (20100229\_155)


                    it's just a square end of the corridor and it's white (20100408\_281)
                  + -


                  entrance

                  - kind of whiteness at the end, at the left hand end of the wall. It just kind of fades into this kind of whiteness (20100222\_33)


                    the left hand end of it is kind of white... I think the right hand end is white as well (20100222\_124)


                    It seems like there's a source of light either end (20100222\_186)


                    Kind of a white light (20100222\_206)


                    Just outdoors I guess (20100408\_285). just imagine a white space out there (20100408\_289)


                    For a moment I got some kind of blue sky type thing (20100408\_297). Sometimes it goes kind of blue with white puffy clouds going by (20100408\_299). So it is like a natural light (20100408\_301)
              + -


              perspective

              - + -


                AB reports that his head is is inside the corridor, through the wall or floor; "I'm kind of standing on one side of the corridor, looking at the other side (20100222\_124)". "Ahm, I feel a bit crushed, like there's not really room for me to stand there, and there's just, somehow I've sort of just got my head in there (20100222\_132)."  So I kind of feel like my head's sort of sticking up through the floor of this corridor and there's not really much room in there for much more than my head (20100222\_136)."

                - I'm kind of standing on one side of the corridor, looking at the other side (20100222\_124)


                  So I'm inside the corridor (20100222\_128)

                  + -


                  Ahm, I feel a bit crushed, like there's not really room for me to stand there, and there's just, somehow I've sort of just got my head in there (20100222\_132)

                  - So it's almost like all the walls, you can kind of melt through them (20100222\_134)

                  So I kind of feel like my head's sort of sticking up through the floor of this corridor and there's not really much room in there for much more than my head (20100222\_136). So I kind of see across the width of the corridor (20100222\_146).


                  So now I've kind of got two fives in this room. If I look over there I've still got this one, if I look here I've got this one (20100222\_273). Well, yeah, this one's just kind of is against the wall, up here, and kind of just about fits, the corridor's just about high enough for that five to fit, and the other one's just here (20100222\_285).


                  It looks like a corridor because if I look down there it's kind of, yeah I do see the wall, it's kind of behind me. (20100222\_126).


                  I've kind of moved along the corridor and the five is now to my left. (20100222\_142)


                  I've kind of shifted perspective now so I'm kind of seeing it down here. I'm looking down quite steeply at it. (20100229\_143)
              + -


              relation to percepts

              - + -


                 Concurrents are reported as either floating in the middle of the corridor (20100320a\_47) or against the wall. Those which are centrally positioned are generally the directly induced concurrent whilst those against the walls are the surrounding concurrents in the number form; "it's not attached to the wall, it seems like it's kind of leaning against the wall (20100222\_227)."

                When AB is examining irrational numbers such as e and the square root of two, the infinite decimal places are presented down the length of the corridor, traveling off into the distance. We also find multiple representations of a single concurrent down the length of the corridor (20100229\_358).

                - it's not attached to the wall, it seems like it's kind of leaning against the wall (20100222\_227)


                  INTERVIEWER: If you look down the new 60, does it looks like it's in the same space as the old 60? AB: So it's on the white thing but then that goes away and then it's kind of now on the corridor, further down the corridor from this one (20100229\_357-358).


                  The square root of 2 is kind of floating above in the middle of the room (20100320a\_47)


                  Because the numbers live in the corridor (20100408\_259)
              + -


              relation to line

              - + -


                 The line for the number form appears along the floor of the corridor (20100222\_49; 20100222\_51). In this context, AB is able to report that the line itself has a 3D form: "Yeah I would say it's kind of sticking up above the floor. So it's kind of so the bottom, the bottom of it is resting on the floor, and then... there's almost like a very, long rectangle, black that's lying, sort of standing up on the, on the floor (20100222\_58)."  Where it reaches the wall, AB describes the line as "going into the wall of the corridor. Hitting the wall of the corridor and the line would go through the wall (20100229\_165-166). "

                - it's kind of along the floor (20100222\_49)


                  The line seems to be on the floor (20100222\_51)


                  Yeah I would say it's kind of sticking up above the floor. So it's kind of so the bottom, the bottom of it is resting on the floor, and then... there's almost like a very, long rectangle, black that's lying, sort of standing up on the, on the floor (20100222\_58)


                  like it has height coming out from the floor (20100222\_56)


                  never quite hits the sort of wall at the back (20100222\_99)


                   I can see the number line as going in. there's more going into the wall of the corridor (20100229\_165). hitting the wall of the corridor and the line would go through the wall (20100229\_166). it's coming from diagonally intersecting the corridor (20100229\_169).


                  piit is going along the corridor. So 3 is erm directly to the left of pi(20100229\_186)
      + -


      Line

      - + -


        The line is present in any form where there are continuous values (i.e. the number and calendar forms but not alphabet). The line is itself 3 dimensional and described as a long black rectangle. It is below the white page such that AB has to look down from the white page to see it; in the corridor context it is resting on the floor of the corridor, bisecting it's width. Concurrents hover just above the line or a small gap in the line. The line appears to play a role in enabling AB to locate concurrents as he follows the changes in gradient to locate the required concurrent.

        - + -


          line

          - + -


            AB reports a 'line' feature in his number and calendar forms. AB reports that the line ios not present in his alphabet form as the alphabet has no continuous values (20100329\_306). The line is seen both in the free space context and in the corridor of the number form. The line is black and has a 3D form like a "very long rectangle" (20100222\_58). The line is below the white page in the vertical plane such that AB has to look down from the white page or free space to see the line. The base of the line rests on the floor of the corridor (bisecting the corridors width) and follows the gradient specified in the number form. If we consider the concurrents to be 'fixed' in their position in external space, the description of the orientation of the line suggests that the number line exists as if extending into space in front of and away from AB (i.e. in the x-z plane) such that in the corridor context iot bisects the width of the corridor. In the white page context AB had adopted a rotated mental view point such that the z direction has become the x and the line is horizontally below the white page. In the free space context, the number concurrents "hover" just above the line (20100215\_172) or the line appears with a gap into which the concurrent would fit if viewed in the corridor (20100222\_95), which acts as a "marker for where the [number] should go." (20100222\_109). There is also a suggestion that the line functions as a reference for directing AB's gaze or attention to other concurrents, as AB follows it's gradient towards where he 'knows' another concurrent to be located (e.g. 20100320a\_298).

            - + -


              present in forms:

              - + -


                The number line has previously been reported in number form synaesthesia {\cite[]}. AB states that the line is not present in his alphabet form (20100329\_221) as the alphabet has no continuous values (20100329\_306). In agreement with this, AB reports the presence of a line in his calendar form, which is continuous.

                - + -


                  not present with alphabet

                  - there's no line (20100329\_221)


                    INTERVIEWER: You said that the alphabet doesn't have a number line because there's no continuous values. <AB agrees> (20100329\_306) - must have been outside of the interview.
                  + -


                  calendar

                  - whatever day I'm focusing on I sort of see that number, but then I am aware of the line around it (20100320a\_318)


                    so a number appears and there's like a line down here (20100320a\_320)
              + -


              background

              - + -


                The number line itself is often described as itself appearing on a coloured background which is similar to the background of the 'free space' context.  The line background is "green" (20100215\_134), "blurry" (20100215\_142) and "unstable... like almost sort of like psychedelic. (20100215\_166)"

                - there's a little bit of err, like a green background to the line, when it first appears (20100215\_134)

                  + -


                  green (20100215\_134)

                  - blurry (20100215\_142)

                  unstable background, like almost sort of like psychedelic. (20100215\_166)


                  When I see the number line that's always against the more kind of coloured background (20100415\_61)
              + -


              features

              - + -


                The number line itself is described as 'black' (20100215\_30; 20100215\_136) and often "suspended in, in mid-air (20100215\_138)" (although see also sec:corridor where line lines up with table top\*\*). \*\*It is also describes as having depth and width, "Yeah I would say it's kind of sticking up above the floor. So [...] the bottom of it is resting on the floor [of the corridor], and then... there's almost like a very, long rectangle, black that's lying, sort of standing up on the, on the floor [of the corridor] (20100222\_58)." As reported elsewhere, AB's number line has distinct angles, turns or corners at specific numbers (20100215\_224).

                - the line itself is black, so the background... Couldn't really pay so much attention to the background... (20100215\_136)


                  it seems a bit like it's sort of suspended in, in mid-air, the line. (20100215\_138)

                  + -


                  bend

                  - there's a clear right hand, there's a bend at the number ten and then the continuation of the number line beyond ten is going to go off, ahm in a different direction (20100215\_28)


                    this is a two dimensional corner (20100215\_41)


                    Err, it's quite, quite sharp (20100215\_144)


                    the shape of the line is always the same and the, the corners are always in exactly the same place (20100215\_224)
                  + -


                  left to right

                  - I see a... see it hovering above a line that's sort of below me like that, sort of where I'm pointing, so looking at an angle away from me, going away from me and from left to right (20100222\_5)

                  i just see a line just ahm, like a black line (20100215\_30)
              + -


              relation to corridor

              - + -


                The line crosses through the walls of the corridor rather than running parallel within it.   \*\*fig. drawing\_20100229\_corridor-axis. The gradient of the corridor reflects the bends in the whole form. The line is not seen outside of the walls of the corridors although AB reports he can "sense it kind of behind the wall (20100229\_366)".

                - it drops down here in the corridor and then there's a line going off that direction (20100229\_303)


                  So this line always constantly moving around so. It's never like one particular position with the wall but this is a general feature there's a line just kind of like that. The line goes into the wall, sort of maybe somewhere there, slightly above the floor (20100229\_228)


                  drawing\_20100229\_corridor-axis


                  Not really much room for a number line, because the five is kind of pretty much against the wall (20100222\_289). They're not on a number line, no (20100222\_291).


                  I can sort of, kind of sense it kind of behind the wall. Because the 60 is right against the wall and the number line would be there (20100229\_366)


                  so I can't see the line beyond 6. So the 6 is parked right against the wall (20100329\_229)
              + -


              relation to percept

              - + -


                Unless the number in question appears at a bend or corner in the number line, there is no visual indication on the line as to where the digits of that number should be positioned, "it just 'hovers' above the line." (20100215\_172).

                Before the context transition to the corridor, i.e. in a white page or free space context with the surrounding numbers from the sequence smaller and below, AB describes a 'gap' into which the larger (free space) number would 'fit' (20100222\_95), which acts as a "marker for where the [number] should go." (20100222\_109).

                - you said the number ten, ahm, I guess that, that appears with a number line behind it and a number line in front of it (20100215\_28)


                   I see, i see again a kind of line with just the number twenty in the middle of... just written in big. So that'll occupy more space than the width of the line (20100215\_41)


                  the number five is sort of erm, suspended, yeah it's kind of suspended above the line (20100215\_83)


                  It's just hovering above it. So there's no, there's no mark precisely where on the line that number five should be, it's just... (20100215\_172).


                  fractions - The line looks pretty much the same as before. There's a number 1 here and there's a line and number 2 peering to the left hand side of the line. Slight gap in er the line at number 2 (20100229\_53)

                  + -


                  56 is kind of above the number line in big. 57 is sort of lower down and smaller as well, just like the 55 (20100305\_35). So actually there's only a line between 55 and 57, so 56 is kind of above (20100305\_39).

                  - 56, it's kind of oscillating a bit between having a line between 55 and 57 and having a little 56 in there and 56 appears as the line kind of goes away (20100305\_53).

                  the white background kind of melts away, and then it's floating above a black line (20100222\_49)

                  + -


                  gap

                  - There's a like a little gap in the number line above where 56 is sort of hovering... (20100222\_93)


                    Just a gap in the line. Seems like you could probably just about squeeze those two numbers five and six into that gap. (20100222\_95)


                    It's kind of like a marker for where the 56 should go (20100222\_109)


                    I can lower the two numbers and they sort of fit nicely in that gap...just to give a bit of space for those two numbers to breathe (20100222\_113)


                    No, no gap for five... hovering above a line, kind of a tighter space there, the five is occupying (20100222\_115)


                    Just a gap in the line. Oh I see the line, just a gap and more line and just comes in here (20100320a\_61).
              + -


              perspective

              - + -


                The line is vertically below the white page (20100215\_153; 20100222\_5) crossing AB from left to right. It appears that AB performs an automatic change in perspective or mental view point depending on which feature of the percepts he is focusing on. For example, "AB: When I see the actual digits themselves, the first thing I think of in the x z plane [moving horizontally away from AB]. INTERVIEWER: Yes, but there is no line in the x z plane is there? AB: No, only digits. So the line is always in the x y plane [horizontally left to right]." (20100229\_291-293).  If we are to consider the line and concurrents themselves to be 'fixed' in external space, when viewing a concurrent in isolation against a white page with the line below, AB has rotated his mental view point such that the z-axis becomes the x-axis. When viewing the number line in the corridor, the perspective is returned to the x-y view with the line extending into the z plane. "When I see the actual digits [i.e. against the white page] and the line is in the vertical plane, but er in front of me. But when I see the number line rather than the digits that's when I see the line is in the horizontal plane on the floor [of the corridor, with the line in the x-z plane)]." (20100229\_261). See figure \*\* for explanation.

                - I see a... see it hovering above a line that's sort of below me like that, sort of where I'm pointing, so looking at an angle away from me, going away from me and from left to right (20100222\_5)


                  when I see the actual digits and the line is in the vertical plane, but er in front of me (20100229\_261)


                  AB: When I see the actual digits themselves, the first thing I think of in the x z plane. INTERVIEWER: Yes, but there is no line in the x z plane is there? AB: No, only digits. So the line is always in the x y plane (20100229\_291-293)


                  I'm looking directly at the page. The page is horizontal in front of me but then the number line is kind of down (20100215\_153).


                  I'm kind of seeing the five right in front of me but the line is... below, I have to look... down in front of me to see the line, the number five is right in front of me (20100215\_170).


                  when I see the actual digits and the line is in the vertical plane, but er in front of me. But when I see the number line rather than the digits that's when I see the line is in the horizontal plane on the floor (20100229\_261).


                  A different thing during the hypnosis session where I was kind of floating above number line. I was kind of looking down at it (20100329\_409).

                  + -


                  looking down

                  - So now I'm kind of more looking down on the page... I can sort of see a line going off, ahm, like the continuation of the number line going off (20100215\_26)


                    I'm sort of looking down on, yeah, if they're on a page... it's much more, err, close, it's closer to the horizontal, err, it's definitely not a horizontal line (20100215\_65)


                    from where I'm centred, I'm looking, I'm looking sort of, it's kind of ahead of, ahead and down from where I'm there, from where my ego's located. (20100215\_151)

                    + -


                    [I can bring a free space 5 into the correct position on the line] So as long as I see it from here. Because I look down now and got 4 5 6. Then the smaller 5 has gone now, but there's still the big 5 right here. (20100329\_147)

                    - INTERVIEWER: What happened to the small 5? AB: Erm, kind of there if I look down. (20100329\_158-159).


                      When I look up slightly I am well aware of the 4 and the 6 over here. And big 5 is kind of blocking out the smaller one. When I shift my perspective down then I just have the 4 5 and 6 much more normal. (20100329\_161).

                      + -


                      It's hard though, because when I kind of focus on the looking down there, then I kind of want to, see what can I see of the bigger 5. I kind of feel my eyes being drawn up to it.  (20100320\_167)

                      - Even staring down there, I can still kind of seem to be able to see it if I focus my attention on it. (20100329\_169)

                    ...during the hypnosis session [conducted seperately from this study, investigating...?] where I was kind of floating above number line. I was kind of looking down at it. I was facing the direction that was being counted.  INTERVIEWER: That doesn't happen normally? AB: Not that today. INTERVIEWER: You mean you were floating along it from the top? AB: From above yeah. (20100408\_409-413)

                  ... see it hovering above a line that's sort of below me like that, sort of where I'm pointing, so looking at an angle away from me, going away from me and from left to right. (20100222\_5)

                  + -


                  So look back from 100... I'm kind of imagining behind the line. (20100229\_343)

                  - INTERVIEWER: So what did you have to do in order to change your perspective? AB: Erm, kind of rotate the image in front of me. So it's kind of over there and I kind of feel a 100 here and 101 there. (20100229\_344-345)
              + -


              'reference' for directing gaze/attention

              - + -


                AB is asked to locate the concurrent "four thousand BC" during in an exploration of his year time-line-form. AB initially reports that he "couldn't find it [4000BC] on this line  (20100320a\_298)". This may suggest that AB uses the line to direct his attention to a desired concurrent. The line may form a reference (with land mark bends at specific locations) thus the required concurrent can be located by interpolated. In practice however, AB found it difficult to draw the line and bends up to 4000BC with precision, perhaps as this is a location he is not familiar with. See drawing\_4000BC.

                - [4000BC] struggling to find it on the number line (20100320a\_294)


                  I couldn't find it [4000BC] on this line  (20100320a\_298)
      + -


      Cave

      - + -


        A series of letters is usually experienced in a 'cave' context, which is described as having a damp or muddy atmosphere. AB's head is positioned in one wall of the cave and concurrents are described as translucent or ghostlike rather than solid. Outside at the entrance of the cave is a naturalistic scene with a sky and clouds, with the entrance of the cave in the side of a mountain.

        - + -


          cave

          - + -


            The 'cave' is the usual context of AB's alphabet form. The cave is described with a distinct "dampness" (20100329\_441) or "muddy" atmosphere (20100408\_315), a dark background made of a natural (opposed to man made) substance and depth. Letter percepts within the cave are described as having "translucent" (20100408\_315) or "ghost-like" qualities (ibid.) rather than a solid form. AB's describes his head as bing in one wall of the cave allowing him to look into the depth or towards the entrance. When the entrance of the cave is explored, AB describes it as positioned in the side of a mountain (20100329\_459) with a naturalistic scene outside and a "sense of an expansive landscape" (20100329\_457), "sky and clouds" (20100329\_461) .

            - + -


              features

              - + -


                 A "cave" is reported as the context of the normal alphabet concurrents.   This context is described as "muddy" (20100329\_441) and with a feeling of "dampness" (20100408\_361); "Kind of wet and kind of cave like atmosphere. (20100329\_441)".   The walls of the cave are are "dark" providing "not much contrast for the letter (20100329\_426)" and also described as "hard and kind of damp and muddy (20100408\_315)". The cave itself has a spatial depth "going just off into a dark hole (20100329\_365)".

                - + -


                  atmosphere

                  - muddy (20100329\_ 439)


                    Kind of wet and kind of cave like atmosphere. (20100329\_441)


                    almost like dampness (20100408\_361)
                  + -


                  walls

                  - I feel that the walls are hard and kind of damp and muddy (20100408\_315)
                  + -


                  depth

                  - INTERVIEWER: So in terms of this kind of tunnel thing that's behind the letters, was that just a metaphor or did you really have a sense of something spatial being there? AB: Erm, yeah I had a sense of something being there. ... I don't know what's beyond where I can see. (20100329\_418-421)


                    going just off into a dark hole (20100329\_365)


                    this cave basically (20100329\_429).
                  + -


                  percept background

                  - browny kind of mess behind (20100329\_235)


                    a kind of dark brown kind of background (20100329\_357)


                    dark, not much contrast for the letter (20100329\_426)


                    seems natural (20100408\_337)
              + -


              percepts

              - + -


                 The alphabet concurrent are "just kind of standing up in the cave" (20100408\_311).  Unlike the number concurrents, the letters are described as having a "translucent" or "ghost-like" quality (20100408\_315); "a bit kind of like vague, like it could just be projection of the B or something (20100408\_311)."  The letters appear to be contained within the cave, against the wall or just outside (20100329\_433). The font size of the letters does not appear to be fixed, e.g. "The A is kind of shrinking to make way for the B (20100329\_433)".

                - Just kind of standing up in the cave. It seems a bit kind of like vague, like it could just be projection of the B or something. Well it is standing up. (20100408\_311). But the B is kind of translucent, ghostlike. (20100408\_315).


                  I got that for a moment here for the B. The A is kind of shrinking to make way for the B, just a bit unstable. Sometimes the B is kind of outside of the cave (20100329\_433).

                  + -


                  scalable

                  - The A is kind of shrinking to make way for the B, just a bit unstable (20100329\_433)


                    Hard to get a number in there. There's no room because the alphabet is in the way (20100408\_255)

                    + -


                    percepts scale to fit in cave

                    - INTERVIEWER:  In terms of the B, is it in the same space [as A] ? AB: Yeah I just got a feeling of presence that it would be on the wall of this cave basically. ... INTERVIEWER: But to get them in the same space. AB: ...The A is kind of shrinking to make way for the B, just a bit unstable. Sometimes the B is kind of outside of the cave. (20100329\_428-433).
              + -


              perspective

              - + -


                 AB reports that his "head is just in one wall [of the cave] (20100329\_451)". By physically moving his head AB is able to look in different directions within the cave. AB describes a "little bit of light coming from [behind] (20100329\_425)" which serves to illuminate the letter concurrents against the dark background. AB stands up and moves towards where he feel the light source is coming from and is able to describe the entrance to the cave.

                - My head is just in one wall. In one wall basically (20100329\_451)


                  Have to get up (20100329\_459) to go look outside/at the entrance


                  INTERVIEWER: ...Can you turn around and see where the light is coming from? AB: Yes, the entrance to the cave. (20100329\_444-445)


                  little bit of light coming from here (20100329\_425). From behind. (20100329\_437).
              + -


              entrance

              - + -


                 At the entrance and outside the cave AB describes a naturalistic scene with "whitish sort of bluish sort of light (20100329\_447)... like the sky (20100329\_449)". AB also describes "a sense of maybe a cliff face (20100329\_455)" at the entrance to the cave. He continues to describe "a sense of an expanse of landscape outside" (20100329\_457). When standing at the entrance of the cave looking out towards this landscape, AB says he is "in the middle of this big hole in the side of this mountain" (20100329\_459) with "green fields, way down... and just kind of sky and clouds" above (20100329\_461).

                - whitish sort of bluish sort of light. (20100329\_447). like the sky (20100329\_449)

                  + -


                  Then I'm kind of getting a sense of maybe a cliff face (20100329\_455)

                  - I'm getting a sense of an expanse of landscape outside (20100329\_457)


                    kind of just huge open space in front. I'm kind of in the middle of this big hole in the side of this mountain (20100329\_459). Kind of green fields, way down. And just kind of sky and clouds (20100329\_461)
      + -


      Tube

      - + -


        AB is directed to attempt to experience a number concurrent in the cave, in doing so the cave transforms into a 'tube' with the number concurrent at the end. The tube is darker and more cylindrical than the cave with solid, shinny walls like black plastic. The number concurrent is translucent or ghostlike, as is the letter in the cave, and the background is a solid red.

        - + -


          tube - number in a cave

          - + -


             AB is asked if he can "out a number in a cave" (20100408\_253) (the cave being the normal context for the AB's alphabet form). In doing so, the cave context transforms into a 'tube'.  The tube is a "cylindrical tunnel" (20100408\_261) which AB describes as "like I'm looking through a little tube actually. So I'm looking down a little telescope or something" (20100408\_303). At the end of the tube is the number concurrent (6) and the surrounding numbers in their respective positions. The concurrent itself is described with similarities to the letters in the cave, with "a kind of ghostlike character" (20100408\_339), as opposed to the numbers in the corridor or free space context which are 'solid'. The numbers surrounding the concurrent are against a red background with which distinct from the corridor. the tube red background is "like a wall. ... Kind of bright paint. Kind of very solid red. (20100408\_365-367)".  The walls of the tube are "pretty solid" (20100408\_359), and 'constructed' from a man-made substance (20100408\_355) which is a "black, shiny black [material]" (20100408\_273) like "hard plastic" (20100408\_363). The walls are not completely opaque, it "doesn't feel hard but, feels like you couldn't penetrate it very far" (20100408\_396). The tube is "darker and more cylindrical than the [cave]" (20100408\_265).

            - + -


              shape

              - So I kind of get like a cylindrical kind of tunnel. It's kind of black. There's kind of a 6 at the end of it (20100408\_261).


                it's darker and more cylindrical than the [cave] (20100408\_265).


                It's kind of black, shiny black [material] (20100408\_273)


                it's circular [all the way round including the floors and walls]. (20100408\_277)


                the material property of the tube is pretty strongly expressed. It's just a hard plastic kind of tube. It's just kind of there looking at the 6 and then all the other numbers are against a big red background (20100408\_363).
              + -


              number percepts

              - the 6 made of? It's like a kind of ghostlike character really (20100408\_339)


                So I kind of get like a cylindrical kind of tunnel. It's kind of black. There's kind of a 6 at the end of it (20100408\_261).


                I kind of feel like I'm looking through a little tube actually. So I'm looking down a little telescope or something at the 6 and then the rest of the numbers are against a red background (20100408\_303).
              + -


              background

              - red (20100408\_303)


                big red (20100408\_336)


                like a wall. ... Kind of bright paint. Kind of very solid red. (20100408\_365-367).
              + -


              wall

              - its wall is kind of a bit fuzzy (20100408\_343)


                Like a sort of hard boundary like the table, it's kind of slightly foggy (20100408\_345)


                the tube is this kind of manmade thing (20100408\_355)


                the walls of the tube are pretty solid (cf. The wall of the corridor is a bit kind of best defined as hard) (20100408\_359)


                doesn't feel hard but, feels like you couldn't penetrate it very far (20100408\_396).
      + -


      Felt presence

      - + -


        AB occasionally refers to the 'presence' of percept which he cannot direct see. We may speculatively group these occasions into 'weak' or unstable percepts and 'know' percepts, where AB knows a concurrent should be, based on the spatial relationships between them, but cannot directly see them.

        - + -


          presence

          - + -


            AB occasionally reports that he can sense the presence of a concurrent but not see it directly. These weak percepts are often described as 'vague', for example "these are bit more vague these ones. They are just kind of more like the feeling or the presence of the three there (20100415\_27)", "I have a sense of the five being somewhere over here. Can't see it very clear" (20100329\_65). "I've kind of like the sense of the six and the seven sort of somewhere around here. Not really quite sure where they are (20100329\_67)".

            The other weak percepts appear to have a spatial reference although they cannot be 'seen', for example, "I can sort of, kind of sense it kind of behind the wall. Because the 60 is right against the wall and the number line would be there (20100229\_366)" or "INTERVIEWER: ...but there's nothing where the four should be? AB: I felt the presence of a four, kind of over there ... But can't see it." (20100222\_242-245). In exploring the calendar from, AB is asked how he knows which month he is experiencing when the dates (1st-30th/31st) are essentially the same: "I just have a vague sense of its [the months] presence. So if I look at 22nd of March being here then the circle of the year is kind of like this. And I know we are in March just because of that position there. (20100320a\_334)." We might term this second group 'know' percepts, as AB knows they should be there via spatial referencing. These 'know' percepts may also include occasions where the percept is outside of AB's field of vision. For example, "I can sense a 3 behind me. INTERVIEWER: Can you describe that sense? AB: It's almost like I have an eye at the back of my head and I can see the 3 there" (20100329\_179-181); "INTERVIEWER: When you're turning away are you aware that the number stays there...?  AB: Yeah, if I think about it I am aware of its presence over there." (20100305\_151-152); "five's just gone out of my field of vision... I'm kind of aware of its presence." (20100222\_202)

            - + -


              weak percepts

              - I'm kind of getting a sense of maybe a cliff face (20100329\_455)


                Ive got a vague sense of kind of something in the womb but don't really know where it's located (20100320b\_110)


                I was aware of those other hands only vaguely aware but I didn't know exactly where [the minute hand] was (20100320b).


                I sense like white behind me (20100415\_264)


                I have a sense of the 5 being somewhere over here. Can't see it very clear, well it's coming into view (20100329\_65). I've kind of like the sense of the 6 and the 7 sort of somewhere around here. Not really quite sure where they are (20100329\_67)


                Yeah still 5, 6, 57. I sense a 54 here, but weaker this one though (20100305\_132)


                these are bit more vague these ones. They are just kind of more like the feeling or the presence of the 3 there (20100415\_27)
              + -


              prior experience "know" but not see

              - I just got a feeling of presence that it would be on the wall of this cave basically (20100329\_429).


                I'm just aware of the whole presence of the whole alphabet (20100329\_257).


                Can feel them all around me now, there's R S T behind me (20100329\_305)


                I got a sense [the number line for ages] might different [to that for normal numbers]. ... the higher numbers ... are less familiar [in my experience of] ... pure maths and ... only really used in the context ... of people's ages (20100320b\_90).


                I just have a vague sense of its presence. So if I look as 22nd of March being here then the circle of the year is kind of like this. And I know we are in March just because of that position there. (20100320a\_334).


                I can sort of, kind of sense it kind of behind the wall. Because the 60 is right against the wall and the number line would be there (20100229\_366)


                [the line] feels on the verge of turning big corner (20100229\_337).


                I kind of feel a 100 here and 101 there (20100229\_345)


                AS: When you're turning away are you aware that the number stays there...?  Yeah, if I think about it I am aware of its presence over there. (20100305\_151-152).


                AB: I can sense a 3 behind me. INTERVIEWER: Can you describe that sense? AB: It's almost like I have an eye at the back of my head and I can see the 3 there (20100329\_179-181).


                INTERVIEWER: So but there's nothing where the four should be? AB: I felt the presence of a four, kind of over there ... But can't see it.  
                (20100222\_242-245)

                + -


                white page

                - I sort of felt the presence of the four, and the six, kind of simultaneously, and four didn't quite come into view ... I sort of feel the presence of a four behind me, and then the six kind of appeared back, kind of above the [white page] (20100222\_233)

                five's just gone out of my field of vision... I'm kind of aware of its presence (20100222\_202).


                I can sort of feel a presence of a six, over here. Ahm, seems like it would be kind of, so it doesn't, doesn't fit in the corridor, it's kind of err hovering around the ceiling (20100222\_215)


                there's just kind all the way up there, I kind of feel the presence of a six but I can't really bring it forward ... But I sense a six, up there (20100222\_287).


                56 is here and I have a sort of vague sense of the 55 belonging here. Erm, but it's not that stable right now. (20100305\_23)
    + -


    Context transitions

    - + -


      There are dynamic transitions between the contexts. These context transitions generally occur automatically and in a predictable manner, although AB is able to trigger them of his own volition to a certain extent. We can present the following diachronic description of events:     
        
      Initially a new concurrent is presented on the white page. Surrounding the white page is a colour which is similar to the free space context. Directed by a change in AB's focus of attention to the area below the page, the white page melts from the bottom to the top. Descriptively, this melting is reported as "imagine the page was made out of ice and there was fire underneath it, and it's kind of melting gradually and kind of in a fairly homogeneous way" (20100329\_375). The shift in focus of attention from the page to the line causes a new concurrent to appear above the line. Once the new concurrent is stable, the line and corridor come into view.

      - + -


        => white page

        - + -


          AB is also able to reverse the process with some effort such that percepts which were in the corridor or against the line can be recreated with a white page. This is achieved by again directing his attention away from the corridor/line and to the top of where a white page percept would be. This process is described as though a "curtain comes down behind" the percept (20100305\_75-79). The effort involved in maintaining this new white page percept is demonstrated as AB feeling as though his eyes are "drawn back to the corridor" (20100229\_128) or as if the white page gets "stripped away" (20100222\_66).

          - + -


            corridor => white page

            - The wall kind of fades away into kind of whiteness if I kind of look up, and the number-line kind of disappears into that whiteness (20100222\_105). Erm, it’s more of kind of like an infinite whiteness (20100222\_107) than the white page...?


              as I soon as I bring my gaze, from say above the 56 then it's kind of like a white curtain comes down behind it (20100305\_79)

              + -


               so I was kind of looking over there towards you and there was, So it's almost like that’s kind of a new, so I get this kind of page but then I’m kind of feel my eyes drawn back to this corridor which I’m now in  (20100229\_128).

              - [AB had been requested to revisit this experience and he appears to have created a new percept on a white page.]
            + -


            line => white page

            - Yeah, it’s hard to bring it backwards, it’s hard to recreate the initial, th-the full white, background with the five. I can get little bits of it, maybe in the top left, but to totally bring back the whole number five... well now it’s kind of coming back. But erm, as soon as I bring it back it kind of gets stripped away again and the five is floating above the line. (20100222\_66)


               it’s hard to recreate the initial, th-the full white, background with the five. I can get little bits of it, maybe in the top left, but to totally bring back the whole number five... well now it’s kind of coming back. But erm, as soon as I bring it back it kind of gets stripped away again and the five is floating above the line (20100222\_66)


              look up (context trans back to white page) => the number-line kind of disappears into that whiteness (20100222\_105)


              as soon as I bring it (the white page) back it kind of gets stripped away again and the five is floating above the line. (20100222\_66)


              if I kind of look up, and the number-line kind of disappears into that whiteness (20100222\_105)
        + -


        white page =>

        - + -


          Looking down from the white page to the number line causes the white page to melt.      
            
          The change in focus of attention ("down") can be brought about by a movement of the eyes or simply by thinking about the relative position of the number on the line: " I can kind of let it melt away a bit from the bottom. Start think about where the fifty six goes in relation to the other numbers" (20100305\_21). In early interviews the melting process was described as: "sometimes the white pi.e of paper is only partially there. ... it sometimes seems like the bottom half sometimes goes first." (20100222\_46).   We later gain a more precise description when examining the alphabet form: "like if you imagine the page was made out of ice and there was fire underneath it, and it's kind of melting gradually and kind of in a fairly homogeneous way." (20100329\_375). The concurrents appear independent from the white page; the melting of the page does not alter  the concurrent: "It's almost like it melts away but whatever the letters are kind of painted in, stops that bit of page from melting away (20100329\_371)." "The letter is just left alone and [the] kind of melting goes through different bits (20100329\_377)". "Just leaving the letter alone. So it's kind of simultaneously on each of side of the letter, reaching the same level at about the same time (20100329\_381)."

          - + -


            melting

            - + -


              number

              - and then this [white page] kind of drifts, and then this kind of melt...like the the background, the white background kind of melts away (20100215\_150)


                Erm, it seemed to melt away more at the bottom first, and then kind of fizzled away at the top (20100215\_163)


                sometimes the white piece of paper is only partially there. And it’s partially... it sometimes seems like the bottom half sometimes goes first (20100222\_46)


                It’s kind of melting away from the bottom upwards. There’s still a little bit of it at the top (20100305\_25)


                staying more on a white background 56 perhaps. ... Little bit more stubborn in the top left corner, it kind of wants to be behind a white background. But I can kind of make it pretty much go away (20100305\_75).


                The white page has a tendency to melt away (20100408\_97)


                white page melts away, ... , number kind of drops down and the line appears. Not quite sure at what stage the line appears in relation to the 60. To the page melting away and the numbers dropping a bit (20100229\_311)


                so the line seems to appear after the thing melting away, after the page melting away. Then the number stays in pretty much the same place (20100229\_313)


                I can kind of let it melt away a bit from the bottom. Start think about where the 56 goes in relation to the other numbers (20100305\_21)


                if I look down at the number line and then back up at the 56. It kind of as I look up the white background kind of sort of lifts (20100305\_77)
              + -


              alphabet

              - Then I’ve got a sense of that lifting up and revealing a kind of background again (20100329\_227)


                it's kind of melting away (20100329\_229) [from the bottom upwards]


                I can strip it away. ... it's kind of quite forced. It's almost like a more violently tearing it. ... It doesn’t really quite go away fully. Now they are in a kind of dark brown kind of background. They are easier to see against the white. (20100329\_353-357).


                It's almost like it melts away but whatever the letters are kind of painted in, stops that bit of page from melting away (20100329\_371). The letter is just left alone and it kind of melting goes through different bits (20100329\_377). just leaving the letter alone. So it's kind of simultaneously on each of side of the letter, reaching the same level at about the same time (20100329\_381).


                like if you imagine the page was made out of ice and there was fire underneath it, and it's kind of melting gradually and kind of in a fairly homogeneous way. It just melts (20100329\_375)


                INTERVIEWER: So, can you let the page melt from the bottom. AB: Yeah. There’s the rest of the alphabet around it. It's kind of against a dark sort of purplish background. (20100415\_243-244).

              [letters] INTERVIEWER: So when that happens [melt] and the background is revealed. ...INTERVIEWER: And at this stage [white page, before the melt]. ... There’s no background showing around the sides? AB: Yeah, it's like the same type of background that is revealed. AB: Kind of like a purple layer here, a green layer here and some black. INTERVIEWER: So if you let the page melt away, it just reveals more of what was already around the page. (20100329\_386-398)


              A 56 is kind of up here on the white background. White background is kind of melting away again from the bottom. So I’m going to look down towards the bottom of the 5 and 6 and then it's against that kind of greeny orangey background. Little bit more stubborn in the top left corner, it kind of wants to be behind a white background. But I can kind of make it pretty much go away [by looking at the number line]. INTERVIEWER: How do you do that? AB: <pauses> Just like I’m with it I guess. I think if I look down at the number line and then back up at the 56. It kind of as I look up the white background kind of sort of lifts[melts]. INTERVIEWER: So does the direction of your gaze, if I understand it correctly, if you trace it up from the bottom where the number line is or supposed to be, up towards the 56 and then trace it on 56. As your gaze progresses the white background disappears. AB: Yeah. And as I soon as I bring my gaze, from say above the 56 then it's kind of like a white curtain comes down behind it. (20100305\_75-79)


              INTERVIEWER: So the white page is associated with the x z axis, but when it starts falling down the white page melts away and that’s when the number falls into the x y plane. AB: So I got 60. Yeah, so the line seems to appear after the thing melting away, after the page melting away. Then the number stays in pretty much the same place, maybe drops down a little bit after the line’s appeared. INTERVIEWER: So the number doesn’t actually fall down into the other plane. Why did you think it was falling down before? AB: It must have fallen down at some point because it's starts off up here and then it kind of sinks more there. It's a bit unstable, it kind of yeah doesn’t really, takes a while to really settle into one place. It seems to depend on where I focus my attention where it ends up.  (20100229\_312-315)
            + -


            white page => corridor

            - So the white... So... so the five on a white background appears first, and then the white kind of melts away. And then the five is kind of floating in this room. (20100322\_35)


              The sequence in the beginning is displayed on a white page and then somewhere it's kind of leaving that and not really sure how. For a bit it's against that kind of erm greenish, reddish orangey background (20100229\_108)


              So this time the white page is kind of melting away and I am kind of against that kind of coloured background again... the other numbers. So then they kind of for a bit appear on the sort of floor. (20100229\_112)


              and then it drops down here in the corridor and then there’s a line going off that direction (20100229\_303)


              So it's on the white thing but then that goes away and then it's kind of now on the corridor, further down the corridor from this one (20100229\_358).


              so the five on a white background appears first, and then the white kind of melts away. And then the five is kind of floating in this room [corridor] (20100222\_35)


              White background is kind of melting away again from the bottom. So I’m going to look down towards the bottom of the 5 and 6 and then it's against that kind of greeny orangey background (20100305\_75).
        + -


        => line

        - + -


          AB looks down to bring the number into the correct position on the line (20100329\_108-109; 20100305\_75-79). Looking down also causes a new percept to be generated in the correct position on the line (20100215\_122; 20100215\_124; 20100215\_151; 20100229\_311). The position the concurrent drops into is dependant on where attention is focused (20100229\_312-315).

          - + -


            white page => line

            - So it seems like the first thing is, is the big number five and then it kind of... moves into position, kind of floating above the line. (20100215\_122) ... I think the line appears a little bit after, so the, the digit gets constructed first, and then, and then... and then the line comes into view (20100215\_124)


              Ahm, starts moves, moves downwards, and then at some point a line appears and the number’s floating, erm kind of above the line. The line is coming from the lower left of the field and going towards the upper right. And I’m... I’m a... sort of, from where I’m centred, I’m looking, I’m looking sort of, it’s kind of ahead of, ahead and down from where I’m there, from where my ego’s located. (20100215\_151).


              I’m looking erm, directly at the page. The page is horizontal in front of me but then the number line is kind of down. (20100215\_153).


              So I’m not quite sure of the, the order of things but erm... Sort of a similar thing sort of... I, I can definitely see it being kind of located below where the page was. (20100215\_155)


              INTERVIEWER: When does the number line appear? AB: Just after the, err, I think just after the white, sort of paper thing has melted away. (20100222\_26-47)


              So five against a white background, the white background kind of melts away, and then it’s floating above a black line, which is going from bottom left to the top right, kind of looking at the... <slowly> The line is looking down from where I’m... it’s kind of along the floor. <faster> And then there’s sort of a wall behind, this colour that I can’t quite make out exactly what colour it is. <slowly> (20100222\_49).


              So I see these two numbers on a... on a white page. ... Eventually they appear against a kind of a number line, (20100222\_90-91)


              INTERVIEWER: When does the number line appear? AB: ... I think just after the white, sort of paper thing has melted away (20100222\_46-47)


              So five against a white background, the white background kind of melts away, and then it’s floating above a black line (20100222\_49).


              as soon as I look at the number line, I’m not seeing the 6 anymore. I’m kind of aware of its presence. But it's not the same 6 dropping. It's like a new 6 gets produced there (20100329\_111)

              + -


              the line seems to appear after the ... page melting away. Then the number stays in pretty much the same place, maybe drops down a little bit after the line disappeared (20100229\_313)

              - the line disappears?

              I think the line appears a little bit after, so the, the digit gets constructed first, and then, and then... and then the line comes into view (20100215\_124)


              So it seems like the first thing is, is the big number five and then it kind of... moves into position, kind of floating above the line. (20100215\_122)
        + -


        => corridor

        - + -


          The line and corridor are created after the concurrent has stabilised in position (20100215\_124; 20100222\_46-47;  20100222\_49; 20100229\_313; ; 20100322\_35)

          - + -


            => corridor

            - Well I’ve just got this vague sense of kind of a metallic five, at first it wasn’t in the corridor, and then when I thought about it some more it ended up becoming grounded in the floor (20100222\_317)

              + -


              No [not on the white page], they seem to just be transported straightaway into the corridor this time. (20100305\_15)

              - interesting that thiese are 'transported'

              I wasn’t aware of the usual process of seeing it on the white page. It was just kind of still right there on the corridor (20100229\_349)


              So I’m going to look down towards the bottom of the 5 and 6 and then it's against that kind of greeny orangey background.  (20100305\_75)
            + -


            free space => corridor

            - INTERVIEWER: [a 6] appeared somewhere up here in the [free, purlpe b/g] space between us. And then you get a sense of the numbers next to it, in this case 5 7 and they are part of the number line [in the corridor] ... you can drop down the 6 into this sequence. <AB agreeing throughout> ... How can you make the two connect [] the free space 6 and the corridor 5, 7? Is there anything that you notice? AB: By looking at the number line (20100329\_108-109). But it's not the same 6 dropping. It's like a new 6 gets produced there.(20100329\_111)
            + -


            outside corridor => inside corridor

            - INTERVIEWER: Okay, so if you will it to come down, what happens? AB: Kind of crept down the wall, and there’s like a small number six against the wall, and there’s a line, joining the five to the six. Well it’s not touching the five or the six but err... (20100222\_218-219).


              Ahm, it was pretty quick, I didn’t see any movement, it just kind of reappeared down at the bottom.  (20100222\_231)
  + -


  Physical-mental room

  - + -


    AB reports that his synaesthetic percepts exist in a 'mental' room, rather than the physical room. This mental room is described as existing in another 'dimension', in a copy of the physical room. AB retains an awareness of the physical room when experiencing his synaesthetic percepts but describes this awareness as "out of focus" like normal peripheral vision (20100320a\_114). Importantly, the physical room appears to hold more salience than the mental room such that an object in the physical room may interfere with AB's ability to perceive a concurrent. This extends into the visual presentation of inducers, where AB reports that in order to experience his synaesthetic percept he has to look away from the "distracting" physical object on which the inducer is presented (20100408\_13), and that this visual induction is "very different" from auditory induction (20100408\_65-67).

    - + -


      Concurrent 'exist' within a 'mental room'

      - + -


        AB describes his synaesthetic percepts as existing in a 'mental room' as opposed to the physical room which contains normal (non-synaesthetic) percepts. The percepts in the mental room "superimposed" on the physical room (20100408\_137) and are said to have the same physical location as items in the mental room, but the mental and physical exist in two "different dimensions" (20100408\_163; \_165; \_189).   
          
        AB reports that he can switch his attention between the physical and mental rooms by defocusing his eyes and "looking into space" (20100408\_33) or switching his focus of attention to the mental room (20100320a\_108). AB retains an awareness of the physical room whilst looking at the synaesthetic percepts (20100320a\_112) and the physical room appears "out of focus" (20100320a\_114) similar to a normal experience of an item in peripheral vision (.ibid).

        - Erm, well I can just alternate my attention between the physical room and the mental room I've got (20100320a\_108)


          INTERVIEWER: So if you focus your attention on the green mist or wall, your visual awareness of the room fades out, the physical room? AB: Yeah. It never leaves me completely, the physical room (20100320a\_111-112).


          I guess all of the physical room is like erm, like what's in my periphery normally. So the focus of my eyes is somewhere about here, because there's nothing in the physical room here. The whole physical room is out of focus (20100320a\_114).


          it's just kind of misty. I'm sort of seeing the room behind. But then I sort of feel the letters are in a different kind of space as well. It's just of, I don't know, browny kind of mess behind (20100329\_235).


          I think my eyes are kind of defocused, just looking in space. So whatever is behind, first physical thing in the direction I am focusing my eyes that aren't focused (20100408\_33)


          It's kind of superimposed (20100408\_137)


          So now I just see my finger as being in the physical space and the 4 as being in the mental one (20100408\_155). It's almost like I'm peering into another dimension or something. And the finger is in front of the 4 (20100408\_163)


          It's like if this room is at 0 in one of the extra dimensions and then the 4 is at 1 or something (20100408\_165). So it's like the 4 is in a different dimension. So it's 3D coordinates are in the same position as the pen (20100408\_189).


          it's almost like you have this whole room and then there's like a copy of this whole room (20100408\_191). And then the pen stays in this room. But all the numbers and things are in the copy of this room. (20100408\_193)
      + -


       Interference from the physical room

      - + -


        The physical room appears to hold generally more salience than the mental room such that objects in the physical room obscure synaesthetic percepts or make them weak and vague: "...if there's an object in this [physical] room, in the same place as one of the numbers are in the other [mental] room, then I see the object rather than the number" (20100408\_195). AB displays a tendency for concurrents to be located in areas of space where there is little interference form physical objects.   
          
        This effect of physical interference extends into the visual presentation of an inducer, where AB reports having to look away from the "distracting pi.e of paper" (20100408\_13) in order to experience his alphabet form: "it's almost like the actual physical H can't be part of the mental alphabet" (2100408\_23). Similarly when AB is shown a number inducer he reports difficulty in perceiving a stable concurrent when viewing the number written on a page and states that the experience is "very different from when you are telling me to think of the number 4" (20100408\_65-67).   
          
        This interaction between the 'physical' and 'mental' room may be of relevance in experimental studies of spatial-form synaesthesia where inducers are presented visually.

        - I don't see them relating to your hand. (20100305\_122)


          ...in relation to the table because when I'm closer to the table, seemed like they were nearer. Because now they are a bit further than arms reach (20100305\_191).

          + -


          Physical object obscures mental percept

          - + -


            It appears that for AB there is a relationship between the presence of objects in the physical room and the default 'placement' of synaesthetic concurrents, such that the presence of physical objects obscure the mental percepts and the concurrents are weak or unstable. AB displays a tendency to place or locate the concurrents in areas where there is little physical interference.  Examples of interference from the physical room are given below:

            "The floor in this room is obscuring the floor in that space [the mental room]. I can't really see down. (20100415\_278)".

            "...that wall's kind of interfering with it. (20100415\_258)."

            "Can't see, the ceiling is kind of interfering with it [C percept] (20100415\_245)"

            "...it didn't really work as it [the floor of the physical room] was interfering with it [the percept]. (20100329\_93)

            "I feel the 6 may be a bit blocked by the table now. Not sure exactly where it is. (20100329\_63)

            "At the moment there's more the table that is present.  (20100329\_29)

            Importantly, for this subject the physical presentation of a letter, i.e. a letter drawn on a page and presented to him, inhibited the induction of the alphabet concurrents: "[*AB was shown a letter H draw on a notepad*. INTERVIEWER: What do you see?] I see the letter H on the page. INTERVIEWER: So what do you need to do in order to see the alphabet line? AB: Erm, have to look away from the image. I can see them here (20100408\_3-5).

            So I think my eyes moved didn't they ... I was kind of immediately aware of the H, just kind of where I put my gaze. Away from the distracting pi.e of paper (20100408\_13). "...when I was focused on the paper there was nothing else except what was physically there. (20100408\_15)" "...it's almost like the actual physical H can't be part of the mental alphabet.  (20100408\_23)." "INTERVIEWER: So how did you know that you had look away from the page for the alphabet to appear? AB: Just because it wasn't appearing when I was looking at it. <laughter> I opened the page for a few seconds and there's nothing happening, so I though wait a minute shouldn't an alphabet appear now and I kind of averted my eyes and then there it was. <laughter> (20100408\_20-21)". The same physical interference is encountered when AB is shown a number written on a page:  "I can either pay attention to the notebook or the three, so don't really focus on one or the other (20100415\_19)." "So I see the four [drawn in a notebook] and then there's nothing really stable coming into view. It's like the number line doesn't know which direction to go in. It's like there are various things down on the table and something over here ... Nothing very precise came in. Just very different from when you are telling me to think of the number four (20100408\_65-67)".

            If this effect of physical interference is found in other subjects, it might be of particular relevance in experimental studies of spatial-form synaesthesia where inducers often presented visually; "And so if there's an object in this room, in the same place as one of the numbers are in the other room, then I see the object rather than the number. (20100408\_195)."

            - the floor in this room is obscuring the floor in that space. I can't really see down. (20100415\_278)


              that wall's kind of interfering with it. Over there, I can kind of see it continuing on, in that way it's almost like we are in a sort of spherical thing. (20100415\_258)


              Can't see, the ceiling is kind of interfering with it [C percept] (20100415\_245)


              And so if there's an object in this room, in the same place as one of the numbers are in the other room, then I see the object rather than the number. (20100408\_195)


              it's harder to see the 4 if I do that because my finger is in the way. ... So it's just kind of like the other ones where I haven't been able to see the numbers because the table is in the way. (20100408\_157-159)


              It's kind of superimposed... I'm aware of the 4 being there, but I can't really keep focused on it. INTERVIEWER: ...so when you move, you look at the physical room but you need to focus your attention again to something that lay on top of it. (20100408\_137-138)


              it's almost like the actual physical H can't be part of the mental alphabet.  (20100408\_23)


              when I was focused on the paper there was nothing else except what was physically there. (20100408\_15)


              it didn't really work as it [the floor of the physical room] was interfering with it [the percept]. (20100329\_93)


              I feel the 6 may be a bit blocked by the table now. Not sure exactly where it is. (20100329\_63)


              At the moment there's more the table that is present.  (20100329\_29)

              + -


              Pen is slightly behind the 4 at the moment. It's kind of going through the 4. ... So it's kind of obscuring some of the 4, but I can kind of see some of the 4 underneath the pen. ... Yeah so it's almost like the 4 is behind the [pen]. ... Yeah, a bit like in another dimension. (20100408\_179-185)

              - It [the pen] just blocks a bit of the 4. I don't see a bit of the 4. ... Yes, if it's behind it's ok, because the 4 is just here. ... Now it's kind of got a gap. It's kind of the pen is blocking it, a strip in the middle of the 4. (20100408\_199-209)

              I see the letter H on the page. INTERVIEWER: So what do you need to do in order to see the alphabet line? AB: Erm, have to look away from the image. I can see them here (20100408\_3-5).


              So I think my eyes moved didn't they and then an alphabet appeared here. Not sure quite what order. I was kind of immediately aware of the H, just kind of where I put my gaze. Away from the distracting pi.e of paper (20100408\_13).

              + -


              if there's an object in this room, in the same place as one of the numbers are in the other room, then I see the object rather than the number (20100408\_195).

              - Can look at the pen or the 6 (20100408\_235).


                I can either pay attention to the notebook or the 3, so don't really focus on one or the other (20100415\_19)


                It's just like another world that I can focus my attention on or I can bring my attention back to the room (20100415\_55)

              It's hard because when I walk, I find it hard to focus my attention on the number so I tend to kind of focus on the physical room because the number kind of disappears a bit (20100408\_135)


              I'm more in this room now. I'm more in the physical world...than... any... the mental world (20100222\_162)


              INTERVIEWER: So how did you know that you had look away from the page for the alphabet to appear? AB: Just because it wasn't appearing when I was looking at it. <laughter> I opened the page for a few seconds and there's nothing happening, so I though wait a minute shouldn't an alphabet appear now and I kind of averted my eyes and then there it was. <laughter> (20100408\_20-21)


              So I see the 4 [drawn in Interviewer's notebook] and then there's nothing really stable coming into view. It's like the number line doesn't know which direction to go in. It's like there are various things down on the table and something over here ... Nothing very precise came in. Just very different from when you are telling me to think of the number 4. (20100408\_65-67)

              + -


              INTERVIEWER: have a look at this page here. What does that do to you? AB: So initially it's just, I can see a number 4 and then a couple of seconds later there's a 3 there and a 5 there [in the correct positions in relation to the visual 4] (20100415\_2-3).

              - INTERVIEWER: What happens to the other two now that I've moved the page away? AB: Now I've got a 3 4 5. INTERVIEWER: Ok the 4 has come up? AB: Yeah (20100415\_8-11).
          + -


          Strong/stubborn mental percept

          - + -


            There are a number of examples where AB reports limited awareness of the physical room. In these cases the mental percept is strong and generally occurs in a physical space which does not contain any specific objects: "Kind of metallic one appearing briefly over there in the middle of the space, and that one won't really change [pattern]. (20100415\_196)." "But sometimes I don't see the table at all and just only aware of them as being inside this corridor (20100329\_25)." "It's more like the floor of the corridor is lined up with the table top (20100329\_27). "[noting there is nothin present on the table]  I don't see the table in that at all (20100329\_43)." "[The concurrent] seems independent of the table. It's like the table just isn't there when I am paying attention only on the fifty five (20100305\_95)."

            - Kind of metallic one appearing briefly over there in the middle of the space, and that one won't really change. (20100415\_196)


              INTERVIEWER: So if you look at the 6, keep focus on the 6, what happens if I move the pen in there? It does interfere or? AB: No. I can just see the 6. INTERVIEWER: So there seems to be a difference between these two kinds of spaces. (20100408\_236-238)


              I don't see the table in that at all (20100329\_43)


               But sometimes I don't see the table at all and just only aware of them as being inside this corridor (20100329\_25)


              I'm kind of seeing them on the table now. But sometimes I don't see the table at all and just only aware of them as being inside this corridor (20100329\_25). It's more like the floor of the corridor is lined up with the table top (20100329\_27)


              AB: I don't see any details, just kind of pure kind of background. INTERVIEWER: But it doesn't have anything of the physical room or presence? AB: No (20100320a\_100-102).


              Seems independent of the table. It's like the table just isn't there when I am paying attention only on the 55 (20100305\_95)
          + -


          Avoiding physical interference

          - + -


            The following are examples of AB's preferential 'placement' of concurrents to avoid physical interference:

            "The C is just kind of in the room at the moment. Probably need to move it a bit more on an empty space" (20100415\_238).  Put the seven and the eight in the corridor? ... Erm, seems hard with the corridor down there [on the floor of the room]. I can have the corridor up here. Got a seven here and eight here. ... Bit of difficulty with the seven. It's kind of like the corridor wants to enlarge and have the seven over here. So that table isn't in the way" (20100329\_103-107). "...it seems like it's easier to appear on a surface. But then I kind of want them to appear in a space. I can kind of engineer that quite straightforwardly" (20100329\_89). "If my eyes are kind of focused on some point in space in which there is not physical object then it's easier for the [concurrent to appear]..." (20100408\_31).

            - Just because it wasn't appearing when I was looking at it. I opened the page for a few seconds and there's nothing happening, so I though wait a minute shouldn't an alphabet appear now and I kind of averted my eyes and then there it was (20100408\_21).


              It's just because I'm focusing on the page, I can't see anything there and so that's why. Whereas if my eyes are kind of focus on some point in space in which there is not physical object then it's easier for the... (20100408\_31). I think my eyes are kind of defocused, just looking in space (20100408\_33).


              INTERVIEWER: When you first saw it, you thought it was a B because of the line coming through the page. Was that enough to trigger your awareness of the B in your alphabet form? AB: Erm, I kind of become aware of it over here. INTERVIEWER: But at the time you were just focused on the page. AB: Yeah, it came later so it's not immediate. ... seems to be appearing to the right of the page (20100404\_56-63).


              I'm kind of seeing them on the table now. But sometimes I don't see the table at all and just only aware of them as being inside this corridor (20100329\_25). It's more like the floor of the corridor is lined up with the table top (20100329\_27)


              I guess all of the physical room is like erm, like what's in my periphery normally. So the focus of my eyes is somewhere about here, because there's nothing in the physical room here. The whole physical room is out of focus (20100320a\_114).


              INTERVIEWER: So how did you know that you had look away from the page for the alphabet to appear? AB: Just because it wasn't appearing when I was looking at it. <laughter> I opened the page for a few seconds and there's nothing happening, so I though wait a minute shouldn't an alphabet appear now and I kind of averted my eyes and then there it was. <laughter> (20100408\_20-21)


              The C is just kind of in the room at the moment. Probably need to move it a bit more on an empty space. (20100415\_238)


              It's just because I'm focusing on the page, I can't see anything there and so that's why. Whereas if my eyes are kind of focus on some point in space in which there is not physical object then it's easier (20100408\_31)


              Put the 7 and the 8 in the corridor? ... Erm, seems hard with the corridor down there [on the floor of the room]. I can have the corridor up here. Got a 7 here and 8 here. ... Bit of difficulty with the 7. It's kind of like the corridor wants to enlarge and have the 7 over here. So that table isn't in the way. (20100329\_103-107)


              it seems like it's easier to appear on a surface. But then I kind of want them to appear in a space. I can kind of engineer that quite straightforwardly. (20100329\_89)


              the floor of the corridor is lined up with the table top. (20100329\_27)


              Just kind of had the intention of seeing some numbers in the corridor. ... The wall of the corridor is a bit fuzzy, so it's not like a hard surface like the table, it's kind of like. 6 is right there, so if you put anything behind the 6 it would go into the fog and you wouldn't be able to see it. (20100408\_243-349)


              When I looked away there was immediately, an alphabet appeared at least as far as H. I was only aware from A to H, I wasn't aware of anything beyond H (20100408\_17).
  + -


  Form descriptions

  - + -


    AB's synaesthetic forms examined in the course of these interviews include a spatial number form, including experiences of mathematical concepts such as pi, a spatial alphabet form, a spatial form for age and seperate spatial calendar forms for years, months of a single year and time (clock faces). All except the clocks contain a line and all include aspects which enable AB to precisely locate specific items by their spatial relationship to one-another. In those forms which contain numbers, AB describes a visual experience of isolated digits which are ordinarily black in an arial-like type face however on occasion (particularly with the number five), the digits appear with textures, patterns or lighting effects.  
      
    Of particular note are the positioning of a comma thousands separator in large numbers to denote a distinction between the normal number form ('one thousand, nine hundred and fifty five') and the year form (nineteen fifty five). In exploration of mathematical concepts AB describes the visual experience of irrational numbers to multiple decimal places, graphical and numerical representations of equations and a 'black hole' which may be conceptually identified with infinity.

    - + -


      number

      - + -


        Number concurrents generally appear as black Arabic symbols in an arial-like type face. Certain numbers (notably five and fifty six) are described as having textures, such as a metallic shine (20100222\_247) or  "velvety texture" (20100222\_321), and also patterning such as "cross hatching" (20100408\_67) or pot marks "as if the ink has come away in lots of places" (20100320a\_246). The texture or patterning may move around and change although the number itself remains static.

        Both fractions and decimals are viewed in the correct position relative to their surrounding numbers. different notations of fractions can be generated on request (such as 1.5, 3/2, 6/4). Different notation appear generally as though superimposed on top of each other. Large number (in the thousands) appear with the comma thousands separator.

        - + -


          general feature description

          - + -


            Numbers generally appear in black Arabic symbols in an arial-like font. The number have a spatial relationship to each other in accordance with AB's number line schematic. The correct numbers in sequence can be seen on either side of the induced number.

            - I see... I see the, the symbols of the numbers, in the Arabic ordinary symbols, ahm, I see them quite big, and black in colour against a white background. Ahm, the number one... is, ahm, sort of, sort of appearing in the middle of the page, and then number two appears up and to the right of number one. And then number three appears up and to the right of number two. So it's sort of in a straight line evenly spaced. <slow> (20100215\_11),


              So now I'm kind of more looking down on the page <louder>... and the angle is slightly different... it's kind of... ahm... it's like the clock has turned a bit more, it's kind of going off more at, ahm, so if the number one is, ahm, so I'm sort of looking, yeah I'm sort of looking down maybe at about an angle of 60 degrees, and number one is, yes, the right way up. And then the number line is going off at an angle of, err, probably the direction of two o'clock on a clock face. Ahm, and, they're in a straight line... and evenly spaced up to ten. Ahm, I can sort of see a line going off, ahm, like the continuation of the number line going off. <slow> (20100215\_26).


              So, well that I see just as a line really. When you tell me to just count to ten I can see the actual digits as far as ten but then I can sort of... I guess as I'm.., I guess as I'm reciting it in my head the number that I'm thinking of appears as a sort of clear digit, ahm, then I can, well I sort of see the number line... well, just the number, just the sort of... well you said the number ten, ahm, I guess that, that appears with a number line behind it and a number line in front of it. And there's a clear right hand, there's a bend at the number ten and then the continuation of the number line beyond ten is going to go off, ahm in a different direction. (20100215\_28).


              Yeah well it gets, yeah it's a bit, a bit blurry. When you said the number ten, and I was counting to number ten, I never saw the number eleven at any stage, but I did see a nine going off in a different direction. I never saw the digits for eleven. (20100215\_31).


              Yeah, this is a two dimensional corner. So the line coming in... sort of from the left... It's not really stabilised, the angle of the turn isn't stabilising. But if you're approaching twenty, from the teens, it's a left turn, at number... at number twenty... I see, i see again a kind of line with just the number twenty in the middle of... just written in big. So that'll occupy more space than the width of the line. (20100215\_41).


              I guess between seven and eight o'clock sort of stabilising a bit. Now I was going to say between seven and nine o'clock, I don't think it is, it's not as sharp as a right angle, it's a bit, it's a, it's like a... yes the number of degrees you're turning is, err, somewhere... somewhere between 30 and 60 degrees probably. <slowly> (20100215\_48).


              [in realtive position with surrounding numbers] the 55 is much smaller, black, totally black (20100305\_33)


              the symbols of the numbers, in the Arabic ordinary symbols, ahm, I see them quite big, and black in colour (02100215\_11)


              it's almost like a projection of the 4 or something (20100408\_153)


              Erm, now what's the 6 made of? It's like a kind of ghostlike character really (20100408\_339)
          + -


          patterning

          - + -


            Number concurrents are occasionally reported with a patterning on them. This patterning appears to be specifically associated with the numbers five and fifty six, for example five was reported with metallic qualities, a " dark, shiny sort of metallic sort of lustre to it" (20100222\_247). There is a consistent description of a darker outline to the number five (grey or black) but the internal colouration is not stable; on occisions it is described as "bluey purple" (20100222\_345) and on another occasion with green (20100305\_146). There are descriptions of variable textures, such as "shimmering" effect (20100222\_255) where a "whiteness fills the bottom bit of the number... goes up to the top then goes back [down to the bottom]" (20100229\_460), a "velvety texture" (20100222\_321), "cross hatching" (20100408\_67), "with a white ribbon on isolated sections" (20100222\_251), "white stripes and spots" (20100229\_394) which appear as though "someone has punched a hole out of the top corner" (20100229\_298) or like "pot marks, as though the ink has come away in lots of places" (20100320a\_246) or "like a poor quality photocopy" (20100415\_37). This patterning appears to move around on the digit whilst the digit is itself static (20100229\_424-426), and different areas of the digit can be patterned differently, for example in a later description of fifty six "one bit of it is made out of feathers...the other bit is kind of black and velvety" (2010030520a\_153-155). The patterns may also 'spill' onto the white page (20100229\_436), \*\*see fig drawing\_20100229\_56pattern. There is also a suggestion that certain numbers (e.g.  fifty six) may form a 'locus' of the patterning such that surrounding numbers to become patterned when they are seen in the presence of the locus. 

            - + -


              5

              - + -


                metalic

                - I thought perhaps if you shone a really bright light on it then it might have a dark sort of, dark, shiny sort of metallic sort of lustre to it (20100222\_247)


                  it's kind of metallic (2010022\_269)


                  So there's kind of like a grey outline, and then kind of on the face of the number kind of bluey purpley kind of colour (20100222\_345)


                  Sometimes it goes a bit black as well, in the middle, so, it gets black in the outside (20100222\_347)

                The five itself isn't very stable ... it seems like there's always kind of ... black outline of the number five, and ... the shading can be varied, and even have patterns on it at times (20100222\_14)


                there's kind like a white sort of ribbon just on one little bit of it ... it's just like it's lighting up a little bit of [the] ... curvy bit (20100222\_251)


                I sort of look at the top of it and it's kind of the black, and I look at the bottom and it kind ... shimmers a bit and sort of... the whole image sort of moves around a bit, sometimes the face, the curvy bit is kind of going the wrong way and it's kind of like this (20100222\_255).


                a black five over here, that's more kind of velvety ... with some sort of white light shining on ... there's kind of this white shimmering light on that bottom half (20100222\_321).


                The black at the top is kind of much darker, so there's a little bit of white light on the top now, because we've introduced this light up here. ... So it's kind of shining, kind of getting a bit of light on top (20100222\_322).


                [free space] It's got kind of patterning on it. It's got kind of cross hatching (20100408\_97)
              + -


              4

              - it's kind of metallic, but ahm it seems softer than the five (20100222\_301)
              + -


              56

              - + -


                56 seems slightly decorated, like there's kind of stripes in various places and like a white spot in the middle of it erm (20100229\_394)

                - a few stripings around the middle of the curvy part of the 5 (20100305\_29)

                just kind of black stripes and just kind of white underneath (20100229\_296)

                + -


                nothing that stays really too stable ... If I really try and focus ... I see a little white so it's kind of mainly black top bit of it. ... I am seeing a little white circles so the 5 this is. The white circle missing from top corner as though someone has punched a hole out of it from the top corner (20100229\_298)

                - So it's like a white hole in the top left corner still. And it's like someone has punched a hole out of the 5. Well it's kind of like a white spot really (20100305\_27)

                drawing\_20100229\_56pattern


                a black splodge around two o'clock around the circular bit of the 6 (20100229\_432). Yeah, the black splodge is a bit of a mess really (20100229\_434). it seems to be spilling onto the page around the 6, so it's kind of destroying the smoothness of the curve there (20100229\_436)

                + -


                stability

                - + -


                  Have to really kind of try and focus on one bit of the number or another (20100229\_422)

                  - I lift and zoom in and there's just a few stripes there and a few stripes down there (20100229\_428)

                  Not really stabilising very well, I mean sometimes I see bits of like stripes and it's hard to draw precisely (20100229\_412)


                  all kinds of marking, but they are kind of not really stable (20100229\_404)


                  Just seems to just be moving around. ... Not the digit itself but the markings on it (20100229\_424-426).


                  INTERVIEWER: Is it shifting around or is it becoming more stable? AB: Becoming more stable (20100229\_433-434)
                + -


                locus

                - the 56 is the locus of this, it's sort of where, it's just got more of it than the others (20100229\_464)


                  56 looks kind of more patterned than the others. Occasionally you get the slight stripe on like 57 but nothing as detailed on the 56 (20100229\_460)

                  + -


                  So I can get kind of get white lines going across the numbers so it's kind of black, mainly black but sometimes you get whiteness fills the bottom bit of the number, kind of goes up to the top and then goes black again. So a shimmering effect (20100229\_466).

                  - it just happened once now with the 59 (20100229\_468)

                  So there were little bits of sort of patterns and greens on it [55]. So it's mainly black, just around the edges there's bits of patterns and greens (20100305\_146)


                  The 9 [of nineteen fifty six] has markings on it as well (20100320a\_242). I noticed markings on the 9 right away. So it's black and white and there's kind of like white sort of markings on it (20100320a\_244).  It's almost kind of like pot mark, likes the ink has come away in lots of places (20100320a\_246).

                So I saw that kind of on a white background. And again it's got sort of patterning on it (20100305\_17)

                + -


                less pattern when surrounded by other numbers or on the line

                - + -


                  when it's down there [with the surrounding numbers 54, 57, but no line] ... It's kind of a bit sort of like pot marked, it's not like purely black. There's little white bits on it (20100229\_446).

                  - INTERVIEWER: Does it [56] have strange markings? AB: The one down here doesn't (20100320a\_124-125). The one down there is a very plain small 5 (2010030520a\_151).

                All kinds of markings on it (2010030520a\_151). Seems like one bit of it is made out of feathers (2010030520a\_153) The other bit of it is kind of black and velvety (2010030520a\_155). that's kind of up here. It's kind almost on brown paper and yeah it's kind of, it's quite a decorated 5. It's kind of like some kind of montage (2010030520a\_199). Sort of made out of all different fabrics and things (2010030520a\_201).
              + -


              6

              - [6 in a corridor] it's got some patternings on it. INTERVIEWER: What, the crossings? Yeah. (20100408\_221-223).
              + -


              3

              - [white page] it's kind of black and a few white markings on it, a white sort of spot. It's like a poor quality photocopy (20100415\_37)
              + -


              2

              - + -


                [free space1]

                - more shiny and sort of metallic (20100415\_149)


                  Shiny kind of metallic sort of shine to it. Not really that black (20100415\_153)


                  slightly metallic shine to it (20100415\_163)


                  Just kind of a dark metal. There's a bit of a kind of purple colouring in it, like one of the 5's I saw a long time ago (20100415\_165)
                + -


                [with other numbers]

                - just kind of black. Kind of soft texture (20100415\_149)


                  smaller one is black (20100415\_157)


                  Smaller, thinner black, sort of just jet black (20100415\_161)
                + -


                [free space2]

                - so it's got a metallic outline and then kind of black in the middle (20100415\_173)
                + -


                [free space3]

                - + -


                  It's got the silver metally thing and the sort of outline and sort of purpley thing on the inside (2010415\_175)

                  - [attempting to modify] You get a bit of green at the bottom. Hard to get it fully metallic (20100415\_189)
                + -


                [free space4]

                - Kind of metallic one appearing briefly over there in the middle of the space, and that one won't really change. (20100415\_196). AB: So it's like a big thick, fat like dark 2, almost black. INTERVIEWER: How hard would it be for example, to raise the luminance and less dark and more metallic?   
                  AB: Erm, quite hard. I mean I got a bit more light, like a light kind of appeared above it, shone on it. It's pretty dark, a little bit of reflection of white light off the side of the 2, but not that much more (2010415\_200-202)
          + -


          fractions

          - + -


            When requested, AB is able to view decimals and fractions between integers in the number line. When initially asked to explore what is "between one and two" (20100229\_8) AB sees "one, one point five and two" in a straight horizontal line rather than at the angle of the normal number line (20100229\_9-14). AB is also able to see this decimal represented as the fraction "three over two", "superimposed" on top of the 1.5, "as if you'd written them on the same pi.e of paper, one on top of the other" (20100229\_22-29). On request, AB is able to view an alternate representation of the fraction 3/2 as "six over four". This representation initially appeared quite briefly and AB reports it "likes to go back to three over two" (20100229\_54-55). Six over four stabilises  "slightly up and to the right" of 1.5 and 3/4 (20100229\_59-63). Following the verbal inducer "one third", AB experiences "one over three" in correct position relative to zero, 1.5 and 1 (20100305\_166-167). Similarly one third can be represented as 0.333, superimposed on the one third with the infiinite decimal places trailing away from AB (20100305\_168-169).

            - they are all black (20100229\_39).

              + -


              4.5

              - INTERVIEWER: ... imagine the number that is sitting between 4 and 5.  AB: So I saw 4.5. So, first I sort of saw 4 and then I saw 5 and then I saw 4, 4.5 and 5 all in a horizontal line. (20100229\_2-3).


                INTERVIEWER: ... old favourite the 5, what does the slope look like? AB:  4, 5, 6, 7, 8. INTERVIEWER: And when let's say 4.5 what happens to the slope? AB: <pauses> So, I saw 4.5 for quite a while with nothing else extra and when other numbers did come into view 4 and 5 that's horizontal. INTERVIEWER: Ok, so in both cases it was turned to horizontal? (20100229\_16-20).
              + -


              1.5

              - INTERVIEWER: Ok. But if you like there was a tendency towards the 3 over 2. Could you describe that? AB: But now I've got both, they're not in the same place (20100229\_64-65).


                So now I've got 6 over 4 as being slightly up and to the right of the 3 over 2 .... It's still overlapping slightly. ... Erm <pauses> so the 6 is kind of unimpeded but then the 4 is getting in the way of the 3 (20100229\_59-63).


                INTERVIEWER: So what if I said think of a different representation once more about these fraction. I mean we could say for example 6 over 4? What do you see? AB: <pause> Quite fleetings. It's kind of appearing in the same place. And likes to go back to 3 over 2 (20100229\_54-55).


                Erm, <long pause> Quite hard to focus on the whole thing as it's a little bit blurry. Erm I can zoom in on one bit of it and sort of see a decimal point, sort of slightly above the line that separates numerator from denominator, slightly to the left of centre. So you got the line separating 3 over 2 and then I've got the point. The decimal point is kind of, slightly above the line and slightly to the left. Slightly to the left of the centre of the line. (20100229\_41).


                Yeah they're just flat on each other. I'm just seeing them on a page basically. INTERVIEWER: Is it the white page? AB: Yeah. INTERVIEWER: Is the white page still there? AB: Yeah, the white page is still there this time.  (20100229\_45-49).


                INTERVIEWER: Can you change the representation of the fraction? AB:  Yeah I can see there is 3 over 2 (20100229\_22-23)


                INTERVIEWER: Can you see any difference between 3 over 2 or 1.5? AB: See them as super imposed, one on top of the other. (20100229\_24-25).


                Erm, <pauses> yeah I can kind of see just like a juxtaposition which one is on top. I can make the 1.5 fade away or I can make the 3 over 2 fade away  (20100229\_27).


                That just as if you've written them on the same bit of paper, one on top of the other (20100229\_29).


                INTERVIEWER: does that mean they are hard to recognise, I mean it would create a mess right? AB: No, they are easy to recognise, erm (20100229\_30-31)


                So the 3 over 2 occupi. more vertical space. So all of the numbers I think are the same size, like all the actual digits. So you got the 3 over 2 and then you got the 1.5 and that (20100229\_33).


                INTERVIEWER: Ok, so they are quite easy to separate because of the 3 over 2 is? AB: Sticking out. INTERVIEWER: Sticking out? AB: Yes, they're not totally covering each other up (20100229\_34-37).


                INTERVIEWER: ... So if we take between say 1 and 2, what do you see? AB: I see 1, 1.5 and 2. It's very interesting, it's horizontal. <quiet> INTERVIEWER: Its normally not horizontal?  AB: Well 1 to 2 without any decimals in between is like that INTERVIEWER: Right AB: But 1, 1.5, 2 is… <pauses> INTERVIEWER: Straight? AB: Straight. (20100229\_8-14)
              + -


              1/3

              - INTERVIEWER: Ok so what if I say one third, how does that appear? AB: So there I kind of, it appeared as one over three. Not quite sure of the order of how things appeared but final image was 0 then one third was quite close to one half and then one is over here. So all those numbers appeared (20100305\_166-167).


                INTERVIEWER: So can you see them in the other notation, not as a fraction but as a AB: So I can see a point three three... three... maybe something intangible going further away from me like the pi, kind of on top of the 1 over 3. (20100305\_168-169).


                How many 3's. Hard to tell, they're kind of like getting smaller and smaller and going off into the distance. (20100305\_171)


                so sort of 0.3 slightly to the left of the 1 over 3 and then there's a, then they get smaller and end up very close to the 1 of the 1 over 3 and then they are going off sort of diagonally to the right and behind of the 1, into the distance. (20100305\_173).

          + -
          thousands
          - + -


            The inducer "one thousand, nine hundred, ninety five", was reported with a thousands separator comma, "one, comma, nine nine five" (20100320a\_286-287). The inducer "nineteen eighty one" appeared with a thousands separator comma when that inducer was understood to relate to numbers rather than years (20100320a\_282-285).

            - INTERVIEWER: So if you were thinking of them as numbers how would they be different? AB: Don't know. Well I saw a comma between the 1 and 9 actually. <laughter> ... Yes, so a comma just kind of flickered between the 1 and the 9 there (20100320a\_282-285).


              Well the nineteen eighty one kind of got a comma added to it (20100320a\_300).


              INTERVIEWER: So if I tell you what about one thousand, nine hundred, ninety five? AB: Up here. 1 comma 9 9 5 (20100320a\_286-287).
      + -


      mathematical concepts

      - + -


        In discussing mathematical concepts, items such as alternate equations appear automatically following a related inducer. The concepts themselves are accompanied not only by visual experiences of letters and numbers to multiple decimal places, but also by their associated graph forms and spatial segregation. Numbers such as pi are experienced in their correct position in relation to the integer on the number line. A "black area" or "black hole" is experienced when the concept involves a number with infinite decimal places, with the decimal numbers trailing towards the black hole, "sucking up all those infinite digits ... just sort of get them out of the way" (20100229\_200). The black hole is flat (it doesn't have any depth within the corridor) and AB can change his perspective to view the black hole from behind.

        - + -


          c & Plancks

          - + -


            The interviewer asks AB to examine his experience with a physical constant, AB suggests the speed of light. (20100320a\_195-196).  
              
            AB describes a visual experience of "C = three times ten to the eight... the approximation that's usually used [in reference to the speed of light]" (20100320a\_200) and "Planck's constant, like the H bar" (20100320a\_202). AB attributes his experience of Plancks' constant to its association as "another fundamental constant of the universe" (20100320a\_211-214). Other apparently associated items which AB automatically experiences are "H bar equals H over 2 pi" (20100320a\_224), "E equals mc squared" (20100320a\_208), "E equals H bar times F" and "H bar times a Greek nu" (20100320a\_219-222). AB reports that these equations of F and nu are "the same equation and a different notation" (20100320a\_224), although he is unsure whether the specifics of the equations are correct (20100320a\_224). These concurrents are described as existing on "a white board [AB] can scribble on" (20100320a\_210), reminiscent of the white page on which most concurrents are initially experienced.

            - INTERVIEWER: Did it happen before you saw the equation C equals 3 times 10 to the 8? AB: Yeah, it happened before the equation (20100320a\_203-204).


              INTERVIEWER: What about a physical constant? AB: Like the speed of light (20100320a\_195-196).


              I can already see a C equals 3 times 10 to the 8. So I'm seeing the approximation there that's usually used (20100320a\_200).

              + -


              Yeah, what else came into view was Planck's constant, like the H bar. I just saw like a H bar and then over here there's an equals and a C square came up (20100320a\_202).

              - INTERVIEWER: Did it happen before you saw the equation C equals 3 times 10 to the 8? AB: Yeah, it happened before the equation (20100320a\_203-204).

              Here is the C equals 3 times 10 to the minus 8. Plank's constant is a little bit behind and then there's the E equals mc squared is up here (20100320a\_\_208)


              It's almost like there's a white board I can scribble on (20100320a\_210).


              INTERVIEWER: So how come the Plank's constant appeared? ... AB: Yeah, I guess because it's another fundamental constant of the universe. It's like C (20100320a\_211-214)


              INTERVIEWER: But you didn't see any digits appear with it [Planck's]? AB: No, they are sort of coming along now (20100320a\_215-216).


              Yeah, it's [Planck's] kind of behind the C, it's difficult to move. <laughter> I get it over here. H equals 6 times 10 to the minus 34 (20100320a\_218).

              + -


              INTERVIEWER: Do you see any equations or graphs? AB: Yeah, I got E equals H bar times F.... And just underneath I've got H bar times a Greek nu as well (20100320a\_219-222).

              - Well those are the same thing, the F and nu, the same equation and a different notation. I've got H bar equals H over 2 pi. Can't even remember if these are correct or not (20100320a\_224)
          + -


          alpha

          - + -


            AB is asked to volunteer any other physical constants which he is particularly familiar with. Here AB reports that "Alpha has come to mind" (20100320a\_226), although with little detail in the description and obvious difficulty in recalling the definition ("It's kind of like a string, string length or the tension of the string or something like that. Can't remember" (20100320a\_227-228)). Other items associated with alpha are also experienced: "alpha prime came in with a dash" (20100320a\_230).

            - INTERVIEWER: What about in string theory, are there any constants there you had to work with through a lot during your PhD? AB: Alpha has come into my mind (20100320a\_225-226)


              INTERVIEWER: What does alpha mean? AB: It's kind of like a string, string length or the tension of the string or something like that. Can't remember (20100320a\_227-228).


              INTERVIEWER: It's just alpha and nothing else? AB: Yeah, well there's alpha and there's alpha prime came in with a dash (20100320a\_229-230).


              INTERVIEWER: ...so [the value for alpha] it's different for each string? AB: There's a universal constant (20100320a\_231-232).


              INTERVIEWER: So what are the digits? Can you see any digits associated? AB: No (20100320a\_235-236).
          + -


          e

          - + -


            AB informs the interviewer that he also has synaesthetic experiences with irrational numbers such as e. When asked to examine this experience AB reports a detailed scene involving both related equations and graphical representations.  The initial experience following the inducer "e" (with prior arrangement that this was in relation to the mathematical concept, not the letter E) was "a flash of the exponential curve and then I saw the letter e, it took a while for the equal sign and numbers to appear but then they are there" (20100320a\_71-72). AB can initially report e to six decimal places, after which he experiences instability and a "dot dot dot" (20100320a\_74).  The graphical representations of e include "little circles coming up in the back like little complex numbers appearing and a little circle unit radius appearing there intermittently as well" and "an exponential curve" (20100320a\_74). There appears to be a clear spatial separation between these different representations: "That's from over there and then there's an e equals two point seven, eight, one, eight, two, eight here. There's kind of an exponential curve over there" (20100320a\_74); "one [graph] over here and one [graph] over there" (20100320a\_137-138). AB's confidence in these spatial relationships enables him to draw a schematic of this experience (AB\_drawing\_20100320a\_E.jpg).   AB describes these representations as occuring in a context with similarities to the corridor context: "floating against a greenish, orangey background again" (20100320a\_78). However the corridor is not explicitly mentioned and there are notable differences in the lack of a reported line, walls, floor etc "just kind of pure kind of background" (20100320a\_99-100) and that the background is "kind of a lighter more towards the green this time" (20100320a\_78). AB reports that during this time he does not have any of the physical room present (20100320a\_101-102). The context for the graphs appears to be quite distinct from that of the e equation, with the unit radius on a "red" background (20100320a\_162) and the exponential graph being uncoloured (20100320a\_171).

            - INTERVIEWER: What about E? AB: So I've got a flash of the exponential curve and then I saw the letter E, it took a while for the equal sign and numbers to appear but then they are there (20100320a\_71-72).


              2.78 erm 1828 and the dot dot dot and there's another 1828 kind of floating behind, kind of a dot dot dot as well. I'm getting little circles coming up in the back like little complex numbers appearing and little circle unit radius appearing there intermittently as well. Circle representing E is to imaginary powers. That's from over there and then there's an E equals 2.781828 here. There's kind of an exponential curve over there. <Quietly spoken> (20100320a\_74).


              INTERVIEWER: ... it took a while for the equals sign to appear, and then the numbers appeared or did they appear the same time as the equals sign? <AB agreeing throughout> AB: Erm, as I just kind of moved my attention from left to right I became sort of aware of them and could see them. (20100320a\_75-76).


              Yeah, it's on a page. Seems to be more floating against a greenish, orangey background again actually. Kind of a lighter more towards the green this time. Yeah, black ink (20100320a\_78).


              Erm, so if I focus my attention on E <pauses> Well I'm just aware of numbers but I can't really say what numbers. I'm only seeing the 2 and maybe the 7 but after, as soon as I focus on them then they kind of, loose my attention <voice trails off> (20100320a\_80).


              INTERVIEWER: Ok, just as a comparison and turn your attention back to the E and try doing the similar trick. AB: Yeah, it gets more and more vague the further away I go. INTERVIEWER: Can you see 7 8. AB: I see equals and the 2.7, but I can't really see beyond that really clearly (20100320a\_87-90).


              I'm kind of right in front of me is the 7. So I've kind of got to move to my left to see the E. It's quite hard to have the E in the middle my focus. It seems like I move it back to the equal sign.  (20100320a\_\_94).


              INTERVIEWER: So which one is the most natural focal point? AB: So maybe the equal sign. (20100320a\_95-96).


              INTERVIEWER: Can you see what is in the room around the equals sign, while keeping your attention focused on it? AB: Very hard to keep my attention totally focused. There's kind of a greeny, orangey colour. I don't see any details, just kind of pure kind of background (20100320a\_99-100).


              INTERVIEWER: But it doesn't have anything of the physical room or presence? AB: No (20100320a\_101-102).

              + -


              graphs

              - + -


                background

                - It's like it's a big, just red, everything sort of after the y axis seems kind of red (20100320a\_162)


                  But the exponential graph is without colour. (20100320a\_171).

                INTERVIEWER: If you go back to E, do you see the two graphs that you mentioned? AB: Yeah, kind of one over here and one over there. (20100320a\_137-138)
          + -


          pi

          - + -


            When the interviewer provides the  inducer pi to four decimal places AB reports, "Greek letter pi equals and then I've got three point one, four, one, five" (20100229\_66-69). AB is also able to report a further four decimal places, although he is unsure whether these numbers are correct and the experience appears vague or unstable: "I'm not even sure if these are right now but I am seeing two, seven, nine, six too. And then just some kind of vague numbers just kind of going off and I can't. I'm not really sure what they are because they are sort of an eight and then another six, two... Erm yeah, seems a bit unstable" (20100229\_72). The "dot dot dot" is reported with a dark area or "blackness" (20100229\_112) towards the end of the corridor where the numbers disappear into infinity.  This experience is initially in the context of the white page and then follows the usual transition of melting and entering the corridor (20100229\_108; 20100229\_112). AB is also able to see the definition of pi in the correct position relative to the integer three.

            - background darker than root2 (20100320a\_43)


              INTERVIEWER:  So, shall we try pi.  AB: Yeah. INTERVIEWER: So 3.1415 and so on. AB: Yeah. So I see the Greek letter pi equals and then I've got 3.1415 (20100229\_66-69).


              2, 7. I'm not even sure if these are right now but I am seeing 2796 too. And then just some kind of vague numbers just kind of going off and I can't. I'm not really sure what they are because they are sort of an 8 and then another 6 2. Erm yeah, seems a bit unstable  (20100229\_72).


              So it's unstable after 3.141527986. Seems pretty unstable after the third or fourth decimal point. (20100229\_73).


              The sequence in the beginning is displayed on a white page and then somewhere it's kind of leaving that and not really sure how. For a bit it's against that kind of erm greenish, reddish orangey background that I think is the same background we were talking about last week and then some point at erm there's this sort of as you go towards the right there's this blackness just sort of hovering there and it's kind of going into that (20100229\_108).


              So this time the white page is kind of melting away and I am kind of against that kind of coloured background again. Then I got pi equals 3.1415, still a 9 up there and 2 there. And then so there's that kind of coloured background behind all of those. And to the right of those there's this kind of blackness <pauses> and so what's happened to the other numbers. So then they kind of for a bit appear on the sort of floor. (20100229\_112).


              INTERVIEWER: So if you keep in mind pi and you also try to think of 3 at the same time ... AB: So I saw a 3 there ... So now I'm still in this kind of coloured sort of corridor. Erm it's a darker sort of light, it's almost, almost like some kind of subterranean place. There's a slight glow of like sort of flames or something like the end of the world or something. Erm so I can see 3 and then I can see pi equals 3.1415 9. Now the 2 is above the 9. They are still not stable <laughter and cross-talking>. And then there's some numbers kind of on the floor in front of the blackness which is covering the wall on the 9 and just kind of going off. ... They're just potentially numbers that could go into the sequence but they don't know where to go (20100229\_113-118).


              INTERVIEWER: But in terms of pi, if you go back to pi, where does the number line head off to? Where does it intersect the wall? AB: Erm, I don't really have a number line appearing in that image. (20100229\_189-190).

              + -


              drawing\_20100229\_pi.orridor

              - Yeah.  So that's the corridor. So then I have 3, then I have pi equals 3.14159 and this is where I have difficulty seeing it. There's this black hole right here and then there is sometimes a few numbers just kind of on the floor here. Sometimes the dot dot dot really changes. Sometimes like little sequences like 8 5 6. Sometimes just loads of numbers just scattered on the floor like that. And then sometimes I see it just kind of curved into there. I mean imagine being sucked into there and then 3.5 is safely over here. (20100229\_236).
          + -


          root 2

          - + -


            When asked to describe the experience of the square root of two, AB reports a visual representation of the equation, including mathematical operators, and instability after two decimal places: "So I saw a briefly flashing the square root of two equals one point four, one, and then like there's a whole load of other numbers to the right, which are not really settling down" (20100320a\_11). This is initially in the white page context and then transitions into the corridor (20100320a\_19-21; 20100320a\_22-23).

            - + -


              background description matches corridor

              - sort of orangey  (20100320a\_41)


                greeny-orangey (20100320a\_100)

              So I saw a briefly flashing the square root of 2 equals 1.41 and then like there's a whole load of other numbers to the right, which are not really settling down. Just seem really random, there's a like kind of a 2 8 7 6 5 4, but that's not the correct expression of it. INTERVIEWER: Ok so to recap, when you thought of the square of 2, you saw the symbol for the square root with the 2 underneath it and the equal sign... (20100320a\_11-12).


              This time they are all kind of on the page, kind of all crowding around each other, much sort of closer together. INTERVIEWER: And so in all of this you had the white background present? AB: Yeah (20100320a\_19-21).


              INTERVIEWER: When you focus on the background what happens? AB: Erm, kind of got the same kind of coloured background I usually have. Kind of like the slightly lighter one not the one I had for pi. Sort of black ink, against the sort of greeny orangey background. (20100320a\_22-23).


              Erm no, it seems a bit lighter than the pi. And it's still kind of floating in the middle of space <external noise> INTERVIEWER: So it hasn't settled down? AB: Doesn't seem to be on the floor this time. Nor just kind of against a wall. (20100320a\_43-45).


              Erm, now I see 1 2 like this, number 1, number 2. The square root of 2 is kind of floating above in the middle of the room. (20100320a\_47).

              + -


              drawing\_20100320a\_root2

              - Erm, so you've got 1 here and 2. Number 1, number 2 and then the square root of 2 is coming across like this, although it's up just here. INTERVIEWER: Ok, it could fit down there if it wants to? AB: It could fit down there because it's kind of horizontal. The number line is kind of like that. INTERVIEWER: And so the wall that you were describing was also cutting across the number line? AB: Erm yeah, exactly. (20100320a\_49-53).


                INTERVIEWER: So we have a 1 and a 2 with a number line and then I got cutting across it where there is a gap in the number line, we have square root of 2 equals 1.4128765 and then there was a 4 with a 2 floating above, and then we have dot dot dot and a collection of  (20100320a\_63).

              It's kind of dropped a little. It's kind of fluctuated between being up here and down there. I saw it for a moment dropped. AB: Seems to be more down now. INTERVIEWER: Did you actually see it moving down or did it just appear at the bottom? AB: Just appeared at the bottom as I was focusing on the number 1 and number 2. (20100320a\_55-59).
          + -


          infinity/black hole

          - + -


            Where AB is describing an experience or concept which is contains and infinite number of decimal places (e.g. the square root of two or 1/3) he describes a "blackness" (20100320a\_132), "black hole" (20100229\_200), or "black sludge" (20100320a\_39) in the corridor. This black hole appears to hang in the corridor 'downstream' from the decimal numbers, invariably at the point where AB can no longer report the numbers with confidence.   For example, the "black hole" is described in association with the infinite decimal places of e (20100320a\_126) and pi, where the "intangible group of numbers [towards the unstable end of pi are] going off towards this black hole" (20100305\_5). Infinite decimal numbers appear to go "into the middle of it [the black hole]" (20100229\_105-106). Similarly the decimal places of one third are described as "going off sort of diagonally to the right ...into the distance, ...[towards] another kind of like black square" (20100305\_173-175).   The black hole is also associated with the "dot dot dot" representation, where the dot dot dot is described as "going slightly behind the numbers [further down the corridor] going towards the black hole " (20100305\_11).

            We might speculate that this "black hole" is associated with a semantic representation infinity. For example, the black area in the corridor is described as "sucking up all those infinite digits in pi to just sort of get them out of the way" (20100229\_200). On another occasion the "endless decimal places of phi, pi... [are] going off into kind of like a black void" (20100229\_99-102) which is also described as "being this infinite thing" (20100229\_161).  The corridor doesn't end at the black hole, rather the black hole appears to block the back half of the corridor although it is itself flat (the black hole it doesn't have any depth in the corridor) and AB can change his perspective to the exit of the corridor from the other side of the black hole (20100229\_119-122).

            - So, it's like that black hole is sucking up all those infinite digits in pi to just sort of get them out of the way. Otherwise they are going to crash into 3.5 (20100229\_200).


              So I'm seeing in this kind of erm slightly infernal corridor <laughter> and the pi equals 3.14159 and then kind intangible group of numbers going off towards this black hole <laughter> (20100305\_5).


              then there's kind of a dot dot dot kind of going slightly behind the numbers going towards the black hole. And then there's sort of over here, sort of some digits just on the floor (20100305\_11).


              [1/3] ... and then they are going off sort of diagonally to the right and behind of the 1, into the distance. INTERVIEWER: Do you see where they end? Where is this distance leading to? AB: There's another kind of like black square, then sort of going off to (20100305\_173-175).


              [root 2] No I didn't see a black spot at the moment. Or I am starting to become aware of it over here so root two equals, no numbers and then there's kind of a black spot here (20100320a\_29).


              [root 2] Erm, yeah it's kind of like a black sludge. They are all kind of overlapping the numbers. I'm sort of aware of the presence of mainly a couple of 6's and a couple of 5's (20100320a\_39).


              [e] It's kind of like a black hole from here (20100320a\_126).


              [e] When trying to focus on the blackness there's nothing there, so I'm totally unfocused on anything (20100320a\_132).


              I'm sort of seeing a dot, dot, dot and then sort of. There seems to be a rotation where it's starting to go off in that direction away from me.  ... When I focus on it there, like those endless decimal places of phi, pi and ... It's going off into kind of like a black void (20100229\_99-102).


              [pi] INTERVIEWER: So, how does this black hole relate to the number line? Is it above it AB: Numbers sort of going into the middle of it (20100229\_105-106).


              [pi] INTERVIEWER: How does the black hole appear on the wall? Is it part of the corridor? AB: It's just like a smudge. ... INTERVIEWER: Does it look like it has any depth or is it flat? AB: It's flat and it's just covering. It's like kind of in like rising so it's like in the middle of the corridor. It's like blocking the back half of the floor and the blocking the wall. And doesn't go on forever though there's like the corridor is a. I can see the wall of the corridor again if I go over here with a kind of white light at the end of the corridor. (20100229\_119-122).


              [pi] There's this big black mass there. Just concentrating on the black then it kind of flickers for a bit and the corridor just kind of comes back for a second (20100229\_124).


              [pi] Yeah so the black smudge is just this kind of presence just blacked out. Since it's being this infinite thing (20100229\_161).

              + -


              form descriptions\_mathematical concepts\_infinity/black hole\_pi hole

              - Erm it's a darker sort of light, it's almost, almost like some kind of subterranean place. There's a slight glow of like sort of flames or something like the end of the world or something (20100229\_116)


                towards the right there's this blackness just sort of hovering there and it's (decimal placecs of pi. kind of going into that (20100229\_108)


                And to the right of those there's this kind of blackness <pauses> (20100229\_112)


                And then there's some numbers kind of on the floor in front of the blackness which is covering the wall on the 9 and just kind of going off (20100229\_116)


                It's just like a smudge (20100229\_120)


                It's flat and it's just covering. It's like kind of in like rising so it's like in the middle of the corridor. It's like blocking the back half of the floor and the blocking the wall. And doesn't go on forever though there's like the corridor is a. I can see the wall of the corridor again if I go over here with a kind of white light at the end of the corridor (20100229\_122)


                There's this big black mass there. Just concentrating on the black then it kind of flickers for a bit and the corridor just kind of comes back for a second (20100229\_124)

                + -


                form descriptions\_mathematical concepts\_infinity/black hole\_pi hole\_dot

                - Sometimes the dot dot dot really changes (20100229\_236)


                  dot dot dot (20100229\_140-141)

                then kind intangible group of numbers going off towards this black hole (20100305\_5)

              No, the colour seems pretty stable. There was, when I was thinking of pi it was kind of, I dunno, it was like a sort of black hole was kind of causing it to have more of a kind of slightly darker, darker kind of glow. Seemed more kind in the underworld. Erm, than the rest of the number line. It was a sort of more cheerful sort of colour, more sort of greens in there I think. (20100305\_65).


              [e] It's kind of like a black hole from here. (20100320a\_126)
      + -


      alphabet

      - + -


        AB's synaesthetic form for the alphabet initially appears as capital letters (20100329\_215) in a type face similar to arial (20100329\_337-339) with a "ghost-like" translucency (20100408\_313). Specific patterns or textures are generally not reported with the letter form, however on close inspection of the letter B it is described as though "it's made of ribbons attached to each other" (20100408\_329).

        - + -


          general feature description

          - + -


            AB's synaesthetic form for the alphabet initially appears as capital letters (20100329\_215) in a black type face (20100329\_216-217) similar to arial (20100329\_337-339) with a "ghost-like" translucency (20100408\_313). Surrounding concurrent are generally present with the induced concurrent, noting however that A and Z were not initially connected (20100329\_290-293).

            - K is there. INTERVIEWER: So, would you say it's in front of you? AB: Yeah, eye level (20100329\_263-265).


              AB: just a fairly plain font. INTERVIEWER: Something Arial or? AB: Yeah, something like that. (20100329\_337-339).


              Capital letters. (20100329\_215)


              INTERVIEWER: So that looked quite big the way you drew it in the air. AB: Yeah, quite big (20100329\_278-279)


              INTERVIEWER: Capital letters and do they have the same texture and appearance as the numbers. AB: Yeah, I guess pretty much. Yeah, it's kind of black, (20100329\_216-217).


              Well it's like the M appeared there. So it's slightly to my right actually it's quite surprising. Looks like it's kind on the same horizontal plane as where the numbers are appearing kind of eye level. The one you made me think of and then the other ones have to go there. N O P have to go down because that's the way the line goes (20100329\_219)


              INTERVIEWER: Is there anything connecting the M to the L? AB: No, just side by side. (20100329\_238-239). They are all on a white page next to each other (20100329\_241)


              INTERVIEWER: Can you zoom out and have a look at the whole alphabet? AB: <pause> Erm yeah. (20100329\_268-269).


              INTERVIEWER: So the zoomed out version of the alphabet disappeared when you switched your attention to the A? AB: Yeah. I mean it's kind of in the distance. It's probably further away from me than I can see. INTERVIEWER: But the A is still appearing? AB: Big A up there. INTERVIEWER: Is that A connected to the alphabet? AB: Yeah, it's kind of in the position of where the A is in that alphabet that is far way. ... Erm, this alphabet is kind of here A B C. INTERVIEWER: Oh it's a different one, sorry? AB: Yeah  INTERVIEWER: So the zoomed out one has moved into the distance and now you have a new one that's appeared with the bigger letters. <AB agreeing throughout> (20100329\_280-290)


              INTERVIEWER: do you have any sense that there is something to the left of the A? AB: No, nothing. INTERVIEWER: There doesn't seem to be anything there? AB: No (20100329\_290-293) AB: I kind of got a Z appearing up and to the left of the A for a moment there. Smaller Z. <laughter> Only after a bit you said is there anything there (20100329\_297).


              INTERVIEWER: Ok, what if I say to you, how many letters are between C and J? AB: Yeah, first number that came into my head was 5, that's a little bit wrong isn't it. D E F G H I. ... Basically, what happened I kind of saw the alphabet form and kind of guessed how many letters were in between, without kind of counting them. ... So the J was there and the C was there, I guess there were about 5 letters in between (20100415\_227-232).


              it's just like an ordinary Y, lower case Y in black ink (20100329\_327)


              Don't know what material it [B] is made of, or if it's solid at all (20100408\_309). like it could just be projection of the B or something (20100408\_311). Well it's not against a wall or anything so I don't see how it could be a projection. But it seems like it's almost like a ghost, like you could put your fingers through it (20100408\_313). the B is kind of translucent, ghostlike (20100408\_315). B has a kind of ghostlike quality to it (20100408\_337).
          + -


          patterning

          - + -


            When AB is directed to focus intently on the form of a B concurrent, he reports that the individual 'strokes' of the B appear as though "it's made of ribbons attached to each other" (20100408\_329). AB reports however that this focused attention seems to "change what it's made of a bit" (20100408\_333).

            - I can focus on the individual lines of the B. I mean if I really focus on it. Now it's like it's made of ribbons attached to each other (20100408\_329). I mean really staring it seems like it changes what it's made of a bit (20100408\_333)
          + -


          context

          - + -


            Letter forms appear in the context of the white page, a cave or free space.

            - + -


              white page

              - Just the white background (20100329\_225)


                It's kind of like a white page. Then I've got a sense of that lifting up and revealing a kind of background again (20100329\_227).
              + -


              cave

              - [background] it's just kind of misty. I'm sort of seeing the [physical] room behind. But then I sort of feel the letters are in a different kind of space as well. It's just of, I don't know, browny kind of mess [the cave] behind  (20100329\_235).
      + -


      age

      - + -


        AB experiences a form similar to his number line (digits and a line) when exploring concepts of age, however he reports that it may be different in a sense as the numbers (e.g. 'twenty eight') are more often used in the context of age rather than the pure mathematics with which he is familiar (20100320b\_90).  
          
        AB's experiences for prenatal ages (conception to birth) are accompanied by static visual scenes from embryonic development to a hospital scene and a woman in labour (20100320b\_103-138). These scenes have precise spatial relations to one another and are identifiably different from a visual memory by AB's dissociated position within the scene. At birth (age 0), the pictoral scenes revert to the normal number form-like experience, however he refers to early age in months, i.e. 6 months, 12 months, 18 months old, rather than using fractions.

        - + -


          different from number line

          - + -


            AB's spatial for for ages has a similar appearance to his number line, INTERVIEWER: "So if I say I'm 28 what happens? AB: So I see two, eight. INTERVIEWER: Right, is it any different to me saying imagine 28? AB: No, it doesn't seem that different actually. Kind of the 27 beneath and the 29 above" (20100320b\_85-88). AB does however report to "get a sense that it might be different" [from the normal number line] (20100320b\_90) and suggests that this may relate to his familiarity to the lower numbers more often used in pure mathematics where as the larger ones are used "more often in the context of peoples age" (20100320b\_90).

            - INTERVIEWER: So if I say I'm 28 what happens? AB: So I see 2 8. INTERVIEWER: Right, is it any different to me saying imagine 28? AB: No, it doesn't seem that different actually. Kind of the 27 beneath and the 29 above. INTERVIEWER: So it could just be a variation of the number on the number line? AB: Yeah. While I got a sense it might different. There's more kind of the higher numbers that are less familiar from kind of pure maths and kind only really used in the context, more often the context of people's ages. (20100320b\_85-90)
          + -


          conception-birth

          - + -


            When examining concurrents for concepts of age, AB reports distinct static visual experiences which represent stages in a foetal and neonatel development.   
              
            At age 'zero' AB describes a scene of a woman giving birth. The description of the scene contains doctors and nurses in a hospital room. Details described in the scene include medical implements on a tray, storage drawers and a work surface, a woman lying on a bed, a window which looks onto the hospital corridor and some unknown electrical equipment over the bed (20100320b\_153). Each of these details have a precise location in space and AB reports that he can hear the sound of a baby crying from somewhere outside the scene (dissociated from the scene) but no other sounds from the hospital room itself (20100320b\_139-149).  
              
            When AB is asked if there is anything 'before' that scene, as with negative numbers, reports a "vague sense of something in the womb" (20100320b\_110) but with an unknown or indistinct location. When the interviewer unintentionally induces "6 months pregnancy" AB is able to identify the location of this age which and report "a sort of fairly black womb with a sort of like foetus in it" (20100320b\_112).   
              
            AB also describes other scenes in seperate spatial locations, including conception "at the very top" as a "magnified picture of an egg with loads of sperm" (20100320b\_127) then progression towards a a fertilised egg and a "clump of cells" (20100320b\_114) followed by further cell divisions in the embryonic development. There is a suggestion that these stages are accompanied by a line similar to the standard number form, but this is not conclusively investigated (20100320b\_113-124).   
              
            Zero and one years of age are separated in space to accommodate "all those first months in the first year of life" (20100320b\_182). When directed to 6 months old AB reports hearing a baby cry and a closer to the zero time point a newborn baby "wrapped up in white blankets" (20100320b\_192).  
              
            The interviewer then proposes that the first year might contain images of a baby "crawling around on the floor, or something like that" (20100320b\_195). At this stage AB remarks that he is beginning to experience "a few more general images" (20100320b\_196) of an occasion where he saw a friends child at one year old. Usefully for the purposes of this exercise AB spontaneously offers that these images are quite distinct from those described in the above; in the "more general images" (ibid.), which we may speculate are reconstructed visual memories, AB is positioned within the scene and it is surrounding him (20100320b\_196), where as in the pictoral representations of age AB did not report himself as a feature of the scene. 

            - INTERVIEWER: What happens at the zero?  AB: Kind of like an image of a woman giving birth <laughter> And there's a number zero <laughter>  INTERVIEWER: You mean there's an image of giving birth. Do you see a woman? AB: Little baby. INTERVIEWER: Is it moving? Do you see doctors? AB: Yeah, there's kind of like a whole room. There's kind of nurses. (20100320b\_103-108)


              AB: Hear a bit of like baby crying there. INTERVIEWER: So in terms of the baby sounds, does it go with the movement in the scene or is it kind of dissociated? AB: Seems kind of dissociated, but it was kind of coming from over there. INTERVIEWER: But it was more like a sequence of snapshots. AB: Yeah. INTERVIEWER: Can you hear the doctors? AB: No. (20100320b\_139-149)


              INTERVIEWER: What about the medical machinery that's in the room? AB: Erm. INTERVIEWER: Can you see it in the pictures? AB: Well I focus my attention as kind of like erm, kind of like some scissor and a white tray on a long surface. INTERVIEWER: Oh yeah, they all need those! AB: Yeah, there's like lots of drawers. There's kind of like drawers and work surface kind of around here. And then there's like the bed here, like the woman kind of lying down there. And there's kind of like a window there at the back. INTERVIEWER: Can you see outside the window? Is it clear. AB: Just kind of like a corridor. INTERVIEWER: Oh, it's into the hospital and not to the outside. AB: Yeah, into the hospital. INTERVIEWER: Where's the doctor standing in relation to all of this? AB: Err, so there's like one here and then there's like another here. INTERVIEWER: Are they interacting with each other or? AB: Can't tell. It's just kind of snapshots. INTERVIEWER: Oh right. AB: Yeah, there's also some kind of machinery above the bed like. I don't really know what it's for. You mean like a drip or? AB: It's that kind of metallic, kind of structure type thing above the bed. INTERVIEWER: Over effect for the curtain maybe? AB: Yeah maybe. INTERVIEWER: Or is it something electronic? AB: Yeah, so that's something electronic. INTERVIEWER: It is or it is not? AB: Seems like its electronic  INTERVIEWER: I guess there will be some lights up there, but that's not what you mean. AB: No, the room's just kind of well lit. (20100320b\_150-175)


              INTERVIEWER: I guess there's nothing before that I mean. I mean for example that you have negative numbers. Does that make sense in this context? AB: No, Ive got a vague sense of kind of something in the womb but don't really know where it's located. INTERVIEWER: Yeah, I guess you could say that you know, what happens at 6 month pregnancy. AB: So it's kind of over here. Yeah, kind of an image of a sort of fairly black womb with a sort of like foetus in it. (20100320b\_109-112)


               INTERVIEWER: Can you trace it's different stages? AB: Erm, yeah the conception is kind of up here. Like a fertilised egg and of a clump of cells. INTERVIEWER: Right. So is there like cell division going on? AB: Yeah. And then there is kind of like a curve at the back of his head. <laughter> INTERVIEWER: So, how many steps are there for each? Is it monthly the images or? AB: Don't know. Well, the cell divisions are kind of much closer together. And here it's kind of expanding. INTERVIEWER: Ok but what are the divisions? You make a motion as going this way. Do you see any particular points on that line? AB: So the focus here, there's kind of like a new embryo type thing. There's kind of like a black kind of sweeping thing that again, the kind of the width of the line getting bigger as you get to here. INTERVIEWER: Kind of like a tunnel? AB: Yeah. Seems like a tunnel yeah. It's kind of winding up. INTERVIEWER: So the tunnel is not... AB: It's kind there's a kind of like a foetus here and foetus there. So more like an embryo here. (20100320b\_113-124)


               INTERVIEWER: So you do you need to put your attention or to see the stage that point is at in the tunnel? AB: Yeah. INTERVIEWER: So it's kind of like the numbers in that sense, I guess. AB: Yeah. INTERVIEWER: So it starts up there somewhere? AB: So at the very top somewhere there is one of those magnified pictures of an egg with loads of sperm <laughter> INTERVIEWER: So just like you would see in a biological textbook. AB: Yeah, more static. It's not like movement. INTERVIEWER: So these are static ones? AB: Yeah. INTERVIEWER: Even those scenes with the doctor here?  AB: Oh yeah, it seemed to be like distinct scenes. So one point the baby was kind of removed and there was kind of like the umbilical cord <laughter> INTERVIEWER: ok, it was snapshots. It wasn't like a scene unfolding. (20100320b\_125-138)


              INTERVIEWER: Where was the zero in all of this? AB: <laughs> kind of superimposed by that scene at the hospital. INTERVIEWER: And then the 1. AB: So there's a 1 over here for like birthday. INTERVIEWER: Ok it's far away. So it's quite separated somehow. AB: Well it's quite separated as there's all those months in the first year of life. INTERVIEWER: Oh right of course, they have to fit in there. AB: So there's a 6 over here, which was the first thing we looked at. INTERVIEWER: ... But there's no imagery associated with 6 months or the first year <question tone> The imagery stops at zero. AB: Erm, when I said that there was kind of an image of a baby, I heard a baby crying just then. INTERVIEWER: Does it still work for 1 year as well. AB: 1 year, just see the number 1 here. I didn't hear any cry when you said. INTERVIEWER: And there's no baby anymore? AB: Don't see a baby. Well there's kind of like a little baby here that looks like a newly born baby. <laughter> ... AB: Ok. Kind of wrapped up in white blankets. INTERVIEWER: I guess that would be. The first month would be more like that, not the first year. AB: Yeah. INTERVIEWER: Yeah I guess there would be crawling around on the floor or something like that. But there's nothing like that going on. (20100320b\_177-195)


              AB: I'm getting a few more general images. Like, being at my friend's house. My friend in Spain, he's got a son whose just approaching 2. I saw him about a year ago and it's a kind of 1 year old crawling. But that's kind of a whole different scene, sometimes I'm in the middle of a house. All around me like life size. INTERVIEWER: So that's quite a vivid. Is that a memory or a reconstruction? AB: I think it's a reconstruction. <pause> (20100320b\_196-198)
          + -


          infancy

          - + -


            AB reports specific locations of ages in months (e.g. 6 months old, 12 months old, 18 months old). (20100320b\_98). When asked to identify a specific point of "one year and two months" (20100320b\_95), AB reports that he is initially rather than the number is was "more aware of the position" (20100320b\_100) of this point on the line, relative to the other months. However as he soon as he focuses on this position he is able to induce the digits "one, four" in the correct position (20100320b\_100).

            - + -


              INTERVIEWER: So imagine that I met a friend this weekend who told me that his baby was 6 months old. What happens with the month? AB: So it's kind of a 6 here (20100320b\_91-92)

              - seperate number line with months. What is the range for this?

              1 year here, it's kind of 12, 1 year 2 months, 18 months is kind of over here (20100320b\_98)


              INTERVIEWER: What if it's 1 year and 2 months, what happens then? (200100320b\_95) AB: Saw it as kind of... What did come into view? I was more aware of position [relative to the markers for 12months, 18months] than of particular number. Well I can do a 14 there now. 1 4 (20100320b\_100).
      + -


      calendar

      - + -


        AB reports to have a spatial forms for calendar aspects such as years,
        months and times. His year form is similar to his number form (digits
        and a line) but is however dissociable from the number representation of
        the same digits by a lack of a comma thousands separator (1,995 cf.
        1995). Of note is the accidental induction of the year calendar form by
        the wording of the inducer in a 'normal' year format, i.e. "nineteen
        fifty six" cf. 'one thousand nine hundred and fifty six" (20100320a\_237)
        . AB is also able to describe and draw a his spatial form for the more
        abstract year inducer of 4000BC, which includes an inflection point at 0
        between BC and AD.  
          
        AB's spatial form for the calendar year (January
        to December) again includes the experience of digits and a line, however
        in this case they are arranged circularly with August at the top,
        progressing clockwise to February at the bottom. Within the months
        themselves each 10 day segment follows a specific direction on the line
        such that AB can identify a specific date not only by the position of
        that month in relation to the others but also by the direction of the
        numbers on the line surrounding the date.  
          
        AB's reports to experience
        a synaesthetic clock in multiple different forms, including an analogue
        clock face with hour and minutes hands, minute markers and larger hour
        markers, an orange digital clock with a flashing semicolon for seconds
        and a simultaneous digital clock in a seperate area of space and a
        different type face. In directing his attention to different aspects of
        the analogue clock, AB is able to induce the experience of digits on the
        analogue clock face and report the apparent motion of a second hand as
        it flashes "in and out of existence" (20100320b\_16-18) each time his
        attention returns to it.

        - + -


          year

          - + -


            AB is offered the inducer "nineteen fifty six" (201000320a\_237) (which he automatically conceptualises as a year (20100320a\_279)) and describes an experience similar to his normal number form, with the individual digits, one, nine, five, six, a line "under the fifties" (20100320a\_240) and with a background colour similar to the corridor (20100320a\_248). AB then continues to describe the spatial relation between distinct points in this series on the number line, "nineteen ten there... nineteen eighty is up there... two thousand is there..." (20100320a\_250-254), all the while retaining the format familiar to descriptions of year dates. When AB is directed to explore his calendar form for a more abstract year, "four thousand BC" (20100320a\_294), the digits appear in conjunction with the letters "BC" and an inflection point at zero where the years change to AD.

            - + -


              1956

              - INTERVIEWER: Let's get back to just the normal number sequence. Just try to explore some of the bigger ones. What about 1956? (20100320a\_237)


                INTERVIEWER: What about nineteen fifty six? AB: nineteen fifty six, erm. So I see one nine five six (20100320a\_237-238)


                There's kind of a line here under the 50's (20100320a\_240)
              + -


              background (corridor)

              - That kind of orangey, greeny background (20100320a\_248)
              + -


              >=1900

              - INTERVIEWER: So if you look at the beginning of these hundreds, nineteen hundred... does the 9 look different [to the nine in 1956]? AB: It's kind of looking more black now. It's not really stabilising that easily. It's kind of black for a bit then it's kind of hollow, just for the outline in black in. That kind of orangey, greeny background, you can see through the outline in ink. (20100320a\_247-248)


                And then I got the line like this, going up to nineteen ten there (20100320a\_250)


                nineteen ninety eight is up here... two thousand is there (20100320a\_252-254).


                Just filling in the white board again (20100320a\_258).


                1910 there's 1920. I don't know. Then I guess the 20's are maybe coming up here (20100320a\_260)
              + -


              1981

              - INTERVIEWER: When were you born? ... AB: nineteen eighty one (20100320a\_263-266)


                [1981] Erm, so that's over here now. Erm so it's kind of against the kind of greenish background. The 8 is kind of hollow again. It's sort of thinner the numbers. In thinner ink, thinner lines and black (20100320a\_268).


                So it's got nineteen eighty one, nineteen eighty two. Eighty's kind of going like this. Hard to say, almost like a kind of nineteen ninety and ninety one are coming out my left (20100320a\_272). There's some kind of 3Dness going on that I can't quite figure out (20100320a\_276)


                INTERVIEWER: We were talking about the different numbers and we mentioned the year of your birth 1981, but it appeared in the same number sequence, it didn't appear as a year. You said you also have synaesthesia with year. <AB agrees throughout> So if you think about it as a year, does it appear differently? AB: Erm, I think I was thinking of it as a year (20100320a\_278-279).
              + -


              1995

              - + -


                INTERVIEWER: Ok what if I tell you now that say Francisco Varela published a nice paper in nineteen fifty five? AB: Yeah, that's over here; 1 9 9 5 (20100320a\_288-289)

                - INTERVIEWER: Do you have both of them in view, the one comma and? AB: Yes, there's one here and one there. There's 1 comma 9 9 5 and it's kind of thinner letters, thinner digits. And this one's got thicker digits 1 9 9 5, no comma (20100320a\_290-291).
              + -


              4000BC

              - 4000 BC. So first I was kind of not... struggling to find it on the number line. See this kind of curve here which was like, you got 0 and these are kind of AD and back into BC. 10 BC, 20 BC (20100320a\_294).


                Then I was kind of losing touch with it and then 4000 BC just in numbers and the capital B and capital C here (20100320a\_296).


                drawing\_20100320a\_4000BC
          + -


          month

          - + -


            AB describes the spatial form for a year as a roughly circular clock face with specific spatial relations between the months (drawing\_20100320a\_calendar). AB is able to selectively draw his attention to  areas of the form to identify specific dates (20100320a\_335-336) and identify which month he is 'in' by it's spatial relation to the rest of the form (20100320a\_321-322). The position of the month in the year is also reflected in the angles between each set of 10 days (20100320a\_305-306), such that the angles within each mont are distinct: "So like the 20's of March are going in that direction, 20's of August are kind of going in that direction, so they have got angles like that in August. And March ones are like this" (20100320a\_338).

            - So the whole year is like one big kind of like clock type thing (20100320a\_308).


              If you zoom out far away it looks round, but as you zoom in, its jagged because there's a corner at every 10 (20100320a\_310).


              INTERVIEWER: So how do you know which month it is? AB: Erm, because where they are on the clock face. So, February is right at the bottom and August is at the top (20100320a\_321-322).


              drawing\_20100320a\_calendar


              INTERVIEWER: Do you already see a bit of the other month as well or is it just focused on a couple of days? AB: Erm, it depends on what I draw my attention to. I can draw my attention to whatever I like really. September is here, October, November, December, January, February (20100320a\_335-336).

              + -


              relation to month

              - So February. Yeah, so the months themselves are in a circle. So the year goes that way around. So February goes from half past 6 to half past 5 and there's the 12 so August is right at the top, goes from half past 12 to half past 11, etc, etc and January is here (20100320a\_324).


                So the 20's of March I'm seeing them like this, kind of in that direction and they're sort of slightly to my right. Whereas the 22nd of August is kind of up here and going that way and then September begins going in that direction (20100320a\_328).


                Same location with respect to the rest of the year (20100320a\_332)


                INTERVIEWER: But the rest of the year is not always visible is it? AB: I just have a vague sense of its presence. So if I look as 22nd of March being here then the circle of the year is kind of like this. And I know we are in March just because of that position there (20100320a\_333-334).


                INTERVIEWER: So what makes the month distinct from each other? AB: Erm the position on the clock and the direction that the days are going. So like the 20's of March are going in that direction, 20's of August are kind of going in that direction, so they have got angles like that in August. And March ones are like this (20100320a\_337-338).


                INTERVIEWER: And when you zoom in you see the 22nd, how do you know it's the 22nd of March and not August? AB: So the 22nd August is up here and the 20's of August are going in that direction, whereas the 20's in March are kind over here and going like that (20100320a\_325-326).
              + -


              similar to number line

              - INTERVIEWER: When you draw it like that, you don't see a line, you just see the number space differently? AB: ... whatever day I'm focusing on I sort of see that number, but then I am aware of the line around it.  INTERVIEWER: When you mean line, is that metaphorically saying you know what numbers could come next or is it actually you see a line? AB: So a similar thing is happening with the number line, so a number appears and there's like a line down here. And then sometimes I see the numbers kind of like that, standing up on the line (20100320a\_317-320).


                INTERVIEWER: So, “Today is the 22nd.” What happens then? AB: So I see a 22 here. So I think each set of 10 days, each 10 day chunk within the year is kind of, has its own kind place and direction it's going in (20100320a\_305-306).
              + -


              drawing\_20100320a\_March

              - So 1st March. <pause> So the 1st to the 20th of March might all be going in the same line, I'm not sure . Then the 20s are like this. So 1st March and 20th March until 31st March and beginning of April. 1st April, 10th April and then there's 20th April, 30th April. INTERVIEWER: And what about February, where does it connect? AB: So February, <inaudible utterances> 20th, 2nd, so something like this (20100320a\_314-316).
          + -


          time

          - + -


            AB reports that he has a synaesthetic experience of time which is expressed in different forms with "all kinds of clocks" (20100320b\_44). Three explored here include i) a round analogue clock with a white face, a black rim and minute markers, ii) an orange digital clock, and iii) a seperate digital clock with more rounded numbers.     
              
            To explore the clock form, AB is offered "fifteen twenty" as an inducer (20100320b\_2). AB reports "a black like outline of the clock....[with]... the minute markes ... going in towards the middle of the clock from the outside, with bigger ones for multiples of 5... [representing]... the hours... and... the hands of the clock showing three twenty (20100320b\_2-5) (note here that the inducer was given in the 24 hour format but AB offers the time in the 12 hour format).   
              
            The face of the clock is initially described without numbers, however when prompted AB states that he can see "vague ones comming in" if he focuses his attention on them (20100320b\_5-7).  The minute and hour hands of the clock appear consistant and stable as "big black lines" with a rectangular end (20100320b\_9-11). The second hand by contrast is thinner and appears to "flicker in and out of existance" such that AB was not initially aware of it (20100320b\_16-18). There appears to be a relation between the focus of ABs attention and the experience of the second hand such that AB doesn't experience the second hand as "going round" until he focuses his attention on it (20100320b\_20). AB reports that "whenever my attention comes back [to the second hand] it's kind of moved on... There's a fifty five now, sixty..." (20100320b\_22) as to give the impression of motion, although AB does not report that he actually sees any motion (note also that non of ABs concurrents have included any motion).   AB later describes a second appearance of what we can reasonably assume is the same clock form, this time explicitly stating that the clock is "not labelled with any numbers" (20100408377) and the hands are not moving (20100408\_381).

            For comparison, AB is asked wether he can see the time "in a digital watch" (20100320b\_35). The experience which follows is described "like an alarm clock" with a semi-colon flashing between the three and the two [of 3:22] "showing the seconds kind of going" (20100320b\_35-38), and again AB is able to provide additional detail on the "individual bars the numbers are made of" if he focuses direct attention on them (20100320b\_41-42). AB also reports a simultanesous experience of a seperate digital clock on a white page with seconds and a more rounded font (20100320b\_46).

            - I have all kinds of clocks (20100320b\_44)

              + -


              round clock, white face, black rim, minute markers

              - + -


                1st appearance

                - + -


                  INTERVIEWER: So now it's fifteen twenty [15:20]. AB: Yeah. So I just see, so it's kind of a black like outline of the clock and then it's got the minute markes and they are kind of going in towards the middle of the clock from the outside, with bigger ones for multiples of 5. (20100320b\_2-3)... the hours are the bigger ones (20100320b\_5)... Yeah and then the basically the hands of the clock showing three twenty [3:20]

                  - note stim was 15:20, but concurrent was 3:20

                  There's no numbers on the clock. INTERVIEWER: No numbers? AB: No. Well I kind of get vague ones coming in along, round the outside of the clock actually. Yeah, If I can focus my attention on it (20100320b\_5-7).


                  INTERVIEWER: And what does the hand look like? AB: Just kind of big black lines. INTERVIEWER: What do they have at their end? AB: Just like erm a rectangle (20100320b\_8-11).

                  + -


                  INTERVIEWER: So the clock is white? AB: Yeah (20100320b\_13-14)

                  - also on white page?
                  + -


                  AB: Second hand kind of flickers in and out of existence. I wasn't aware of that at all. INTERVIEWER: Does the pointer look the same as the other two? AB: Looks thinner. It's not really going around (20100320b\_16-18)... That line goes around as I focus on it <laughter> (20100320b\_20)

                  - So I don't see it like every second it's just whenever my attention comes back it's kind of moved on. There's a 55 now, 60. So yeah, it's just kind of moving around (20100320b\_22).


                    I wasn't paying any attention to the minute hand, but then when I drew my attention back to it, it was pointing at 3.21 (20100320b\_30)


                    [the second hand] it's kind of like appearing, its kind of flashing (20100320b\_32).
                + -


                2nd appearence

                - AB: Well, I mean a clock just kind of appears here. INTERVIEWER: What in the middle of the room, not against the wall? AB: No. INTERVIEWER: What kind of clock is it? AB: Just a clock with hands. INTERVIEWER: So it's just a brown one? AB: It's kind of just a white face, black dashes for each of the, for each minute. The bigger ones for every five minutes and also the hours. It's not labelled with any numbers on it. It's got the two hands.  (20100408\_371-377).


                  Showing quarter to eleven, sorry.  INTERVIEWER: ... Are the hands moving? AB: No. (20100408\_379-381).
              + -


              orange digital clock

              - INTERVIEWER: Is it possible for you to see the time in a digital watch? AB: So I just saw 3.22 kind of like orange, and it's kind of like an alarm clock. So it's kind of double dots between the 3 and 2 and they are kind of flashing ...  [the flashing] it's showing the seconds kind of going (20100320b\_35-38).


                INTERVIEWER: can you see the individual bars the numbers are made of? AB: If I zoom in close enough, if I pay attention (20100320b\_41-42).
              + -


              digital clock, rounded numbers

              - There's another one with more smooth kind of rounded 3's and 2's over here on a white background, well just kind of on the page. 3 dot dot 22 and then there's a kind of like 10 seconds (20100320b\_46).


                INTERVIEWER: So do you not have two clocks? AB: I have all kinds of clocks <laughter> INTERVIEWER: What do you mean? AB: There's another one with more smooth kind of rounded 3's and 2's over here on a white background, well just kind of on the page. 3 dot dot 22 and then there's a kind of like 10 seconds. INTERVIEWER: Do we have a clock in here? No, unfortunately not. I was just thinking of other variations. Oh what about this, here's a challenge for you, can you do big ben?  AB: Yeah, big ben is over here <laughter> it's showing 6 o'clock though. (20100320b\_43-48)
              + -


              big ben

              - INTERVIEWER: here's a challenge for you, can you do big ben?  AB: Yeah, big ben is over here <laughter> it's showing 6 o'clock though. INTERVIEWER: So do you see the whole clock face? AB: So I see the whole tower, houses of parliament, Westminster bridge. Yeah, I'm kind of standing diagonally across the street from it. Got the whole kind of houses of parliament and traffic going around. <Slow> (20100320b\_47-50).


                big ben is over here ... it's showing 6 o'clock though [not the correct time] (20100320b\_48). So I see the whole tower, houses of parliament, Westminster bridge. Yeah, I'm kind of standing diagonally across the street from it. Got the whole kind of houses of parliament and traffic going around. ... So a whole scene of London. ... It was kind of just a lot there, but kind of taking in. If I was kind transported physically and I was kind of taking one thing after another and being aware of all the different things. ... Yeah, it was like I was there and focusing my attention on different things. INTERVIEWER: What do you see in the street? AB: Erm, road, lanes and some cars going by quite quickly, traffic lights. Feels like I'm kind of in the middle of the road and hovering above the road. INTERVIEWER: Let's say can you stop the traffic? AB: Erm, let's the traffic light is kind of sideways on, so you can't actually see what colour it is. AB: Well there's a kind of a red man, like the kind of pedestrian crossing thing. It's like a red man. INTERVIEWER: Can you make it go green so that pedestrians can go across? AB: I can get the red and the green to be simultaneously, but I can't get rid of the red. AB: Oh now it's gone green. So the red is flickering a bit. INTERVIEWER: Have the cars stopped? AB: Erm, there's a kind of like almost like ghosts of cars kind of going around the corner. There is a car stop there. INTERVIEWER: Can you switch it back so it can go on its home, go on its way? AB: Not that easily. <quiet> AB: So there's a lot of stuff kind of going on there. There's like a red car just stopped at the lights <laughter> INTERVIEWER: But is everybody else taking a different road now? AB: Erm dunno. There's like ghost cars that are just kind going through around the corner. Kind of like an old car. INTERVIEWER: Do you see any people? AB: Yes. People kind of crossing the street. INTERVIEWER: Oh that's why you can't turn over because there's people crossing the street. AB: Yeah <laughter> There's someone waiting to cross, oh with a buggy. <laughter> INTERVIEWER: So, the scene look contemporary? AB: Yeah it looks contemporary. There's a car kind of waiting at the lights, it's a kind of old fashioned kind of car. INTERVIEWER: I mean do people look dressed as you and me would be dressed? AB: Erm yeah. (20100320b\_50-84).
