# Supplementary material for: An extended case study on the phenomenology of sequence-space synesthesia
Source: Front Hum Neurosci. 2014 Jul 3;8:433. doi: 10.3389/fnhum.2014.00433 (PMC4080762; doi:10.3389/fnhum.2014.00433)
Supplement: Supplementary file 1 [file Presentation1.ZIP › Supp Mat 2.ZIP!/Referenced_Interview_Transcipts/AB - 20100215.doc]

# Name: AB - 20100215

# Description: Dialogue between TF and AB on 15/02/2010

# ¶1: Dialogue between TF and AB on 15/02/2010

¶2:

¶3: TF: So the first thing to say that, you know, because I’m not synaesthetic I don’t have anything to work with from my own experience. So I think probably a good place to start would be to first get a description of what it is that you’re experiencing before we go on to how you experience it, both in terms of temporal progression, and in the sensorial registers and so on so at least, just something to work with really, in the beginning and then we’ll build on that. And really all I am doing here is just to facilitate your becoming aware of the situation so all I’m going to be doing is to ask questions and if I think that maybe we’re drifting off the topic I will try to guide you back to the experience in question and so on. And of course, take your time, you know, there is no rush, we have the whole hour and everything, and we’ll try to repeat it maybe over a couple of weeks or so and see whether we can get like more fine grained descriptions by practising together. Right, so maybe to start with, so how, in what circumstances does your experience appear?

¶4: <overtalking – AB agreeing throughout>

¶5: <long pause>

¶6: AB: Yes, we’re talking about the, ahm... the... the calendar or the days of the week or the numberline? Yes I’ve...

¶7: TF: The calendar or the numberline... Well let’s start with the one that’s more familiar. The one that’s more often than the other one? The one that’s maybe more stable in your experience and is easy to work with?

¶8: AB: Oh, ahm... they’re probably the same really <quiet>

¶9: TF: The same? Okay so, let’s start with the numberline then. So under what conditions does the numberline appear in your experience?

¶10: AB: I guess from counting. <questioning tone>

¶11: TF: Okay, say counting, also when I’m counting or just when you’re counting?

¶12: AB: Ahm, if i’m counting in my head.

¶13: TF: Okay. So, if I tell you to count to three, then what do you see?

¶14: AB: I see... I see the, the symbols of the numbers, in the Arabic ordinary symbols, ahm, I see them quite big, and black in colour against a white background. Ahm, the number one... is, ahm, sort of, sort of appearing in the middle of the page, and then number two appears up and to the right of number one. And then number three appears up and to the right of number two. So it’s sort of in a straight line evenly spaced. <slow>

¶15: TF: There’s a sort of progression going one, two, three?

¶16: AB: Yeah.

¶17: TF: Okay so you’re saying on a white background, black ink, rather big letters?

¶18: AB: Yeah.

¶19: TF: Can you say something of where this is being presented to you?

¶20: AB: Where in space?

¶21: TF: Yes

¶22: AB: Yeah, ahm...

¶23: TF: Is it in front of you, or...?

¶24: AB: It’s hard to say, ahm <quiet>

¶25: <pause>

¶26: AB: Yeah, I think I’m projecting a mental image in front of me. Ahm...

¶27: <pause>

¶28: TF: Okay, well maybe we’ll come back to that later. So, the progression stops at the numbers that you have counted, it doesn’t have any continuation, it only figures the ones that you have actually been counting...

¶29: AB: <interruption> yeah, every time there’s a count, yeah, just ahm, yeah. And actually just thinking about how i would see a longer number line, beyond the number ten, that does seem quite different.

¶30: TF: Okay, so what if I tell you right now, count to ten? Just do it and see what happens!

¶31: <long pause>

¶32: AB: So now I’m kind of more looking down on the page <louder>... and the angle is slightly different... it’s kind of... ahm... it’s like the clock has turned a bit more, it’s kind of going off more at, ahm, so if the number one is, ahm, so I’m sort of looking, yeah I’m sort of looking down maybe at about an angle of 60 degrees, and number one is, yes, the right way up. And then the number line is going off at an angle of, err, probably the direction of two o’clock on a clock face. Ahm, and, they’re in a straight line... and evenly spaced up to ten. Ahm, I can sort of see a line going off, ahm, like the continuation of the number line going off. <slow>

¶33: TF: Does it become smaller, the numbers?

¶34: AB: So, well that I see just as a line really. When you tell me to just count to ten I can see the actual digits as far as ten but then I can sort of... I guess as I’m.., I guess as I’m reciting it in my head the number that I’m thinking of appears as a sort of clear digit, ahm, then I can, well I sort of see the number line... well, just the number, just the sort of... well you said the number ten, ahm, I guess that, that appears with a number line behind it and a number line in front of it. And there’s a clear right hand, there’s a bend at the number ten and then the continuation of the number line beyond ten is going to go off, ahm in a different direction.

¶35: TF: Okay, so, in the beginning when I asked you to count from one to three, you said there was black digits on a white background, like a white page, but now I’m understanding that there are only black digits when they’re specifically mentioned. But in between the white page, and the black numbers there’s actually also the other numbers that have not been mentioned but they’re in a different kind of state?

¶36: <overtalking: AB agreeing throughout>

¶37: AB: Yeah, i just see a line just ahm, like a black line... and it’s only when I think of one particular number that... ahm

¶38: <pause>

¶39: AB: Yeah well it gets, yeah it’s a bit, a bit blurry. When you said the number ten, and I was counting to number ten, I never saw the number eleven at any stage, but I did see a nine going off in a different direction. I never saw the digits for eleven.

¶40: TF: Okay. You said there was a bend there?

¶41: AB: Yeah

¶42: TF: What do you mean with, “bend”?

¶43: AB: So... so I just see a line continuing from the number ten like a... like a sort of, like a thick, black line.

¶44: TF: But where does the spacial aspect come into it? Does it have a three-dimensional contour of the line, or is it two dimensional?

¶45: AB: Ahm, it’s two dimensional. Ahm, If I think of how the numbers are laid out, from one to... I’m looking - if I’m looking at all the numbers together from one to thirty it’s perhaps getting a little bit three-dimensional. Ahm.

¶46: TF: So when I say now to you, when you are looking at one to thirty: Twenty. What happens? Does it appear out, is it a distinct number?

¶47: <pause>

¶48: AB: Yes, all the digits, twenty. Ahm, I also see, again I see a... like a... I see a twenty thing like at a corner, ahm...

¶49: TF: And this corner is two-dimensional?

¶50: AB: Yeah, this is a two dimensional corner. So the line coming in... sort of from the left... It’s not really stabilised, the angle of the turn isn’t stabilising. But if you’re approaching twenty, from the teens, it’s a left turn, at number... at number twenty... I see, i see again a kind of line with just the number twenty in the middle of... just written in big. So that’ll occupy more space than the width of the line.

¶51: TF: So, they are bigger than the line, the numbers?

¶52: AB: Yeah

¶53: TF: So you are saying that there is a black line, coming from the left-bottom somehow, and at the place where there’s the numbers, the digits, they are bigger than the line, there’s a bend in that line, and what happens at the bend? It goes deeper, higher up, or does it level off?

¶54: AB: Ahm... I’m looking at it... at the moment I’m projecting it onto the screen infront of me and just... So we’re on that screen.

¶55: TF: So if you look at that screen...

¶56: AB: The precise angle is a bit tricky to say precisely. It’s kind of between... so if the twenties are going off at twelve o’clock, and the teens are approaching from either sort of, somewhere between seven and... <slowly>

¶57: AB: I guess between seven and eight o’clock sort of stabilising a bit. Now I was going to say between seven and nine o’clock, I don’t think it is, it’s not as sharp as a right angle, it’s a bit, it’s a, it’s like a... yes the number of degrees you’re turning is, err, somewhere... somewhere between 30 and 60 degrees probably. <slowly>

¶58: TF: Downwards... <affirmative tone>

¶59: AB: Umm, it’s 30 to... you’re turning 30 to 60 degrees to your left, so when I put it onto a screen in front of me, it looks like the umm, the number 20 is in the middle of a clock face, and 21, 22, 23 etc, well it just appears as a line...

¶60: TF: Going upwards? <interrupts)

¶61: AB: Yep, going upwards, and the teens are coming in, in a straight line from, err, somewhere between 7 and 8 o’clock

¶62: TF: Ah, okay

¶63: AB: <inaudible word> 17, 18, 19, 20 and 20’s in the middle.

¶64: TF: And then it goes straight up?

¶65: AB: Yep.

¶66: TF: So, if I said to you now, 15, is this angle being placed at the front of 15 and all the numbers are going straight up?

¶67: AB: No, it’s a bit different with 15, erm

¶68: TF: So it depends on which number it is?

¶69: AB: Yeah.

¶70: <Pause>

¶71: AB: You say 15, I’m actually seeing more digits now. I sort of see 14 and 16 and 17 errr...

¶72: TF: So, do these additional numbers also stick out over the line, and do they then eventually fade into the line, as you move away?

¶73: AB: Errm...

¶74: TF: So if I say 15...

¶75: <Pause>

¶76: AB: Yeah I only really clearly see 13, 14, 15, 16, 17 and err... again they’re going, so... in a ‘ss’... now I’m sort of... now I’m sort of looking down on, yeah, if they’re on a page they’re... 14 is up and to the right of 13, 15 is up and to the right of 14, and so on, it’s kind of a straight line, going in. It’s at a more gentle angle though than the 1,2,3 before, it’s much more, err, close, it’s closer to the horizontal, err, it’s definitely not a horizontal line, so, ahm...

¶77: TF: So what happens with 16 and 17, are they continuing that line or are they going upwards?

¶78: AB: They’re going, yes they’re all going upwards. It’s a straight line, it’s erm

¶79: TF: After 15 it’s a straight line. <questioning tone>

¶80: AB: 13 to 17 is a straight line...

¶81: TF: Oh, okay.

¶82: <pause, someone exhales>

¶83: AB: Yeah <barely audible>

¶84: AB: A straight line, evenly spaced. I’m seeing all of the figures

¶85: TF: Okay, so if I say, 25, do you see all the figures now?

¶86: <Long pause>

¶87: AB: Now the line appeared, so I saw 25, didn’t see any other digits very clearly, hard to stabilise... but the line is going in a different direction now.

¶88: AB: The line is, erm, so 24 is down and to the right, yeah, down and to the right of 25 and 26 is up and to the left.

¶89: TF: Okay.

¶90: AB: A different direction!

¶91: <Both laugh>

¶92: TF: Okay, well let’s try a few other numbers, just to see whether this is a very variable thing, or whether it has to do with certain ranges, it might be something to do with like between 10 and 20 is one thing and so on.

¶93: TF: Now if you go back to the beginning, and I say, five...

¶94: AB: Yep

¶95: TF: What do you see?

¶96: AB: So I see the number five,

¶97: <long pause>

¶98: AB: And then I saw a line, so now the numbers are sort of erm, so there’s, so there’s a line, there’s a line there, erm, and the number five is sort of erm, suspended, yeah it’s kind of suspended above the line.

¶99: AB: This time the line is going from down and to the left, and the higher numbers are up and to the right. There’s more of a sort of 45 degree. Err, and a sort of horizontal...

¶100: TF: Okay, so this is the same angle, as if I said to you, “count, from one to three” <questioning tone>

¶101: AB: Yep. No, slightly err, slightly more towards the horizontal.

¶102: TF: More towards the horizontal... <confirming tone, as if writing it down>

¶103: AB: And...

¶104: AB: I’m having trouble stabilising the digits. <animated> I keep seeing the number six the wrong, wrong side of number five. Although now it’s kind of, err, so it’s kind of jumping around a bit, the actual digits. Erm, number five is staying still, somehow the six is erm, popping back to where I kind of know four should be but. And with seven, the other side but then, but then it’s kind of moving around a bit.

¶105: TF: So some digits are jumping, but some remain stable?

¶106: AB: Erm,

¶107: TF: Or is it generally a bit unstable? Do some digits exude more of a stable presence, than others, is it like that?

¶108: AB: Ahm, I think I ... at one point I was seeing six, five, seven, and then when six jumped err, to where it should be, the seven kind of disappeared.

¶109: TF: Just coming back to the bend that you were talking about, and to the higher digits, in this case when I say five, you didn’t say anything about ten, there was no ten?

¶110: AB: No.

¶111: TF: Okay.

¶112: AB: Err, there is only a bend at, a bend at ten, and at 20,

¶113: TF: Oh, okay. So if I said to you: “15”, then it’s a line going from the bottom left, to the top right, but without any bends, and you see extra digits next to the line, the number?

¶114: <AB agreeing throughout>

¶115: AB: Yes, I see erm, yeah I can see 14 and 16, and 17.

¶116: TF: Okay

¶117: AB: 15 comes into place first, and then 14 and then 16, erm 17 is pretty vague. Unless I let my mind wander up the line. But if I sort of, if I... if I... centering my attention on 15 then I get fleeting images of the 14, 16, maybe 17. Even slighter, 13.

¶118: TF: So...

¶119: AB: <cuts in> I guess it depends on how long I let my attention wander as to how far up and down the line I can ‘grip’. So just, the process of talking about 13, 17, 18 popped up, err, for a moment, and as 19 just popped up and 20 there. But if I bring my attention back to 15, it won’t go any further.

¶120: TF: So I think that’s enough about what’s going on. Maybe later I can try to see whether I can probe a little bit deeper into the temporal structure of this experience. So when you say 15 popped up, what do you see?

¶121: AB: Number... So the first thing is the digits, 15 quite big, and black, big black, ink, against a kind of white background.

¶122: <sound of chair scraping>

¶123: TF: So, what is before this? Is there some sort of expectation to it?

¶124: AB: The expectation what I’m going to see... <questioning tone>

¶125: TF: Well you said there is the 15, in big black letters, with the white background. So before I say the numbers, is there just the white background?

¶126: AB: Ahm, don’t know...

¶127: TF: So if I say 15, what happens?

¶128: AB: So possibly, erm, there’s a white background and the number revealed itself from the bottom up, the...

¶129: TF: So there’s a movement?

¶130: AB: But I’m not sure, I need to kind of focus on that a bit more to really...

¶131: TF: Okay, so let’s try with another number, if I say to you, “five”?

¶132: AB: If anything that appeared from the... started at the top left and then was revealed diagonally, coming down...

¶133: AB: There’s a little bit of colour in there as well, <more animated>

¶134: AB: As kind of, erm, sort of, like little sparks of sort of green and umm... purple I think, as it was, erm, sort of coming into view, but then, it kind of stabilises as black.

¶135: TF: So, during the initial phase, when I said five, there was a movement from the top left corner, and it moved down to the place on its line?

¶136: AB: So I didn’t see a line when you just said five, actually, this time.

¶137: TF: It was just the number five coming in from the top left corner?

¶138: AB: Yeah <over TF saying ‘corner’>

¶139: <pause>

¶140: AB: So it seems like the first thing is, is the big number five and then it kind of... moves into position, kind of floating above the line.

¶141: TF: So is the line already there when I say, “five”?

¶142: <pause>

¶143: AB: I think the line appears a little bit after, so the, the digit gets constructed first, and then, and then... and then the line comes into view

¶144: TF: Okay, so you said there was a little bit of colour, do you know where this colour appears?

¶145: AB: Ahm...

¶146: TF: Can you see the colour?

¶147: AB: Well it’s just a little splashes like around the, the bend, the curvy sort of bit at the number five. It’s little, little splashes of... almost like, like someone’s spilled a little bit of paint on there. <slow>

¶148: TF: And then when the five starts moving to its proper location the colour disappears, or does it stay?

¶149: AB: Yeah <over TF saying “or”>

¶150: AB: Yeah, it disappears.

¶151: <pause>

¶152: TF: Okay, so if I say, “five” to you, do you see where the line comes from?

¶153: AB: It just appeared, ahm...

¶154: <pause>

¶155: AB: I didn’t notice what order it appeared in, it just seemed to appear. Err, there’s a little bit of err, like a green background to the line, when it first appears.

¶156: TF: <agreeing sound>

¶157: <pause>

¶158: TF: And then the green fades into black, or what happens?

¶159: AB: Ahm, the line itself is black, so the background... Couldn’t really pay so much attention to the background...

¶160: TF: You mean the background behind the line?

¶161: AB: Yeah, it seems a bit like it’s sort of suspended in, in mid-air, the line.

¶162: TF: And the shadow is green, did you say, or...?

¶163: <pause>

¶164: AB: Ahm.

¶165: TF: Just recall for the experience, if I say “five”.

¶166: <long pause>

¶167: AB: I just see a line going from... it’s kind of blurry the background, it’s kind of the line suspended in air.

¶168: TF: But the line itself is well defined, or is it also blurry?

¶169: AB: Err, it’s quite, quite sharp.

¶170: <pause>

¶171: TF: Okay, so if I say “five”, does the number appear suspended?

¶172: AB: It starts off right in front of me as a... as like on a white background, kind of, like on a rectangular sheet of paper, and filling good proportion of the paper. Like as if there’s only room for that one digit. Sort of centred, as if...

¶173: TF: A big number filling out the page.

¶174: AB: Yep. With a good margin around the edge, but no room for anything else...

¶175: TF: Yep.

¶176: AB: ...On the page. And... and then this kind of drifts, and then this kind of melt...like the the background, the white background kind of melts away and the digit kind of <fast, animated>

¶177: AB: Ahm, starts moves, moves downwards, and then at some point a line appears and the number’s floating, erm kind of above the line. The line is coming from the lower left of the field and going towards the upper right. And I’m... I’m a... sort of, from where I’m centred, I’m looking, I’m looking sort of, it’s kind of ahead of, ahead and down from where I’m there, from where my ego’s located.

¶178: TF: So you’re looking down, on the page and the number five fills up most of the page, and then you said...

¶179: AB: I’m looking, I’m looking erm, directly at the page. The page is horizontal in front of me but then the number line is kind of down.

¶180: TF: So the number line is actually below the page, or does it just come after the page has melted away?

¶181: AB: Ahm,

¶182: <pause>

¶183: AB: So I’m not quite sure of the, the order of things but erm... Sort of a similar thing sort of... I, I can definitely see it being kind of located below where the page was.

¶184: TF: <agreeing sounds>

¶185: AB: But I don’t really know how the page melts away.

¶186: TF: Okay. So if I recall the sequence of events, and correct me if I’m wrong. So if I say “five” to you, the first thing that appears to you is a big number five on a white background.

¶187: AB: Yep.

¶188: TF: Then the white background seems to melt away,

¶189: AB: Yep.

¶190: TF: And while, or after it’s melting away, there’s a number line below where the white page used to be and the five moves down onto its location on that line?

¶191: AB: So this time you just asked, you just discussed that process, I was kind of, err, aware of, of the melting away of the... of the white background, and was kind of like the melting process. Erm, it seemed to melt away more at the bottom first, and then kind of fizzled away at the top.

¶192: AB: And then the backgrounds are very unstable, it’s kind of, erm it’s kind of lots of different colours sort of popping up... erm... but yeah, it’s sort of generally a bit, a bit misty.

¶193: TF: So, when the background melts away, and all these colours appear on it, it kind of melts away at the bottom first, and then at the top, it kind of like fizzles away, what do you see then?

¶194: AB: Well now I’m just seeing a five, black five against, kind of unstable background, like almost sort of like psychedelic. And then the line is just there underneath it. So I haven’t really seen the five move away now.

¶195: TF: It’s staying, the background?

¶196: AB: Yeah, and then it’s just there above the line.

¶197: TF: So it’s suspended above the line would you say?

¶198: AB: Yeah, yeah. Yeah so now I’m kind of seeing the five right in front of me but the line is as I said it’s kind of, erm, yeah it’s below, I have to look down, down in front of me to see the line, the number five is right in front of me.

¶199: TF: But do you see any connection between the five and the line?

¶200: <banging sound on tape>

¶201: AB: It’s just hovering above it. So there’s no, there’s no mark precisely where on the line that number five should be, it’s just...

¶202: TF: Okay.

¶203: AB: It’s just floating above the line.

¶204: TF: So if I said to you: “four”

¶205: AB: Yep.

¶206: TF: How does the scenario change?

¶207: AB: So... so I was kind of focusing on the five, when you were s..., when you said four, so I kind of erm, erm, kind of made me shift my attention, down the line, down, down and to the left down the line, and... sort of got a, getting a... flashing of the number four, appearing sort of at the bottom left of my field of vision there it’s just sort of on the edge, and it’s like. It’s like when I’m looking at the external world and I’m trying to paying attention to something that’s erm that’s sort of just on the edge of my visual field. The four - you can only just see it but there’s a black, you can see there’s a number four there, there’s a black four quite thick ink again, and again just kind of above the line, above the... the... the number line.

¶208: TF: Okay, just to make sure I got your sequence of events, when I said “four”, what you did is you shifted your attention down to the number line, and at the position where the four should be, a four appeared that was suspended above the line at that position.

¶209: AB: Yep, yeah. It’s almost like I’m seeing like a kind of like a picture frame or something around it there. There’s like a corner of a picture frame and the four is kind of, erm, it’s kind of flashing a bit and it sort of sometimes kind of the ink, the, the sort of black in the number four is kind of erm, having to go over the, over the picture frame, even though the line is kind of going underneath the picture frame. Ahm...

¶210: TF: So the number is framed, and the frame is below the number, and hovering above the number line?

¶211: AB: Yep, Yeah.

¶212: TF: What does the frame look like?

¶213: AB: It’s kind of made of like, wooden, err, brown.

¶214: TF: It’s too small for the number, you say...?

¶215: AB: It’s too small for the number four, it’s not quite fitting.

¶216: TF: So the number is overflowing on to the sides you say?

¶217: AB: Yeah. Yeah, not really stabilising, kind of flashing a little bit.

¶218: TF: What did the flashes look like?

¶219: AB: Just the number four disappearing, it seems like there’s a clash, like the number four, like there’s not really room for it. Umm, umm, I’ve centred it on number five and so there’s not really room for the number four there. But,

¶220: TF: Ah okay.

¶221: AB: But I’m focusing my attention on it and so it’s kind of, kind of appearing. But the number five has kind of moved off, it’s kind of seems to be... it doesn’t seem like there’d be room for number six at all, in this frame.

¶222: TF: So the five, is also on the frame you say?

¶223: AB: So we <unintelligible>, there’s still room for the number five. Five is sort of, the upper right hand corner, not too close to the edge, then the four is sort of in the, bottom left corner and kind of not really, there’s... Yeah, there’s not, not really enough room for the four, it’s kind of yeah, it’s go...going over, over the edge.

¶224: TF: But they’re both on the same frame hovering above the number line, or are they in different frames?

¶225: AB: Err, they’re in the same frame.

¶226: <pause>

¶227: TF: So if I said four to you, and the frame appeared, the frame could come past both of the numbers?

¶228: AB: Yep.

¶229: <pause>

¶230: TF: So you...

¶231: AB: It’s kind of moved a little bit now, now erm cause we’re talking more about four than five, so it’s kind of I feel it... My, my perspective has changed a little bit and it’s kind of, I’ve got four and five are fitting a bit more clearly into the frame now.

¶232: AB: And I’ve still got a bit of a “shower”, still flicking backwards and forwards between the two, err, so I’ve got a little, glim...glimmer of the previous image of four kind of not fitting in the frame, but erm... Four’s becoming a little more centred now so I had a, I had one glimmer of four and five fitting in the frame, now I’m kind of getting three, four and five fitting in the frame.

¶233: TF: Okay.

¶234: AB: Ahm...

¶235: <pause>

¶236: TF: And still the wooden frame hovering above, or has the frame changed?

¶237: AB: Ahm, so there’s a line now in... of a framed line, with erm... there’s not really stabilising, you’re getting a different a, different erm, number... different number of digits appearing in it like a kind of err, started a bit of motion there and so I’ve kind of, erm, got as far as, well I get different images popping up so one moment there’s two three, four, five, six, seven in the frame and the next there’s, I’ve got number one appearing and I’ve the whole number line shrinking as I zoom out, and and just seeing a straight line going from the bottom left, to the, top right. The line is hitting the left hand side of the frame, so kind of starting, ahm, near the bottom of the left hand side of the frame, and then, hitting, hitting the err, hitting the top of the frame which is slightly to the left of the top right hand corner.

¶238: TF: So just to follow you in what you were doing there, you said you zoomed out

¶239: AB: Yeah.

¶240: TF: Ahm, can you explain to me what you were doing, when you were zooming out?

¶241: AB: Erm, They’re just like I... I... erm, so we talked about four and five being sort of centred in the frame, four, four in the lower le... lower left, and five in upper right, but then just as in the erm, whilst I was talking about that erm, my attention was wondering “ well where’s three and two, and six and seven” and when I started thinking about that then that caused the motion of the thing to zoom out and so then two appeared in the lower left and it went as far as erm... like every instant there’s a different set of numbers in the frame, so one minute there’s two, three, four, five, six, seven, they’re all going in the same, the direction’s staying the same.

¶242: TF: Okay, so If I understand you correctly, what happened is you were thinking about four and five, and then your attention drifted to more numbers?

¶243: AB: Yep.

¶244: TF: And as your attention drifted towards these extra numbers, the experience was of the frame with the numbers zooming out, and therefore making more space for the numbers, and then what happened?

¶245: AB: Yeah making more space for more numbers to fit in the frame.

¶246: <pause>

¶247: TF: So, when you say you saw the zooming out, did that mean that the screen that you were seeing moved backwards, or did the numbers become smaller, or what do you mean with zooming out?

¶248: AB: Yeah the numbers became smaller, so numbers... the frame stayed the same size and the numbers become smaller.

¶249: <pause>

¶250: AB: And then when I put the number one in, it kind of shrinks a lot, and then the number one... and then everything becomes kind of really small and... and I can see as far as about the number nine, I think. Forming, so the line is going in the same direction but, erm, the digits one up to nine don’t, don’t reach the top of the frame, and they’re still somewhere in the middle. And then when I focus my attention on sort of number, number... where the, where’s the rest of that line gone, that’s when I see the right hand ten, the number ten, and then I see it go off at... so then from ten onwards where, where the teens would be, I mean I only see it as a line, that is going a horizontal line, from ten onwards, and then it’s hitting the right hand margin.

¶251: TF: So it’s the number ten, where the line is bent?

¶252: AB: Yeah.

¶253: TF: And then it’s hitting the margin of that frame?

¶254: AB: Ahm, so at one point it hits the margin, if I try and erm, if I then look for where the number 20 is it keeps zooming out, and it doesn’t ever reach the edge of the page. So then number twenty it’s sort of goes off, ver... starts going vertically again.

¶255: TF: So do you see any specific numbers when you are seeing this?

¶256: AB: Erm...

¶257: TF: When you are seeing the line, how do you know it’s 20, and ten?

¶258: AB: Erm, it’s from past experience because I’ve visualised this lots of times. I’ve never been aware of the frame before, but the shape of the line is always the same and the, the corners are always in exactly the same place.

¶259: TF: So what is the relation of this frame that you are seeing, with the white background that you were describing before? Do they appear together?

¶260: AB: Yeah, they appear together, it’s almost like the, the white page is sort of... is the... the frame, and its... there’s a... glass or something covering up the paper so there’s kind of a slight shininess... to it.

¶261: TF: So the frame is not hovering above the white page it’s kind of covering the white page?

¶262: AB: Yeah.

¶263: <pause>

¶264: TF: Alright, I think we can probably leave it there for this time.

¶265: AB: Yeap.

¶266: TF: Okay, thanks.
