# Supplementary material for: An extended case study on the phenomenology of sequence-space synesthesia
Source: Front Hum Neurosci. 2014 Jul 3;8:433. doi: 10.3389/fnhum.2014.00433 (PMC4080762; doi:10.3389/fnhum.2014.00433)
Supplement: Supplementary file 1 [file Presentation1.ZIP › Supp Mat 2.ZIP!/Referenced_Interview_Transcipts/AB - 20100222.doc]

# Name: AB - 20100222

# Description: Dialogue between TF: and AB: on 22/02/2010

# ¶1: Dialogue between TF: and AB: on 22/02/2010

¶2:

¶3: TF: So, for this week, I think a good start would be to just go to the type of experience we were looking at last week, just to see whether we can find the same kind of experiential aspects that we reported on last time and then we’ll go from there and explore a few new areas. Okay to begin with, we talked about the number five, so can you visualise the five? What do you see?

¶4: <AB: agreeing throughout>

¶5: AB: So I see a... erm, a black number five in front of me... against a white background. <slowly>

¶6: TF: Yep.

¶7: AB: Erm... and if I think a little bit more then I see a... see it hovering above a line that’s sort of below me like that, sort of where I’m pointing, so looking at an angle away from me, going away from me and from left to right.

¶8: TF: Okay so there’s a black five, hovering in space in front of you?

¶9: AB: Yep.

¶10: TF: In front of a white background, and below this, there’s a line, a black line?

¶11: <pause> <AB: agreeing throughout>

¶12: TF: Okay, so keep focus on the five, if you can, and let’s try a little experiment.

¶13: <AB: agreeing throughout>

¶14: TF: If you focus on the five, and what are you aware of in the periphery?

¶15: AB: In the periphery, ahm...

¶16: <pause>

¶17: AB: I’m not aware of very much in the periphery.

¶18: TF: Okay.

¶19: AB: Not very stable. The five itself isn’t very stable, and in terms of err, colour, and shading of it... it seems like there’s always kind of a... a black, sort of black outline of the number five, and then err, shading can be varied, and even have patterns on it at times.

¶20: TF: So the outline stays the same, but the filling colour changes?

¶21: AB: Yeah. Yeah it’s not staying, staying, staying stable, stable black.

¶22: TF: Okay, what is the relationship between the outline and the background?

¶23: AB: The background’s further away.

¶24: TF: Can you describe it?

¶25: <long pause>

¶26: AB: I think it mainly is green, and there’s appearing a kind of a wall...

¶27: TF: The background...

¶28: AB: So, so you can sort of see a floor underneath the five, and then there’s kind of a wall... ahm.

¶29: TF: What does the wall look like?

¶30: AB: It looks like a plain, plain sort of... fairly plain...

¶31: <pause>

¶32: AB: There’s light perhaps against the wall, so the five’s kind of, umm, kind of in the middle of the room and then there’s... can kind of see the floor and the ceiling, and then there’s kind of... can’t see the sort of left end or the end hand of the wall, it’s kind of extending, out into space

¶33: TF: What colours did you see?

¶34: AB: There’s kind of a greeny, green-brown, orange, sort of somewhere in there, it’s not... it’s not that stable.

¶35: TF: <agreeing sound>. So, the background is behind the five, it’s a kind of greeny-browny orange and it connects with a floor, at the bottom?

¶36: AB: Yeah.

¶37: TF: What does the floor look like?

¶38: AB: Kind of the same as the wall.

¶39: <pause>

¶40: TF: And if you focus on the wall and the floor, and try to keep your focus there, how does the periphery look? You said it’s undefined, or does it go off into the distance?

¶41: AB: Yeah, so I’ve sort of got the number five to the right of my focus now, and kind of in front of my focus. Possibly a kind of whiteness at the end, at the left hand end of the wall. It just kind of fades into this kind of whiteness.

¶42: TF: Okay, well this brings me onto my next question. When I first said five to you the first thing that you reported was a black five at least in the contours, against a white background. But then you reported that it was green, brownish, and now we’re going back to the white. How do these things relate?

¶43: <AB agreeing throughout>

¶44: AB: So the white... So... so the five on a white background appears first, and then the white kind of melts away. And then the five is kind of floating in this room.

¶45: TF: So, is the white a spacial aspect, like the wall?

¶46: AB: The white is... so it’s as if the five is just written on a sheet of paper, but then the piece of paper melts away.

¶47: TF: So the wall is behind the white sheet? In spatial terms...

¶48: AB: Yeah.

¶49: TF: Or is it coinciding...

¶50: AB: Yeah, it’s behind <at the same time as TF saying the above>

¶51: <pause>

¶52: TF: So, when I say five to you, and the first instance the black five appears on the whiteness.

¶53: AB: Yeah.

¶54: TF: Do you really see the number line at this point?

¶55: AB: Not initially.

¶56: TF: When does the number line appear?

¶57: AB: Just after the, err, I think just after the white, sort of paper thing has melted away.

¶58: TF: Okay, so let’s just try to do it slowly, and then have a look if you can see what happens. If you think of the five, what happens?

¶59: <pause>

¶60: AB: So five against a white background, the white background kind of melts away, and then it’s floating above a black line, which is going from bottom left to the top right, kind of looking at the... <slowly>

¶61: AB: The line is looking down from where I’m... it’s kind of along the floor. <faster>

¶62: AB: And then there’s sort of a wall behind, this colour that I can’t quite make out exactly what colour it is. <slowly>

¶63: TF: Okay. So what is the lines relationship with the floor?

¶64: AB: The line seems to be on the floor.

¶65: TF: Directly on it?

¶66: <pause>

¶67: AB: Yeah.

¶68: TF: So the line is flat? Let’s have another look at the sequence perhaps.

¶69: AB: Mmm <agreeing sound>

¶70: <pause>

¶71: AB: It’s hard to tell... it could be sticking up above it, like as if it’s erm, like a long... like it has height coming out from the floor, it’s quite vague.

¶72: TF: Is it vague because it’s spread away, or is it intrinsically blurry?

¶73: AB: Ahm,

¶74: <long pause>

¶75: AB: Well there’s definitely a sort of... definitely a steady angle ahm, it’s not really moving around. Yeah I would say it’s kind of sticking up above the floor. So it’s kind of so the bottom, the bottom of it is resting on the floor, and then... there’s almost like a very, long rectangle, black that’s lying, sort of standing up on the, on the floor.

¶76: TF: Okay. Maybe we can come back to that later. What really interests me is the unfolding of the sequence. So I’m not quite sure yet how the sequence gets initiated. So If I say to you, “five” what happens in the very beginning?

¶77: <AB: agreeing throughout>

¶78: AB: Big number five...

¶79: TF: Is this appearance automatically triggered by my saying it, or are you thinking of the number five at the same time, is there any kind of mental gesture involved, or feeling, any other sensations in your body?

¶80: AB: It seems to get triggered by you saying it, <unintelligible word>.

¶81: TF: Okay, if I say it again now: “five”.

¶82: <pause>

¶83: AB: Quite vague, I’ve got a sort of jumbled up collection of images there’s the five, and, sort of sometimes on a white piece of paper, sometimes the white piece of paper is only partially there. And it’s partially... it sometimes seems like the bottom half sometimes goes first. And then sometimes, sometimes it’s sort of in that room that I was describing

¶84: TF: Can you tell any difference between the two, under what conditions does one happen, and the other?

¶85: AB: Yeah, it’s hard to bring it backwards, it’s hard to recreate the initial, th-the full white, background with the five. I can get little bits of it, maybe in the top left, but to totally bring back the whole number five... well now it’s kind of coming back. But erm, as soon as I bring it back it kind of gets stripped away again and the five is floating above the line.

¶86: TF: So it seems to have some sort of automatic sequence of events as soon as it’s brought out, is this the case, or?

¶87: AB: Yeah.

¶88: TF: So it’s actually my voice that triggers it.

¶89: AB: Yeah

¶90: TF: So when I say five, do you immediately see the five?

¶91: AB: It seems to take a little while.

¶92: TF: Okay. So maybe we can repeat this experiment with the number that you emailed me, it had interesting properties, to see whether we find the same sequence of events, or different ones. So the number was 56? So if I say 56, what do you see?

¶93: <AB: agreeing throughout>

¶94: AB: So I see, see the number 56, written on a white background. So there’s kind of a big err, ahm, so it’s got to be in a smaller font now, to fit on this, err page, the page is err, sort of upright, portrait, err orientation, and so the... so there’s sort of more white at the top and bottom now because the five and six occupies the whole width of the page, pretty much.

¶95: TF: You said it’s a smaller font, but there’s more white?

¶96: AB: There’s umm, the-the-there’s much more white at the top and the bottom than in the middle, because the five and the six are occupying pretty much the whole width of the page now.

¶97: TF: More white, the top than the bottom?

¶98: AB: Yeah. No, there’s equal amounts at the top and the bottom.

¶99: TF: Are you saying there’s more white at the top and the bottom in relation to the middle?

¶100: AB: Yeah, yep.

¶101: TF: So when I said 56, you said the font was smaller, how do you know that?

¶102: AB: Because if the... if it had been as big as the number five, they wouldn’t have fitted, there wouldn’t have been enough err width on the page for them.

¶103: TF: So... the page stays the same size in both cases?

¶104: AB: Yes, I think so.

¶105: TF: What if I just do them sequentially... “Five” “56”

¶106: <pause>

¶107: AB: I get too much stable... stability there. It seems pretty much how I described. It seems like the 56 is err actually shorter aswell, so there’s more, more white at the top. But that makes sense doesn’t it, because ahm, if... if the 56, if the five, the five and the six in 56 had been as tall as the, ahm, as the single number five, then it wouldn’t have... there wouldn’t have been room for it. So... so they’re scaled down in order to fit the two characters on the page.

¶108: TF: I mean they could have been scaled only in terms of width rather than height.

¶109: AB: Yeah but they’re scaled down so, so you have to scale down the width in order for them to fit where erm, into the width of the page, and in so doing it seems like that the height has been scaled proportionately, thus giving you more room at the top and the bottom so they’re kind of in the middle of the page.

¶110: TF: I think it would be interesting to look into the transition of these two numbers, but maybe we can focus on the 56 for now, and we can explore a little bit more about this number. So if I say to you 56, can you tell me the whole progression that you see?

¶111: <AB: agreeing throughout>

¶112: AB: So I see these two numbers on a... on a white page.

¶113: <pause>

¶114: AB: Eventually they appear against a kind of a number line, kind of going like this there’s a fifty there and sixty here.

¶115: TF: So...

¶116: AB: <interrupts> There’s a like a little gap in the number line above where 56 is sort of hovering...

¶117: TF: Okay. Can you describe the gap?

¶118: AB: Just a gap in the line. Seems like you could probably just about squeeze those two numbers five and six into that gap.

¶119: TF: What do you see around the line?

¶120: AB: It’s kind of on a floor... ahm... the floor is not so stable I can’t tell if it’s kind of wooden, or... exactly what colour it is...

¶121: TF: And if you keep focus on the 56, now that your awareness expands to include some of the surrounding area, do you notice anything?

¶122: <long pause>

¶123: AB: So... so I’m seeing sort of bit, bit... bits and pieces of the number line coming in, I’m seeing sort of an extension of this floor, so it’s almost like at 60 it starts sort of going up, and away from me... ahm, so it kind of never quite hits the sort of wall at the back.

¶124: TF: So there’s still the wall there in the back?

¶125: AB: Yeah.

¶126: TF: Can you see what colour the wall is?

¶127: <pause>

¶128: AB: Kind of an orangey browney, greeney colour. I should mention I’m red-green colour-blind so, I don’t know if that...

¶129: TF: How tall is the wall?

¶130: AB: The wall kind of fades away into kind of whiteness if I kind of look up, and the number-line kind of disappears into that whiteness.

¶131: TF: Is it the same kind of whiteness that you see initially?

¶132: AB: Erm, it’s more of kind of like an infinite whiteness.

¶133: <pause>

¶134: TF: Can you see why there’s a gap in the line?

¶135: AB: Why there’s a gap....? It’s kind of like a marker for where the 56 should go.

¶136: TF: So you have a sense that this is where the 56 belongs?

¶137: AB: Yeah.

¶138: TF: Can you describe that sense?

¶139: AB: Yes, I can lower the two numbers and they sort of fit nicely in that gap. So sort of facing a different way, cause the lines kind of like this, and then you’ve got five and six are kind of standing there, vertically, and then so you’ve got the line, is here, and here, so, it’s just to give a bit of space for those two numbers to breathe.

¶140: TF: So when I said to you five, and you recall the experience of the five, there is no gap?

¶141: <AB: agreeing throughout>

¶142: AB: No, no gap for five... hovering above a line, kind of a tighter space there, the five is occupying.

¶143: TF: How can you tell?

¶144: AB: It’s kind of hemmed in, it’s kind of like touching the ceiling of the... pretty much touching the ceiling of the room that it’s in. This is more like a corridor.

¶145: TF: Okay, so the five is touching the ceiling?

¶146: <Banging sounds on tape>

¶147: AB: Erm, can’t quite tell from where I’m sort of centred.

¶148: TF: How do you know there’s a ceiling?

¶149: AB: Cause if I look slightly away from the five, then I can see, see the top of the wall. So it’s kind of a wall here and then there’s a ceiling coming this way, ahm... I think looking up, the five isn’t quite touching the ceiling.

¶150: AB: Now I look up, this five is pretty big, it’s kind of like that. Big black, number five.

¶151: TF: Is there any front to this corridor?

¶152: AB: Front? Ahm, there’s the left hand end of it is kind of white... I think the right hand end is white as well and then I’m kind of standing on one side of the corridor, looking at the other side, so I don’t know what happens behind me.

¶153: TF: Did you say you were inside...

¶154: AB: <interrupts> It looks like a corridor because if I look down there it’s kind of, yeah I do see the wall, it’s kind of behind me.

¶155: TF: So, you’re inside the corridor?

¶156: AB: So I’m inside the corridor.

¶157: TF: Or are you looking in from the outside?

¶158: AB: Ahm, seems like I’m probably against, pretty much against... against the wall that’s behind of me.

¶159: TF: How do you see yourself in the corridor? In what way are you situated in there?

¶160: AB: Ahm, I feel a bit crushed, like there’s not really room for me to stand there, and there’s just, somehow I’ve sort of just got my head in there.

¶161: TF: Can you...

¶162: AB: So it’s almost like all the walls, you can kind of melt through them.

¶163: TF: What do you mean when you say you can melt through the walls?

¶164: AB: So I kind of feel like my head’s sort of sticking up through the floor of this corridor and there’s not really much room in there for much more than my head

¶165: TF: So would you say the numbers are as big as your head

¶166: AB: No, bigger than my head. It’s kind of, it’s big, big five.

¶167: TF: So, could you move in this space?

¶168: AB: So I can move, ahm. Yeah I can bring myself along the corridor. There’s the number five, appearing at the other end of the corridor now.

¶169: TF: Sorry can you repeat that?

¶170: AB: So I’ve kind of moved along the corridor and the five is now to my left.

¶171: TF: So in what direction did you move?

¶172: AB: So I moved to my right, my head’s kind of, just kind of in the corridor.

¶173: TF: Okay.

¶174: AB: Kind of along the sort of... one wall. So I kind of see across the width of the corridor. Still this kind of undefined colour, there’s kind of a yellowish light kind of permeating it.

¶175: TF: Permeating what?

¶176: AB: The corridor But I still can’t tell what colour the walls and things are... kind of orangey reddy greeney colour.

¶177: TF: How has to appearance of the five changed, in relation to this movement?

¶178: AB: The five has got smaller now, it’s kind of in the distance. But it’s still got the same orientation, it’s facing a wall that my head is sort of stuck in, erm, and sort of fills about three quarters of the height of the... the corridor.

¶179: TF: And so you can see behind it now, or

¶180: AB: I can’t see behind the five.

¶181: TF: Okay so just coming back to the movement, can you move back again into the original position?

¶182: AB: Yes, it’s a fairly sort of instantaneous movement. I don’t really know how I’ve got there. Just kind of wiggled it I guess.

¶183: TF: Once again, let’s try it again. So if I say to you, “move to your right”, what happens?

¶184: AB: Yes I’ve already done it before you said that.

¶185: TF: <laughing> Okay!

¶186: AB: Okay now I’m back in front of the five.

¶187: TF: If you just go back and forth a few times, do you notice anything?

¶188: AB: Uh, it’s very quick. Just kind of moved my, it kind of seems like I'm just sort of, it kind of goes in an instant as I move my eyes.

¶189: TF: So if you move your eyes slowly, can you slow down the movement?

¶190: AB: Err... quite hard because I’ve been taken out of it and I’m more in this room now. I’m more in the physical world...than... any... the mental world

¶191: TF: Okay, we’ll take it easy, let’s start from the top. So if I say to you... well have a glass of water, have a quick rest! <laughs>

¶192: <pause>

¶193: AB: There’s kind of a green-like quality to it.

¶194: TF: The corridor

¶195: AB: Mmm <agreeing>

¶196: TF: Yes, okay, so let’s see if I can transport you back in there, and let’s have another look. So if I say “five”, can you see the five?

¶197: <AB Agreeing throughout>

¶198: AB: Yep

¶199: TF: You said there was a yellowish light in the corridor, can you localise the source?

¶200: AB: No, it’s hard to see from where I am now, because I’ve got this... got this big back five sort of looming right there.

¶201: TF: Okay.

¶202: AB: So it’s probably like the five is sort of darkening, darkening my experience of it.

¶203: TF: is there a shadow under the five?

¶204: AB: No shadow.

¶205: <pause>

¶206: TF: So, if you now try to move to the side of the five...

¶207: AB: Well it seems like I only have two perspectives, one was like... it’s very hard to get in between the two, two positions. The five’s all the way over there now.

¶208: TF: It’s over there?

¶209: AB: Yeah

¶210: TF: Could you guess how far, is it about three or four metres?

¶211: AB: Yeah, it’s probably about three or four metres.

¶212: TF: Okay so from this vantage point, does the corridor look different?

¶213: AB: Yeah, it’s lighter.

¶214: TF: Do you see why it is lighter?

¶215: AB: Erm...

¶216: TF: is there a source of light?

¶217: AB: It seems like there’s a source of light either end. The five’s kind of disappeared now completely, into the floor!

¶218: TF: Into the floor! <laughing> Can you bring it back?

¶219: AB: Ahm.

¶220: TF: If I say “five”.

¶221: AB: Yeah, umm, umm I’m back in front of it.

¶222: TF: So, what about if you moved left to the other side?

¶223: AB: Left of the five, yeah I’m right next to it still.

¶224: TF: So it’s closer to you?

¶225: AB: Yes

¶226: TF: How’s the lighting conditions now?

¶227: AB: Err, still quite dark, err, there’s a white light still down there

¶228: TF: What do you see behind the five?

¶229: AB: The five is just flat, there’s nothing behind it.

¶230: TF: I’m sorry, I meant along the corridor.

¶231: AB: Along the corridor... Can’t see the actual other end of the corridor, just see the wall.

¶232: TF: How do you know you can’t see the end?

¶233: AB: It’s a long corridor... I see the end now, just white... it’s very long. Kind of err... kind of got the... five’s just gone out of my field of vision... I’m err just slightly behind the five. I’m kind of aware of its presence.

¶234: TF: Is the end of tunnel providing luminance?

¶235: AB: Probably a little bit.

¶236: TF: But it’s not yellow.

¶237: AB: Kind of a white light.

¶238: TF: Can you move closer to it, further away from the five? Is the five still there?

¶239: AB: The five’s still there. Ahm, right... right on the side of the five now, it’s kind of, so it’s very flat.

¶240: <pause>

¶241: AB: So this time I’m still very close to the five and now I’ve kind of turned my whole head to look at it.

¶242: TF: so if you look at it from this new vantage point, how far would you say it is?

¶243: AB: So it’s right here.

¶244: TF: Okay so, maybe half an arm.

¶245: AB: Yeah.

¶246: TF: Okay, so let me bring in a new element, just to spice it up a little bit. Do you have any sense of any other numbers in the area?

¶247: AB: I can sort of feel a presence of a six, over here. Ahm, seems like it would be kind of, so it doesn’t, doesn’t fit in the corridor, it’s kind of err hovering around the ceiling, ahm, somewhere,.

¶248: TF: Are you sensing it above the ceiling, outside the corridor?

¶249: AB: Yeah, just above it, and smaller.

¶250: TF: Okay, so if you will it to come down, what happens?

¶251: AB: Kind of crept down the wall, and there’s like a small number six against the wall, and there’s a line, joining the five to the six. Well it’s not touching the five or the six but err...

¶252: TF: Below the...

¶253: AB: Yep.

¶254: TF: What distance is the six?

¶255: AB: The six is just about touching, it’s just there.

¶256: TF: Okay. So it came from above the ceiling, along the backside of the corridor...

¶257: <AB: agreeing throughout>

¶258: AB: Yeah, it’s about, yeah about where the ceiling joins the wall, and kind of just plonked itself on the floor.

¶259: TF: But it’s right attached to the wall, or is it in front of the wall?

¶260: AB: Err.. it seems atta... it seems... it’s not attached to the wall, it seems like it’s kind of leaning against the wall.

¶261: TF: Okay, so there’s a separation there somehow.

¶262: AB: Yep.

¶263: TF: Okay so, can you recall, when it came down, what did you see?

¶264: AB: Ahm, it was pretty quick, I didn’t see any movement, it just kind of reappeared down at the bottom.

¶265: TF: Okay, so there was no white. <questioning tone>

¶266: AB: There was no white no, no. I sort of felt the presence of the four, and the six, kind of simultaneously, and four didn’t quite come into view but then the six is... ahm, I sort of feel the presence of a four behind me, and then the six kind of appeared back, kind of above the thing.

¶267: TF: So if you turn around, do you see the four?

¶268: AB: Ahm, now, what’s going to happen if I turn around... ahm, can’t see anything behind me...

¶269: TF: So the five has remain...

¶270: AB: <Interrupts> There’s kind of a black hole behind me.

¶271: TF: So the five has remained on its spot?

¶272: AB: The five is... five is still kind of yeah, there.

¶273: TF: And the six is there?

¶274: AB: The six is over there, yeah.

¶275: TF: So but there’s nothing where the four should be?

¶276: AB: I felt the presence of a four, kind of over there.

¶277: TF: Okay.

¶278: AB: But can’t see it.

¶279: TF: Okay. So when you looked really close at the five, it was right next to you, has the colour stabilised now

¶280: AB: Ahm... yeah, it’s black, it’s black. I thought perhaps if you shone a really bright light on it then it might have a dark sort of, dark, shiny sort of metallic sort of lustre to it, but erm... it’s quite sort of vague.

¶281: TF: Dark, metallic and shiny? So the yellow light in the corridor, does it give the impression that the five is shiny, or how do you know that it has this quality?

¶282: AB: Just from imagining the light shining on it from above.

¶283: TF: Can you turn the light on and off?

¶284: AB: Did see briefly, there’s kind like a white sort of ribbon just on one little bit of it, ahm, most of it’s pretty black, it’s just like it’s lighting up a little bit of err, err, curvy bit. And th-th-the top bit is... is sort of straight lines at the top bit, it’s completely black.

¶285: TF: And what did you see at the bottom bit when it was lit up?

¶286: AB: It was like a kind of like a white, kind of crescent type thing.

¶287: TF: And how did the five look?

¶288: AB: So it kind of became a bit erm, a bit disentangled, like I could... I wasn’t really aware of the whole number five at, at the same time. So, I sort of look at the top of it and it’s kind of the black, and I look at the bottom and it kind of sh... it kind of shimmers a bit and sort of... the whole image sort of moves around a bit, sometimes the face, the curvy bit is kind of going the wrong way and it’s kind of like this.

¶289: TF: Do you have a sense of why that is?

¶290: AB: No. Err. Still like a five, but, still thinking of it as five, but then I suddenly realise that five doesn’t go that way round.

¶291: TF: Okay.

¶292: AB: And then it kind of went back.

¶293: TF: Okay, if you don’t mind, I’d like to probe the shininess a little bit more. You reported the shininess even when there was no light... and you had a sense that even if there was no light, it would be shiny. Can you go back to that moment before the light, let’s have another look at whether you can see any hints of why you might be under the impression that it’s shiny, even without the light

¶294: AB: Erm, I think I was just envisioning another number five, that was kind of here. And it had a kind of dark grey colour to it, just like kind of metallic.

¶295: TF: And how was that five related to the other five?

¶296: AB: So... just kind of appeared, it wasn’t in the same room.

¶297: TF: And then you were able to compare the two?

¶298: AB: Yeah, yeah. Yeah when I compared the two, a light kind of started shining on the actual five that was down here.

¶299: TF: And when that light shone on the other one, did it reveal to have the same colour at that spot, or a different one?

¶300: AB: Ahm, it’s kind of different because the one that appeared here was much better illuminated.

¶301: TF: Can you say something about its texture?

¶302: AB: The one that appeared here? It’s kind of metallic.

¶303: TF: And the one that was in the spotlights, did it have a similar texture? Or was it a different one?

¶304: AB: Err, it was much err thinner, so one in front of me, that was like had a sort of er width to it, like a depth, ahm, but this one’s kind of much flatter.

¶305: TF: <agreeing sound> You said it was outside the room. How did you see that?

¶306: AB: So I don’t know. Now, now when I sort of think of it I kind of see, it is kind of juxtaposed and it’s kind of in the room. So now I’ve kind of got two fives in this room. If I look over there I’ve still got this one, if I look here I’ve got this one.

¶307: TF: So how do they relate to each other? How does the number line connect them?

¶308: AB: So there’s a number line going this way, through the big one there, and this one... if I look over there there’s a little six, so there’s like two number lines now. But erm... I can only see one at a time

¶309: TF: So you have to change perspective to view them? So when you have both of them present, is the metallic one that’s in front of you also in the corridor, or is it just in an empty space?

¶310: AB: It’s in the corridor now

¶311: TF: The same corridor, or a different corridor?

¶312: AB: It’s... all in the same corridor

¶313: TF: So...

¶314: AB: <interrupts> So I can kind of see it here, I can kind of see the six over there.

¶315: TF: So how does the corridor...

¶316: AB: <interrupts> The six is connected to this five.

¶317: TF: So how does the corridor fit both of them?

¶318: AB: Well, yeah, this one’s just kind of is against the wall, up here, and kind of just about fits, the corridor’s just about high enough for that five to fit, and the other one’s just here.

¶319: TF: And the six that would match with this five, can you see that one too? Can you bring it into view?

¶320: AB: Erm... there’s just kind all the way up there, I kind of feel the presence of a six but I can’t really bring it forward, it seems like a heavy like metallic five, that’s hard to move. But I sense a six, up there.

¶321: TF: And there’s a number line at the floor, going in that direction, or...?

¶322: AB: Erm... Not really much room for a number line, because the five is kind of pretty much against the wall, so... I saw a whole cluster of numbers over there, trying to kind of ‘get in on the scene’, like fours and threes and twos and things!

¶323: TF: So, they really want to be part of the game! Which number line are they part, or which...

¶324: AB: <interrupts> They’re not on a number line, no.

¶325: TF: So, it hasn’t been decided yet which way they are going to go?

¶326: AB: No.

¶327: TF: Are they the heavy type or a new type, or...?

¶328: AB: They’re just little black things, like with this... kind or err, kind of scramb... almost like kind of toys, so err, like, like a kid has just left them on a mess on the floor!

¶329: <both laugh>

¶330: TF: Well, this is all from me now, if you would like to explore anything else, just let me know, and then I can try to listen to more experiences. Is there anything in this that has raised your interest? Is there anything that you would like to do? Or any final comments?

¶331: <AB: Agreeing throughout>

¶332: AB: Yeah, don’t know, I want to see if I can move that big five, that’s... that’s appeared there.

¶333: TF: Can you move in relation to it, like you did before, with the other five?

¶334: AB: Yeah now there’s a five that’s against the wall, there’s a four, looking down the corridor there’s kind of a four, over there against the other wall, so that’s where the four is.

¶335: TF: is it a metallic four or is it a black four?

¶336: AB: Ahm, it’s kind of metallic, but ahm it seems softer than the five. And from my perspective now, four’s kind of the wrong way round. Well I mean yeah, it’s kind of sort of so it would be the right way round if I was standing sort of over there, but the four’s kind of there and I'm here, so... see what I mean. The same way round as the five, but the five’s there and the four’s there.

¶337: TF: So can you shift your perspective to see it properly?

¶338: AB: Ahm, it’s kind of hard, erm, I come along, ahm, it’s kind of stuck against the wall there.

¶339: TF: Does the physical wall interact with the number?

¶340: AB: Ahm, it seems a little bit stuck to the wall, but there’s not a line connecting the two of them, it doesn’t touch either of them, but it’s just kind of err, on the floor.

¶341: TF: What kind of texture does the line have?

¶342: AB: It’s black.

¶343: TF: Is it the same as before, or a different one?

¶344: AB: The same.

¶345: TF: So you can move in relationship to these metallic numbers, but you have difficulty of moving them around?

¶346: AB: Yeah. So now four’s there and the five is here again.

¶347: TF: Okay.

¶348: AB: Yeah, I can't get like in front of the four.

¶349: TF: Okay.

¶350: AB: I don’t know if I am, it’s kind of like, I'm kind of in the four.

¶351: TF: <laughs> So in terms of the five, can you just kind of remember the experience when it first appeared?

¶352: AB: Well I’ve just got this vague sense of kind of a metallic five, at first it wasn’t in the corridor, and then when I thought about it some more it ended up becoming grounded in the floor

¶353: TF: And do you recall why you had a sense of the metallic five?

¶354: AB: Don’t know why, I think we, were we talking about what colour the five was?

¶355: TF: We were talking about the black five. So is this the sequence of events, just trying to recall. So we were talking about the black five, and then you said “oh, I think it might look a little bit shiny or metallic”, and then I said “how do you know that”, and then you said “oh because there was this metallic five here”. Is that how it happened?

¶356: <AB: agreeing throughout>

¶357: AB: So I can't remember the order now, so I’ve sort of got some images of er... a black five over here, that’s more kind of velvety, erm... and with this some sort of white light shining on, because sometimes the bottom of that five is kind of the wrong way, err the curve goes the wrong way. But erm, there’s kind of this white shimmering light on that bottom half.

¶358: AB: The black at the top is kind of much darker, so there’s a little bit of white light on the top now, because we’ve introduced this light up here. So.. So it’s kind of shining, kind of getting a bit of light on top.

¶359: TF: So the light that’s shining on that five actually has a source?

¶360: AB: Yes, it’s up here.

¶361: TF: Is it in the corridor, or...

¶362: AB: Erm, kind of like a sort of sun roof has opened up in the corridor.

¶363: TF: So, what does it look like, the point where the light crosses into the corridor?

¶364: AB: Ahm, it’s just like a white hole.

¶365: TF: A round hole?

¶366: AB: No it’s just err, it’s like a square hole? It goes from one wall to the other. Ahm The whole corridor’s becoming a bit less stable now, it’s kind of err, the roof from this point onwards has just kind of err melted away.

¶367: TF: And what is revealed, behind the...

¶368: AB: <interrupts> Just whiteness.

¶369: TF: But this whiteness, is it same or different than the light that is shining through?

¶370: AB: It’s not as pure white, errm, it’s not as pure white, there’s kind of a few colours in there.

¶371: <Pause>

¶372: AB: A few little numbers floating around up there as well.

¶373: TF: <laughs> Outside the corridor?

¶374: AB: Yeah, like up in this kind of white space. It’s not as pure a white, there’s kind of a creamy colour in there and sort of, some very soft blues and yellows and things.

¶375: TF: So it’s a nice white?

¶376: AB: Yeah, it’s quite nice to look at. Kind of... Almost like a cream cake kind of thing.

¶377: TF: Okay. So what interests me is some of the differences that became clear in the two sessions. So last time you said there was a frame. This time, you didn’t see any frame?

¶378: AB: I didn’t see a frame.

¶379: TF: And, last time you said there was also a few colours around the numbers, this time there are no colours around the numbers?

¶380: <AB: agreeing throughout>

¶381: AB: There was a little bit on the metallic five, at times there was like a, erm sort of like blue or purple, something like that. Ahm,

¶382: TF: Okay, so if you look at the metallic five...

¶383: AB: So there’s kind of like a grey outline, and then kind of on the face of the number kind of bluey purpley kind of colour. Ahm, slightly unsure which. Blue and purple kind of “accounts” on my colour blindness kind of seeing which is which, it’s kind of in between kind of.

¶384: TF: Okay. So the outline is grey, and the middle is a bit of these colours?

¶385: <AB: agreeing throughout>

¶386: AB: Sometimes it goes a bit black as well, in the middle, so, it gets black in the outside.

¶387: TF: Okay, so just out of curiosity, the four was here?

¶388: AB: The four...

¶389: TF: Or was it the six?

¶390: AB: So... There’s a little six over there, err, at one point there was a four kind of around my face, sort of against the wall over there.

¶391: TF: Which one is bigger, the four? Can you still see the four?

¶392: AB: Yeah four’s much bigger than the six.

¶393: TF: So if you look at the four, and in terms of the colours and the texture, how does it compare with the five?

¶394: AB: It’s flat, the four’s kind of black, kind of like, so it’s kind of like this five.

¶395: TF: So it’s velvety like the five? You said before the five was kind of black-velvety.

¶396: AB: It seems kind of black and like stuck on the wall.

¶397: TF: Okay.

¶398: <long pause>

¶399: TF: What do you see?

¶400: AB: Black four, against the wall.

¶401: TF: You were just looking at the black four?

¶402: AB: Yeah.

¶403: TF: Okay, so shall we leave it here for this week?

¶404: AB: Okay, thanks <quietly>
