# Supplementary material for: An extended case study on the phenomenology of sequence-space synesthesia
Source: Front Hum Neurosci. 2014 Jul 3;8:433. doi: 10.3389/fnhum.2014.00433 (PMC4080762; doi:10.3389/fnhum.2014.00433)
Supplement: Supplementary file 1 [file Presentation1.ZIP › Supp Mat 2.ZIP!/Referenced_Interview_Transcipts/AB - 20100229.doc]

# Name: AB - 20100229

# Description: Dialogue between TF and AB on 29/02/2010

# ¶1: Dialogue between TF and AB on 29/02/2010

¶2:

¶3: TF: Ok so to begin with, in this session, did you have a look at the number 5 last time and you just suggested number 4? What if we say, imagine the number that is sitting between 4 and 5.

¶4: AB: So I saw 4.5. So, first I sort of saw 4 and then I saw 5 and then I saw 4, 4.5 and 5 all in a horizontal line.
¶5:
¶6: TF: So the 4.5 <pauses> is presented in the same digits and the same form as the 4 and the 5?
¶7:
¶8: AB: Yeah

¶9: TF: Interesting, we found the number that actually has the two digits in there.
¶10:
¶11: AB: Yeah <laughs> yeah, that’s strange
¶12:
¶13: <more laughter from TF and AB>

¶14:
¶15: TF: Ok, so just to check, take another fraction. So if we take between say 1 and 2, what do you see?

¶16: AB: I see 1, 1.5 and 2. It’s very interesting, it’s horizontal. <quiet>
¶17:
¶18: TF: Its normally not horizontal?
¶19:
¶20: <over-talking>
¶21: AB: Well 1 to 2 without any decimals in between is like that

¶22: TF: Right

¶23: AB: But 1, 1.5, 2 is… <pauses>
¶24:
¶25: TF: Straight? <questioning tone>
¶26:
¶27: AB: Straight
¶28:
¶29: TF: So, if you just forget about the fraction right now and just say, let’s go back to our old favourite the 5, what does the slope look like?
¶30:
¶31: AB: 4, 5, 6, 7, 8
¶32:
¶33: <cross-talking and laughter>
¶34:
¶35: TF: And when let’s say 4.5 what happens to the slope?

¶36: AB: <pauses> So, I saw 4.5 for quite a while with nothing else extra and when other numbers did come into view 4 and 5 that’s horizontal.
¶37:
¶38: TF: Ok, so in both cases it was turned to horizontal?
¶39:
¶40: AB: Yeah

¶41: TF: Ok so if you go back to the, between 1 and 2. Can you change the representation of the fraction?
¶42:
¶43: AB: Yeah I can see there is 3 over 2
¶44:
¶45: TF: Can you see any difference between 3 over 2 or 1.5?
¶46:
¶47: AB: See them as super imposed, one on top of the other

¶48: TF: They are both present at the same time <questioning tone>

¶49: (3.02)

¶50: AB: Erm, <pauses> yeah I can kind of see just like a juxtaposition <unclear> which one is on top. I can make the 1.5 fade away or I can make the 3 over 2 fade away

¶51: TF: How can they be at the same place at the same time?
¶52:
¶53: AB: <long pause> That just as if you’ve written them on the same bit of paper, one on top of the other
¶54:
¶55: TF: And so they actually, does that mean they are hard to recognise, I mean it would create a mess right?
¶56:
¶57: AB: No, they are easy to recognise, erm

¶58: TF: So do the contours of the two different symbols go into each other?
¶59:
¶60: (3.50)
¶61: <over-talking>

¶62: AB: So the 3 over 2 occupies more vertical space. So all of the numbers I think are the same size, like all the actual digits. So you got the 3 over 2 and then you got the 1.5 and that

¶63: TF: Ok, so they are quite easy to separate because of the 3 over 2 is <questioning tone>
¶64:
¶65: AB: Sticking out
¶66:
¶67: TF: Sticking out <questioning tone>
¶68:
¶69: AB: Yes, they’re not totally covering each other up
¶70:
¶71: TF: Yes, but where they are covering is it that the colour of the 2 I presume are black?
¶72:
¶73: AB: Yeah they are all black
¶74:
¶75: TF: Is it that they basically, what’s it called you know, merge or is there separation where they overlap?
¶76: AB: Erm, <long pause>
¶77: (4.54)
¶78: AB: quite hard to focus on the whole thing as it’s a little bit blurry. Erm I can zoom in on one bit of it and sort of see a decimal point, sort of slightly above the line that separates numerator from denominator, slightly to the left of centre. So you got the line separating 3 over 2 and then I’ve got the point. The decimal point is kind of, slightly above the line and slightly to the left. Slightly to the left of the centre of the line
¶79:
¶80: TF: So if you wanted to look at them from the side let’s say would that separate them out?

¶81: AB: Uhm

¶82: TF: Or do they fall flat on each other?

¶83: AB: Yeah they’re just flat on each other. I’m just seeing them on a page basically.
¶84:
¶85: TF: Is it the white page?
¶86:
¶87: AB: Yeah

¶88: TF: Is the white page still there?

¶89: AB: Yeah, the white page is still there this time
¶90:
¶91: TF: Can you see the number line at the same time?
¶92:
¶93: AB: Yeah, when I see the number line it’s going like this and below. So I can sort of see 1 and 2. Now its kind hovering above mid way between 1 and 2, these fractions
¶94:
¶95: TF: Does the line look different beneath them?
¶96:
¶97: AB: The line looks pretty much the same as before. There’s a number 1 here and there’s a line and number 2 peering to the left hand side of the line. Slight gap in er the line at number 2
¶98:
¶99: TF: So what if I said think of a different representation once more about these fraction. I mean we could say for example 6 over 4 <questioning tone> What do you see?
¶100:
¶101: AB: <pause> Quite fleetings. It’s kind of appearing in the same place. And likes to go back to 3 over 2

¶102: TF: It likes to go back to 3 over 2 <questioning tone>

¶103: AB: Yeah

¶104: TF: So does it have a tendency towards 3 over 2?
¶105:
¶106: AB: So now I’ve got 6 over 4 as being slightly up and to the right of the 3 over 2
¶107:
¶108: TF: so it’s a different…
¶109:
¶110: AB: It’s still overlapping slightly
¶111:
¶112: TF: But there’s some more space now <questioning tone>

¶113: AB: Erm <pauses> so the 6 is kind of unimpeded but then the 4 is getting in the way of the 3
¶114:
¶115: TF: Ok
¶116:
¶117: TF: But if you like there was a tendency towards the 3 over 2. Could you describe that?
¶118:
¶119: AB: But now I’ve got both, they’re not in the same place
¶120:
¶121: TF: So, shall we try pie?
¶122:
¶123: AB: Yeah
¶124:
¶125: TF: So 3.1415 and so on
¶126:
¶127: AB: Yeah. So I see the Greek letter pie equals and then I’ve got 3.1415
¶128:
¶129: TF: Let’s see how far you can see
¶130:
¶131: AB: 2, 7
¶132:
¶133: AB: I’m not even sure if these are right now but I am seeing 2796 too. And then just some kind of vague numbers just kind of going off and I can’t. I’m not really sure what they are because they are sort of an 8 and then another 6 2. Erm yeah, seems a bit unstable
¶134:
¶135: (8.56)
¶136: TF: Okay, so let me just rephrase <unclear> So when I told you to think of pie you saw the symbol pie with the equal sign and then the numbers. Just correct me if I’m wrong: 3.141527962862
¶137:
¶138: AB: Right. So it’s unstable after 3.141527986. Seems pretty unstable after the third or fourth decimal point.
¶139:
¶140: TF: Ok, so just curious. What does unstable look like?
¶141:
¶142: AB: Erm <long pause to think> So the focus on one number. The one to right is kind of just changed. Because it’s not really holding still it’s kind of changing because of other numbers trying to get in from above.
¶143:
¶144: TF: So there’s some numbers floating above the unstable digit <questioning tone>
¶145:
¶146: AB: So it’s 3.14 and 1 and a 5 and that’s where I start getting difficulties. Next to the 5 there’s like I say a 2 to the right of the 5 but there’s a 9 above the 2
¶147:
¶148: TF: How is it above the 2?
¶149:
¶150: AB: Just directly above on the page
¶151:
¶152: TF: Is it stable up there?
¶153:
¶154: AB: I see a 1 a 2 and a 9. I see a 7 to the right of the 2 and the 9, slightly above the 2. I just see a jumble of numbers. When I am focusing on that region now the pie has gone. I’ve just brought that back by saying that
¶155:
¶156: (11.33)
¶157: AB: Pie equals 3.1415926. Yeah and the 9 to the side of me <unclear> just kind of still there. Just kind of not really working themselves out into anything coherent.
¶158:
¶159: TF: So the stable numbers are on the left side as you go right eventually you hit a point where the numbers are unstable. Does that mean it’s not there or it’s there but there are numbers above it right <questioning tone>
¶160:
¶161: AB: Yeah so as I focus on it, it becomes stable. So it’s like, so it’s almost like I have to fill it in with something. But they just seem to be random numbers.
¶162:
¶163: TF: So could you make that choice, let’s say you want it to be a 9?
¶164:
¶165: AB: A 9 where, after the 5 <questioning tone>
¶166:
¶167: TF: After the 5 you told me it's a 2. The 9 hovering above it
¶168:
¶169: AB: Yeah, so can I make. Can I get rid of the 2 and put a 9 there?
¶170:
¶171: TF: Yeah
¶172:
¶173: AB: Difficult. The 2 doesn’t really want to move out of the way
¶174:
¶175: TF: Ok so what if you move one further along
¶176:
¶177: (12:46)
¶178: <cross-talking>
¶179:
¶180: AB: I didn’t know what the next digit pie is actually 15
¶181:
¶182: TF: <laughs>
¶183:
¶184: TF: I’m only interested in what you know
¶185:
¶186: AB: I’m not 100 per cent sure after the err. But definitely sure of 141 and fairly sure of 1415 and then if I had to make a guess about the next one I would say 9 but there’s a stubborn 2 that’s erm not quite getting out of the way <laughs>
¶187:
¶188: TF: Right so I mean. Forget about what you think in numbers. I mean it doesn’t matter if it's right or wrong. What we are interested is like the patterns of stabilisation and instability and how they change and so on. So it's a stable 2 there with a 9 hovering above?
¶189:
¶190: AB: Yeah, well the 9 is kind of going above on top of the 2 a bit like the fraction was before
¶191:
¶192: TF: What do you see if you look beyond the 2 further down the sequence?
¶193:
¶194: AB: An 8, a 6 and a 5
¶195:
¶196: TF: And they also have numbers above them or they just <questioning tone>
¶197:
¶198: AB: Sort of floating there. I’m sort of seeing a dot, dot, dot and then sort of. There seems to be a rotation where it's starting to go off in that direction away from me.
¶199:
¶200: (14.10)
¶201: <cross talking, unclear>
¶202:
¶203: AB: From the number line actually yeah. When I focus on it there, like those endless decimal places of phi, pie and
¶204:
¶205: TF: So before the number was
¶206:
¶207: AB: It’s going off into kind of like a black void
¶208:
¶209: TF: So before the number was not in line with the number line, because you said that at this point it turns to the direction of the number line <questioning tone>
¶210:
¶211: AB: Yeah, so after the er. I guess after the 2 and 9 are sort of sitting there struggling. Erm and then somewhere like an 8, 5 and a 6 and then just kind of seems to vanish and then it's kind of like dot, dot, dot and then sort seems to head towards this black hole
¶212:
¶213: TF: So, how does this black hole relate to the number line? Is it above it
¶214:
¶215: AB: Numbers sort of going into the middle of it
¶216:
¶217: TF: What is the sequence displayed on?
¶218:
¶219: AB: The sequence in the beginning is displayed on a white page and then somewhere it's kind of leaving that and not really sure how. For a bit it's against that kind of erm greenish, reddish orangey background that I think is the same background we were talking about last week and then some point at erm there’s this sort of as you go towards the right there’s this blackness just sort of hovering there and it's kind of going into that
¶220:
¶221: TF: Ok. So can you see at which digit the white page ends?
¶222:
¶223: (16.14)
¶224: AB: Erm <pauses> No <speaks quietly and is unclear> maybe have to get back to the beginning.
¶225:
¶226: TF: So if I say pie and you see the symbol pie and you see the white page behind and then we go along, what do you see?
¶227:
¶228: AB: So this time the white page is kind of melting away and I am kind of against that kind of coloured background again. Then I got pie equals 3.1415, still a 9 up there and 2 there. And then so there’s that kind of coloured background behind all of those. And to the right of those there’s this kind of blackness <pauses> and so what’s happened to the other numbers. So then they kind of for a bit appear on the sort of floor.
¶229:
¶230: TF: So if you keep in mind pie and you also try to think of 3 at the same time
¶231:
¶232: AB: So I saw a 3 there
¶233:
¶234: TF: So it's in a different location <questioning tone>
¶235:
¶236: AB: So now I’m still in this kind of coloured sort of corridor. Erm it's a darker sort of light, it's almost, almost like some kind of subterranean place. There’s a slight glow of like sort of flames or something like the end of the world or something. Erm so I can see 3 and then I can see pie equals 3.1415 9. Now the 2 is above the 9. They are still not stable <laughter and cross-talking>. And then there’s some numbers kind of on the floor in front of the blackness which is covering the wall on the 9 and just kind of going off.
¶237:
¶238: TF: Are those numbers potentially part of the sequence or are those the numbers of the number line?
¶239:
¶240: AB: They’re just potentially numbers that could go into the sequence but they don’t know where to go
¶241:
¶242: TF: How does the black hole appear on the wall? Is it part of the corridor?
¶243:
¶244: (19.04)
¶245: AB: It’s just like a smudge. So it's just <cross-talking>
¶246:
¶247: TF: Does it look like it has any depth or is it flat?
¶248:
¶249: AB: It’s flat and it's just covering. It's like kind of in like rising so it's like in the middle of the corridor. It's like blocking the back half of the floor and the blocking the wall. And doesn’t go on forever though there’s like the corridor is a. I can see the wall of the corridor again if I go over here with a kind of white light at the end of the corridor.
¶250:
¶251: TF: So there’s still a white light at the end of the corridor <questioning tone>
¶252:
¶253: (19:40)
¶254: AB: Yeah. There’s this big black <inaudible> there. Just concentrating on the black then it kind of flickers for a bit and the corridor just kind of comes back for a second
¶255:
¶256: TF: So if you start from the beginning again. I’m just interested also in the white page so if you think of pie in the beginning is the white page there again?
¶257:
¶258: AB: Yeah
¶259:
¶260: TF: What is happening is it staying?
¶261:
¶262: AB: Erm. No, kind of goes away and <pauses to think>. So it's almost like that’s kind of a new, so I was kind of looking over there towards you and there was, so I get this kind of page but then I’m kind of feel my eyes drawn back to this corridor which I’m now in. There’s number 3 and then we’ve got pie equals 3.14159, 2 floating above this. It's because all of the digits have gone smaller
¶263:
¶264: TF: Oh yes, let’s try that. I mean, if you look at the number pie from the side, does that give you a better view of what’s going on?
¶265:
¶266: AB: Erm, it's very vague uhm <pauses to think>
¶267:
¶268: TF: So I noticed just now that it didn’t actually shift around orderly. Were you still able to shift the view?
¶269:
¶270: AB: Erm, yes I’m kind of looking down now pie is kind of over here and it's just got much smaller. So there’s just kind of lots of numbers but I can’t really read them. I mean I can focus on erm individual numbers I can just see a 4 there and a 9. Well now I’m seeing a sequence 4 9 3 2 8 6 but it's like I see sequences of random numbers. Erm, well I just can’t relate those to the beginning of 5.
¶271:
¶272: TF: So it's just a chunk of sequence basically <questioning tone>
¶273:
¶274: AB: Yeah
¶275:
¶276: TF: Ok. So there’s another space for pie in the corridor then. Is that what’s going on?
¶277:
¶278: AB: Yeah. I think that’s what’s going on. So I looked to the left of those random numbers I’ve just said and then I saw 3.14159 again. Then I seem to not be able to get back to the same numbers as I approach it again from the left hand side.
¶279:
¶280: TF: So there’s a piece in the middle missing somehow <questioning tone>
¶281:
¶282: AB: Yeah.
¶283:
¶284: AB: And then I see dot dot dot
¶285:
¶286: TF: So you actually see 3 dots in the corridor?
¶287:
¶288: AB: Yeah
¶289:
¶290: TF: Before the black hole or where do they appear?
¶291:
¶292: AB: So I’m kind of shifted perspectives now so I’m kind of seeing it down here. I’m looking down quite steeply at it. Yeah I am aware of the black hole over there.
¶293:
¶294: TF: But it's further away from the sequence.
¶295:
¶296: AB: Further away from the sequence now
¶297:
¶298: TF: So the dot dot dot is actually in the middle of the corridor <questioning tone>
¶299:
¶300: AB: So now I’ve got 3.14159 … 8656 and then a whole pool a whole load of numbers just kind of like jumbled up like kind of just there not really knowing where to go.
¶301:
¶302: TF: What to the side of it <questioning tone>
¶303:
¶304: AB: Er yeah kind to the side and behind the main sequence. Kind of just lying on the floor.
¶305:
¶306: TF: Ok, so is there anything else you want to explore about this number?

¶307: AB: Erm <pauses>
¶308:
¶309: TF: I mean maybe one thing if you’re interested. You said the lighting conditions were different. Can you see why that is?
¶310:
¶311: AB: Erm, maybe because of the black presence has made it kind of darker in there.
¶312:
¶313: TF: The smudge on the wall <questioning tone>
¶314:
¶315: AB: Yeah. I look down that end and there’s a white light.
¶316:
¶317: TF: I mean we could try something if you want. Can you try shining a light on the black smudge?
¶318:
¶319: AB: Doesn’t do anything. Black smudge won’t go away <quiet>.
¶320:
¶321: TF: Ok. Is it that the lighting conditions doesn’t change because of that?
¶322:
¶323: AB: Erm, no <pauses to think> All you get is little light overhead lights. It's like a ceiling with little electrical lights. Little circles of light going down the corridor.
¶324:
¶325: TF: But it just shows you the black smudge basically <questioning tone>
¶326:
¶327: AB: Yeah. Yeah so the black smudge is just this kind of presence just blacked out. Since it's being this infinite thing.
¶328:
¶329: TF: But I guess it must since it's a different kind of infinite than the white light.
¶330:
¶331: AB: Yeah
¶332:
¶333: TF: Because the white light is also where the number line goes off to infinity.

¶334: AB: Errrm. Well, I haven’t seen any numbers go in to those white lights. I can see the number line as going in. there’s more going into the wall of the corridor.
¶335:
¶336: (25.52)
¶337: TF: Jiggles and <cross talking>
¶338:
¶339: AB: Last time I remember seeing the 5 and 6, sort of hitting the wall of the corridor and the line would go through the wall. And, I don’t know of any numbers over there. Both either end of the corridor <spoken quietly>
¶340:
¶341: TF: I must have misunderstood. I thought the number line was going one end of the corridor to the other side and both were.
¶342:
¶343: AB: No, it's coming from diagonally intersecting the corridor.
¶344:
¶345: TF: Ok. So the corridor is not following the number line <questioning tone>
¶346:
¶347: AB: No, it's really strange <quiet>
¶348:
¶349: TF: So what is down there at the end? I mean it's just an empty corridor or?
¶350:
¶351: AB: Yes, empty corridor.
¶352:
¶353: TF: Can you shift the corridor?
¶354:
¶355: (26.33)
¶356: <cross talking>
¶357:
¶358: AB: Always just like the stage is set for these numbers that I am thinking about
¶359:
¶360: TF: Ok, so but the more numbers you think of it's actually what happens the corridor gets wider <questioning tone>
¶361:
¶362: AB: The numbers themselves shrink
¶363:
¶364: TF: They shrink <questioning tone>
¶365:
¶366: AB: Yeah.
¶367:
¶368: TF: So how wide is the corridor then? Can you give me the dimensions?
¶369:
¶370: AB: Erm <pauses> probably about within maybe just within maybe a little less wide than I am high.
¶371:
¶372: TF: A little less <quiet>
¶373:
¶374: AB: Seems to change a bit by trying to imagine myself lying across it. It's kind of, seems like there is room for me to lie down. So maybe a bit wider than I am
¶375:
¶376: TF: Ok. And so the number normally comes across as a diagonal?
¶377:
¶378: AB: Yeah
¶379:
¶380: TF: So is that how you can measure the angle then in terms of what’s the angle in relation to the side wall on the corridor?
¶381:
¶382:
¶383: AB: Yeah. Just now when you said 3, think of 3 and pie it is going along the corridor. So 3 is erm directly to the left of pie. When I think of 4 and 5, they are more like this <external sound> (28.05)
¶384:
¶385: TF: Ok and how does it intersect the wall over there?
¶386:
¶387: AB: Erm, the number line disappears into the other wall. Into the back wall.
¶388:
¶389: TF: But there’s no difference in colouration or anything like that to mark the exit basically. But in terms of pie, if you go back to pie, where does the number line head off to? Where does it intersect the wall?
¶390:
¶391: AB: Erm, I don’t really have a number line appearing in that image.
¶392:
¶393: TF: But you have a 3 right?
¶394:
¶395: AB: I have a 3
¶396:
¶397: TF: You have a pie equals 3 1 4, but they don’t have a number line <questioning tone>
¶398:
¶399: AB: No. I had a vague number line popping into view coming out of the 3. Or stemming from near the 3 and going into the wall near the 3. But 3 is all the way over here and the black hole is over there. The number line from the 3 is coming over here, getting a <inaudible due to external noise> (29.15) and the black hole only starts here.
¶400:
¶401: TF: Ah ok. Maybe we can like scope out the space a little bit. So if I said 3.5 we should be on the other side I guess.
¶402:
¶403: AB: Yeah, that appeared over here. Away from the black hole.
¶404:
¶405: TF: So you have 3 on your left hand side, 3.5 on your right hand side and pie somewhere in the middle <questioning tone>
¶406:
¶407: AB: Yeah. Closer to 3 than 3.5.
¶408:
¶409: TF: And
¶410:
¶411: AB: So, it's like that black hole is sucking up all those infinite digits in pie to just sort of get them out of the way. Otherwise they are going to crash into 3.5.
¶412:
¶413: TF: Yeah so there’s no space to fit in between <questioning tone>
¶414:
¶415: AB: No, so they are curving away from that axis along the corridor.
¶416:
¶417: TF: Ok, so now I understand so there’s 3 and 3.5 are on one slope so to speak and closer to the 3 you have this pie sequence. But because there’s no space for it, it curves after a few numbers and hits the wall at a difference phase and then the number line which goes to the 3.5.
¶418:
¶419:
¶420: AB: I don’t see a number line, any line connecting the 9, but I do see 3.5 and I do see 4 in the distance along there so.
¶421:
¶422: TF: So what do you see on the floor?
¶423:
¶424: (30.41)
¶425: <Cross talking>
¶426:
¶427: AB: There’s a horizontal sequence 3 3.5 4. Yeah.
¶428:
¶429: TF: Ok. So, horizontal you mean in parallel with the corridor or?
¶430:
¶431: AB: In parallel, in lengthwise along the corridor <quiet>
¶432:
¶433: TF: So how does the end of <inaudible> (31.01)
¶434:
¶435: TF: So what is on the floor if there’s no number line?
¶436:
¶437: AB: There’s nothing, just pure colour.
¶438:
¶439: TF: Ok.
¶440:
¶441: AB: The same kind of slightly indeterminate colour that I was describing before.
¶442:
¶443: TF: Ok, so that’s interesting. So, somehow the pie is preventing a number line from appearing from the floor <questioning tone>
¶444:
¶445: AB: Yeah. There’s also nothing between 3.5 and 4.
¶446:
¶447: TF: And generally <questioning tone>
¶448:
¶449: AB: Yes. So it's almost like the segment between 3 and 4 is not really a line, it's just <pauses> dunno, dunno what it is, it’s just <pauses> I can’t think, I can’t think of 5 as being further down from that 4 in the same place, in the direction. It kind of likes to leap over there so more, so kind of the diagonal relations of 4. And the same with 2. I can get it to appear for a second to the left of the 3. It likes to kind of move behind.
¶450:
¶451: (32.26)
¶452: <Cross talking>
¶453:
¶454: TF: So, would you please just draw it to me perhaps, it would be easier?
¶455:
¶456: AB: Yeah.
¶457:
¶458: TF: So the 2 3 and 4 are in one <questioning tone>
¶459:
¶460: AB: So 2 3 and 4 would be like that. And <pauses>
¶461:
¶462: TF: Ok, also draw the corridor in relation to that please.
¶463:
¶464: AB: Er, so I can’t see that right now in the corridor. If I do think about that then it's going to be the wall is there. So I’ve got the white light there.
¶465:
¶466: TF: And what about the sequence <questioning tone>
¶467:
¶468: AB: My head is here. And er, I can’t quite see the wall behind so I can only see there.
¶469:
¶470: TF: So your head is already past the wall basically?
¶471:
¶472: AB: Yeah. So there’s always changes every time I look at it. Because, I can get a number 1 appearing here.
¶473:
¶474: TF: But they are all on the same
¶475:
¶476: AB: So this line always constantly moving around so. It's never like one particular position with the wall but this is a general feature there’s a line just kind of like that. The line goes into the wall, sort of maybe somewhere there, slightly above the floor.
¶477:
¶478: TF: Ok. So this is for the normal number sequence <questioning tone>
¶479:
¶480: AB: Yeah.
¶481:
¶482: TF: Now if I bring back pie into it.
¶483:
¶484: AB: Yeah.
¶485:
¶486: TF: How does the view change?
¶487:
¶488: AB: So then I have
¶489:
¶490: TF: Can you just do a new drawing?
¶491:
¶492: (34.17)
¶493: <Cross talking>
¶494:
¶495: AB: Yeah. So that’s the corridor. So then I have 3, then I have pie equals 3.14159 and this is where I have difficulty seeing it. There’s this black hole right here and then there is sometimes a few numbers just kind of on the floor here. Sometimes the dot dot dot really changes. Sometimes like little sequences like 8 5 6. Sometimes just loads of numbers just scattered on the floor like that. And then sometimes I see it just kind of curved into there. I mean imagine being sucked into there and then 3.5 is safely over here.
¶496:
¶497: TF: And the 2 was somewhere on that side.
¶498:
¶499: AB: Yeah, and then 2 I can’t really stabilise very well. So sometimes it's appearing right down the corridor down here. Sometimes it's appearing sort of behind so it's more kind of like down there.
¶500:
¶501: TF: Does that mean that the angle of this line has changed with the appearance of this number?
¶502:
¶503: AB: No, I don’t think this line changes. Basically, I can see 3.5 and 4, a horizontal line, but I can’t stabilise any kind image of anything longer than just err, between two whole numbers in that horizontal way.
¶504:
¶505: TF: Ok cool.
¶506:
¶507: TF: Alright ok. So maybe we can leave pie for now. So there’s two options: either we have a look at some of the higher numbers and the other thing we wanted to look at was how the visualisation of the lets say the numbers in the corridor interfaces with say the room here. Which one do you prefer to look at?
¶508:
¶509: AB: Shall we look at some of the higher numbers <questioning tone>
¶510:
¶511: TF: Ok. So, any preferred range?
¶512:
¶513: AB: So, like from err 60 to 100. How many we can look at.
¶514:
¶515: TF: Ok 60 to 100. So if I say just use 60 let’s start with that then <questioning tone>
¶516:
¶517: AB: Yeah.
¶518:
¶519: TF: What do you see?
¶520:
¶521: AB: So just then I saw 6 0. Then actually I just saw the numbers 1 2 3 4 5 6 7 8 9 again after that.
¶522:
¶523: TF: So are you talking about 61 62 or are you talking about 1?
¶524:
¶525: AB: Not sure.
¶526:
¶527: TF: Ok. Let’s start from the beginning then. Say 60, what happens?
¶528:
¶529: AB: I see 6 0. And then 1 2 3 4 5 6 7 8 9 <quiet>
¶530:
¶531: TF: Ok. So if you now try to focus on 61, what happens?
¶532:
¶533: AB: It appeared up on to the right of 60, but not as steep as I would have expect from 1 to 10, a bit more like that.
¶534:
¶535: TF: So less steep angle between the two <questioning tone>
¶536:
¶537: AB: Yeah.
¶538:
¶539: TF: So the steepest of this angle is it only in relationship to the side of the corridor or does it have also an angle of, what’s it called the wide dimension you know height?
¶540:
¶541: AB: Erm, yes I’m only seeing them. Yeah, I’m not seeing them in the corridor. I’m just seeing them on a white page in front of me.
¶542:
¶543: TF: Oh right ok.
¶544:
¶545: AB: That’s the thing, when I see the actual digits and the line is in the vertical plane, but er in front of me. But when I see the number line rather than the digits that’s when I see the line is in the horizontal plane on the floor.
¶546:
¶547: TF: So when you were talking about a less steep angle <unclear> (38.53) to what were you referring?
¶548:
¶549: AB: To the, need to draw a line from the centre of the 6 0 to the centre of the 6 1. Here less steep line than the line from the centre of number 1 to the centre of number 2
¶550:
¶551: TF: So the movement
¶552: <Cross talking>
¶553:
¶554: AB: So 1 2 60 61. So 60, so it's kind of bigger, you know you want two digits kind of there and then 61 is kind of here. Whereas 1 is here 1 2
¶555:
¶556: TF: So the difference is in terms of how steeply the numbers go upwards <questioning tone>
¶557:
¶558: AB: Yeah.
¶559:
¶560: TF: Ok so and you said that’s different than if you look also at the number line <questioning tone>
¶561:
¶562: AB: Yeah.
¶563:
¶564: TF: So if you look down at the number line, how’s the angle of that line in relation to the corridor?
¶565:
¶566: AB: <pauses to think> So kind of stabilising that, not sure whether I’m thinking of 1 to 10 or 60’s but erm but I’ve got one line kind of going more like this.
¶567:
¶568: TF: Yes, so definitely 60. It made you think of the 60.
¶569:
¶570: AB: Yeah.
¶571:
¶572: TF: What happens if you look down there and think of the 60?
¶573:
¶574: AB: <pauses to think> Erm so look at the line going off here to the 60.
¶575:
¶576: TF: Do you notice anything different between this segment and the segment we looked at last week?
¶577:
¶578: AB: So there’s another, so the line coming from before 60 is kind of struggling to stabilise but it's at a different angle. Sort of somewhere more like this. A sharper angle I think. 60 is kind of in a corner.
¶579:
¶580: TF: So if I draw it like this coming up from here and then 60 and then less steep.
¶581:
¶582: AB: Yeah.
¶583:
¶584: TF: So if I drew axis to this right, this would be like height <questioning tone>
¶585:
¶586: AB: Erm, well I am looking at it in the corridor, that would be the x axis and that would be the y axis. And then the height would be the z axis yeah. And the line is kind of like that on the x y plane.
¶587:
¶588: TF: So this angle
¶589:
¶590: AB: So, so when I first saw the 60 and 61 it was in the x z plane.
¶591:
¶592: TF: So this curve I’m actually drawing is in the x z plane.
¶593:
¶594: AB: Right.
¶595:
¶596: TF: This angle.
¶597:
¶598: AB: Yeah.
¶599:
¶600: TF: And so then we have another dimension down here.
¶601:
¶602: AB: Yeah.
¶603:
¶604: TF: And then here, this floor is where the number line is <questioning tone>
¶605:
¶606: AB: Erm, yeah erm yes so when I, when I go into the, when I see it as a line rather as the than as the digits then it's in the x y plane. When I see the actual digits themselves, the first thing I think of in the x z plane.
¶607:
¶608: TF: Yes, but there is no line in the x z plane is there?
¶609:
¶610: AB: No, only digits. So the line is always in the x y plane.
¶611:
¶612: TF: So when you say there’s a steeper angle, it's just because the numbers
¶613:
¶614: AB: The numbers themselves
¶615:
¶616: TF: Are in correct positions.
¶617:
¶618: AB: Yeah.
¶619:
¶620: TF: So how does this get transposed, this difference in height here down to the x y plane, or is there no relationship?
¶621:
¶622: AB: So, that’s a bit more difficult. That’s when I have to focus just on 60, rather than 60 and 61. I was having more trouble I think because I was thinking of 60 and 61 and then trying to put them down on the
¶623:
¶624: TF: Both of them together <questioning tone>
¶625:
¶626: AB: Yeah. It didn’t really work.
¶627:
¶628: TF: So if we just took 60 and you look at it first in the vertical plane.
¶629:
¶630: AB: Yeah, and then it drops down here in the corridor and then there’s a line going off that direction.
¶631:
¶632: TF: So it's just basically as if this whole thing was flipped over down to the, so it's as if the x z plane got flipped down into the x y plane.
¶633:
¶634: AB: Yeah, it takes it down like that.
¶635:
¶636: TF: Right, so it moves down.
¶637:
¶638: AB: Yeah.
¶639:
¶640: TF: Ok, so do you see this movement happening?
¶641:
¶642: AB: Erm
¶643:
¶644: TF: So if I say again let’s start from the beginning, so if I say 60 what happens?
¶645:
¶646: AB: So I just see the number 60 then sort of white page melts away, quite similar to the, number kind of drops down and the line appears. Not quite sure at what stage the line appears in relation to the 60. To the page melting away and the numbers dropping a bit.
¶647:
¶648: TF: So the white page is associated with the x z axis, but when it starts falling down the white page melts away and that’s when the number falls into the x y plane.
¶649:
¶650: AB: So I got 60. Yeah, so the line seems to appear after the thing, after the page melting away. Then the number stays in pretty much the same place, maybe drops down a little bit after the line disappeared.
¶651:
¶652: TF: So the number doesn’t actually fall down into the other plane. Why did you think it was falling down before?
¶653:
¶654: AB: It must have fallen down at some point because it's starts off up here and then it kind of sinks more there. It's a bit unstable, it kind of yeah doesn’t really, takes a while to really settle into one place. It seems to depend on where I focus my attention where it ends up. I am thinking more about
¶655: the numbers below 60 or the numbers above 60.
¶656:
¶657: TF: So it's the, so that’s interesting let’s have a look at that a bit more. How is it context dependent? Let’s say I say think of the numbers from 50 to 60 and now let them drift away and now think of 60.

¶658:
¶659: AB: So I got a bit of like the 50’s kind of coming like this. And then, yeah and kind of seeing there is digits kind of down here. And then 60 sort of appearing bigger than the others. And then there is a line going off into the wall there so.
¶660:
¶661: TF: And what does that happen with the position of the 60?
¶662:
¶663: AB: So at the moment it's kind of just in front of the wall.
¶664:
¶665: TF: Right.
¶666:
¶667: AB: Kind of on the floor 6 0
¶668:
¶669: TF: Standing upright <questioning tone>
¶670:
¶671: AB: Yeah, standing upright.
¶672:
¶673: TF: Ok, so let’s try it the other way around now see if you notice any difference. So if I told you just think of the 60 to 70 and then try to just let them fade, now think of the 60.
¶674:
¶675: AB: So it's being a bit stubborn the 60. It's kind of staying here. So for a moment I got like the 60’s going like this across the corridor, but then when that kind faded away the 60 was still there.
¶676:
¶677: TF: Was it in the same place as before?
¶678:
¶679: AB: Yeah.
¶680:
¶681: TF: Ok so we’re
¶682:
¶683: AB: Ok so it's almost like, there’s almost like that 60 has rooted now because we’ve been talking about it for quite a bit and erm when I zoom, when I kind of think of the 60 as I get it and a line of digits kind of across the corridor with kind of 70 appearing over there.
¶684:
¶685: TF: What does the line of digits look like?
¶686:
¶687: AB: Erm I can just see all the numbers so 61 62 63 64 65 66 67 68
¶688:
¶689: TF: Ok so both digits are present <questioning tone>
¶690:
¶691: AB: Yeah.
¶692:
¶693: TF: Well since the 60 is so stubborn, maybe we can just jump really far right and say I don’t know, say 99 and just have a look at that area for a while and we’ll come back to the other one later.
¶694:
¶695: AB: So <pauses> so I’m sort of seeing it up in space. Sort of seeing 99 and then a 100 sort of kind of behind the 99 and then I’m seeing the 100 kind of coming over here.
¶696:
¶697: TF: Sort of turning back basically.
¶698:
¶699: AB: Yes, so feels on the verge of turning big corner.
¶700:
¶701: TF: How far is the 99?
¶702:
¶703: AB: Roughly from where I am sitting it's kind of there and that way <speaks quietly>
¶704:
¶705: TF: Ok, so it's, you are seeing it from a little bit of a distance or
¶706:
¶707: AB: Not that far away, but a little bit further perhaps than some of the others up there pulling forward a bit.
¶708:
¶709: TF: Ok, so what if I said can you please look at it from the other side, I mean looking from the 100 onwards down the number line. Can you switch your perspective?
¶710:
¶711: AB: So look back from 100. It’s tricky, please stabilise! <quiet> <inaudible> (50.58) something behind the line
¶712:
¶713: AB: <more inaudible utterances> like that (51.02)
¶714:
¶715: TF: So what did you have to do in order to change your perspective?
¶716:
¶717: AB: Erm, kind of rotate the image in front of me. So it's kind of over there and I kind of feel a 100 here and 101 there. Erm, yeah all the other numbers are kind of going down.
¶718: <Cross talking>
¶719:
¶720: TF: So you’re were taking
¶721:
¶722: AB: 90 is over there. And erm, I can kind of see this line that sort of zig zags around.
¶723:
¶724: TF: Ok so, now we can come back to the 60. Can you please have another look at the 60?
¶725:
¶726: AB: Yes, still there.
¶727:
¶728: TF: It never disappeared at all <questioning tone>
¶729:
¶730: AB: I wasn’t aware of it all this time.
¶731:
¶732: <laughter>
¶733:
¶734: AB: Erm, I wasn’t aware of the usual process of seeing it on the white page. It was just kind of still right there on the corridor.
¶735:
¶736: TF: I couldn’t trick it.
¶737: <laughter>
¶738:
¶739: AB: I can get a second 60 appearing over here but.
¶740:
¶741: TF: Really! <questioning tone>
¶742:
¶743: AB: Yeah.
¶744:
¶745: TF: Why is that? Why are there two now?
¶746:
¶747: AB: Just because thinking of 60.
¶748: <laughter>
¶749:
¶750: TF: Ok, we had that before remember when we had the lines
¶751: <cross talking>
¶752:
¶753: TF: If you look down the new 60, does it looks like it's in the same space as the old 60?
¶754:
¶755: AB: So it's on the white thing but then that goes away and then it's kind of now on the corridor, further down the corridor from this one.
¶756:
¶757: TF: Ok and if you look on the floor beneath it, what do you see?
¶758:
¶759: AB: Just floor
¶760:
¶761: TF: No number line <questioning tone>
¶762:
¶763: AB: No number line
¶764:
¶765: TF: Is there one under the original 60?
¶766:
¶767: AB: Erm, no.
¶768:
¶769: TF: Ok.
¶770:
¶771: AB: I can sort of, kind of sense it kind of behind the wall. Because the 60 is right against the wall and the number line would be there.
¶772:
¶773: TF: Ok, but it's out of view basically <questioning tone>
¶774:
¶775: AB: Yeah. It's out of view.
¶776:
¶777: TF: Ok, so was there any special number in this sequence between when I said to you have a look at 50 to 60?
¶778:
¶779: AB: Yes, so nothing popped out so yeah.
¶780:
¶781: TF: Nothing popped out <questioning tone>
¶782:
¶783: AB: Yeah, so I was maybe expecting 56 to be a bit different but it didn’t really, it didn’t look different.
¶784:
¶785: TF: If I said to you now have a closer look at 56 and let’s say bring out the 55 as well. Do they look different?
¶786:
¶787: AB: 56 seems to have some more elaborative pattern on it and 55 is looking a bit more boring
¶788:
¶789: TF: Can you have a closer look?
¶790:
¶791: AB: So they’re kind of down here the 60’s. Start and stop and their still there
¶792: <laughter and cross talking
¶793:
¶794: TF: Ok, let’s leave the 60 aside for now, let’s see whether we can have a closer look at why the 56 appears to be so special. So if you change your perspective so you can see the 56 a little bit closer. Maybe you can put it in front of you.
¶795:
¶796: AB: <inaudible utterances> (54.53)
¶797: <Laughter>
¶798:
¶799: AB: Maybe I can start again.
¶800:
¶801: TF: Ok if you want to have a short break and we’ll start with the 56 maybe ok.
¶802:
¶803: AB: Yeah
¶804:
¶805: TF: Yeah we’ve been going for a bit.
¶806:
¶807: AB: Can I have a sip of water?
¶808:
¶809: TF: Yeah sure.
¶810:
¶811: AB: Yeah by the time you start with a clean slate and then you get bogged up with loads of numbers! Well I seemed to eradicate the 3 3.14 and all that.
¶812:
¶813: TF: Yeah, but I couldn’t eradicate the 60 even when I tried to like lead you a little bit astray in the other direction
¶814:
¶815: AB: Yeah.
¶816:
¶817: TF: But maybe you had it in the back of your mind that it was
¶818:
¶819: <cross talking>
¶820: AB: My view it went back to the 3. That’s all still there as well.
¶821:
¶822: TF: Ok do you want to try it. Have a look at
¶823:
¶824: AB: Well, I caught a glimpse of it just then.
¶825: <laughter>

¶826:
¶827: TF: ok <laughter> So it's still hovering as well. But this is important to know for future sessions, because if you really want to have a pristine look at how these things appear then we can’t let the sessions go on too long because otherwise these other interferences will start having an effect. Ok, but maybe we can finish on 56 for today, just have another closer look at that one and then we’ll call it a day and do some more stuff next week. Are you ready to have another look?
¶828:
¶829: AB: Yeah. So I still caught a glimpse of 55 and 56 kind of fairly small over there, so I’ve got to see if I can bring it right in front of me. I can see it on a page.
¶830:
¶831: TF: Ok good.
¶832:
¶833: AB: 56 seems slightly decorated, like there’s kind of stripes in various places and like a white spot in the middle of it erm.
¶834:
¶835: TF: What colour are the stripes?
¶836:
¶837: AB: Erm just kind of black stripes and just kind of white underneath.
¶838:
¶839: TF: Underneath the stripe <questioning tone>
¶840:
¶841: AB: Yeah. Erm but nothing that stays really too stable. If I really try and focus I don’t know, I see a little white so it's kind of mainly black top bit of it. Erm, then I am seeing a little white circles so the 5 this is. The white circle missing from top corner as though someone has punched a hole out of it from the top corner.
¶842:
¶843: TF: If I draw a 5 for you can you indicated the hole? <external noise>
¶844:
¶845: AB: Just there.
¶846:
¶847: TF: Ok can you draw the 6 please as well next to it.
¶848:
¶849: AB: <Inaudible utterances> (57.55) It's quite hard to draw. It's not as fat as that.
¶850:
¶851: TF: Ok, does it have anything on it?
¶852:
¶853: AB: Erm <pauses to think> all kinds of marking, but they are kind of not really stable. It's almost like I see for a moment some stripes around here.
¶854:
¶855: TF: So these are black stripes
¶856:
¶857: AB: Yeah.
¶858:
¶859: TF: On a white background <questioning tone>
¶860:
¶861: AB: Yeah. And then a little bit of it here is black.
¶862:
¶863: TF: But the digit itself is black or <questioning tone>
¶864:
¶865: AB: Erm, so the outline is black.
¶866:
¶867: TF: What is the colour in the middle of the outline?
¶868:
¶869: AB: So it's black at the top and there’s kind of white bits <pauses> Not really stabilising very well, I mean sometimes I see bits of like stripes and it's hard to draw precisely.
¶870:
¶871: TF: Ok, and what about the 5 is it the same kind of patterning?
¶872:
¶873: AB: Yes.
¶874:
¶875: TF: So also stripes and
¶876:
¶877: AB: Kind of around the base, curvy bit
¶878:
¶879: TF: So the top bit is <questioning tone>
¶880:
¶881: AB: Is black
¶882:
¶883: TF: Ok. So the contour bits is the bit you filled in, with a little piece missing at the top. And then somewhere starting at the curvy bit, that’s when it starts. Ok you can continue.
¶884:
¶885: AB: From there. So that’s kind of black up to there. And then curvy bit and a few stripes around here. And the bottom is just white. There it's a bit unclear.
¶886:
¶887: TF: You mean the
¶888:
¶889: AB: Have to really kind of try and focus on one bit of the number or another. Apparently just see it all <cross talking>
¶890:
¶891: TF: Why is that? What do you see when you say you don’t see it clearly?
¶892:
¶893: AB: Just seems to just be moving around.
¶894:
¶895: TF: The digit itself <questioning tone>
¶896:
¶897: AB: Not the digit itself but the markings on it.
¶898:
¶899: TF: Ok, so sometimes there will be white at the bottom and sometimes there would not be white at the bottom.
¶900:
¶901: AB: Erm, yeah it's all white at the bottom when I lift and zoom in and there’s just a few stripes there and a few stripes down there. So
¶902:
¶903: TF: Put white over here.
¶904:
¶905: AB: Yeah and then there’s a few stripes there.
¶906:
¶907: TF: Does the 6 have any white in it?
¶908:
¶909: AB: 6 is white. The 6 is white. The hole in the middle of it is black. Again, there’s a few stripes going around the circular bit of it. Black stripes on the left hand side. Couple of stripes maybe kind of a black splodge around two o’clock around the circular bit of the 6.
¶910:
¶911: TF: Is it shifting around or is it becoming more stable?
¶912:
¶913: AB: Becoming more stable. Yeah, the black splodge is a bit of a mess really.
¶914:
¶915: TF: Can you make it more defined or is it resisting?
¶916:
¶917: AB: so it seems to be spilling onto the page around the 6, so it's kind of destroying the smoothness of the curve there.
¶918:
¶919: TF: so destroying the white page behind the number as well.
¶920:
¶921: AB: Yeah. There’s a black a hole in the middle of the circular bit.
¶922:
¶923: TF: So it doesn’t show the white through basically < questioning tone>

¶924: AB: No.
¶925:
¶926: TF: So the contour of the 6 is not solid.
¶927:
¶928: <pause>
¶929: AB: Yeah but white over here with black smudge and that kind of upper section of the circular bit.
¶930: <pause> Mainly white in the back.
¶931:
¶932: TF: Ok so, maybe we can try and understand where this is coming from by having a look at the area around it. I mean is the lighting different or is it the same as we had it when we had 60 for example?
¶933:
¶934: AB: Erm, it's kind of still on the page in front of me.
¶935:
¶936: TF: Ok so it hasn’t actually fallen down into its usual place here?
¶937:
¶938: AB: I can kind of see it over here as I still kind of, 60 is still here. 56 is still there. I kind of see it when it's down there. It's kind of a bit sort of like <inaudible> (1.04) it's not like purely black. There’s little white bits on it.
¶939:
¶940: TF: Right ok, what about the size in relation to the other numbers?

¶941: AB: Same size
¶942:
¶943: TF: Same size <questioning tone>
¶944:
¶945: AB: Yeah.
¶946:
¶947: TF: What about, can you see the number line?
¶948:
¶949: AB: There’s no line with the digits now, so 54 5 56 57. They’re all just standing out.
¶950:
¶951: TF: Can you make a number line appear?
¶952:
¶953: AB: Erm, yeah I can get flashes of it in between the numbers. There’s a broken line and gaps for each of the digits.
¶954:
¶955: TF: There’s gaps for each of the digits but only the 56 is opposite <questioning tone>
¶956:
¶957: AB: Yeah.
¶958:
¶959: TF: I mean you say gaps for each of the digits, do you mean each of the numbers or each of the digits?

¶960: AB: Each of the numbers.
¶961:
¶962: TF: Ok, do you want to explore anything else around the 56, anything that grabs your attention?
¶963:
¶964: AB: Erm, so we see if any of the other numbers around there have any of these sort hot marked features. I don’t really know how to that. It looked pretty black and 56 looks kind of more patterned than the others. Occasionally you get the slight stripe on like 57 but nothing as detailed on the 56.
¶965:
¶966: TF: So sometimes whatever is going on with the 56 spills over to the neighbouring numbers.
¶967:
¶968: AB: Yeah.
¶969:
¶970: TF: But the 56 is definitely the locus of this activity or where is it coming from?
¶971:
¶972: AB: Yeah, the 56 is the locus of this, it's sort of where, it's just got more of it than the others.
¶973:
¶974: TF: Ok
¶975:
¶976: <long pause>
¶977:
¶978: AB: So I can get kind of get white lines going across the numbers so it's kind of black, mainly black but sometimes you get whiteness fills the bottom bit of the number, kind of goes up to the top and then goes black again. So a shimmering effect.
¶979:
¶980: TF: Ok, so it's starts with white at the bottom of the numbers, then it becomes
¶981:
¶982: AB: Whilst you knew it just happened once now with the 59.
¶983:
¶984: TF: Oh ok, I thought you were talking about the 56.
¶985:
¶986: AF: Yeah. 56 is just kind of hot marked and a bit like mainly white, with these like black splodges and erm

¶987: TF: But it's also white at bottom and black at the top, like the 59 just was <questioning tone>
¶988:
¶989: AB: Erm, the 5 has more white at the bottom than at the top. The 6
¶990:
¶991: TF: Has the white on the neck
¶992:
¶993: AB: Yeah, more of, yeah and there’s this black splodge in the upper right bit of the sort of circular part.
¶994:
¶995: TF: Ok. So we haven’t seen anything like this in the lower numbers?
¶996:
¶997: AB: No. We had the 5 last time we were kind of shiny metal, with the more purpley face.
¶998:
¶999: TF: That was like a special 5
¶1000:
¶1001: AB: Special 5 added to the other one
¶1002: <laughter>
¶1003:
¶1004: TF: Ok, I think we can leave it there for this time and we’ll have another look next week.
¶1005:
¶1006: AB: yeah ok thanks.
¶1007:
¶1008: (1.51)
¶1009: **End of Interview.**
