# Supplementary material for: An extended case study on the phenomenology of sequence-space synesthesia
Source: Front Hum Neurosci. 2014 Jul 3;8:433. doi: 10.3389/fnhum.2014.00433 (PMC4080762; doi:10.3389/fnhum.2014.00433)
Supplement: Supplementary file 1 [file Presentation1.ZIP › Supp Mat 2.ZIP!/Referenced_Interview_Transcipts/AB - 20100305.doc]

¶1: **Name:** AB - 20100305

¶2:

**¶3: Description:** Dialogue between TF and AB on 05/03/2010

¶4:

¶5: (0.00)

¶6:

¶7: TF: Ok so maybe to start to bring us into the mood we can revisit some of our old friends from last week. So one of the particularly interesting numbers we looked at was pie. So can you think of pie please? Tell me what happens.

¶8:

¶9: AB: I’m right back in the scene from last week <laughter>

¶10:

¶11: TF: Ok can you give us maybe a rough description first of what you’re seeing right now.

¶12:

¶13: AB: So I’m seeing in this kind of erm slightly infernal corridor <laught

¶14:

¶15: ¶1:

**¶16: ¶2: Dialogue between TF and AB on 05/03/2010**
¶17: ¶3:
¶18: ¶4: (0.00)
¶19: ¶5:
¶20: ¶6: TF: Ok so maybe to start to bring us into the mood we can revisit some of our old friends from last week. So one of the particularly interesting numbers we looked at was pie. So can you think of pie please? Tell me what happens.
¶21: ¶7:
¶22: ¶8: AB: I’m right back in the scene from last week <laughter>
¶23: ¶9:
¶24: ¶10: TF: Ok can you give us maybe a rough description first of what you’re seeing right now.
¶25: ¶11:
¶26: ¶12: AB: So I’m seeing in this kind of erm slightly infernal corridor <laughter> and the pie equals 3.14159 and then kind intangible group of numbers going off towards this black hole <laughter>
¶27: ¶13:
¶28: ¶14: TF: Ok so you don’t notice anything different from last week or?
¶29: ¶15:
¶30: ¶16: AB: Think the 9 has gone into place as I now know for sure that’s the next digit and not the 2 that was kind of jostling for position.
¶31: ¶17:
¶32: ¶18: TF: Oh really! So what is the one after the 9 this time?
¶33: ¶19:
¶34: ¶20: AB: Don’t know as it's not very clear.
¶35: ¶21:
¶36: ¶22: TF: So there not even an option between two numbers as there was with the 9.
¶37: ¶23:
¶38: ¶24: AB: I think I’m just seeing kind of dot dot dot. And then there’s, I’ve got 3.14159 and then I’ve got this kind of a gap and then there’s kind of a dot dot dot kind of going slightly behind the numbers going towards the black hole. And then there’s sort of over here, sort of some digits just on the floor.
¶39: ¶25:
¶40: ¶26: TF: What do you see behind the background of the numbers?
¶41: ¶27:
¶42: ¶28: AB: Behind <questioning tone>
¶43: ¶29:
¶44: ¶30: TF: I mean, are they written on something?
¶45: ¶31:
¶46: ¶32: AB: No, they seem to just be transported straightaway into the corridor this time.
¶47: ¶33:
¶48: ¶34: TF: So another number that we looked at was 56. What do you see?
¶49: ¶35:
¶50: ¶36: AB: So I saw that kind of on a white background. And again it's got sort of patterning on it.
¶51: ¶37:
¶52: ¶38: TF: What happened to the white background?
¶53: ¶39:
¶54: ¶40: AB: Erm, seems to be pretty much still there.
¶55: ¶41:
¶56: ¶42: TF: Still there.
¶57: ¶43:
¶58: ¶44: AB: I can kind of let it melt away a bit from the bottom. Start think about whether 56 goes in relation to the other numbers.
¶59: ¶45:
¶60: ¶46: TF: So if you bring in let’s say 55, what effect does that have?
¶61: ¶47:
¶62: ¶48: AB: Has, 56 is here and I have a sort of vague sense of the 55 belonging here. Erm, but it's not that stable right now.
¶63: ¶49:
¶64: ¶50: TF: What happened to the white background?
¶65: ¶51:
¶66: ¶52: AB: It’s kind of melting away from the bottom upwards. There’s still a little bit of it at the top.
¶67: ¶53:
¶68: ¶54: TF: So you said there was a patterning in the numbers. Can you have a closer look at what the patterning looks like?
¶69: ¶55:
¶70: ¶56: AB: So it's like a white hole in the top left corner still. And it's like someone has punched a hole out of the 5. Well it's kind of like a white spot really.
¶71: ¶57:
¶72: ¶58: TF: And do you see any stripes or other markings?
¶73: ¶59:
¶74: ¶60: AB: Yeah, a few stripings around the middle of the curvy part of the 5.
¶75: ¶61:
¶76: ¶62: TF: What colour are the numbers then?
¶77: ¶63:
¶78: ¶64: AB: Erm the 56 is black and a few sort of white markings on it.
¶79: ¶65:
¶80: ¶66: TF: Do you notice any other differences between this number and the 55?
¶81: ¶67:
¶82: ¶68: AB: So the 55 is much smaller, black, totally black. So I’ve kind of got a 55 kind of down here and 56 up here. 56 is much bigger.
¶83: ¶69:
¶84: ¶70: TF: And what if you move to the next number <questioning tone>
¶85: ¶71:
¶86: ¶72: AB: And 57 is kind of over here. So the number line going here. 56 is kind of above the number line in big. 57 is sort of lower down and smaller as well, just like the 55
¶87: ¶73:
¶88: ¶74: TF: So if you would lower your
¶89: ¶75:
¶90: ¶76: AB: Same with 58 and 59 has let my attention wonder up the line.
¶91: ¶77:
¶92: ¶78: TF: Ok so you’ve mentioned the number line. What does it look like underneath the 56?
¶93: ¶79:
¶94: ¶80: AB: It's a little bit unstable. Sometimes I’m seeing I’m seeing sort of digits so there’s 55 here. There’s kind of a line. Yeah, I guess the digits are kind of to the left of the line, well it's not really very stable. There’s kind of a line underneath the 56 and then you got 57 and 58. So actually there’s only a line between 55 and 57, so 56 is kind of above and then you got the 55 here, 57 written there. 58 kind of standing up 59.
¶95: ¶81:
¶96: ¶82: TF: What happens for the 56 within the line? You said the line connects 55 to 57
¶97: ¶83:
¶98: ¶84: AB: 55 to 57. 56 is kind of hovering above.
¶99: ¶85:
¶100: ¶86: TF: Are you saying that the numbers 55 and 57 are closer, like lower down on the ground?
¶101: ¶87:
¶102: ¶88: AB: Yeah, they are on the ground.
¶103: ¶89:
¶104: ¶90: TF: And the 56 is not on the ground <questioning tone>
¶105: ¶91:
¶106: ¶92: AB: No. I can get a small 56 appearing briefly on the ground.
¶107: ¶93:
¶108: ¶94: TF: You mean in the sequence, matching the sequence?
¶109: ¶95:
¶110: ¶96: AB: Yeah, but there’s still a big 56 hovering above as well. With these kinds of happenings on it.
¶111: ¶97:
¶112: ¶98: TF: Let’s see. So do you see any relationship between the 56 and the other smaller numbers you called into your vision?
¶113: ¶99:
¶114: ¶100: AB: Just slots in between 55 and 57.
¶115: ¶101:
¶116: ¶102: TF: But you said there could be another one down there. So is it distinct from the number line that is that’s going on in the bottom?
¶117: ¶103:
¶118: ¶104: AB: The big 56. Erm, so it's almost like the small one is being magnified. Don’t know. The small one doesn’t really have the markings on it.
¶119: ¶105:
¶120: ¶106: TF: And there’s no gaps in the number line either?
¶121: ¶107:
¶122: ¶108: AB: Erm, I don’t actually see a line between any of the other numbers. Only between 55 and 57. But when 56, it's kind of oscillating a bit between having a line between 55 and 57 and having a little 56 in there and 56 appears as the line kind of goes away.
¶123: ¶109:
¶124: ¶110: TF: So, in terms of where these numbers are located what do you see in the surroundings?
¶125: ¶111:
¶126: ¶112: AB: Erm, again it's this kind of, this colour I’m not quite sure what it is. This greeny orangey colour.
¶127: ¶113:
¶128: ¶114: TF: Ok, let’s try something new, just want to see whether you can match the colour that you’re trying to describe with the colour that you see when you close your eyes. Try closing your eyes and see what the colour is. And now if you think of the corridor, if you recall the corridor you’ve seen.
¶129: ¶115:
¶130: ¶116: AB: They only give me one colour when I close my eyes. I think I saw the colour for a little bit and then there was kind of red and then there was black.
¶131: ¶117:
¶132: ¶118: TF: Ok, just trying to see whether there was the same mixture of colours as it could have, but it's not.
¶133: ¶119:
¶134: ¶120: AB: I didn’t really get one stable colour .
¶135: ¶121:
¶136: ¶122: <cross talking – unclear> (9.14)
¶137: ¶123:
¶138: ¶124: TF: One stable colour.
¶139: ¶125:
¶140: ¶126: AB: It is one of the ones that was kind of in that mix.
¶141: ¶127:
¶142: ¶128: TF: Ok so.
¶143: ¶129:
¶144: ¶130: AB: So it appeared for a moment and then red appeared for a moment and then black appeared for a moment.
¶145: ¶131:
¶146: ¶132: TF: Right. Ok, so the difficulties in describing the colours, not that the colour itself is somehow undefined or unstable.
¶147: ¶133:
¶148: ¶134: AB: No, the colour seems pretty stable. There was, when I was thinking of pie it was kind of, I dunno, it was like a sort of black hole was kind of causing it to have more of a kind of slightly darker, darker kind of glow. Seemed more kind in the underworld. Erm, than the rest of the number line. It was a sort of more cheerful sort of colour, more sort of greens in there I think.
¶149: ¶135:
¶150: ¶136: TF: Right.
¶151: ¶137:
¶152: ¶138: AB: With these kind of white lights at either end.
¶153: ¶139:
¶154: ¶140: TF: Ok so at the moment we were talking about the 56. The lighting conditions are the same as let’s say, if you say think of 5. There’s no difference in the environment between the two numbers. It's just the number itself that looks different. So just to explore a little bit, how what you see relates to, let’s say the normal visual perception. What happens when you try to think of these numbers and visualise the scene when you close your eyes? So if I tell you now 56, what happens?
¶155: ¶141:
¶156: ¶142: AB: So the pattern 5 6 on a white background.
¶157: ¶143:
¶158: ¶144: TF: Do you notice any change in the colouration, or the lighting?
¶159: ¶145:
¶160: ¶146: AB: Saw a few spots on the 6, kind of around the sort of neck of the 6. Kind of patches of sort of stripes.
¶161: ¶147:
¶162: ¶148: TF: But you see the numbers just as vividly as you would with your eyes open.
¶163: ¶149:
¶164: ¶150: AB: Yeah.
¶165: ¶151:
¶166: ¶152: TF: What about the background. Can you see the corridor in which the numbers are?
¶167: ¶153:
¶168: ¶154: AB: Erm, staying more on a white background 56 perhaps. Erm, I can the other numbers kind of below. A 56 is kind of up here on the white background. White background is kind of melting away again from the bottom. So I’m going to look down towards the bottom of the 5 and 6 and then it's against that kind of greeny orangey background. Little bit more stubborn in the top left corner, it kind of wants to be behind a white background. But I can kind of make it pretty much go away.
¶169: ¶155:
¶170: ¶156: TF: How do you do that?
¶171: ¶157:
¶172: ¶158: AB: <pauses> Just like I’m with it I guess. I think if I look down at the number line and then back up at the 56. It kind of as I look up the white background kind of sort of lifts.
¶173: ¶159:
¶174: ¶160: TF: So does the direction of your gaze, if I understand it correctly, if you trace it up from the bottom where the number line is or supposed to be, up towards the 56 and then trace it on 56. As your gaze progresses the white background disappears.
¶175: ¶161:
¶176: ¶162: AB: Yeah. And as I soon as I bring my gaze, from say above the 56 then it's kind of like a white curtain comes down behind it.
¶177: ¶163:
¶178: ¶164: TF: Ok so let’s try that out with another number. So if you bring in the 55 from the side. What do you see?
¶179: ¶165:
¶180: ¶166: AB: So 55 is down here, down below on the number line.
¶181: ¶167:
¶182: ¶168: TF: Can you bring it up?
¶183: ¶169:
¶184: ¶170: AB: <pauses> it's kind of fighting with the 56.
¶185: ¶171:
¶186: ¶172: TF: Ok what if you go down to it instead. Is that possible? Can you shift your prespective?
¶187: ¶173:
¶188: ¶174: AB: Quite hard. Erm, dunno if it would be easier if I moved or.
¶189: ¶175:
¶190: ¶176: TF: I mean, if it's easier, maybe with the eyes open. Try that as well.
¶191: ¶177:
¶192: ¶178: AB: Erm, so they are quite small the rest of them.
¶193: ¶179:
¶194: ¶180: TF: Actually when you did just that, when you just opened your eyes, the scene stayed exactly the same
¶195: ¶181:
¶196: ¶182: AB: Erm, no noticeable differences.
¶197: ¶183:
¶198: ¶184: TF: So when you were looking at the 55 eyes closed and you open them up, same location
¶199: ¶185:
¶200: ¶186: AB: Same location right here.
¶201: ¶187:
¶202: ¶188: TF: Same appearance
¶203: ¶189:
¶204: ¶190: AB: Yeah.
¶205: ¶191:
¶206: ¶192: TF: Because this is something else we could also explore a little bit. How does the 55 relate to it's, say or do you perceive as the table. Is it hovering above it?
¶207: ¶193:
¶208: ¶194: AB: Seems independent of the table. It's like the table just isn’t there when I am paying attention only on the 55.
¶209: ¶195:
¶210: ¶196: TF: So if you look at the 55. So where is it?
¶211: ¶197:
¶212: ¶198: (16.10)
¶213: ¶199: AB: Erm, I guess it's kind of roughly on the table <unclear> location.
¶214: ¶200:
¶215: ¶201: TF: So, the numbers are not going through the table are they?
¶216: ¶202:
¶217: ¶203: AB: Very hard to say exactly. I don’t know if I sat back a bit and erm and have the freedom to move my arm a bit, now and under the table. <laughter>
¶218: ¶204:
¶219: ¶205: TF: If you want to stand up and see what effect it has, go ahead.
¶220: ¶206:
¶221: ¶207: AB: Yeah. Stand up.
¶222: ¶208:
¶223: ¶209: TF: Has their location changed at all?
¶224: ¶210:
¶225: ¶211: AB: Yeah, I’m kind of more above them now.
¶226: ¶212:
¶227: ¶213: TF: But the location of the 55 has remained the same <questioning tone>
¶228: ¶214:
¶229: ¶215: AB: Yeah.
¶230: ¶216:
¶231: ¶217: TF: Still on top of the table there <questioning tone>
¶232: ¶218:
¶233: ¶219: AB: So it's either on top of the table or perhaps slightly below the surface of the table. Err hard to tell.
¶234: ¶220:
¶235: ¶221: TF: If you move a little bit to the side, the numbers stay in the same location <questioning tone>
¶236: ¶222:
¶237: ¶223: AB: The numbers are over there. I can bring them over here.
¶238: ¶224:
¶239: ¶225: TF: Ok, can you do that?
¶240: ¶226:
¶241: ¶227: AB: So I’ve just brought them over there.
¶242: ¶228:
¶243: ¶229: TF: What did you do in order to do that?
¶244: ¶230:
¶245: ¶231: AB: Erm, dunno. Just wanted them to be a bit closer <laughter>
¶246: ¶232:
¶247: ¶233: TF: So how are they relating will the wall then?
¶248: ¶234:
¶249: ¶235: AB: So 54 is about here, 55 56 57 58
¶250: ¶236:
¶251: ¶237: TF: Ok and can you move them to be in the middle of the space because that’s like again. So if I said for example can you put a 55.
¶252: ¶238:
¶253: ¶239: AB: So I’ve got one lot over here and one lot over there.
¶254: ¶240:
¶255: ¶241: TF: Are there still remains over here?
¶256: ¶242:
¶257: ¶243: AB: Yeah. But look over there, there’s some numbers over there.
¶258: ¶244:
¶259: ¶245: TF: Do you mind if I test how accurately you can place them? <AB agrees> What if I say put a 55 in my hand?
¶260: ¶246:
¶261: ¶247: AB: <laughs>
¶262: ¶248:
¶263: ¶249: TF: Can you do it or shift the ones?
¶264: ¶250:
¶265: ¶251: AB: I saw it appear for a moment, but <pauses to think>. It's little bit weird because I don’t see them relating to your hand.
¶266: ¶252:
¶267: ¶253: TF: Is it easier maybe to shift the ones from the wall over or?
¶268: ¶254:
¶269: ¶255: AB: I get a bit of a 55 appearing on your hand. There’s still a number line over here and a number line over there.
¶270: ¶256:
¶271: ¶257: TF: So it would be interesting to see what happens if you remove the support, I mean that’s why I put my hand and you see a 55 on top of it. Can you see it now? <AB agreeing throughout>
¶272: ¶258:
¶273: ¶259: AB: Not very clear. For a moment it was like hovering about there.
¶274: ¶260:
¶275: ¶261: TF: So it would be interesting to then remove it to then see whether it stays in the air or whether it's somewhere related to a <cross talking>
¶276: ¶262:
¶277: ¶263: AB: Kind of got a 55 about there.
¶278: ¶264:
¶279: ¶265: TF: So your bodily movements don’t seem to affect their position <questioning tone>
¶280: ¶266:
¶281: ¶267: AB: Erm, there’s some small numbers over here.
¶282: ¶268:
¶283: ¶269: TF: What if you. Ok let’s just say if you take them out of view completely so if you look behind you so immediately you don’t see either of them and then you turn around again. Do they remain exactly where they were before?
¶284: ¶270:
¶285: ¶271: AB: Yeah still 5 6 57. I sense a 54 here, but weaker this one though.
¶286: ¶272:
¶287: ¶273: TF: So maybe we can explore a little bit more what you did in order to
¶288: ¶274:
¶289: ¶275: AB: Reappearing though.
¶290: ¶276:
¶291: ¶277: TF: to create a new number line there. So it wasn’t easy to do it from my hand. Could you try it at another target that you think would be suitable to create a different number line. And then when you do it just try to pay attention and become aware of the sequence of events as it happens. Maybe over there for example, if you say 55 over in that direction <AB agreeing throughout>

¶292: ¶278:
¶293: ¶279: AB: Yeah, I’m kind of get it against the white background. It's a little bit further away from me.
¶294: ¶280:
¶295: ¶281: TF: Where did they appear?
¶296: ¶282:
¶297: ¶283: AB: Sort of about here.
¶298: ¶284:
¶299: ¶285: TF: So do you have any idea why that particular location, do you have any feeling or sensation of where they were going to be?
¶300: ¶286:
¶301: ¶287: AB: We’re aiming to get one over here.
¶302: ¶288:
¶303: ¶289: TF: Yes sure. I was saying specifically or you just waited for them to appear?
¶304: ¶290:
¶305: ¶291: AB: I just faced this direction and then thought of 55. A little bit more colourful this time than the 55.
¶306: ¶292:
¶307: ¶293: TF: The 55 looks more colourful <questioning tone>
¶308: ¶294:
¶309: ¶295: AB: Yeah.
¶310: ¶296:
¶311: ¶297: TF: Ok have another closer look at it. Could you describe it please?
¶312: ¶298:
¶313: ¶299: AB: So there were little bits of sort of patterns and greens on it. So it's mainly black, just around the edges there’s bits of patterns and greens.
¶314: ¶300:
¶315: ¶301: TF: Ok
¶316: ¶302:
¶317: ¶303: AB: I feel the number line kind of like much bigger this time.
¶318: ¶304:
¶319: ¶305: TF: So it's sitting on a number line somehow.
¶320: ¶306:
¶321: ¶307: AB: Yeah 54
¶322: ¶308: <interrupted by another speaker>
¶323: ¶309:
¶324: ¶310: Other speaker: Can I, I think you asked for a. When you turn away to say the first time and turn back. When you’re turning away are you aware that the number stays there even you’re not floating?
¶325: ¶311:
¶326: ¶312: AB: Yeah, if I think about it I am aware of its presence over there.
¶327: ¶313:
¶328: ¶314: Other speaker: So you would expect me to see if you moved your head back you would have some expectations it's still there <AB agreeing throughout>
¶329: ¶315:
¶330: ¶316:
¶331: ¶317: TF: So when you were looking at that number and describing it's colours, were you aware of any sensations coming in from the two number lines that are out of your view?
¶332: ¶318:
¶333: ¶319: AB: I wasn’t aware of those at all. But if I pay attention to those and small numbers down here there’s a bigger 54 here.
¶334: ¶320:
¶335: ¶321: Other speaker: Does paying attention change the size of the numbers that you see?
¶336: ¶322:
¶337: ¶323: AB: Erm, if I focus my attention on these ones, they’re quite small at the moment.
¶338: ¶324:
¶339: ¶325: Other speaker: Do they get smaller or do they get bigger?
¶340: ¶326:
¶341: ¶327: AB: I think these are, so what did seem to disappear was the big 56 that was hovering above it. Instead I’ve just got small numbers down here now.

¶342: ¶328:
¶343: ¶329: Other speaker: So paying attention down there made the non attended look larger numbers to go away a bit.
¶344: ¶330:
¶345: ¶331: (24.26)
¶346: ¶332: AB: Yeah. But now I’ve just kind of remembered the 56 that’s kind of here. <inaudible> But I didn’t notice the presence of the 56 just now. I was only aware of those for a moment.
¶347: ¶333:
¶348: ¶334: TF: What other thing we could have looked at is another irrational number. Can you suggest one?
¶349: ¶335:
¶350: ¶336: AB: So e was briefly mentioned in the pub the other night. It seemed like for that one I was getting more of a picture of a sort of exponential curve coming in. Yeah, it is a number I didn’t encounter until I was a lot older. <laughter>
¶351: ¶337:
¶352: ¶338: TF: Well what about.
¶353: ¶339:
¶354: ¶340: AB: I knew about when I was pretty young.
¶355: ¶341:
¶356: ¶342: TF: Ok so what if I say 1/3, how does that appear?
¶357: ¶343:
¶358: ¶344: AB: So there I kind of, it appeared as 1 over 3. Not quite sure of the order of how things appeared but final image was 0 then 1/3 was quite close to 1/2 and then 1 is over here. So all those numbers appeared.
¶359: ¶345:
¶360: ¶346: TF: So can you see them in the other indentation, not as a fraction but as a
¶361: ¶347:
¶362: ¶348: AB: So I can see a .333 maybe something intangible going further away from me like the 5, kind of on top of the 1 over 3.
¶363: ¶349:
¶364: ¶350: TF: So if you focus on the .333 what happens towards the end of that sequence? How far can you see it?
¶365: ¶351:
¶366: ¶352: AB: How many 3’s. Hard to tell, they’re kind of like getting smaller and smaller and going off into the distance.
¶367: ¶353:
¶368: ¶354: TF: Where are they going? Are they going?
¶369: ¶355:
¶370: ¶356: AB: so sort of 0.3 slightly to the left of the 1 over 3 and then there’s a, then they get smaller and end up very close to the 1 of the 1 over 3 and then they are going off sort of diagonally to the right and behind of the 1, into the distance.
¶371: ¶357:
¶372: ¶358: TF: Do you see where they end? Where is this distance leading to?
¶373: ¶359:
¶374: ¶360: AB: There’s another kind of like black square, then sort of going off to
¶375: ¶361:
¶376: ¶362: TF: Black square <questioning tone> What about any of the lighting conditions that you described in terms of pie. Anything like that going on?
¶377: ¶363:
¶378: ¶364: AB: I’m just aware of white and black at the moment.
¶379: ¶365:
¶380: ¶366: TF: Ok so it doesn’t have the same atmosphere?
¶381: ¶367:
¶382: ¶368: AB: No.
¶383: ¶369:
¶384: ¶370: TF: There’s one more thing I want to try, just to jump back to previously. Close your eyes please and now think about favourite number, number 5. Do you see it?
¶385: ¶371:
¶386: ¶372: AB: Takes a little while. Sense a 5 just over here. It's a little bit, it's a bit of an unstable image at the moment.
¶387: ¶373:
¶388: ¶374: TF: Can you try to concentrate on it?
¶389: ¶375:
¶390: ¶376: AB: Yeah. This time it's kind of gone a little bit. A bit distorted the shape.
¶391: ¶377:
¶392: ¶378: TF: You mean the contours?
¶393: ¶379:
¶394: ¶380: AB: Yeah it's not so well formed, so there’s like, so the top is sort of normal, so the neck going down is a bit short and then curve is kind of elongated. There’s a bit of purple and red on it. It's oscillating a bit. I’m kind of getting glimpses of a better formed 5.
¶395: ¶381:
¶396: ¶382: TF: And in the same location?

¶397: ¶383: AB: Yeah.
¶398: ¶384:
¶399: ¶385: TF: Ok if you open up your eyes now. What happens to the 5?
¶400: ¶386:
¶401: ¶387: AB: Seems pretty much the same.
¶402: ¶388:
¶403: ¶389: TF: Is it in the location where you expect it to be?
¶404: ¶390:
¶405: ¶391: AB: Yes, here. So what’s happening in relation to the table because when I’m closer to the table, seemed like they were nearer. Because now they are a bit further than arms reach
¶406: ¶392:
¶407: ¶393: TF: But still over the table <questioning tone>
¶408: ¶394:
¶409: ¶395: AB: Yes about here.
¶410: ¶396:
¶411: ¶397: TF: Ok, I think let’s leave it there for this week. Thanks.

¶412: ¶398: **End of interview.**
