# Supplementary material for: An extended case study on the phenomenology of sequence-space synesthesia
Source: Front Hum Neurosci. 2014 Jul 3;8:433. doi: 10.3389/fnhum.2014.00433 (PMC4080762; doi:10.3389/fnhum.2014.00433)
Supplement: Supplementary file 1 [file Presentation1.ZIP › Supp Mat 2.ZIP!/Referenced_Interview_Transcipts/AB - 20100320a.doc]

**Name:** AB - 20100320a

**Description:** Dialogue between TF and AB on 20a/3/2010

¶1: **Dialogue between TF and AB on 20a/3/2010**

¶2: (0.00)

¶3: TF: Ok to begin this session, something that we wondered about last time. Did you notice any numbers when you came into this room?
¶4:
¶5: AB: No.
¶6:
¶7: TF: So none of the stuff that we were talking about 2 weeks hung around basically?
¶8:
¶9: AB: No, erm maybe I can remember there was something here.
¶10:
¶11: TF: But it's a memory, it’s not a visual presence.
¶12:
¶13: AB: Erm, so I start to think about it like 54 55 56. Hard to tell if it's just a memory or if it's really there.

¶14: TF: Well have a look at the wall.
¶15:
¶16: AB: I remember something being over there as well. Can’t remember what number it was whether it was a 55 or a 56. Yes, so there’s obviously nothing there that’s sort of firm there. Kind of got a bit of an image of a big 55 there then a big 56 sort of stuck on the wall there. Nothing really that stable.

¶17: TF: I think let’s leave it at that for a while. So we wanted to look at some irrational numbers. So what happens if you think of the square root of 2?
¶18:
¶19: AB: So I saw a briefly flashing the square root of 2 equals 1.41 and then like there’s a whole load of other numbers to the right, which are not really settling down. Just seem really random, there’s a like kind of a 2 8 7 6 5 4, but that’s not the correct expression of it.
¶20:
¶21: TF: Ok so to recap, when you thought of the square of 2, you saw the symbol for the square root with the 2 underneath it and the equal sign and then it said <questioning tone>
¶22:
¶23: AB: And then 1.41. I am seeing 2 8 7 6 5 now. Not sure if those are correct numbers.
¶24:
¶25: TF: What do you see after the 5?
¶26:
¶27: AB: It’s kind of dot dot dot, and then there’s kind of some rather blurry numbers to the right in a crowd.
¶28:
¶29: TF: Oh so it's a collection of numbers again.
¶30:
¶31: AB: Yeah
¶32:
¶33: (3.04)
¶34: TF: They have been described <inaudible word> for high?
¶35:
¶36: AB: This time they are all kind of on the page, kind of all crowding around each other, much sort of closer together.
¶37:
¶38: TF: And so in all of this you had the white background present?
¶39:
¶40: AB: Yeah.
¶41:
¶42: TF: When you focus on the background what happens?
¶43:
¶44: AB: Erm, kind of got the same kind of coloured background I usually have. Kind of like the slightly lighter one not the one I had for pie. Sort of black ink, against the sort of greeny orangey background.
¶45:
¶46: TF: Can you describe the black spot?
¶47:
¶48: AB: The?
¶49:
¶50: TF: The black spot.
¶51:
¶52: AB: Black spot <questioning tone>
¶53:
¶54: TF: Did you say there was a?
¶55:
¶56: AB: No I didn’t see a black spot at the moment. Or I am starting to become aware of it over here so root two equals, no numbers and then there’s kind of a black spot here.
¶57:
¶58: TF: Ah ok. It must have been 30 I was anticipating. So describe to me the sequence again, you were looking at the background and what happened?
¶59:
¶60: AB: So I’ve got root 2 equals 1.4128765. I saw a 4 and then a 2 above the 4 and I see dot dot dot. Then I’ve got a crowd of numbers, some 6’s and 5’s in this crowd. And then there’s kind of like a black hole to the right of that crowd.
¶61:
¶62: TF: What is the relationship between the crowd and the black hole?
¶63:
¶64: AB: Erm, just to the left to the black hole.
¶65:
¶66: TF: So just a spatial relationship <questioning tone>
¶67:
¶68: AB: Yeah.
¶69:
¶70: TF: Do you see any movement?
¶71:
¶72: AB: No movement.
¶73:
¶74: TF: So the crowd is still?
¶75:
¶76: AB: Erm, yeah it's kind of like a black sludge. They are all kind of overlapping the numbers. <Sneezes> I’m sort of aware of the presence of mainly a couple of 6’s and a couple of 5’s.
¶77:
¶78: TF: So you said the lighting condition is lighter, the colours lighter. Is that because of the light?
¶79:
¶80: AB: Erm, don’t know it's kind of sort of orangey colour.
¶81:
¶82: TF: But it doesn’t have any of the gloominess that the pie had.

¶83: AB: Erm no, it seems a bit lighter than the pie. And it's still kind of floating in the middle of space <external noise>
¶84:
¶85: TF: So it hasn’t settled down?
¶86:
¶87: AB: Doesn’t seem to be on the floor this time. Nor just kind of against a wall.
¶88:
¶89: TF: So what’s on the floor of that wall?
¶90:
¶91: AB: Erm, now I see 1 2 like this, number 1, number 2. The square root of 2 is kind of floating above in the middle of the room.
¶92:
¶93: TF: But it would fit below?
¶94:
¶95: AB: Erm, so you’ve got 1 here and 2. Number 1, number 2 and then the square root of 2 is coming across like this, although it's up just here.

¶96: TF: Ok, it could fit down there if it wants to?
¶97:
¶98: AB: It could fit down there because it's kind of horizontal. The number line is kind of like that.
¶99:
¶100: TF: And so the wall that you were describing was also cutting across the number line <questioning tone>
¶101:
¶102: AB: Erm yeah, exactly.
¶103:
¶104: TF: Do you see any?
¶105:
¶106: AB: It's kind of dropped a little. It’s kind of fluctuated between being up here and down there. I saw it for a moment dropped.
¶107:
¶108: TF: But it's still once in a while going up back again?
¶109:
¶110: AB: Seems to be more down now.
¶111:
¶112: TF: Did you actually see it moving down or did it just appear at the bottom?
¶113:
¶114: AB: Just appeared at the bottom as I was focusing on the number 1 and number 2.
¶115:
¶116: TF: What does the spot on the number line look like?
¶117:
¶118: AB: Just a gap in the line. Oh I see the line, just a gap and more line and just comes in here.

¶119:
¶120: TF: Ok, if you let me try to draw you what you are saying.
¶121:
¶122: <Pause>
¶123:
¶124: TF: So we have a 1 and a 2 with a number line and then I got cutting across it where there is a gap in the number line, we have square root of 2 equals 1.4128765 and then there was a 4 with a 2 floating above, and then we have dot dot dot and a collection of
¶125:
¶126: AB: yeah, except the line is going that way
¶127:
¶128: TF: Which one?
¶129:
¶130: AB: The number 1 and number 2 should be more like that.
¶131:
¶132: TF: Oh sorry I was drawing it from my perspective.

¶133: AB: Right ok. So this would be more like that. Oh no, hang on, yes like this. Sorry the root of 2 equals blah blah, more like that.
¶134:
¶135: TF: In that direction. About the wall, does it have all the features that you usually see, like having lights on the sides?
¶136:
¶137: AB: Erm, think there’s a white light on the corridor
¶138:
¶139: TF: Ok shall we try another one. What about E?
¶140:
¶141: AB: So I’ve got a flash of the exponential curve and then I saw the letter E, it took a while for the equal sign and numbers to appear but then they are there.
¶142:
¶143: TF: Ok so which numbers can you see?

¶144: AB: 2.78 erm 1828 and the dot dot dot and there’s another 1828 kind of floating behind, kind of a dot dot dot as well. I’m getting little circles coming up in the back like little complex numbers appearing and little circle unit radius appearing there intermittently as well. Circle representing E is to imaginary powers. That’s from over there and then there’s an E equals 2.781828 here. There’s kind of an exponential curve over there. <Quietly spoken>
¶145:
¶146: TF: Ok let’s focus on the numbers first. Or actually let me just try to recap the sequence of events. So, when I told you to think of the number E, the first thing you noticed was flash of the exponential graph and then the E and then it took a while for the equals sign to appear, and then the numbers appeared or did they appear the same time as the equals sign? <AB agreeing throughout>
¶147:
¶148: AB: Erm, as I just kind of moved my attention from left to right I became sort of aware of them and could see them.

¶149:
¶150: TF: So your attention was revealing the numbers as you moved along the, was it the page?
¶151:
¶152: AB: Yeah, it's on a page. Seems to be more floating against a greenish, orangey background again actually. Kind of a lighter more towards the green this time. Yeah, black ink.
¶153:
¶154: TF: Ok so that’s interesting. So the E appears and then the equals sign and so the numbers come into view if you move your attention rightwards. So, what was there before you moved your attention there?
¶155:
¶156: AB: Erm, so if I focus my attention on E <pauses> Well I’m just aware of numbers but I can’t really say what numbers. I’m only seeing the 2 and maybe the 7 but after, as soon as I focus on them then they kind of, loose my attention <voice trails off>
¶157:
¶158: TF: Right yes. It's difficult even with physical objects, say if I focus on the object in front of me the chair. I can stay focused there and can be aware of the say the periphery. Can you do that with the numbers?
¶159:
¶160: AB: Erm, seems very hard without moving my eyes. Like I’m focusing my eyes on the E. Seems like I am little eye movements across to read the other numbers
¶161:
¶162: TF: I didn’t see any eye movements. Try again, try with an object first. Look at your bottle in front of you and then say keep your eyes focused on the blue cap on the top. Can you see the colours of things that around it without shifting your view away from the top?
¶163:
¶164: AB: Yeah, I can see sort of yellow things on the wall.
¶165:
¶166: TF: So in this case you don’t have difficulty seeing objects around the bottle cap, while keeping your eye focused on the cap.
¶167:
¶168: AB: No, not that easy. Depends how focused I am on the bottle cap.
¶169:
¶170: TF: Ok, just as a comparison and turn your attention back to the E and try doing the similar trick.
¶171:
¶172: AB: Yeah, it gets more and more vague the further away I go.
¶173:
¶174: TF: Can you see 7 8
¶175:
¶176: AB: I see equals and the 2.7, but I can’t really see beyond that really clearly.

¶177:
¶178: TF: Ok in principal there’s no difference between the two experiences would you say?
¶179:
¶180: AB: I think there may be a little bit of movement of the number as I move my attention down, so literally have to move my eyes.
¶181:
¶182: TF: Can you describe that movement.
¶183:
¶184: AB: <pauses> Seems like if I move my head the whole numbers moving in synch like in the direction of my head. I’m kind of right in front of me is the 7. So I’ve kind of got to move to my left to see the E. It's quite hard to have the E in the middle my focus. It seems like I move it back to the equal sign.
¶185:
¶186: TF: So which one is the most natural focal point?
¶187:
¶188: AB: So maybe the equal sign.
¶189:
¶190: TF: Ok so if you try focusing on the equal sign, just like you did with the bottle cap. Then could you please describe like the peripheral feel around without moving your attention from the equal sign.
¶191:
¶192: AB: The E to the left and 2 to the right.
¶193:
¶194: TF: Ok now let’s try another <inaudible word> ?challenge, so keep your attention focused on the equal sign. Can you see what is in the room around the equals sign, while keeping your attention focused on it?
¶195:
¶196: AB: Very hard to keep my attention totally focused. There’s kind of a greeny, orangey colour. I don’t see any details, just kind of pure kind of background.
¶197:
¶198: TF: But it doesn’t have anything of the physical room or presence?
¶199:
¶200: AB: No
¶201:
¶202: TF: So when you focus on the equal sign, you say you see the green background. While focusing on the equal sign and being aware of the green background, are you also aware of where the green background ends and the physical room begins?
¶203:
¶204: AB: Erm, yeah the green background is kind of about here.
¶205:
¶206: TF: So it has a definite size? <AB agrees> So ok, let’s explore that space.
¶207:
¶208: AB: Hard to say if it's just kind of a mist at the end of my visibility or if it's definitely a wall.

¶209: TF: Let’s just briefly explore the boundary of this mist or a wall. So if you move your attention to say the top order, how does it interact with the physical room and the edge? Can you see what’s behind it?

¶210: AB: Erm, well I can just alternate my attention between the physical room and the mental room I’ve got.
¶211:
¶212: TF: But there’s no overlapping between the two?
¶213:
¶214: AB: No.
¶215:
¶216: TF: So if you focus your attention on the green mist or wall, your visual awareness of the room fades out, the physical room?
¶217:
¶218: AB: Yeah. It never leaves me completely, the physical room.
¶219:
¶220: TF: How’s the physical room present when you look at the number space?
¶221:
¶222: AB: I guess all of the physical room is like erm, like what’s in my periphery normally. So the focus of my eyes is somewhere about here, because there’s nothing in the physical room here. The whole physical room is out of focus.
¶223:
¶224: TF: So for example here is the box just below it. So what happens with the box?
¶225:
¶226: AB: Well I look at the box and the box is shut <cross talking> if I look at
¶227:
¶228: TF: If you look at the box is the space still here? So if you focus your attention on it and do the trick again, try to become aware of the periphery or it just physical room in that case?
¶229:
¶230: AB: Erm. Yes, so then there’s like orangey stuff up here in the periphery, just like those red yellow things on the wall in the periphery when I was looking at this.
¶231:
¶232: TF: And when you look at the bottom of the green space. I mean sorry, the green mist, green wall, if you look at it's bottom border, where does it end, can you point out how high it is here?
¶233:
¶234: AB: Erm. How high is it, it's kind of up here somewhere.
¶235:
¶236: TF: The bottom bit <questioning tone>
¶237:
¶238: AB: The top.
¶239:
¶240: TF: Er sorry, where does it end at the bottom.

¶241: AB: Oh where does it end at the bottom. I can’t see very much when I’m focusing on the box. I am only aware of it up here, I’m not aware of anything down here.
¶242:
¶243: TF: No sorry, I meant if you look at the green wall and then try to. Can you look at the bottom of the wall and then indicate where you are looking at now, where you’re eyes focused at what height.
¶244:
¶245: AB: It's kind of like a black hole from here.
¶246:
¶247: TF: Oh so it's green above?
¶248:
¶249: <pause>
¶250: AB: It’s kind of black. It’s kind of dark all round.
¶251:
¶252: TF: What happens with the presence of the physical room?
¶253:
¶254: AB: When I think of the black bits <questioning tone>
¶255:
¶256: TF: Yeah.
¶257:
¶258: AB: Again it's in my periphery. When trying to focus on the blackness there’s nothing there, so I’m totally unfocused on anything.
¶259:
¶260: TF: But there’s a sense of the layout of the room. <AB agrees> OK right, so maybe we could leave that. I just wanted to see how the two spaces interfaced with each other. So I think there’s a lot of interesting stuff there already. But I want to return to something else that seems peculiar to this number, which was the first thing you saw was a graph, so that hasn’t happened to any of the numbers right?
¶261:
¶262: AB: No, I didn’t see anything geometric with pie.
¶263:
¶264: TF: Even though pie is a shared geometic number. That’s interesting!

¶265: AB: Strange.

¶266: TF: If you go back to E, do you see the two graphs that you mentioned?
¶267:
¶268: AB: Yeah, kind of one over here and one over there.
¶269:
¶270: TF: Are they also on white pages?
¶271:
¶272: AB: Erm yeah. I’ve got a little formula over here of E to the i pie equals minus 1 <laughter> Well they are kind of in the wrong place in the diagram of the complex plane, it's kind of plus 1 rather than minus 1.

¶273: TF: Can you draw it, just do a new one?
¶274:
¶275: AB: So kind of got that and got the circle, so here got pie minus 1.
¶276:
¶277: TF: What happens if you go up the y axis?
¶278:
¶279: AB: Up the y axis.
¶280:
¶281: TF: Yeah. How does it end?
¶282:
¶283: AB: Just like an error. There’s a letter y. kind of overlapping with that y there’s kind of like i m.
¶284:
¶285: TF: And the x axis?
¶286:
¶287: AB: Say there’s an arrow here. It's kind of got a bit of a black line stopping, there being a bit of y then the arrow.
¶288:
¶289: TF: Are you saying there’s a gap in the axis?
¶290:
¶291: AB: Kind of seems to be fading a bit.
¶292:
¶293: TF: But this didn’t happen for the y axis.
¶294:
¶295: AB: No.
¶296:
¶297: TF: And the x is below the axis <questioning tone>
¶298:
¶299: AB: x is to the right of the arrow here. There’s an x and an r e overlapping.
¶300:
¶301: TF: What you say the two axes are of equal length?
¶302:
¶303: AB: Yeah, although the y one seems longer right now.
¶304:
¶305: TF: Is there any visible relationship between this graph, the other graph and the numbers or are they just occupying different spaces?
¶306:
¶307: AB: Seems to be occupying different spaces.
¶308:
¶309: TF: Do they share the same background?
¶310:
¶311: AB: Erm. There’s kind of white, then coming from the left to the right there’s kind of redness, to the left hand side of the y axis now.
¶312:
¶313: TF: You mean of the unit circle?
¶314:
¶315: AB: Just red all the way from the top to the bottom. It's like it's a big, just red, everything sort of after the y axis seems kind of red.
¶316:
¶317: TF: Of which graph?
¶318:
¶319: AB: The graph of the complex plain.
¶320:
¶321: TF: So the left. Didn’t you say before on the left hand side was the exponential or am I mixing up the left hand?
¶322:
¶323: AB: Exponential graph is over here.
¶324:
¶325: TF: So what happens to the red, does it stop before the other graph starts?
¶326:
¶327: AB: Yeah.
¶328:
¶329: TF: So if you move your attention from one graph to the other, what’s in between?
¶330:
¶331: AB: Erm, nothing in between, it's just like a little thin black line, it's just kind of two sheets of paper kind of touching each other.
¶332:
¶333: TF: But the exponential graph is without colour.
¶334:
¶335: AB: Yeah.
¶336:
¶337: TF: Are the axis labelled?
¶338:
¶339: AB: Yeah. There’s x and y. Arrow heads and x and y. There’s a few little like tips on the axis as well, where the different numbers lie.
¶340:
¶341: TF: Can you see the numbers on the axis?
¶342:
¶343: AB: I can see 1 2 3 4 on the x axis.
¶344:
¶345: TF: Are the axis fixed and static or could you add one more number? What’s the last one that you see in the x for example?
¶346:
¶347: AB: Can add a 5
¶348:
¶349: TF: You can add a 5 <questioning tone>
¶350:
¶351: AB: 6 yeah.
¶352:
¶353: TF: What happens to the graph when you add numbers?
¶354:
¶355: AB: Numbers seem to bunch up, but the axis itself seems to stay the same.
¶356:
¶357: TF: And the curve?
¶358:
¶359: AB: Curve seems to stay the same.
¶360:
¶361: TF: Shouldn’t it change with the additional numbers?
¶362:
¶363: AB: Well, the scale of the y axis must be changing to compensate for that.
¶364:
¶365: TF: OK, let’s just check that out. What’s the highest on the y axis?
¶366:
¶367: AB: There are no numbers on the y axis.
¶368:
¶369: TF: Oh, there are no numbers just marks.
¶370:
¶371: AB: Yeah, just a few marks.
¶372:
¶373: TF: Well that’s quite clever of the y axis. <laughter> Is there anything else you want to explore about rational numbers? I think for me we can move onto maybe another topic? Maybe we should have a quick quick break. <AB agrees> Quite interesting that there’s associated graphs with this one.
¶374:
¶375: (34.46)
¶376: AB: What I like about <inaudible>
¶377:
¶378: TF: Is there any other irrational numbers that you?
¶379:
¶380: AB: Erm, not really. All the other ones are kind of like very obscure. Ones that have a symbol and then there’s endless ones you can do with square roots.
¶381:
¶382: TF: What about a physical constant?
¶383:
¶384: AB: Like the speed of light.
¶385:
¶386: TF: Yeah.
¶387:
¶388: AB: Yeah we could have one of those.
¶389:
¶390: TF: Shall we have a quick look to get a feel for it?
¶391:
¶392: AB: I’m already seeing a C equals 3 times 10 to the 8. So I’m seeing the approximation there that’s hugely used.
¶393:
¶394: TF: But no formulas or anything like that appearing with it?
¶395:
¶396: AB: Yeah, what else can interviewers Plank’s constant, like the H bar. I just saw like a H bar and then over here there’s an equals and a C square came up.
¶397:
¶398: TF: Did it happen before you saw the equation C equals 3 times 10 to the 8?
¶399:
¶400: AB: Yeah, it happened before the equation.
¶401:
¶402: TF: Are they still there, all the other associated things that appeared?
¶403:
¶404: AB: Yeah, they are still there.
¶405:
¶406: TF: But they appear in different locations than the graph for E <questioning tone>
¶407:
¶408: AB: So the graph for E was kind of here. Here is the C equals 3 times 10 to the minus 8. Plank’s constant is a little bit behind and then there’s the E equals mc squared is up here.
¶409:
¶410: TF: Ok so it's also to the top. <AB agrees>
¶411:
¶412: AB: It's almost like there’s a white board I can scribble on. <laughter>
¶413:
¶414: TF: So how come the Plank’s constant appeared?
¶415:
¶416: AB: Erm,
¶417:
¶418: TF: Did you think of the Plank’s constant as well?
¶419:
¶420: AB: Yeah, I guess because it's another fundamental constant of the universe. It's like C.
¶421:
¶422: TF: But you didn’t see any digits appear with it?
¶423:
¶424: AB: No, they are sort of coming along now.
¶425:
¶426: TF: Ok so if. Can you shift that one to the centre, so a closer look at Plank’s constant?
¶427:
¶428: AB: Yeah, it's kind of behind the C, it's difficult to move. <laughter> I get it over here. H equals 6 times 10 to the minus 34.
¶429:
¶430: TF: Do you see any equations or graphs?
¶431:
¶432: AB: Yeah, I got E equals H bar times F.
¶433:
¶434: TF: It's also sitting on top <questioning tone>
¶435:
¶436: AB: Yeah. And just underneath I’ve got H bar times a Greek niu as well.
¶437:
¶438: TF: So these are popular equations?
¶439:
¶440: AB: Well those are the same thing, the F and niu, the same equation and a different notation. I’ve got H bar equals H over 2 pie. Can’t even remember if these are correct or not. <laughter>
¶441:
¶442: TF: What about in string theory, are there any constants there you had to work with through a lot during your PhD?
¶443:
¶444: AB: Alpha has come into my mind.
¶445:
¶446: TF: What does alpha mean?
¶447:
¶448: AB: It's kind of like a string, string length or the tension of the string or something like that. Can’t remember. <laughter>
¶449:
¶450: TF: It’s just alpha and nothing else?
¶451:
¶452: AB: Yeah, well there’s alpha and there’s alpha prime came in with a dash.
¶453:
¶454: TF: But you don’t remember the. Oh, so it's different for each string?
¶455:
¶456: AB: There’s a universal constant.
¶457:
¶458: TF: There is a constant. So each string has the same tension?
¶459:
¶460: AB: Erm yeah.
¶461:
¶462: TF: So what are the digits? Can you see any digits associated?
¶463:
¶464: AB: No.
¶465:
¶466: TF: Ok so, that was a small side exploration. Let’s get back to just the normal number sequence. Just try to explore some of the bigger ones. What about 1956?
¶467:
¶468: AB: 1956 erm. So I see 1 9 5 6.
¶469:
¶470: TF: The 5 6 is not in anyway different than the 1 9?
¶471:
¶472: AB: Erm no. There’s kind of a line here under the 50’s.
¶473:
¶474: TF: I was just wanting to check whether the 56 might be different, because at least in the 50’s when we go from 1 to 100 it’s different, but in this case it looked the same.
¶475:
¶476: AB: Erm yeah, it's going to be about the same. I mean the, while I was talking about it the 1 9 is a bit more in the background and the 5 6 is a bit more here closer to me. The 5 6 looks similar to the 56 I saw before with kind of markings on it. The 9 has markings on it as well.
¶477:
¶478: TF: They appeared with the change.
¶479:
¶480: AB: I noticed markings on the 9 right away. So it's black and white and there’s kind of like white sort of markings on it.
¶481:
¶482: TF: Is it the stripes that you saw?
¶483:
¶484: AB: Erm no. It's almost kind of like pot mark, likes the ink has come away in lots of places.
¶485:
¶486: TF: So if you look at the beginning of these 100’s 1900, does it again, does the 9 look different?
¶487:
¶488: AB: It’s kind of looking more black now. It's not really stabilising that easily. It's kind of black for a bit then it's kind of hollow, just for the outline in black in. That kind of orangey, greeny background, you can see through the outline in ink.
¶489:
¶490: TF: Ok so it's just the contour you mean?
¶491:
¶492: AB: And then I got the line like this, going up to 1910 there.
¶493:
¶494: TF: So it's moved from 1900 to 1910, it's moving leftwards. So at the end of the sequence 1998, where would that be?
¶495:
¶496: AB: 1998 is up here.
¶497:
¶498: TF: And does it look?
¶499:
¶500: AB: 2000 is there.
¶501:
¶502: TF: How did it get from here to there!? <laughter> It must have taken a <inaudible word>
¶503:
¶504: AB: It’s really weird <laughter>
¶505:
¶506: TF: They look different <questioning tone>
¶507:
¶508: AB: Just filling in the white board again.
¶509:
¶510: TF: Wherever there’s space.
¶511:
¶512: AB: 1910 there’s 1920. I don’t know. Then I guess the 20’s are only coming up here.
¶513:
¶514: TF: But the 9 in 98, what do they look like?
¶515:
¶516: AB: They are kind of black, big thick black. 8 has a bit of things going on with it. Kind of a bit of the outline again. 1 is much smaller than the other numbers, like much thinner.
¶517:
¶518: TF: Seems to be quite a lot going on in this range <laughter> When were you born?
¶519:
¶520: AB: What year?
¶521:
¶522: TF: Yeah.
¶523:
¶524: AB: 1981
¶525:
¶526: TF: So what does that number look like?
¶527:
¶528: AB: Erm, so that’s over here now. Erm so it's kind of against the kind of greenish background. The 8 is kind of hollow again. It's sort of thinner the numbers. In thinner ink, thinner lines and black.
¶529:
¶530: TF: So you said it was here <questioning tone>
¶531:
¶532: AB: Yes here.
¶533:
¶534: TF: Ok so we’re going make this this, 20’s up and then we are I guess going vertically up to the 90’s.
¶535:
¶536: AB: So it's got 1981 1982. 80’s kind of going like this. Hard to say, almost like a kind of 1990 and 91 are coming out my left.
¶537:
¶538: TF: Quite sharp turns.
¶539:
¶540: AB: Yeah.
¶541:
¶542: TF: Much sharper than the beginning of the number line.
¶543:
¶544: AB: There’s some kind of 3Dness going on that I can’t quite figure out.
¶545:
¶546: TF: Well it looked like you said that the 20’s were actually further back here. So it wasn’t all like in a vertical plain. <AB agreeing throughout> We have to get you a 3D tool package to like trace it out.
¶547:
¶548: TF: I am curious, maybe another little side tour. We were talking about the different numbers and we mentioned the year of your birth 1981, but it appeared in the same number sequence, it didn’t appear as a year. You said you also have synaesthesia with year. <AB agrees throughout> So if you think about it as a year, does it appear differently?
¶549:
¶550: AB: Erm, I think I was thinking of it as a year.
¶551:
¶552: TF: So all of those, you were thinking of them as years?
¶553:
¶554: AB: Yeah, I think so.
¶555:
¶556: TF: So if you were thinking of them as numbers how would they be different?
¶557:
¶558: AB: Don’t know. Well I saw a comma between the 1 and 9 actually. <laughter>

¶559: TF: But that would indicate it's a number and not the year <questioning tone>
¶560:
¶561: AB: Yes, so a comma just kind of flickered between the 1 and the 9 there.
¶562:
¶563: TF: So if I tell you what about 1,995?
¶564:
¶565: AB: Up here. 1 comma 9 9 5.
¶566:
¶567: TF: Ok what if I tell you now that say Francisco published a nice paper in 1995?
¶568:
¶569: AB: 1 9 9 5
¶570:
¶571: TF: Do you have both of them in view, the one comma and?
¶572:
¶573: AB: Yes, there’s one here and one there. There’s comma 9 9 5 and it's kind of thinner letters, thicker digits. This one’s got thicker digits 1 9 9 5, no comma.
¶574:
¶575: TF: But otherwise they look the same, thinner and a comma. Those are the differing factors. <AB agrees>
¶576:
¶577: <pause>
¶578: TF: What happens when we go to the past, say 4000 BC?
¶579:
¶580: AB: 4000 BC. So first there’s kind of not struggling to find it on the number line. See this kind of curve here which was like, you got 0 and these are kind of AD and back into BC. 10 BC, 20 BC.
¶581:
¶582: TF: Can you draw it, just the beginning?
¶583:
¶584: AB: So something like that, so that’s 0, like 10 AD, 10 BC, 20 BC and then I was kind of here and it was kind of going off here. Then I was kind of losing touch with it and then 4000 BC just in numbers and the capital being capital C here.
¶585:
¶586: TF: So there doesn’t seem to be any regularity in it? At least not in the initial years.
¶587:
¶588: AB: No. Well this is similar to the like positive and negative number. So there I would see 10 0 -10 -20. This bit is like 1 to 10, 10 to 20 positive numbers and I’ve got a kind of curve there. Yeah, I couldn’t find it on this line and eventually disappeared in the digits <laughter>
¶589:
¶590: TF: Right. It seems that the representation of the numbers is the same, at least as far as we can tell from here. While we were looking at the two 1995, like the 1,995 and the 1 comma 995, they didn’t appear in the same location right?
¶591:
¶592: AB: No. Well the 1981 kind of got a comma added to it.
¶593:
¶594: TF: Ok, are there any other contexts in which numbers appear? We’ve looked at physics, arithmetic, years.
¶595:
¶596: AB: So there’s the dates of the calendar is the other.
¶597:
¶598: TF: You mean the days?
¶599:
¶600: AB: The days of the year.
¶601:
¶602: TF: The days of the year. So today is the 22nd what happens then?
¶603:
¶604: AB: So I see a 22 here. So I think each set of 10 days, each 10 day chunk within the year is kind of, has its own kind place and direction it's going in.
¶605:
¶606: TF: It’s in chunks of 10 <questioning tone>
¶607:
¶608: AB: So the whole year is like one big kind of like clock type thing.
¶609:
¶610: TF: Like an oval or more round?
¶611:
¶612: AB: If you zoom out far away it looks round, but as you zoom in, its jagged because there’s a corner at every 10.
¶613:
¶614: TF: Can you draw a month please?
¶615:
¶616: AB: So erm.
¶617:
¶618: TF: Let’s start with one, let’s say the 1st March.
¶619:
¶620: AB: So 1st March. <pause> So the 1st to the 20th <inaudible word> will be going in the same line. Then 20 will be going like this. So 1st March and 20th March until 31st March and beginning of April. 1st April, 10th April and then there’s 20th April, 30th April.
¶621:
¶622: TF: And what about February, where does it connect?
¶623:
¶624: AB: So February, <inaudible utterances> 20th, 2nd, so something like this.
¶625:
¶626: TF: When you draw it like that, you don’t see the line you see the number space differently <questioning tone>
¶627:
¶628: AB: It’s whatever day I’m focusing on I sort of see that number, but then I am aware of the line around it.
¶629:
¶630: TF: When you mean line, is that metaphorically saying you know what numbers could come next or is it actually you see a line?
¶631:
¶632: AB: So a similar thing is happening with the number line, so a number appears and there’s like a line down here. And then sometimes I see the numbers kind of like that, standing up on the line.
¶633:
¶634: TF: Ok so it's very similar to the normal number sequence. So how do you know which month it is?
¶635:
¶636: AB: Erm, because where they are on the clock face. So, February is right at the bottom and August is at the top.
¶637:
¶638: TF: So there’s a circle. <questioning tone>
¶639:
¶640: AB: So February. Yeah, so the months themselves are in a circle. So the year goes that way around. So February goes from half past 6 to half past 5 and there’s the 12 so August is right at the top, goes from half past 12 to half past 11, etc, etc and January is here.
¶641:
¶642: TF: And when you zoom in you see the 22nd, how do you know it's the 22nd of March and not August?
¶643:
¶644: AB: So the 22nd August is up here and the 20’s of August are going in that direction, whereas the 20’s in March are kind over here and going like that.
¶645:
¶646: TF: So does that mean when you focus on one date you still have a presence of the rest of the circle?
¶647:
¶648: AB: Yeah. So the 20’s of March I’m seeing them like this, kind of in that direction and they’re sort of slightly to my right. Whereas the 22nd of August is kind of up here and going that way and then September begins going in that direction.
¶649:
¶650: TF: So even when you’re looking close and say looking at the 22nd, you have the 21st and 23rd, only way you can tell you are dealing with March is due to the spatial location of those numbers in relation to the rest of the circle?
¶651:
¶652: AB: Yeah.
¶653:
¶654: TF: So they all appeared in the same location?
¶655:
¶656: AB: Same location with respect to the rest of the year.
¶657:
¶658: TF: But the rest of the year is not always visible is it?
¶659:
¶660: AB: I just have a vague sense of its presence. So if I look as 22nd of March being here then the circle of the year is kind of like this. And I know we are in March just because of that position there.
¶661:
¶662: TF: Right. Do you already see a bit of the other month as well or is it just focused on a couple of days?
¶663:
¶664: AB: Erm, it depends on what I draw my attention to. I can draw my attention to whatever I like really. September is here, October, November, December, January, February.
¶665:
¶666: TF: So what makes the month distinct from each other?
¶667:
¶668: AB: Erm the position on the clock and the direction that the days are going. So like the 20’s of March are going in that direction, 20’s of August are kind of going in that direction, so they have got angles like that in August. And March ones are like this.
¶669:
¶670: TF: In January there’s a trend basically, because they will have to fall into a circular pattern. They would somehow even though their angles are different, generally match up to a certain point. <AB agreeing throughout>
¶671:
¶672: AB: So I don’t know if it forms a consistent circle actually, the different directions, the angles there.
¶673:
¶674: TF: Yes some synasthetics have a little bit of a skewed circle, where some months take up more space than others. Or some of it is circular and others turn out to be more of a corner. What interests me is that you seem to be very sure of where one month begins and one ends, but that place is not specially marked in anyway. It's just from past experience you know that there is where the break happens in relation the rest of the patterns of the circle. <AB agreeing throughout> Is there any ambiguous crossing? So for example here, we are looking at March. You said from 1 to 20 is pretty much a straight line even though there is a 10 in the middle. But you don’t have that smooth connecting months.
¶675:
¶676: AB: Between any two months. December to January is kind of like that. January to February kind of January ends with it like that. February to March is kind of a bit like that. March to April, got this. April to May. June to July. July is starting off in that direction.
¶677:
¶678: TF: Ok it would be interesting perhaps to see whether each one of those corners you describe has a unique angle. I don’t know exactly how we record that. But maybe we could give you either a, erm what’s it called the thing where you draw an angle?
¶679:
¶680: AB: Protractor.
¶681:
¶682: TF: And then we’ll go through each of the corners and then perhaps try to match it and then see when we look at them they are all different, like significantly different from each other.
¶683:
¶684: AB: Yeah, it would be interesting. Yeah, so we can get a 360 degree protractor and line it up, all in the same place each time and then we can look at the angle for when the old month is coming in and the angle for the new month is coming out.
¶685:
¶686: TF: Right exactly. And from what you describe just now we would have to test it, but it sounded like each of them had a slightly. None of these are the same for one thing, but they might all be quite significantly different. I don’t know what the variance is between them. <AB agrees>
¶687:
¶688: AB: Yeah interesting.
¶689:
¶690: TF: Ok well maybe we’ll just leave it at that.
¶691:
¶692: AB: Yeah.
¶693:
¶694: TF: Great.

¶695: **End of interview.**
