# Supplementary material for: An extended case study on the phenomenology of sequence-space synesthesia
Source: Front Hum Neurosci. 2014 Jul 3;8:433. doi: 10.3389/fnhum.2014.00433 (PMC4080762; doi:10.3389/fnhum.2014.00433)
Supplement: Supplementary file 1 [file Presentation1.ZIP › Supp Mat 2.ZIP!/Referenced_Interview_Transcipts/AB - 20100320b.doc]

**Name:** AB - 20100320b

**Description:** Dialogue between TF and AB on 20b/03/2010

¶1: **Dialogue between TF and AB on 20b/03/2010**

¶2: (0.00)

¶3: TF: Ok so just to continue from where we left off a minute ago. You said that times of the day also elicited an experience and you said you see a clock face and the hands of the clock are pointing so that you can read off the time. <questioning tone> What does the clock like? Let’s take the time we have. So now it's 15.20.

¶4: AB: Yeah. So I just see, so it's kind of a black like outline of the clock and then it's got the minute markers and they are kind of going in towards the middle of the clock from the outside, with bigger ones for multiples of 5. Erm
¶5:
¶6: TF: And the hours
¶7:
¶8: AB: Yes. So, the hours are the bigger ones. There’s no numbers on the clock.
¶9:
¶10: TF: No numbers <questioning tone>
¶11:
¶12: AB: No. Well I kind of get vague ones coming in along, round the outside of the clock actually. Yeah, If I can focus my attention on it. Yeah and then the basically the hands of the clock showing 3.20
¶13:
¶14: TF: And what does the hand look like?
¶15:
¶16: AB: Just kind of big black lines.

¶17: TF: What do they have at their end?
¶18:
¶19: AB: Just like erm a rectangle
¶20:
¶21: TF: Oh so they are not pointed or anything?
¶22:
¶23: TF: So the clock is white?
¶24:
¶25: AB: Yeah.

¶26: TF: What about the seconds?
¶27:
¶28: AB: Second hand kind of flickers in and out of existence. I wasn’t aware of that at all.
¶29:
¶30: TF: Does the pointer look the same as the other two?
¶31:
¶32: AB: Looks thinner. It’s not really going around.
¶33:
¶34: TF: Ok, if I told you about seconds <laughs>. So if it had flickered it's not like it's moving around to the next minute from when I said what time it was.
¶35:
¶36: AB: Mm no. It's kind of going up towards somewhere between 5 and 10 seconds. 9 10 11 12. <cross talking> That line goes around as I focus on it <laughter>
¶37:
¶38: TF: So what happens when you let it go around once?
¶39:
¶40: AB: <pauses> There’s a 45 there. So I don’t see it like every second it's just whenever my attention comes back it's kind of moved on. There’s a 55 now, 60. So yeah, it's just kind of moving around.
¶41:
¶42: TF: But what happened to the minute.
¶43:
¶44: AB: <pauses> So the minute is saying 3.21 now.
¶45:
¶46: TF: You didn’t see it change when it hit the 60?
¶47:
¶48: AB: No, I wasn’t paying attention. I was only paying attention, so I was aware of those other hands only vaguely aware but I didn’t know exactly where it was.
¶49:
¶50: TF: And you didn’t see any movement of the finger pointing to a new number?
¶51:
¶52: AB: No
¶53:
¶54: TF: Even when the seconds was moving around it was more like, it wasn’t the shifting from one to the other it was more like and disappearing in one and appearing in the next when you
¶55:
¶56: AB: I wasn’t paying any attention to the minute hand, but then when I drew my attention back to it, it was pointing at 3.21.
¶57:
¶58: TF: But in terms of the second, you described the movement not as being one of, you know it's not continually going around.
¶59:
¶60: AB: No, it's kind of like appearing, its kind of flashing
¶61:
¶62: TF: But it's not doing it automatically, you have to look at it <questioning tone>
¶63:
¶64: AB: Yeah, pay attention to it.
¶65:
¶66: TF: Is it possible for you to see the time in a digital watch?
¶67:
¶68: AB: So I just saw 3.22 kind of like orange, and it's kind of like an alarm clock. So it's kind of double dots between the 3 and 2 and they are kind of flashing
¶69:
¶70: TF: They’re flashing <laughs>
¶71:
¶72: AB: Yeah it's showing the seconds kind of going.
¶73:
¶74: TF: Oh so it has seconds as well <questioning tone>
¶75:
¶76: AB: Yeah
¶77:
¶78: TF: So it's a, can you see the individual bars the numbers are made of?
¶79:
¶80: AB: If I zoom in close enough, if I pay attention.
¶81:
¶82: TF: So do you not have two clocks?
¶83:
¶84: AB: I have all kinds of clocks
¶85: <laughter>
¶86:
¶87: TF: What do you mean? <laughs>
¶88:
¶89: AB: There’s another one with more smooth kind of rounded 3’s and 2’s over here on a white background, well just kind of on the page. 3 dot dot 22 and then there’s a kind of like 10 seconds.
¶90:
¶91: TF: <inaudible utterances> (5.53)
¶92:
¶93: TF: I was just thinking of other variations. Oh what about this, here’s a challenge for you, can you do big ben?
¶94:
¶95: AB: Yeah, big ben is over here <laughter> it’s showing 6 o’clock.
¶96:
¶97: TF: So do you see the whole clock face?
¶98:
¶99: AB: So I see the whole tower, houses of parliament, Westminster bridge. Yeah, I’m kind of standing diagonally across the street from it. Got the whole kind of houses of parliament and traffic going around.
¶100:
¶101: TF: Wow! So was there actually a lively scene?
¶102:
¶103: AB: So a whole scene of London.
¶104:
¶105: TF: But it was not a static scene <questioning tone>
¶106:
¶107: AB: It was kind of just a lot there, but kind of taking in. If I was kind transported physically and I was kind of taking one thing after another and being aware of all the different things.
¶108:
¶109: TF: I am just interested in whether it was more like, it wasn’t more like a picture quality it's more as if you were there.
¶110:
¶111: AB: Yeah, it was like I was there and focusing my attention on different things.
¶112:
¶113: TF: Right. <pauses> And can you still see it?
¶114:
¶115: AB: Yeah.
¶116:
¶117: TF: Ok. Just took <unclear> (7.28) If you let your gaze go down the tower and look at the street in front. What do you see in the street?
¶118:
¶119: AB: Erm, road, lanes and some cars going by quite quickly, traffic lights. Feels like I’m kind of in the middle of the road and hovering above the road.
¶120:
¶121: TF: Ok, let’s have a look whether you can influence the scene or whether it would resist it. Can you change the traffic light to a different colour? Let’s say can you stop the traffic?
¶122:
¶123: AB: Erm, let’s the traffic light is kind of sideways on, so you can’t actually see what colour it is.
¶124:
¶125: TF: Ah ok.
¶126:
¶127: AB: Well there’s a kind of a red man, like the kind of pedestrian crossing thing. It’s like a red man. <laughter>
¶128:
¶129: TF: Can you make it go green so that pedestrians can go across?
¶130:
¶131: AB: I can get the red and the green to be simultaneously, but I can’t get rid of the red.
¶132:
¶133: TF: Outside the
¶134:
¶135: AB: Oh now it’s gone green <laughter> So the red is flickering a bit.
¶136:
¶137: TF: Have the cars stopped?
¶138:
¶139: AB: Erm, there’s a kind of like almost like ghosts of cars kind of going around the corner. There is a car stop there.
¶140:
¶141: TF: Can you switch it back so it can go on its home, go on its way?
¶142:
¶143: AB: Not that easily. <quiet?
¶144:
¶145: TF: Ok well. What would do the trick? Well just imagine anybody wanted to go across now, had their chance and moved on so.
¶146:
¶147: AB: So there’s a lot of stuff kind of going on there. There’s like a red car just stopped at the lights <laughter>
¶148:
¶149: TF: But is everybody else taking a different road now?
¶150:
¶151: AB: Erm dunno. There’s like ghost cars that are just kind going through around the corner. Kind of like an old car.
¶152:
¶153: TF: Do you see any people?
¶154:
¶155: AB: Yes. People kind of crossing the street.
¶156:
¶157: TF: Oh that’s why you can’t turn over because there’s people crossing the street.
¶158:
¶159: AB: Yeah <laughter> There’s someone waiting to cross, oh with a buggy. <laughter>
¶160:
¶161: TF: So, the scene look contemporary?
¶162:
¶163: AB: Yeah it looks contemporary. There’s a car kind of waiting at the lights, it’s a kind of old fashioned kind of car.
¶164:
¶165: TF: I mean do people look dressed as you and me would be dressed?
¶166:
¶167: AB: Erm yeah.
¶168:
¶169: TF: Well there would be loads to explore here but maybe we’ll leave it and ok. Well, oh there was one more age. So if I say I’m 28 what happens?
¶170:
¶171: AB: So I see 2 8.
¶172:
¶173: TF: Right, is it any different to me saying imagine 28?
¶174:
¶175: AB: No, it doesn’t seem that different actually. Kind of the 27 beneath and the 29 above.
¶176:
¶177: TF: So it could just be a variation of the number on the number line <questioning tone>
¶178:
¶179: AB: Yeah. While I got a sense it might different. There’s more kind of the higher numbers that are less familiar from kind of pure maths and kind only really used in the context, more often the context of people’s ages.
¶180:
¶181: TF: Ok. So imagine that I met a friend this weekend who told me that his baby was 6 months old. What happens with the month?
¶182:
¶183: AB: So it's kind of a 6 here. Yeah going back towards birth. Birth is kind of over there. First year seems to look something like that. A little bit different.
¶184:
¶185: TF: So ok. It's interesting, so the months just slotted in to fill, become like a number sequence in themselves. It's not like a fraction of the years.
¶186:
¶187: AB: No.
¶188:
¶189: TF: So how was it. What if it's 1 year and 2 months, what happens then?
¶190:
¶191: AB: Erm. I don’t know.
¶192:
¶193: TF: So you have a 1 year.
¶194:
¶195: AB: 1 year here, it's kind of 12, 1 year 2 months, 18 months is kind of over here.
¶196:
¶197: TF: So did you see it as 2 months or did you see it as 14 months?
¶198:
¶199: (13.25)
¶200: AB: Saw it as kind of, <inaudible> interview. I was more aware of position than of particular number. Well I can do a 14 there now. 1 4
¶201:
¶202: TF: When I said it before you just knew where the number would be, but there was no specific image for the number.
¶203:
¶204: AB: No.
¶205:
¶206: TF: Now here’s a strange question. What happens at the zero?
¶207:
¶208: AB: Kind of like an image of a woman giving birth <laughter> And there’s a number zero <laughter>
¶209:
¶210: TF: You mean there’s an image of giving birth. Do you see a woman?
¶211:
¶212: AB: Little baby.
¶213:
¶214: TF: Is it moving? Do you see doctors?
¶215:
¶216: AB: Yeah, there’s kind of like a whole room. There’s kind of nurses.
¶217:
¶218: TF: I guess there’s nothing before that I mean. I mean for example that you have negative numbers. Does that make sense in this context?
¶219:
¶220: AB: No, it would have kind of made sense as something in the womb but don’t really know where it faces.
¶221:
¶222: TF: Yeah, I guess you could say that you know, what happens at 6 month pregnancy
¶223:
¶224: AB: So it's kind of over here. Yeah, kind of an image of a sort of fairly black womb with a sort of like foetus in it.
¶225:
¶226: TF: Can you trace it's different stages?
¶227:
¶228: AB: Erm, yeah the conception is kind of up here. Like a fertilised egg and of a clump of cells.
¶229:
¶230: TF: Right. So is there like cell division going on?
¶231:
¶232: AB: Yeah. And then there is kind of like a curve at the back of his head. <laughter>
¶233:
¶234: TF: So, how many steps are there for each? Is it monthly the images or?
¶235:
¶236: AB: Don’t know. Well, the cell divisions are kind of much closer together. And here it's kind of expanding.
¶237:
¶238: TF: Ok but what are the divisions? You make emotion as going this way. Do you see any particular points on that line?
¶239:
¶240: AB: So the focus here, there’s kind of like a new embryo type thing. There’s kind of like a black kind of sweeping thing that again, the kind of the width of the line getting bigger as you get to here.
¶241:
¶242: TF: Kind of like a tunnel <questioning tone>
¶243:
¶244: AB: Yeah. Seems like a tunnel yeah. It's kind of winding up.
¶245:
¶246: TF: So the tunnel is not
¶247:
¶248: AB: It’s kind there’s a kind of like a foetus here and foetus there. So more like an embryo here.
¶249:
¶250: TF: So you do you need to put your attention or to see the stage that point is at in the tunnel?
¶251:
¶252: AB: Yeah.
¶253:
¶254: TF: So it's kind of like the numbers in that sense, I guess.
¶255:
¶256: AB: Yeah.
¶257:
¶258: TF: So it starts up there somewhere?
¶259:
¶260: AB: So at the very top somewhere there is one of those magnified pictures of an egg with loads of sperm <laughter>
¶261:
¶262: TF: So just like you would see in a biological textbook. Is it like, ok so in contrast to the
¶263: London scene, what’s the quality of these images like? is it like I said a textbook?
¶264:
¶265: AB: Yeah, more static. It's not like movement.
¶266:
¶267: TF: So these are static ones<questioning tone>
¶268:
¶269: AB: Yeah.
¶270:
¶271: TF: Even those scenes with the doctor here?
¶272:
¶273: AB: Oh yeah, it seemed to be like distinct scenes. So one point the baby was kind of removed and there was kind of like the umbilical cord <laughter>
¶274:
¶275: TF: ok, it was snapshots. It wasn’t like a scene unfolding.
¶276:
¶277: AB: Hear a bit of like baby crying there.
¶278:
¶279: TF: Wow! You are moving onto a new level. We never had any sound experience from other interviews. What about the tower, could you hear the big Ben?
¶280:
¶281: AB: No, didn’t notice any sounds.
¶282:
¶283: TF: Because it was at 6 right <questioning tone>
¶284:
¶285: AB: Yeah. I didn’t hear it.
¶286:
¶287: TF: So in terms of the baby sounds, does it go with the movement in the scene or is it kind of dissociated?
¶288:
¶289: AB: Seems kind of dissociated, but it was kind of coming from over there.
¶290:
¶291: TF: But it was more like a sequence of snapshots.
¶292:
¶293: AB: Yeah.
¶294:
¶295: TF: Can you hear the doctors?
¶296:
¶297: AB: No.
¶298:
¶299: TF: What about the medical machinery that’s in the room <questioning tone>
¶300:
¶301: AB: Erm
¶302:
¶303: TF: Can you see it in the pictures?
¶304:
¶305: AB: Well I focus my attention as kind of like erm, kind of like some scissor and a white tray on a long surface.
¶306:
¶307: TF: Oh yeah, they all need those!
¶308:
¶309: AB: Yeah, there’s like lots of drawers. There’s kind of like drawers and work surface kind of around here. And then there’s like the bed here, like the woman kind of lying down there. And there’s kind of like a window there at the back.
¶310:
¶311: TF: Can you see outside the window? Is it clear?
¶312:
¶313: AB: Just kind of like a corridor.
¶314:
¶315: TF: Oh, it's into the hospital and not to the outside.
¶316:
¶317: AB: Yeah, into the hospital.
¶318:
¶319: TF: Where’s the doctor standing in relation to all of this?
¶320:
¶321: AB: Err, so there’s like one here and then there’s like another here.
¶322:
¶323: TF: Are they interacting with each other or?
¶324:
¶325: AB: Can’t tell. It's just kind of snapshots.
¶326:
¶327: TF: Oh right.
¶328:
¶329: AB: Yeah, there’s also some kind of machinery above the bed like. I don’t really know what it's for.
¶330:
¶331: TF: You mean like a drip or <questioning tone>
¶332:
¶333: AB: It's that kind of metallic, kind of structure type thing above the bed.
¶334:
¶335: TF: Over effect for the curtain maybe <questioning tone>
¶336:
¶337: AB: Yeah maybe.
¶338:
¶339: TF: Or is it something electronic?
¶340:
¶341: AB: Yeah, so that’s something electronic.
¶342:
¶343: TF: It is or it is not?
¶344:
¶345: AB: Seems like its electronic <cross talking>
¶346:
¶347: TF: I guess there will be some lights up there, but that’s not what you mean.
¶348:
¶349: AB: No, the room’s just kind of well lit.
¶350:
¶351: TF: They know enough about nursery to ask any more precise questions I guess <laughter>
¶352:
¶353: TF: Ok, so maybe one thing that we can look at. So we had this whole scene with the bed and the drawers and work surface. Where was the zero in all of this?
¶354:
¶355: AB: <laughs> kind of superimposed by that scene at the hospital.
¶356:
¶357: TF: And then the 1
¶358:
¶359: (21.34)
¶360: AB: So there’s a 1 over here for like <cross talking> birthday
¶361:
¶362: TF: Ok it's far away. So it's quite separated somehow.
¶363:
¶364: AB: Well it's quite separated as there’s all those months in the first year of life.
¶365:
¶366: TF: Oh right of course, they have to fit in there.
¶367:
¶368: AB: So there’s a 6 over here, which was the first thing we looked at.
¶369:
¶370: TF: Ah ok, so it stayed there this time. See it's much more useful for you. I have to try to remember all these locations that you point to <laughter> But there’s no imagery associated with 6 months or the first year <question tone> The imagery stops at zero.

¶371:

¶372: AB: Erm, when I said that there was kind of an image of a baby, I heard a baby crying just then.
¶373:
¶374: TF: Does it still work for 1 year as well.
¶375:
¶376: AB: 1 year, just see the number 1 here. I didn’t hear any cry when you said.
¶377:
¶378: TF: And there’s no baby anymore?
¶379:
¶380: AB: Don’t see a baby. Well there’s kind of like a little baby here that looks like a newly born baby.
¶381:
¶382: <laughter>
¶383:
¶384: TF: That’s cheating! <laughter>
¶385:
¶386: AB: Ok. Kind of wrapped up in white blankets.
¶387:
¶388: TF: I guess that would be. The first month would be more like that, not the first year.
¶389:
¶390: AB: Yeah.
¶391:
¶392: TF: Yeah I guess there would be crawling around on the floor or something like that. But there’s nothing like that going on.
¶393:
¶394: AB: I’m getting a few more general images. Like, being at my friend’s house. My friend in Spain, he’s got a son whose just approaching 2. I saw him about a year ago and it's a kind of 1 year old crawling. But that’s kind of a whole different scene, sometimes I’m in the middle of a house. All around me like life size.
¶395:
¶396: TF: So that’s quite a vivid. Is that a memory or a reconstruction?
¶397:
¶398: AB: I think it's a reconstruction.
¶399:
¶400: <pause>
¶401:
¶402: TF: Ok cool <laughter>
¶403:
¶404: **End of interview.**
