# Supplementary material for: An extended case study on the phenomenology of sequence-space synesthesia
Source: Front Hum Neurosci. 2014 Jul 3;8:433. doi: 10.3389/fnhum.2014.00433 (PMC4080762; doi:10.3389/fnhum.2014.00433)
Supplement: Supplementary file 1 [file Presentation1.ZIP › Supp Mat 2.ZIP!/Referenced_Interview_Transcipts/AB - 20100408.doc]

¶1: **Name:** AB - 20100408

¶2:

**¶3: Description:** Dialogue between TF and AB on 08/04/2010

¶4:

¶5: (0.00)

¶6:

¶7: TF: Ok to start I have a little surprise for you. Right, what does that do to you looking at that?

¶8:

¶9: AB: I see the letter H on the page. <laughter>

¶10:

¶11: TF: So what do you need to do in order to see the alphabet line?

¶12:

¶13: AB: Erm, have to look away from the image. I can them here.

¶14:

¶15: TF: So where did the H appear?

¶16:

¶17: AB: There was an H here.

¶18:

¶19: TF: Ok so the whole thing all at once?

¶20:

¶21: AB: Erm I don’t know. <laughter>

¶22:

¶23: TF: So just looking at the letter t

¶24:

¶25: ¶1: **Dialogue between TF and AB on 08/04/2010**
¶26: ¶2:
¶27: ¶3: (0.00)
¶28: ¶4:
¶29: ¶5: TF: Ok to start I have a little surprise for you. Right, what does that do to you looking at that?
¶30: ¶6:
¶31: ¶7: AB: I see the letter H on the page. <laughter>
¶32: ¶8:
¶33: ¶9: TF: So what do you need to do in order to see the alphabet line?
¶34: ¶10:
¶35: ¶11: AB: Erm, have to look away from the image. I can them here.
¶36: ¶12:
¶37: ¶13: TF: So where did the H appear?
¶38: ¶14:
¶39: ¶15: AB: There was an H here.
¶40: ¶16:
¶41: ¶17: TF: Ok so the whole thing all at once?
¶42: ¶18:
¶43: ¶19: AB: Erm I don’t know. <laughter>
¶44: ¶20:
¶45: ¶21: TF: So just looking at the letter that doesn’t do the job.
¶46: ¶22:
¶47: ¶23: AB: No.
¶48: ¶24:
¶49: ¶25: TF: So when you said that you had to look away, what did you do?
¶50: ¶26:
¶51: ¶27: AB: So I think my eyes moved didn’t they and then an alphabet appeared here. Not sure quite what
¶52: ¶28: order. I was kind of immediately aware of the H, just kind of where I put my gaze. Away from the distracting piece of paper.
¶53: ¶29:
¶54: ¶30: TF: So all you were aware of was looking away, or did you notice any mental effort?
¶55: ¶31:
¶56: ¶32: AB: Yeah, when I was focused on the paper there was nothing else except what was physically there.
¶57: ¶33:
¶58: ¶34: TF: So how did you know?
¶59: ¶35:
¶60: ¶36: AB: When I looked away there was immediately, an alphabet appeared at least as far as H. I was only aware from A to H, I wasn’t aware of anything beyond H.
¶61: ¶37:
¶62: ¶38: TF: A to H <questioning tone>
¶63: ¶39:
¶64: ¶40: AB: Yeah, straight line.
¶65: ¶41:
¶66: ¶42: TF: So how did you know that you had look away from the page for the alphabet to appear?
¶67: ¶43:
¶68: ¶44: AB: Just because it wasn’t appearing when I was looking at it. <laughter> I opened the page for a few seconds and there’s nothing happening, so I though wait a minute shouldn’t an alphabet appear now and I kind of averted my eyes and then there it was. <laughter>
¶69: ¶45:
¶70: ¶46: TF: So there’s something about the page that wasn’t allowing it to happen <questioning tone>
¶71: ¶47:
¶72: ¶48: <pause>
¶73: ¶49: AB: Yeah, it's almost like the actual physical H can’t be part of the mental alphabet.
¶74: ¶50:
¶75: ¶51: TF: Oh so it couldn’t be that there would be letters coming here and then there would be slots. <questioning tone>
¶76: ¶52:
¶77: ¶53: AB: Don’t think so. I mean I can sort of imagine A B C D E F G kind of coming in like this.
¶78: ¶54:
¶79: ¶55: TF: But does it look?
¶80: ¶56:
¶81: ¶57: AB: Takes more effort.
¶82: ¶58:
¶83: ¶59: TF: Takes more effort, but does it look the same as the one that appeared in space?
¶84: ¶60:
¶85: ¶61: AB: Don’t know. Now, I’ve got an A B C D E F G coming in diagonally from kind of the top north west.
¶86: ¶62:
¶87: ¶63: TF: So when you say it takes more effort, what do you mean?
¶88: ¶64:
¶89: ¶65: AB: It's just because I’m focusing on the page, I can’t see anything there and so that’s why. Whereas if my eyes are kind of focus on some point in space in which there is not physical object then it's easier for the.
¶90: ¶66:
¶91: ¶67: TF: So do you focus your eyes?
¶92: ¶68:
¶93: ¶69: AB: I think my eyes are kind of defocused, just looking in space. So whatever is behind, first physical thing in the direction I am focusing my eyes that aren’t focused.
¶94: ¶70:
¶95: ¶71: TF: Right. So is the alphabet still in front of you?
¶96: ¶72:
¶97: ¶73: AB: Yeah, it's kind of here.
¶98: ¶74:
¶99: ¶75: TF: Ok so last time you said that in order to keep it there you had to internally always repeat the letter. Are you doing something like that this time?
¶100: ¶76:
¶101: ¶77: AB: Erm, after internally kind of say the letter that I am looking at that I mean I can have, I am going to be aware of the sequence with several letters in between A and H.
¶102: ¶78:
¶103: ¶79: TF: So when you looked at the H on the page and then you looked up, did you say H to yourself when you were looking up?
¶104: ¶80:
¶105: ¶81: AB: Mmm, don’t think so. Don’t know really.
¶106: ¶82:
¶107: ¶83: TF: When you look at the number line now, oh sorry the alphabet line, are you aware of having to say H in order to keep it in focus?
¶108: ¶84:
¶109: ¶85: AB: No, I think once it's there I don’t have to say H.
¶110: ¶86:
¶111: ¶87: TF: What about if you transfer it to the other end of the alphabet Z?
¶112: ¶88:
¶113: ¶89: AB: Just have to relook where it is.
¶114: ¶90:
¶115: ¶91: TF: It's just a movement of the eyes <questioning tone>
¶116: ¶92:
¶117: ¶93: AB: Yeah.
¶118: ¶94:
¶119: ¶95: TF: You don’t have to say anything.
¶120: ¶96:
¶121: ¶97: AB: No, I think you saying it is enough.
¶122: ¶98:
¶123: ¶99: TF: Ok. So what if you pick a random one, just choose it yourself, how would you go to it?
¶124: ¶100:
¶125: ¶101: AB: Yes, then I would have to say it in my head. So I think Q was kind of here.
¶126: ¶102:
¶127: ¶103: TF: So what did you do in order to see Q?
¶128: ¶104:
¶129: ¶105: AB: I kind of just thought of the letter Q by saying it in my head, then I kind of looked for it.
¶130: ¶106:
¶131: ¶107: TF: So it seems like perhaps what was difficult about using the page, was I didn’t say the letter when I showed it to you. You can try it with, let’s have a look at this. <AB agrees> Close your eyes. Ok you can look. What happens when you see this?
¶132: ¶108:
¶133: ¶109: AB: So at first I saw a B. I think it's the effect of the ink. <laughter> Then I saw 3.
¶134: ¶110:
¶135: ¶111: TF: Ok when you first thought it was a B.
¶136: ¶112:
¶137: ¶113: AB: Then I got a sense of a number line here.
¶138: ¶114:
¶139: ¶115: TF: Right. Because I try to figure out the order of how things happen. When you first saw it, you thought it was a B because of the line coming through the page. Was that enough to trigger your awareness of the B in your alphabet form?
¶140: ¶116:
¶141: ¶117: AB: Erm, I kind of become aware of it over here.
¶142: ¶118:
¶143: ¶119: TF: But at the time you were just focused on the page.
¶144: ¶120:
¶145: ¶121: AB: Yeah, it came later so it's not immediate.
¶146: ¶122:
¶147: ¶123: TF: Right.
¶148: ¶124:
¶149: ¶125: AB: But then neither was it immediate when you just say numbers and letters to me, assumedly it takes a few seconds to appear even then doesn’t it.
¶150: ¶126:
¶151: ¶127: TF: Yeah but then.
¶152: ¶128:
¶153: ¶129: AB: But then they are attached to the original one that I see. Whereas here it's always appeared, seems to be appearing to the right of the page.
¶154: ¶130:
¶155: ¶131: TF: I was just curious to see whether there is a difference between the letters and the numbers in terms of having to say them or whether seeing them is enough. So don’t know, it's difficult because you actually saw it as a letter first. <laughter> Ok, try another one. Look away. <pause> So what happened there?
¶156: ¶132:
¶157: ¶133: AB: So I see the 4 and then there’s nothing really stable coming into view. It's like the number line doesn’t know which direction to go in. It's like there are various things down on the table and something over here.
¶158: ¶134:
¶159: ¶135: TF: Ok so nothing.
¶160: ¶136:
¶161: ¶137: AB: Nothing very precise came in. Just very different from when you are telling me to think of the number 4.
¶162: ¶138:
¶163: ¶139: TF: But it seems almost like there’s an additional step that happens between coming into contact with a number letter and the form actually appearing. <AB agrees> So, perhaps when I say to you, say 5, your favourite, what happens then?
¶164: ¶140:
¶165: ¶141: AB: Number 5 here, 9 here, 4 6.
¶166: ¶142:
¶167: ¶143: TF: What was different there then I could have shown it to you on the page?
¶168: ¶144:
¶169: ¶145: AB: Erm, mental 5 on the page, like here 7. <laughter>
¶170: ¶146:
¶171: ¶147: TF: Is it something to do with when I say it, it has more the character of a command is in like I’m telling you to imagine 5? Whereas you seeing it on the page doesn’t tell you what to do with it.
¶172: ¶148:
¶173: ¶149: AB: Mmm, I don’t know.
¶174: ¶150:
¶175: ¶151: TF: So before you said it takes more effort to bring the alphabet and to touch with the H on the page. Can you say a little bit more of what you mean by effort?
¶176: ¶152:
¶177: ¶153: AB: Well it just appears first away from the page. I have to will an alphabet on the page in order to get it to happen.
¶178: ¶154:
¶179: ¶155: TF: Right. So what do you do in order to will it to appear on the page?
¶180: ¶156:
¶181: ¶157: AB: Erm, I just look around the H for sign of other letters.
¶182: ¶158:
¶183: ¶159: TF: So you look at say on the left of it and then you look for <inaudible word> with another letter B. <AB agrees> How do you know when you have found one?
¶184: ¶160:
¶185: ¶161: AB: Because it's just there. <laughter>
¶186: ¶162:
¶187: ¶163: TF: Are there any signs that you are looking for it?
¶188: ¶164:
¶189: ¶165: AB: Don’t know. I have to kind of go back in the beginning I guess.
¶190: ¶166:
¶191: ¶167: TF: Are there.
¶192: ¶168:
¶193: ¶169: AB: I mean I know it's sort of north west of it now.
¶194: ¶170:
¶195: ¶171: TF: Ok, so we can try a different one. So what happens if you want to have a number line here?
¶196: ¶172:
¶197: ¶173: AB: Yeah, I mean I can put a 1 and a 2 here.
¶198: ¶174:
¶199: ¶175: TF: So what do you do in order to make that happen? Did you have to look for where they could be?
¶200: ¶176:
¶201: ¶177: AB: It's kind of newer. We’re going to the.
¶202: ¶178:
¶203: ¶179: TF: And how did you know that?
¶204: ¶180:
¶205: ¶181: AB: Because I know where 1 and 2 are in relation to 3.
¶206: ¶182:
¶207: ¶183: TF: But I mean it could have come here, could have come here or you know. Why did they appear from this side?
¶208: ¶184:
¶209: ¶185: AB: I guess I kind of knew to look for them there.
¶210: ¶186:
¶211: ¶187: TF: You weren’t aware of any signs that would you tell that this is the area to look in?
¶212: ¶188:
¶213: ¶189: AB: Erm, normally it's like I said to myself so it's a case of it's a 3, so down to the left of 3 there should be a 1 and 2 and then <inaudible word>
¶214: ¶190:
¶215: ¶191: TF: Is the 5 still here?
¶216: ¶192:
¶217: ¶193: AB: Erm, I’m being aware of it now you said is there a 5 there?
¶218: ¶194:
¶219: ¶195: TF: Is it one of those embedded in the physical space of the room or is it one that’s seems to be in this other space?
¶220: ¶196:
¶221: ¶197: (14.03)
¶222: ¶198: AB: Seems to be floating up here on a white page. <Inaudible words> The white page has a tendency to melt away. It's got kind of patterning on it. It's got kind of cross hatching.
¶223: ¶199:
¶224: ¶200: TF: I’m looking for one that is part of this room. Would any one of the things that appeared on the table?
¶225: ¶201:
¶226: ¶202: AB: Yeah, there’s a 4 and a 6 kind of here.
¶227: ¶203:
¶228: ¶204: TF: There’s a 4 and 6 and they are sitting on top of a table <questioning tone>
¶229: ¶205:
¶230: ¶206: AB: Yeah.
¶231: ¶207:
¶232: ¶208: TF: So I’m just curious to see how adapted they are to your movements. If you moved your head from side to side like this, how does your perception of the number change?
¶233: ¶209:
¶234: ¶210: AB: Only it's still kind of like this, so I kind of can’t see it, can see it only side on. Erm, sometimes it tilts a little bit so it's kind of bi-stable.
¶235: ¶211:
¶236: ¶212: TF: But if you went like, if I wanted to see let’s say the other side of the bottle I could go like this right, look around. Can you do that to the number?
¶237: ¶213:
¶238: ¶214: AB: I can see the back of the 4. Yeah I can kind of see the 4 backwards. Sometimes it sort of spins around.
¶239: ¶215:
¶240: ¶216: TF: Oh when you look at it backwards.
¶241: ¶217:
¶242: ¶218: AB: So it's always kind of bi-stable, but yeah there’s kind of one where it's kind of back to front. Because I see it orientated with the front, this side.
¶243: ¶219:
¶244: ¶220: TF: If I looked at the bottle for example, there would be minute changes to when I’m moving. So there would always be a little bit less of this side I can see when I’m moving in this direction. Do you get the same effect?
¶245: ¶221:
¶246: ¶222: <pause>
¶247: ¶223: AB: It's kind of a bit distorted the effect, but it's not as much as it should be.
¶248: ¶224:
¶249: ¶225: TF: So it's not as smooth basically. <questioning tone>
¶250: ¶226:
¶251: ¶227: AB: Yeah.
¶252: ¶228:
¶253: ¶229: TF: If I move just a little bit, the perspective changes continuously while I move. Does it change continuously for you as well? Yeah well if you look down on it from the top, how does it look from there?
¶254: ¶230:
¶255: ¶231: AB: Yeah, I can see a bit more of the 4 than I should, like if I’m right above I should just all black. I can see more of it than I would expect. <laughter>
¶256: ¶232:
¶257: ¶233: TF: So you can still see that’s it's a 4, even though from the top it should be just a black line really. <AB agrees>
¶258: ¶234:
¶259: ¶235: AB: But it does kind of open up wider when I come in front.
¶260: ¶236:
¶261: ¶237: TF: Ok so, let’s look at that particular sequence. When you look at it from the top and then you sat down again and you said it opened up a little bit wider. How did it open up a little bit wider?
¶262: ¶238:
¶263: ¶239: AB: It's like the hole is bigger.
¶264: ¶240:
¶265: ¶241: TF: But I mean for example, how many steps did it take to open up?
¶266: ¶242:
¶267: ¶243: AB: So the 4 that I see is like that basically, a bit wider. It’s got some width to it.
¶268: ¶244:
¶269: ¶245: TF: When you see it from the top <questioning tone>
¶270: ¶246:
¶271: ¶247: AB: So it's just like this bit is kind of smaller, kind of closed in as you would expect, but not as much as it should be.
¶272: ¶248:
¶273: ¶249: TF: I’m still not quite clear on like let’s say, if I look at the bottom to the top and sat down again, there would be one continuous change from the one form to the other. How does that happen with the form? So if you look at it from the top and keep looking at it when you sit down again?
¶274: ¶250:
¶275: ¶251: AB: Yeah, it doesn’t really seem to change continuously.
¶276: ¶252:
¶277: ¶253: TF: Do you know how many steps? Is it just the two states and it flips from one to the other or are there some in between? Can you say how many?
¶278: ¶254:
¶279: ¶255: AB: Yeah, it's like there’s two here. So there’s one where the 4 is just like that and then is another one where it's kind of.
¶280: ¶256:
¶281: ¶257: TF: I’ve noticed that at the moment you are actually standing still. Can you see both of the different ones even though you are not moving?
¶282: ¶258:
¶283: ¶259: AB: Yeah, there’s kind of one like this and there’s one kind of like that.
¶284: ¶260:
¶285: ¶261: TF: So you have two different forms there <questioning tone>
¶286: ¶262:
¶287: ¶263: AB: Yeah.
¶288: ¶264:
¶289: ¶265: <pause>
¶290: ¶266:
¶291: ¶267: TF: So it seems like there are some preferred angles from which it can be viewed. Is that an accurate description?
¶292: ¶268:
¶293: ¶269: AB: Yeah. So it kind of moves a bit, in order to give me that angle. It's almost like the 4 just bends back there. <laughter>
¶294: ¶270:
¶295: ¶271: TF: Did you notice how many different states there were? I mean for example, if you just did a 45 degree angle or let’s say a straight on angle, would it be two different kinds of 4’s you are seeing or is it the same presentation?
¶296: ¶272:
¶297: ¶273: AB: Yeah, it's like they are slightly. Yeah, it's kind of hard to get a really clear view of it going on.
¶298: ¶274:
¶299: ¶275: TF: So what happened there when you took those two steps? Did your perceptions of the number change?
¶300: ¶276:
¶301: ¶277: AB: <pauses> Because I walk, I find it hard to focus my attention on the number so I tend to kind of physical room because the number kind of disappears a bit.
¶302: ¶278:
¶303: ¶279: TF: Oh but, this was one of the numbers that it's sitting on the table right. <AB agrees> So but it still not the same kind of space as the physical space <questioning tone>

¶304: ¶280:
¶305: ¶281: AB: No. It's kind of superimposed. Soon as my attention, but I’m aware of the 4 being there, but I can’t really keep focused on it.
¶306: ¶282:
¶307: ¶283: TF: Oh, so when you move, you look at the physical room but you need to focus your attention again to something that lay on top of it. <AB agrees> So what do you do in order to impose it onto the physical room?
¶308: ¶284:
¶309: ¶285: AB: Just say right I’m going to look for the 4 now! <laughter>
¶310: ¶286:
¶311: ¶287: TF: Do you actually have an internal dialogue going on when you tried that?
¶312: ¶288:
¶313: ¶289: AB: Erm, a voice. There’s like a decision.
¶314: ¶290:
¶315: ¶291: TF: Like an intention <questioning tone>
¶316: ¶292:
¶317: ¶293: AB: Yeah, an intention sort of before.
¶318: ¶294:
¶319: ¶295: TF: There are a couple of other things that I wanted to try. So is it still there?
¶320: ¶296:
¶321: ¶297: AB: Yeah.
¶322: ¶298:
¶323: ¶299: TF: What happens if you want to touch it? Have you ever tried that?
¶324: ¶300:
¶325: ¶301: AB: No. <pause> I don’t feel anything.
¶326: ¶302:
¶327: ¶303: TF: Does it stay there? Stable perception? <AB agrees> So what happens with your finger, does it go through it or going around it?
¶328: ¶304:
¶329: ¶305: AB: Goes around it. I’m just kind of stroking the outside of it.
¶330: ¶306:
¶331: ¶307: TF: But you don’t feel anything in your finger when you do that <questioning tone>
¶332: ¶308:
¶333: ¶309: AB: No. Run my finger through it.
¶334: ¶310:
¶335: ¶311: TF: Nothing?
¶336: ¶312:
¶337: ¶313: AB: No. So it's almost like a projection of the 4 or something.
¶338: ¶314:
¶339: ¶315: TF: But do you see your finger and the 4 the same time when you do that? So, with a projection you would have the 4 distorted by your finger.
¶340: ¶316:
¶341: ¶317: AB: <pauses> So now I just see my finger as being in the physical space and the 4 as being in the mental one.
¶342: ¶318:
¶343: ¶319: TF: Oh so, it doesn’t look like it's on the table anymore the 4 or <questioning tone>
¶344: ¶320:
¶345: ¶321: AB: Erm, yeah I just have to. Yeah I mean it's harder to see the 4 if I do that because my finger is in the way.
¶346: ¶322:
¶347: ¶323: TF: So it's harder to.
¶348: ¶324:
¶349: ¶325: AB: So it's just kind of like the other ones where I haven’t been able to see the numbers because the table is in the way.
¶350: ¶326:
¶351: ¶327: TF: Ok so it's hard for me to understand how to picture that because if it is that these are non overlapping spaces, then why should the physical object make a difference to how hard or how not hard they are to see?
¶352: ¶328:
¶353: ¶329: AB: Mmm. Well if my finger is in front of the 4, so it's almost like.

¶354: ¶330:
¶355: ¶331: TF: But if you switch your attention to the 4, then your perception of the physical world or is it because you can’t defocus your eyes?
¶356: ¶332:
¶357: ¶333: AB: It's almost like I’m peering into another dimension or something. And the finger is in front of the 4.
¶358: ¶334:
¶359: ¶335: TF: But the finger is in this dimension, not in the other dimension, right? I was just wondering how.
¶360: ¶336:
¶361: ¶337: AB: It’s like if this room is a 0 in one of the extra dimension and then the 4 is 1 or something.
¶362: ¶338:
¶363: ¶339: TF: <laughs> Why?
¶364: ¶340:
¶365: ¶341: AB: Then that number of fingers is 0 and I’m kind of and my face is -1 so then the finger is in front. <laughter>
¶366: ¶342:
¶367: ¶343: TF: Yes ok.
¶368: ¶344:
¶369: ¶345: AB: So the finger blocks it.
¶370: ¶346:
¶371: ¶347: TF: So is it because. Ok maybe I’m getting it now. Is it because when you transposed let’s say the number space, you or yourself are also in that space?
¶372: ¶348:
¶373: ¶349:
¶374: ¶350: AB: Erm,
¶375: ¶351:
¶376: ¶352: TF: So I mean you bring your body with you when you look at that number space. That’s why the finger is in front of the 4.
¶377: ¶353:
¶378: ¶354: AB: Erm, I’m still in the physical space because I see the finger.
¶379: ¶355:
¶380: ¶356: TF: You remember, I don’t know how many sessions ago, but you said it's almost like your head was in the <inaudible word> or looking at the numbers and so on. So this is what I mean, you seem to have a bodily representation and number space as well. <AB agreeing throughout> So I’m wondering is it your bodily representation that is interfering with the 4?
¶381: ¶357:
¶382: ¶358: AB: Yeah, I don’t know.
¶383: ¶359:
¶384: ¶360: TF: Ok let’s try this for example. Where is it?
¶385: ¶361:
¶386: ¶362: AB: It's here.
¶387: ¶363:
¶388: ¶364: TF: So what if I put my pen. Now the pen is not part of your body.
¶389: ¶365:
¶390: ¶366: AB: Pen is slightly behind the 4 at the moment. It's kind of going through the 4.
¶391: ¶367:
¶392: ¶368: TF: So it's in the.
¶393: ¶369:
¶394: ¶370: AB: So it's kind of obscuring some of the 4, but I can kind of see some of the 4 underneath the pen.
¶395: ¶371:
¶396: ¶372: TF: Ok, what about the number.
¶397: ¶373:
¶398: ¶374: AB: Yeah so it's almost like the 4 is behind the.
¶399: ¶375:
¶400: ¶376: TF: The pen <questioning tone>
¶401: ¶377:
¶402: ¶378: AB: Yeah, a bit like in another dimension.
¶403: ¶379:
¶404: ¶380: TF: So there is a kind of mix between the two somehow.
¶405: ¶381:
¶406: ¶382: AB: So it is.
¶407: ¶383:
¶408: ¶384: TF: How did the pen.
¶409: ¶385:
¶410: ¶386: AB: So it's like the 4 is in a different dimension. So it's 3D coordinates are in the same position as the pen.
¶411: ¶387:
¶412: ¶388: TF: So they have the same spatial frame of reference, but they have different references. So different instances of the same.
¶413: ¶389:
¶414: ¶390: AB: Yeah, so it's almost like you have this whole room and then there’s like a copy of this whole room.
¶415: ¶391:
¶416: ¶392: TF: And then this pen goes into that or. <questioning tone>
¶417: ¶393:
¶418: ¶394: AB: And then the pen stays in this room. But all the numbers and things are in the copy of this room.
¶419: ¶395:
¶420: ¶396: TF: So that’s overlaid onto this room. <questioning tone>
¶421: ¶397:
¶422: ¶398: AB: And so if there’s an object in this room, in the same place as one of the numbers are in the other room, then I see the object rather than the number.
¶423: ¶399:
¶424: ¶400: TF: So it has more presence somehow.
¶425: ¶401:
¶426: ¶402: AB: Yeah.
¶427: ¶403:
¶428: ¶404: TF: So is there an interference or something like that? Is it, sorry or was it here?
¶429: ¶405:
¶430: ¶406: AB: Just. It just blocks a bit of the 4. I don’t see a bit of the 4.
¶431: ¶407:
¶432: ¶408: TF: What if it's in the middle of it; does it shine through the 4?
¶433: ¶409:
¶434: ¶410: AB: Yes, if it's behind it's ok, because the 4 is just here.
¶435: ¶411:
¶436: ¶412: TF: Is there any thickness to the 4, so it could be you know in the 4 so to speak?
¶437: ¶413:
¶438: ¶414: AB: The 4 seems very thin.
¶439: ¶415:
¶440: ¶416: TF: Ok.
¶441: ¶417:
¶442: ¶418: AB: So I’m going a 4 in front.
¶443: ¶419:
¶444: ¶420: TF: Ok move slightly forward. Can you see how the object and the 4 interface? What happens at the borders?
¶445: ¶421:
¶446: ¶422: AB: Yeah, now the 4 is kind of. It's almost like it's going through the hole in the floor.
¶447: ¶423:
¶448: ¶424: TF: Ok, so if I go like this, just changing the angles slightly.
¶449: ¶425:
¶450: ¶426: AB: Now it's kind of got a gap. It’s kind of the pen is blocking it, a strip in the middle of the floor.
¶451: ¶427:
¶452: ¶428: TF: It's because the 4 doesn’t have any width basically.
¶453: ¶429:
¶454: ¶430: AB: No.
¶455: ¶431:
¶456: ¶432: TF: It's not like if it was like it was like thick and I could put an object inside it.
¶457: ¶433:
¶458: ¶434: AB: No.
¶459: ¶435:
¶460: ¶436: TF: Was there one around here which was what we called the corridor, any of the numbers that appeared?
¶461: ¶437:
¶462: ¶438: AB: Erm no, I didn’t notice a corridor.
¶463: ¶439:
¶464: ¶440: TF: No. Can you imagine one that’s not on the table, but it's in a completely different space.
¶465: ¶441:
¶466: ¶442: AB: Yeah, I’ve kind of got a corridor. Got a 6 against the wall of the corridor over here.
¶467: ¶443:
¶468: ¶444: TF: Here?
¶469: ¶445:
¶470: ¶446: AB: Yeah, right there.
¶471: ¶447:
¶472: ¶448: TF: And what does it look like?
¶473: ¶449:
¶474: ¶450: AB: Erm, it's got some patternings on it.
¶475: ¶451:
¶476: ¶452: TF: What, the crossings. <questioning tone>
¶477: ¶453:
¶478: ¶454: AB: Yeah.
¶479: ¶455:
¶480: ¶456: TF: Black and white?
¶481: ¶457:
¶482: ¶458: AB: Yeah black and white. All of the corridor is that kind of that orangey, greeny colour.
¶483: ¶459:
¶484: ¶460: TF: Ok the usual. And the light’s coming in from the side. Is it already placed on its number line or not?
¶485: ¶461:
¶486: ¶462: AB: Yeah, it's kind of a line here 5 4
¶487: ¶463:
¶488: ¶464: TF: Ok so, if I remember correctly the line is going through the wall. <questioning tone>
¶489: ¶465:
¶490: ¶466: AB: Yeah, so I can’t see the line beyond 6. So the 6 is parked right against the wall.
¶491: ¶467:
¶492: ¶468: TF: So, now this would be a space where if you attend to it, you are not looking at the physical room at all. <AB agrees> So what if I try to find this with my pen, does it interfere as well?
¶493: ¶469:
¶494: ¶470: AB: So 6 is here.
¶495: ¶471:
¶496: ¶472: TF: If I put here.
¶497: ¶473:
¶498: ¶474: AB: And can find 6.
¶499: ¶475:
¶500: ¶476: TF: Move a bit forward.
¶501: ¶477:
¶502: ¶478: AB: Can look at the pen or the 6.
¶503: ¶479:
¶504: ¶480: TF: So if you look at the 6, keep focus on the 6, what happens if I move the pen in there? It does interfere or?
¶505: ¶481:
¶506: ¶482: AB: No. I can just see the 6.
¶507: ¶483:
¶508: ¶484: TF: So there seems to be a difference between these two kinds of spaces.
¶509: ¶485:
¶510: ¶486: AB: Yeah.
¶511: ¶487:
¶512: ¶488: TF: Did you notice doing anything differently to evoke the one rather than the other?
¶513: ¶489:
¶514: ¶490: AB: Yeah, it's like I could stare back and pause the pen and the 6.
¶515: ¶491:
¶516: ¶492: TF: No, I mean when I asked you to bring forth one that was in a different space rather than let’s say on the table. Did you notice doing anything differently than getting one that appears overlaid into this space?
¶517: ¶493:
¶518: ¶494: AB: Just kind of had the intention of seeing some numbers in the corridor.
¶519: ¶495:
¶520: ¶496: TF: Sounds too easy to me. <laughter> With attention everything appears magically! So how flexible is it, so last time we had let’s say the tunnel. If it's just above your attention, what if I asked you could you have a number that’s in the tunnel?
¶521: ¶497:
¶522: ¶498: AB: What do you mean?
¶523: ¶499:
¶524: ¶500: TF: Well, we did it with the alphabet last time. Could you transpose, instead of having numbers in there. Sorry, instead of having letters in there could you have numbers in there?
¶525: ¶501:
¶526: ¶502: AB: In the tunnel <questioning tone>
¶527: ¶503:
¶528: ¶504: TF: Yes.
¶529: ¶505:
¶530: ¶506: AB: You mean a letter instead of a number?
¶531: ¶507:
¶532: ¶508: TF: No, I’m not talking about the corridor.
¶533: ¶509:
¶534: ¶510: AB: Oh.
¶535: ¶511:
¶536: ¶512: TF: You remember you said, I think it was. We were looking at a letter in the alphabet and then we were looking at something brown behind it and then it was brown. <AB agreeing throughout>
¶537: ¶513:
¶538: ¶514: AB: So, can I put a number in a cave.
¶539: ¶515:
¶540: ¶516: TF: Oh a cave, sorry, that’s what I mean!
¶541: ¶517:
¶542: ¶518: AB: Erm, so I’ve kind of got the cave over here. Hard to get a number in there. There’s no room because the alphabet is in the way.
¶543: ¶519:
¶544: ¶520: TF: Oh it's already in there? <laughter>
¶545: ¶521:
¶546: ¶522: AB: Yeah, it's right in there.
¶547: ¶523:
¶548: ¶524: TF: I’m just trying to scope out the different intentions. I guess it's not just about the intentions right. Something seems to be easier and fit together and some things not. Because why can’t you just have an intention saying I want a cave with a number?
¶549: ¶525:
¶550: ¶526: AB: Because the numbers live in the corridor <laughter>
¶551: ¶527:
¶552: ¶528: TF: Can you try just to see? Might be more effortful, but the question is it impossible or does it just take more effort?
¶553: ¶529:
¶554: ¶530: AB: So I kind of get like a cylindrical kind of tunnel. It's kind of black. There’s kind of a 6 at the end of it.
¶555: ¶531:
¶556: ¶532: TF: Do they look similar, the one with the alphabet in it and the one with the numbers?
¶557: ¶533:
¶558: ¶534: AB: Erm, not really.
¶559: ¶535:
¶560: ¶536: TF: So it's darker <questioning tone>
¶561: ¶537:
¶562: ¶538: AB: Yeah, it's darker and more cylindrical than the passage.
¶563: ¶539:
¶564: ¶540: TF: So you see the end of it. <questioning tone>
¶565: ¶541:
¶566: ¶542: AB: No, the 6 is occupying the whole thing.
¶567: ¶543:
¶568: ¶544: TF: Ah ok, so it's blocking the view.
¶569: ¶545:
¶570: ¶546: AB: Yeah, a big fat 6. Then there is kind of an axis here with the numbers going 5 6 7 8 9.
¶571: ¶547:
¶572: ¶548: TF: Is it black because of a lack of light or because of the material that it is made of?
¶573: ¶549:
¶574: ¶550: AB: It's black because of the material it's made of.
¶575: ¶551:
¶576: ¶552: TF: Can you see the material?
¶577: ¶553:
¶578: ¶554: AB: It's kind of black, shiny black.
¶579: ¶555:
¶580: ¶556: TF: Oh so it's not natural.
¶581: ¶557:
¶582: ¶558: AB: No, it's not like a natural environment. It’s more kind of manmade. Whereas the 3 was in a damp, brownish cave.
¶583: ¶559:
¶584: ¶560: TF: Can you see the floor? Is it the same all the way around.
¶585: ¶561:
¶586: ¶562: AB: Yeah, it's circular.
¶587: ¶563:
¶588: ¶564: TF: And it's the same texture.
¶589: ¶565:
¶590: ¶566: AB: Yeah.
¶591: ¶567:
¶592: ¶568: TF: Last time we had a look at what was at the entrance at the cave. We never did that for the corridor to see where the light was coming from the sides. <AB agreeing throughout> Do you want to have a look?
¶593: ¶569:
¶594: ¶570: AB: Yeah, I mean it's just a square end of the corridor and it's white.
¶595: ¶571:
¶596: ¶572: TF: But where is it coming from?
¶597: ¶573:
¶598: ¶574: AB: The light <questioning tone>
¶599: ¶575:
¶600: ¶576: TF: Yeah.
¶601: ¶577:
¶602: ¶578: AB: Just outdoors I guess.
¶603: ¶579:
¶604: ¶580: TF: If you turned can you see the light?
¶605: ¶581:
¶606: ¶582: AB: Yeah.
¶607: ¶583:
¶608: ¶584: TF: Can you see where it's coming from?
¶609: ¶585:
¶610: ¶586: AB: No, it's almost just like, just imagine a white space out there.
¶611: ¶587:
¶612: ¶588: TF: Can you get close to it?
¶613: ¶589:
¶614: ¶590: AB: Yeah.
¶615: ¶591:
¶616: ¶592: TF: Did anything change?
¶617: ¶593:
¶618: ¶594: AB: Just white.
¶619: ¶595:
¶620: ¶596: TF: So it's not like you can look down on it.
¶621: ¶597:
¶622: ¶598: AB: The wall’s kind of annoying. <laughter>
¶623: ¶599:
¶624: ¶600: TF: But before in the other case you could actually see a floor.
¶625: ¶601:
¶626: ¶602: AB: For a moment I got some kind of blue sky type thing.
¶627: ¶603:
¶628: ¶604: TF: Is that the other one?
¶629: ¶605:
¶630: ¶606: AB: Yeah. Sometimes it goes kind of blue with white puffy clouds going by.
¶631: ¶607:
¶632: ¶608: TF: So it's a natural light, it's not an artificial light.
¶633: ¶609:
¶634: ¶610: AB: So it is like a natural light.
¶635: ¶611:
¶636: ¶612: TF: I mean it's interesting that there’s two lights for this one and there’s only one light for the alphabet. <AB agrees> Well there was only one light for the artificial tunnel you were looking at the 6. Was it coming from behind?
¶637: ¶613:
¶638: ¶614: AB: I kind of feel like I’m looking through a little tube actually. So I’m looking down a little telescope or something at the 6 and then the rest of the numbers are against a red background.
¶639: ¶615:
¶640: ¶616: TF: Oh so it's red rather than.
¶641: ¶617:
¶642: ¶618: AB: So it's just something with a big red space and I’m looking down a little tube that’s the 6. I’ve got this kind of <inaudible due to external noise> something made out of black. I’m just looking down there at the 6 there. And the other numbers are kind of connected to the 6 on a number line, going like this.
¶643: ¶619:
¶644: ¶620: TF: You said that the alphabet doesn’t have a number line because there’s no continuous values. <AB agrees> So one thing that would interest me, if you look at the H on the page. There’s several perspectives that we can take on it. We can look at what it's made of, in this case some ink and we can see paper and we can see some of the markings of the page showing through. But then we can also see it as a symbol for the letter H, but the symbol is made out this stuff that is the page, we can either focus what it's made of or the symbol. So did you have a B somewhere or what was it?
¶645: ¶621:
¶646: ¶622: AB: Yeah B is right there somewhere.
¶647: ¶623:
¶648: ¶624: TF: If you look at the B can you see what it is made of?
¶649: ¶625:
¶650: ¶626: AB: Don’t know what material it's made of, or if it's solid at all.
¶651: ¶627:
¶652: ¶628: TF: But does it appear on a background out of which you know?

¶653: ¶629:
¶654: ¶630: AB: Just kind of standing up in the cave. It seems a bit kind of like vague, like it could just be rejection of the B or something. Well it is standing up.
¶655: ¶631:
¶656: ¶632: TF: So when you say standing up, does that mean you feel like it's a physical object or it looks like its upright?
¶657: ¶633:
¶658: ¶634: AB: It looks like its upright. Well it's not against a wall or anything so I don’t see how it could be a projection. But it seems like it's almost like a ghost, like fingers through it.
¶659: ¶635:
¶660: ¶636: TF: Like we did with the woman.
¶661: ¶637:
¶662: ¶638: AB: Yeah. I keep moving the context of the cave. The cave I feel that the walls are hard and kind of damp and muddy. But the B is kind of translucent, ghostlike.
¶663: ¶639:
¶664: ¶640: TF: So does that mean you can see through it, the cave?
¶665: ¶641:
¶666: ¶642: AB: Can’t see through it.
¶667: ¶643:
¶668: ¶644: TF: Didn’t you say it was translucent?
¶669: ¶645:
¶670: ¶646: AB: It's kind of black but I get a feeling that if I touched it, my finger would just go through it.
¶671: ¶647:
¶672: ¶648: TF: Well, have you tried touching the cave wall?
¶673: ¶649:
¶674: ¶650: AB: Well I mean I would just go like that wouldn’t I.
¶675: ¶651:
¶676: ¶652: TF: Is there any difference between touching the H and the wall.
¶677: ¶653:
¶678: ¶654: AB: The B.
¶679: ¶655:
¶680: ¶656: TF: Sorry the B.
¶681: ¶657:
¶682: ¶658: AB: Not really.
¶683: ¶659:
¶684: ¶660: TF: It just goes through?
¶685: ¶661:
¶686: ¶662: AB: Yeah.
¶687: ¶663:
¶688: ¶664: TF: So in that case, would you say that there’s a perceptual difference looking at the H and the B, in a sense this seems to be made out of something and the B doesn’t seem to be made out of anything?
¶689: ¶665:
¶690: ¶666: AB: Erm, well I can focus on the individual lines of the B. I mean if I really focus on it. Now it's like it's made of ribbons attached to each other.
¶691: ¶667:
¶692: ¶668: TF: Ribbons! <questioning tone>
¶693: ¶669:
¶694: ¶670: AB: They’re not very stable.
¶695: ¶671:
¶696: ¶672: TF: Ok, well I didn’t mean to like focus. In this case looking at the H on the page, we don’t have to stress about seeing that it's made out of ink and paper, I was wondering whether you get something similar in terms of the B.
¶697: ¶673:
¶698: ¶674: AB: Erm yeah, not immediately. I mean really staring it seems like it changes what it's made of a bit.
¶699: ¶675:
¶700: ¶676: TF: Right.
¶701: ¶677:
¶702: ¶678: AB: Yeah, at the beginning I wasn’t resembling any kind of man made substance or a natural substance of the world.
¶703: ¶679:
¶704: ¶680: TF: Yeah, you were trying to describe it as a projection and it didn’t quite fit. <AB agrees> So the background actually seems more natural right?
¶705: ¶681:
¶706: ¶682: AB: Yeah, the background seems more natural. B has a kind of ghostlike quality to it.
¶707: ¶683:
¶708: ¶684: TF: Is it the same with, what was it the 6?
¶709: ¶685:
¶710: ¶686: AB: Mmm. Erm, now what’s the 6 made of? It's like a kind of ghostlike character really.
¶711: ¶687:
¶712: ¶688: TF: It's the same kind of presence as the B?
¶713: ¶689:
¶714: ¶690: AB: Yeah.
¶715: ¶691:
¶716: ¶692: TF: And in this case, the background looked a bit more manmade. But it looks like it's a manmade quality we can find in the world.
¶717: ¶693:
¶718: ¶694: AB: Mmm, it's a bit ghostlike as well. I mean its wall is kind of a bit fuzzy.
¶719: ¶695:
¶720: ¶696: TF: So unlike the cave.
¶721: ¶697:
¶722: ¶698: AB: Like a sort of hard boundary like the table, it's kind of slightly foggy.
¶723: ¶699:
¶724: ¶700: TF: Ok. What about the corridor, is it still there somewhere?
¶725: ¶701:
¶726: ¶702: AB: Yeah.
¶727: ¶703:
¶728: ¶704: TF: Does it also look. That was more stable right?
¶729: ¶705:
¶730: ¶706: AB: That’s what I’m saying. The wall of the corridor is a bit fuzzy, so it's not like a hard surface like the table, it's kind of like. 6 is by there, so if you put anything behind the 6 it would go into the fog and you wouldn’t be able to see it.
¶731: ¶707:
¶732: ¶708: TF: Or wasn’t it the 6 in the two you were describing?
¶733: ¶709:
¶734: ¶710: AB: Oh the 1 6 in the corridor and then there’s another 6 in the tube.
¶735: ¶711:
¶736: ¶712: TF: Oh right.
¶737: ¶713:
¶738: ¶714: AB: Yeah, I was looking at the 6 in the corridor.
¶739: ¶715:
¶740: ¶716: TF: What about the 6 in the tube?
¶741: ¶717:
¶742: ¶718: AB: Erm, yeah I mean it's kind of ghostlike as well. Yeah, so the tube is this kind of manmade thing and the 6 is ghostlike.
¶743: ¶719:
¶744: ¶720: TF: So both the corridor and the tube, both look manmade but they don’t have this kind of presence as the cave does?
¶745: ¶721:
¶746: ¶722: AB: Erm, no they have the same kind of presence.
¶747: ¶723:
¶748: ¶724: TF: I mean in terms of these two backgrounds seem to be more I don’t know, transparent, not solid.
¶749: ¶725:
¶750: ¶726: AB: Yeah. Well the walls of the tube are pretty solid. The wall of the corridor is a bit kind of best defined as hard.
¶751: ¶727:
¶752: ¶728: TF: Well in terms of the cave you had a strong sensation of.
¶753: ¶729:
¶754: ¶730: AB: Yeah almost like dampness.
¶755: ¶731:
¶756: ¶732: TF: The whole material properties of it. But the material properties are less strongly expressed than the other two cases. Is that <inaudible> (51.24)
¶757: ¶733:
¶758: ¶734: AB: Erm the material property of the tube is pretty strongly expressed. It's just a hard plastic kind of tube. It's just kind of there looking at the 6 and then all the other numbers are against a big red background.

¶759: ¶735:
¶760: ¶736: TF: When you say red background do you mean it's like a wall?
¶761: ¶737:
¶762: ¶738: AB: Yeah like a wall.
¶763: ¶739:
¶764: ¶740: TF: Is it painted red?
¶765: ¶741:
¶766: ¶742: AB: Kind of bright paint. Kind of very solid red.
¶767: ¶743:
¶768: ¶744: TF: So it's made out of something red?
¶769: ¶745:
¶770: ¶746: AB: There’s again all that slightly ghostlike quality of the wall of the corridor where it's kind of, doesn’t feel hard but, feels you can penetrate it very far.
¶771: ¶747:
¶772: ¶748: TF: Did you ever notice any other kinds of background for the other forms that you have for example, years or I guess that clock could always be manmade. <AB agrees> So how hard would it be to interface a clock with this room, if this room doesn’t have a clock?
¶773: ¶749:
¶774: ¶750: AB: Well, I mean a clock just kind of appears here.
¶775: ¶751:
¶776: ¶752: TF: What in the middle of the room, not against the wall?
¶777: ¶753:
¶778: ¶754: AB: No.
¶779: ¶755:
¶780: ¶756: TF: What kind of clock is it?
¶781: ¶757:
¶782: ¶758: AB: Just a clock with hands.
¶783: ¶759:
¶784: ¶760: TF: So it's just a brown one. <questioning tone>
¶785: ¶761:
¶786: ¶762: AB: It's kind of just a white face, black dashes for each of the, for each minute. The bigger ones for every five minutes and also the hours. It's not labelled with any numbers on it. It's got the two hands.
¶787: ¶763:
¶788: ¶764: TF: Ok.
¶789: ¶765:
¶790: ¶766: AB: Showing quarter to eleven, sorry.
¶791: ¶767:
¶792: ¶768: TF: Quarter to eleven. Are the hands moving?
¶793: ¶769:
¶794: ¶770: AB: No.
¶795: ¶771:
¶796: ¶772: TF: Do you ever see any movement in any of your forms?
¶797: ¶773:
¶798: ¶774: AB: Erm, not other than the ones we’ve been described.
¶799: ¶775:
¶800: ¶776: TF: I mean perspectives shift.
¶801: ¶777:
¶802: ¶778: AB: Yeah, perspectives shift.
¶803: ¶779:
¶804: ¶780: TF: I remember we had once, you know. Looked up and down the numbers from two different perspectives. Can we just try that again to see how it compares to last time? Do you have any number line ready that we could do that with?
¶805: ¶781:
¶806: ¶782: AB: Erm, I’ve got all sorts of number lines. <laughter>
¶807: ¶783:
¶808: ¶784: TF: I don’t know, which one looks attractive to you?
¶809: ¶785:
¶810: ¶786: AB: Erm, I’ve got one just right here.
¶811: ¶787:
¶812: ¶788: TF: Are you saying it's starting with 1?
¶813: ¶789:
¶814: ¶790: AB: Yeah.
¶815: ¶791:
¶816: ¶792: TF: Erm ok, so good choice. If you look up the numbers, going bigger, can you see the 2 the 3?
¶817: ¶793:
¶818: ¶794: AB: Yeah, 1 2 3 turns about here. 20
¶819: ¶795:
¶820: ¶796: TF: Ok. So, now as I understand your number line, the negative numbers also start around where the 1 is close by.
¶821: ¶797:
¶822: ¶798: AB: Yeah, the negative ones will be the ones here. Around the 0 -1.
¶823: ¶799:
¶824: ¶800: TF: Ok. Let’s try this then. What if you position yourself at the 0. Can you do that, can you see the 0? What numbers do you see next to it?
¶825: ¶801:
¶826: ¶802: AB: So there’s a 1 here and a -1 there.
¶827: ¶803:
¶828: ¶804: TF: Ok. So, how did we do it last time? So can you look at the 0 coming from the positive numbers?
¶829: ¶805:
¶830: ¶806: AB: Looking here, so kind of 1 0
¶831: ¶807:
¶832: ¶808: TF: And what about from the negative numbers?
¶833: ¶809:
¶834: ¶810: AB: Yeah, 0 -1 -2, 0 is there.
¶835: ¶811:
¶836: ¶812: TF: So it's equally easy to go both ways? I mean except for the fact the negative numbers move <cross talking>
¶837: ¶813:
¶838: ¶814: AB: Kind of move. Sometimes I can do it with just sitting still, I can shift perspective.
¶839: ¶815:
¶840: ¶816: TF: But sometimes movement helps. <questioning tone>
¶841: ¶817:
¶842: ¶818: AB: Yeah, movement is definitely easier.
¶843: ¶819:
¶844: ¶820: TF: So, what do you do in that case? You focus your attention in the number you are interested in and you just move in respect to it?
¶845: ¶821:
¶846: ¶822: AB: Yeah. I can just move. I can just set a particular number right in front of me, I guess.
¶847: ¶823:
¶848: ¶824: TF: Yeah but how?
¶849: ¶825:
¶850: ¶826: AB: A different thing during the hypnosis session where I was kind of floating above number line. I was kind of looking down at it. I was facing the direction that was being counted.
¶851: ¶827:
¶852: ¶828: TF: That doesn’t happen normally. <questioning tone>
¶853: ¶829:
¶854: ¶830: AB: Not that today.
¶855: ¶831:
¶856: ¶832: TF: You mean you were floating along it from the top?
¶857: ¶833:
¶858: ¶834: AB: From above yeah.
¶859: ¶835:
¶860: ¶836: TF: But did the numbers look as if they were <inaudible word> from the top or were they presenting themselves as if you were looking at them from the front?
¶861: ¶837:
¶862: ¶838: AB: Yeah, I was looking at them from the front. But they kind of turned around for me. <laughter>
¶863: ¶839:
¶864: ¶840: TF: Right, shall we leave it there for today?
¶865: ¶841:
¶866: ¶842: AB: Yeah.
¶867: ¶843:
¶868: ¶844: **End of interview.**
