# Supplementary material for: An extended case study on the phenomenology of sequence-space synesthesia
Source: Front Hum Neurosci. 2014 Jul 3;8:433. doi: 10.3389/fnhum.2014.00433 (PMC4080762; doi:10.3389/fnhum.2014.00433)
Supplement: Supplementary file 1 [file Presentation1.ZIP › Supp Mat 2.ZIP!/Referenced_Interview_Transcipts/AB - 20100415.doc]

**Name:** AB - 20100415

**Description:** Dialogue between TF and AB on 15/04/2010

¶1: **Dialogue between TF and AB on 15/04/2010**

¶2: (0.00)

¶3: TF: Ok, so to begin have a look at this page here. What does that do to you?
¶4:

¶5: AB: So initially it's just, I can see a number 4 and then a couple of seconds later there’s a 3 there and a 5 there.

¶6:
¶7: TF: Ah so it's very integrated or is there like a synaesthetic form as well?
¶8:
¶9: AB: No, there’s no synaesthetic form
¶10:
¶11: TF: Ok so it's kind of like in the middle where it should be <questioning tone>

¶12: AB: Yeah.
¶13:
¶14: TF: What about now? What happens to the other two now that I’ve moved the page away?
¶15:
¶16: AB: Now I’ve got a 3 4 5
¶17:
¶18: TF: Ok the 4 has come up <questioning tone>
¶19:
¶20: AB: Yeah
¶21:
¶22: TF: Ok so the 3 is about here?
¶23:
¶24: AB: Yeah.
¶25:
¶26: TF: Can you describe it for me?
¶27:
¶28: AB: Erm, it's kind of black, just floating in space.
¶29:
¶30: TF: In the room space <questioning tone>
¶31:
¶32: AB: Yeah, I guess the room space <quiet>
¶33:
¶34: TF: So you see the notebook that’s on the table behind it and everything?
¶35:
¶36: AB: Erm, it's like I can either pay attention to the notebook or the 3, so don’t really focus on one or the other.
¶37:
¶38: TF: So they’re not present at the same time?
¶39:
¶40: AB: No.
¶41:
¶42: TF: So when you do look at the 3 what else is present around it?
¶43:
¶44: AB: The 4 and 5.
¶45:
¶46: TF: But I guess what are the distinguished?
¶47:
¶48: AB: Erm… <pauses>
¶49:
¶50: TF: I mean for instance, I guess it's not black because otherwise how would you be able to tell where the number begins and where it ends like the 4.
¶51:
¶52: AB: Yeah. <pauses> Yeah, these are bit more vague these ones. They are just kind of more like the feeling or the presence of the 3 there.
¶53:
¶54: TF: So it’s not on a white page for example <questioning tone>
¶55:
¶56: AB: No.
¶57:
¶58: TF: Is it a bit blurred?
¶59:
¶60: AB: Erm, well I can sort of see a…see it in black. I mean perhaps anything that’s probably kind of like a purplish background behind it. It's not really smooth; it's kind of a bit lumpy sort of texture. It seems like I only brought that into awareness sort of much later and perhaps not aware of that at all.
¶61:
¶62: TF: Ok. So what do you have to do in order to bring a more stable number experience?
¶63:
¶64: AB: Erm, well I just think about it for quite a long time.
¶65:
¶66: TF: But I mean sometimes they seem to be appearing much more spontaneously and much more vividly as well. I don’t know, maybe what about if it's more an open space, that’s say in the middle of the room <questioning tone>
¶67:
¶68: AB: Yeah, so it's much bigger 3 over there.
¶69:
¶70: TF: Can you see it more clearly?
¶71:
¶72: AB: Yeah, it's kind of black and a few white markings on it, a white sort of spot. It's like a poor quality photocopy <laughter>. Erm, yeah it's kind of on a white page. No the 4 and 5 are sort of in the position that they are usually in, above and to the right of it. They’re just sort of more floating in the room.
¶73:
¶74: TF: The 3 as well or do you mean all of them?
¶75:
¶76: AB: All of them yeah.
¶77:
¶78: TF: So you can look at them and the room is still present at the same time?
¶79:
¶80: AB: Erm, just a focus of attention. I mean the room is there but kind of blurry. My eyes are focused in the middle of the room I think.
¶81:
¶82: TF: Ok, I can kind of try to replicate that if I just focus my eyes on a. Well for me an empty spot in the middle of the room. The other thing to do is come out of focus <AB agreeing throughout>.
¶83:
¶84: <pause>
¶85: TF: Are the other two numbers also marked with white spots?
¶86:
¶87: AB: Erm, they are a bit more vague. They haven’t got the background behind them, so I can’t see them as well.
¶88:
¶89: TF: Are there white pages missing?
¶90:
¶91: AB: Yeah.
¶92:
¶93: TF: So what if you shift your focus to, let’s say 4. Does it become more vivid?
¶94:
¶95: AB: Erm, it's a bit of a thinner sort of stroke than the 3 and it's kind of got a slight curve in the line. Sort of angle like.
¶96:
¶97: TF: You mean like the right downward stroke is a little bit curved <questioning tone>
¶98:
¶99: AB: Yeah, it goes from the top to the bottom left and is slightly curved. Kind of thinner, still black and yet sort of floating in a kind of a sea space. Yeah, it's kind of like a sort of a turquoisey background. Not really sure what colours … <inaudible> (6.14)
¶100:
¶101: TF: Do you mean the background in terms of what the white page is the background for the 3 or do you mean the more general background?
¶102:
¶103: AB: No, a bigger background.
¶104:
¶105: TF: And does this turquoise overlay the room?
¶106:
¶107: AB: Er, yeah.
¶108:
¶109: TF: So it makes the room a little bit coloured <questioning tone>
¶110:
¶111: AB: Oh no it doesn’t make the room coloured. It’s just like another world that I can focus my attention on or I can bring my attention back to the room.
¶112:
¶113: TF: Ok so, because we talking about the 3, it was the room that was a little bit blurred in the background. But not that you could shift your attention to the fore. Actually it's when you put your attention to it; you are actually focusing on the synaesthetics space rather than the room space. <AB agreeing throughout>
¶114:
¶115: TF: How come it was a different background for the 3 and the 4?
¶116:
¶117: AB: I don’t know.
¶118:
¶119: TF: It just changes?
¶120:
¶121: AB: I think it's because the 3 you know I looked at first.
¶122:
¶123: TF: And then when you got more into it you shifted your attention more to the synaesthetics space.
¶124:
¶125: AB: Yeah. <pauses> I never get more than one number at a time appearing on the white background. When I see the number line that’s always against the more kind of coloured background.
¶126:
¶127: TF: Right. So because there was maybe. So you were starting to look at them as a sequence rather than just one particular number.
¶128:
¶129: AB: Yeah exactly.
¶130:
¶131: TF: Ok so now that you have had a few stable ones here, can you bring one up more closely?
¶132:
¶133: AB: So I’ve got a 2 here.
¶134:
¶135: TF: Ok a 2. So do you remember what we did last time of trying to look at it from different angles to see what happens?
¶136:
¶137: AB: Yeah.
¶138:
¶139: TF: Maybe we can have a recap of that. So how does it change when you stand up for example?
¶140:
¶141: AB: <pauses> Yeah looks like I am kind of above it, kind of like this. It's kind of got some depth to it this time.
¶142:
¶143: TF: So a bit more chunky in depth?
¶144:
¶145: AB: Yeah.
¶146:
¶147: TF: So did it again angle backwards as you got up?
¶148:
¶149: AB: Erm, not really I don’t think. Maybe a little bit.
¶150:
¶151: TF: So it looks exactly as if it was a 2 standing up straight off the table <questioning tone>
¶152:
¶153: AB: Pretty much.
¶154:
¶155: TF: Oh yeah if you go exactly above it.
¶156:
¶157: AB: Kind of disappeared now.
¶158:
¶159: TF: I bet it had some <inaudible> (9.08) before right?
¶160:
¶161: AB: Yeah. Seems to be pretty much vertical. I mean if anything, I’d sort of see a thick line there or two should be.
¶162:
¶163: TF: So and.
¶164:
¶165: AB: I am also seeing a 2 like that as well simultaneously.
¶166:
¶167: TF: Oh, like the same spot, as if it was lying on the table <questioning tone>
¶168:
¶169: AB: Yeah.
¶170:
¶171: TF: So when did that one appear?
¶172:
¶173: AB: Just now. And I was trying to work out what I was seeing. It gets a bit fuzzy when I’m standing directly above it.
¶174:
¶175: TF: Right.
¶176:
¶177: AB: It’s sort of different images coming into my mind. One is just of erm top of the 2. Another is it with it slightly angled. Then another is the 2 just flat on the table.
¶178:
¶179: TF: Right. So at least three different kinds of fitting here.
¶180:
¶181: AB: Yeah. That’s just sort of less stable.
¶182:
¶183: TF: If you sit down, what happens with them?
¶184:
¶185: AB: So, I’ve got a 2 flat on the table now. Then I’ve got the other one is kind of there standing up and <inaudible> (10.31) through to.
¶186:
¶187: TF: So in terms of the one that is standing up, when you got up you stood for quite a long time looking down on it before you said something <AB agreeing throughout>. What were you doing during that time?
¶188:
¶189: AB: I wasn’t really aware of that much. I mean the presence of the 2 and could only see the table top.
¶190:
¶191: TF: So it took a while for it to become present again <questioning tone>. So were you actually officially aware of the 2 while you were rising from your chair?
¶192:
¶193: AB: While I was rising. No, I think I was a bit distracted.
¶194:
¶195: TF: Ok, shall we try it again? If you just focus on the one that’s standing up and then just rise up while looking at it. What happens to it? (11.17)
¶196:
¶197: <pause>
¶198: AB: Yeah, it seemed to jump for a moment like flat on the table. And then sort of comes back and is standing up. So it's kind of hard to stand up slowly enough to <laughter>
¶199:
¶200: TF: Why do you say that? I mean I am curious, you trying to stand up slowly in order to see what?
¶201:
¶202: AB: Yeah, where some kind of change happens.
¶203:
¶204: TF: You think speed has something to do with it?
¶205:
¶206: AB: Don’t know. So it seems to flicker. I’m getting images of a 2 going just flat.
¶207:
¶208: TF: Flat on the table <questioning tone>
¶209:
¶210: AB: Yeah, but then it kind always come back up again.
¶211:
¶212: TF: So when it lies flat on the table, that’s present throughout the movement, is that true?
¶213:
¶214: AB: Erm, well it wasn’t there the first time I was sort of sitting down.
¶215:
¶216: TF: Well, the first time I suppose I recall you said you were distracted, so you didn’t actually see anything and that’s why when you were stood looking at it from the top down it took a while to come back. But then it created this flat one on the table and that’s stayed there when you were again sitting down and now when you were getting up <AB agreeing throughout>. So, I mean I don’t want to speculate too much, but it doesn’t make sense that if it takes too much effort to kind of like see all the different possible angles too. If you put it flat on the table, the perspective is hooked onto a physical object simply. So it's, because the surface of the table will basically give a reference point. I’m not sure whether there is something to that. But, that’s why I was interested in why whether when you were getting up or down, we know that standing up to it was flicking in and out of existence, but was the one flat on the table present the whole time now in the second round when you were standing up and down?
¶217:
¶218: AB: No, it just came into view when I sort of reached there at a certain angle.
¶219:
¶220: TF: Is it not on the table right now?
¶221:
¶222: AB: It's sort of flickering.
¶223:
¶224: TF: Right. And then when start moving at some point it appears on the table <questioning tone>
¶225:
¶226: AB: Erm…
¶227:
¶228: TF: Or it, sorry it stabilises?
¶229:
¶230: AB: Well that was what happened last time. Erm, because you told me to focus on the vertical 2.
¶231:
¶232: TF: Right yes. When you were doing that, you focusing on the vertical, did it at any time disappear and then the other one?
¶233:
¶234: AB: Yeah, when I got to a certain height.
¶235:
¶236: TF: And then when you were at the top basically stood up, the standing up one came back.
¶237:
¶238: AB: Yeah, yeah and then continued to alternate with the <unclear overtalking> (15:16)
¶239:
¶240: TF: And then both of them
¶241:
¶242: AB: Yeah.
¶243:
¶244: TF: Ok that’s interesting. So do you still have the 2 over there?
¶245:
¶246: AB: Yeah.
¶247:
¶248: TF: The standing up one? <AB agrees> Now, here’s a little experiment, what if I ask you to say imagine or visualise a 2 next to it. How would that look different to the 2 that’s already there?
¶249:
¶250: AB: Erm, just kind of like a duplicated, number 22 right now.
¶251:
¶252: TF: <laughter> Ok, but you know, say for example imagine your desk in your office. Kind of gives you a visual experience I think. So, if I say imagine a 2 on the table, is there any difference between those two experiences?
¶253:
¶254: AB: Erm, well the 2 on the table is kind of, seems to be in an indefinite position. Well I mean it seems to be supported by the, just then the image of my office is kind of somewhere over here. I was sort of I don’t. It's like I’m sort of standing in the middle of the office and the desk was sort of over here. The whole office was sort of smaller than the real life.
¶255:
¶256: TF: <laughter> I get that too actually!
¶257:
¶258: AB: Back to locate it within this physical room and my whole desk would sort of fit in around here.
¶259:
¶260: TF: In that basis, I can relate to you experience because you know if I thought of like the office I would probably visualise it slightly up and maybe to the right and it would be more smaller. So the question is then, if that’s like a visualise an output of the imagination, what are the qualitative differences between that and say imagining a number. So for example, I could try to imagine a 2 on this table, it's just that it would be a very unstable, very fleeting image. So I wonder if you try to imagine the 2, does it look exactly the same as the kind of like you know spontaneous 2 that appeared previously?
¶261:
¶262: AB: It took a while to stabilise so I got sort of repeats of the 2 that had been there for a while on either side and then in the end the new one was sort of thinner and blacker.
¶263:
¶264: TF: Ok, so is it still there?
¶265:
¶266: AB: Yeah.
¶267:
¶268: TF: So can it be part of a number line?
¶269:
¶270: AB: Erm, not really on a stable number line.
¶271:
¶272: TF: So it doesn’t appear with a 1 and a 3?
¶273:
¶274: AB: No, think I am a bit confused about whether it's a 2 2 or a 22
¶275:
¶276: TF: <laughter> Ok well assuming that it's a 22, if you let your gaze drift towards the right you should encounter the 23 if it is a number line.
¶277:
¶278: <Pauses>
¶279: AB: Yeah, it's bit unstable. So I can kind of see a 23 here and a 24 there, but I’m also getting like a 21 here and a 23 above it.
¶280:
¶281: TF: Oh there’s two different! A bit of a confusion going on between the different number lines then?
¶282:
¶283: AB: Yeah.
¶284:
¶285: TF: Ok maybe it was not a good idea to have them right next to each other. <laughter>
¶286:
¶287: AB: No, I did get a flickering 1 3 here linking up with the second 2.
¶288:
¶289: TF: What the newer one, the newer 2?
¶290:
¶291: AB: Yeah.
¶292:
¶293: TF: Are they also more slim characters?
¶294:
¶295: AB: Yeah.
¶296:
¶297: TF: So they fit into the style of the 2?

¶298: AB: Yeah.
¶299:
¶300: TF: I wonder how to probe this further, because as far as I understand from you there doesn’t seem to be any difference between the two kinds of numbers. So sometimes they appear voluntarily, but you can also make them appear by willing it to be so. And between these two conditions the end result doesn’t seem to be qualitatively different. Well there’s a little bit of an autonomy in the output too as well, because you said the new 2, even though I asked you to try to imagine another 2 like the one that was there, appeared slimmer than the 2 that is already there. <AB agrees throughout> If you look at both of them now, do they have the same texture?
¶301:
¶302: AB: No, so the one on the left is kind of more shiny and sort of metallic, the other one is just kind of black. Kind of soft texture.
¶303:
¶304: TF: Was this the one that had the white spots on it?
¶305:
¶306: AB: No, that was a 3 in the middle of the room.
¶307:
¶308: TF: Oh that’s right. Sorry could you please repeat that, the left one has the shiny black texture.
¶309:
¶310: AB: Shiny kind of metallic sort of shine to it. Not really that black.
¶311:
¶312: TF: It's more metallic than black.
¶313:
¶314: AB: Yeah.
¶315:
¶316: TF: It was the other one that’s black, the first one <questioning tone>
¶317:
¶318: AB: No, no the newer smaller one is black.
¶319:
¶320: TF: Ok, so the left one is the newer one right?
¶321:
¶322: AB: The right is the newer one.
¶323:
¶324: TF: oK, that’s what. So the right is the newer one and it's the smaller black.
¶325:
¶326: AB: Smaller, thinner black, sort of just jet black.
¶327:
¶328: TF: The older one that spontaneously appeared.
¶329:
¶330: AB: Is next to it, and a slightly metallic shine to it.
¶331:
¶332: TF: Can you see what kind of metal it is?
¶333:
¶334: AB: Just kind of a dark metal. There’s a bit of a kind of purple colouring in it, like one of the 5’s I saw a long time ago.
¶335:
¶336: TF: So visually when you look at both of them comparatively, they’re quite distinct in terms of their appearance <AB agrees> Ok shall we try it again to see what happens. Maybe you could try aiming a bit further away so we don’t get the interference directly. What if we try to get another 2 where you try to get the metallic one which was the first one right? Try to imagine the metallic one in a different location.
¶337:
¶338: AB: Yeah, I got one over there in the far desk.

¶339: TF: Right. Ok, and what does this one look like?
¶340:
¶341: AB: It's pretty similar, with a silver outline, kind of black in the middle.
¶342:
¶343: TF: So, does it look closer to the original 2 than the first try?
¶344:
¶345: AB: Yeah.
¶346:
¶347: TF: But it still has a little bit of black in it like the first try
¶348:
¶349: AB: Yeah, so it's got a metallic outline and then kind of black in the middle.
¶350:
¶351: TF: Ok, well we are getting closer. One more try, what about in that direction?
¶352:
¶353: AB: Yeah, it's kind of more similar now. It's got the silver metally thing and the sort of outline and sort of purpley thing on the inside.
¶354:
¶355: TF: So it's not as black anymore?
¶356:
¶357: AB: No.
¶358:
¶359: TF: Is it getting more shiny?
¶360:
¶361: AB: Yeah, so I can see a bit of side of the 2 and it's kind of shiny.
¶362:
¶363: TF: Is it the same kind of metallic shininess than the 2 in front of you?
¶364:
¶365: AB: Yeah.
¶366:
¶367: TF: So can you easily switch your attention between the 2 in front of you and the 2 over there?
¶368:
¶369: AB: Yeah.
¶370:
¶371: TF: Are they in the same kind of space or are they both?
¶372:
¶373: AB: Both kind of sitting on the tables. So this one is directly facing me and that one is kind of facing that way. So I’m kind of slightly sideways on.
¶374:
¶375: TF: So if I looked at it, I would see it face on <questioning tone>
¶376:
¶377: AB: Yeah, pretty much. Well, if you are in the chair one to your right would face you.
¶378:
¶379: TF: So let’s try one more thing. If I imagine something fantastic, let’s say a pink elephant. You could try this as well, then I decide no I don’t want a pink one I want a grey one. I can kind of change it. In terms of the 2 over there it's not quite to the 2 that’s in front of you. So do you think you can imagine it would vary like the purple bit in the middle bit to disappear or be replaced by the metallic outer rim?
¶380:
¶381: AB: Erm, it's harder with the elephant. You get a bit of green at the bottom. Hard to get it fully metallic.
¶382:
¶383: TF: <laughter> Does this one have green at the bottom?
¶384:
¶385: AB: No.
¶386:
¶387: TF: So there’s a new colour appeared, so it's actually resisting a little bit more than the imagination of the elephant? <AB agreeing throughout>
¶388:
¶389: TF: Ok, it would be interesting to explore the nature of this resistance a little bit more. So when you say it's harder, what does that mean? Does that mean when you try something unexpected happens or nothing happens?
¶390:
¶391: AB: Nothing happened for a while and then I got a bit of green at the bottom.
¶392:
¶393: TF: So even though you said ok I want to imagine it, I want to change it to be metallic in the middle, it didn’t happen for a while.
¶394:
¶395: AB: Kind of metallic one appearing briefly over there in the middle of the space, and that one won’t really change.
¶396:
¶397: TF: Which one was the metallic one here on the front somewhere above?
¶398:
¶399: AB: Yes, in there, facing you.
¶400:
¶401: TF: Oh that’s cool. <laughter> So this one, can you please describe this one.
¶402:
¶403: AB: So it's like a big thick, fat like dark 2, almost black.
¶404:
¶405: TF: So it has the metallic shininess of the first 2, but it has still a little bit of the dark blackness of the other tries. <AB agreeing throughout> How hard would it be for example, to raise the luminance and less dark and more metallic?
¶406:
¶407: AB: Erm, quite hard. I mean I got a bit more light, like a light kind of appeared above it, shone on it. It's pretty dark, a little bit of reflection of white light off the side of the 2, but not that much more.
¶408:
¶409: TF: When you said that just now to me, you didn’t actually look there did you? Were you able to somehow recall it while looking at me?
¶410:
¶411: AB: Yeah.
¶412:
¶413: TF: How did you do that?
¶414:
¶415: AB: Oh just recalling what I saw over there.
¶416:
¶417: TF: Oh ok. So it's not like you had a visualisation while you were talking to me?
¶418:
¶419: AB: Might have done. <laughter> Well, I was just remember what was going on over there.
¶420:
¶421: TF: Ok, but it was a visual memory <questioning tone>
¶422:
¶423: AB: Yeah.
¶424:
¶425: <Pause>
¶426: TF: Ok, because I noticed that several times today you were able to describe in quite precise detail the textures and colours and the different comparisons between the numbers. And I expected you to actually look at one of them and look at the other back and forth to make the comparison, but most of the time you were looking at me directly and still you know giving me quite precise details. But you didn’t make any eye movements so it wasn’t like there was like an imaginative comparison, I guess. <AB agrees throughout> Let’s try. If I imagine my room and the office and I was suppose to compare one wall with the other let’s say, I am tempted to make an eye movement, but I think I can do it without them straining. That’s hard. <pauses to think> So when it's part of the matter, it was easier to create a new 2 then to change the one that was already there in the left corner. But it's still not quite the same right?
¶427:
¶428: AB: No.
¶429:
¶430: TF: Ok, can you try one last time and then we’ll switch topics. Choose another spot maybe there and then look first again. Ok, and then try very hard to get a similar one.
¶431:
¶432: AB: Yeah, there’s a very similar one on top of the chair over there next to you
¶433:
¶434: TF: This one here, how does one look?
¶435:
¶436: AB: Yeah, pretty similar to this one.
¶437:
¶438: TF: So it's not that dark anymore.
¶439:
¶440: AB: I’d say that one over there is very similar as well.
¶441:
¶442: TF: Ah but didn’t that have some sort of purplish thing in the middle?
¶443:
¶444: AB: Yeah, this one has gone a bit more purple in the middle.
¶445:
¶446: TF: Oh really <laughter> So this won’t change?
¶447:
¶448: AB: Yeah
¶449:
¶450: TF: Right, let’s leave it there for now. So there were some other things we wanted to look at. One thing I was curious about was, in one of the sessions you described this kind of damp cave for the alphabet. I just wanted to ask a few more questions, if I say to you what about the letter C, can you see it?
¶451:
¶452: AB: Yeah, it's kind of there next to you.

¶453: TF: Does it appear in the cave context?
¶454:
¶455: AB: No, it's in space.
¶456:
¶457: TF: Ok, what if I say to you, how many letters are between C and J?
¶458:
¶459: AB: Yeah, first number compared was 5, that’s a little bit wrong isn’t it. D E F G H I.
¶460:
¶461: TF: Between C and J right, I said. <AB agrees>
¶462:
¶463: AB: Basically, what happened I kind of saw the alphabet form and kind of guessed how many letters were in between, without kind of counting them.
¶464:
¶465: TF: Right, so without this alphabet form, where does it appear, Just there?
¶466:
¶467: AB: So the J was there and the C was there, I guess there were about 5 letters in between.
¶468:
¶469: TF: So you didn’t actually go count them visually?
¶470:
¶471: AB: No, I didn’t count them.
¶472:
¶473: TF: So what do we need to do in order to bring back the background in which the alphabet previously appeared?
¶474:
¶475: AB: Don’t know.
¶476:
¶477: TF: Ok let’s try this. So, if we have the C, what does the background of the C?
¶478:
¶479: AB: The C is just kind of in the room at the moment. Probably need to move it a bit more on an empty space.
¶480:
¶481: TF: So what about in the middle of the room, there’s a nice big empty space <questioning tone>
¶482:
¶483: AB: Yeah. So I’ve kind of got a C.
¶484:
¶485: TF: What does it look like?
¶486:
¶487: AB: It's kind of the black page you get, the white page sorry, black ink with the sort of slightly poor photocopy <laughter>
¶488:
¶489: TF: So, can you let the page melt from the bottom of.
¶490:
¶491: AB: Yeah. There’s the rest of the alphabet around it. It's kind of against a dark sort of purplish background.
¶492:
¶493: TF: Can you say where the dark purplish background ends? Does it have an outline or does it just go off, traces to the sides?
¶494:
¶495: AB: Yeah, it's kind of around where the furthest desks are in this room.
¶496:
¶497: TF: Do the letters appear directly attached to it?
¶498:
¶499: AB: Erm no, it's kind of in front of it.
¶500:
¶501: TF: The letters are in front of it <questioning tone>
¶502:
¶503: AB: The background yeah.
¶504:
¶505: TF: So in that case, there is some sort of depth to the image. So how far are the letters away from the background?
¶506:
¶507: AB: Not that far.
¶508:
¶509: TF: If you let your gaze wander let’s say upwards, when does the background end?
¶510:
¶511: <pauses>
¶512: AB: Can’t see, the ceiling is kind of interfering with it.
¶513:
¶514: TF: So it's higher than the ceiling?
¶515:
¶516: AB: Yeah.
¶517:
¶518: TF: What about the width?
¶519:
¶520: AB: Yeah, that wall’s kind of interfering with it. Over there, I can kind of see it continuing on, in that way it's almost like we are in a sort of spherical thing.
¶521:
¶522: TF: So it's coming back around, it's curving in?
¶523:
¶524: AB: Yeah, there’s a bit of a white light coming in from over there, so it ends about there.
¶525:
¶526: TF: So it's a slightly different space from what we had before. So there’s no hole into nowhere, so it's all kind of like a solid background. Made out of some sort of purpley stuff?
¶527:
¶528: AB: Yeah, it's a bit kind of translucent, a bit like the texture of the iris of an eye.
¶529:
¶530: TF: The texture. Ok, I see what you mean. So where’s the light coming from?
¶531:
¶532: AB: So there’s a bit of white kind of coming in from over there. So I sense like white behind me.
¶533:
¶534: TF: And that’s illuminating the alphabet?
¶535:
¶536: AB: Yeah. Well I mean the letters are black, but they’re of standing out against a purplish background.
¶537:
¶538: TF: Right.
¶539:
¶540: AB: But the purplish background seems kind of luminescent itself as if it's giving off its own light over there.
¶541:
¶542: TF: So it's a bit radiant?
¶543:
¶544: AB: Yeah.
¶545:
¶546: TF: So that means that when you see the black letters in front of the backgrounds there’s a little bit of luminance coming from behind <questioning tone>
¶547:
¶548: AB: Erm, not really. Not enough to like see a shadow or anything.
¶549:
¶550: TF: But I guess it must be lighter than the letters themselves?
¶551:
¶552: AB: Yeah.
¶553:
¶554: TF: Well I guess there couldn’t be a shadow anyway, because the shadow would be falling this way.
¶555:
¶556: AB: Yeah. I can’t really see the floor.
¶557:
¶558: TF: Oh yeah, what about the floor? <cross talking>
¶559:
¶560: AB: Yeah, the floor in this room is obscuring the floor in that space. I can’t really see down.
¶561:
¶562: TF: It goes deeper?
¶563:
¶564: AB: It goes deeper than the room. Can’t really see the bottom. If anything it's kind of black at the bottom.
¶565:
¶566: TF: And what about this side, is the wall curving here like it does on that side of the room behind us?
¶567:
¶568: AB: Erm, no it just kind of eventually kind of blackness.
¶569:
¶570: TF: So it stops, the purplish?
¶571:
¶572: AB: Erm, well it just kind of goes beyond the eye can see. So there’s like a vanishing point and there’s not enough light to illuminate it beyond a certain point.
¶573:
¶574: TF: Oh the light from there, coming from your left <questioning tone>
¶575:
¶576: AB: Yeah, but it's kind of like being shon into like a really big cave, so eventually you can’t see any further.
¶577:
¶578: TF: But the material in the cave this time is quite different to what we had last time. <AB agrees> If you were to touch it, what would it be like? Does it feel like, you know organic material, does it look like super solid like a crystal?
¶579:
¶580: AB: Er no, it seems kind of ghost like.
¶581:
¶582: TF: So it looks like you could actually go through it.
¶583:
¶584: AB: Yeah.
¶585:
¶586: TF: So I’m just curious. So is the light, so there’s an opening? Right, let’s switch twice a little bit, you wanted to look at some arithmetic problems <AB agrees throughout>. So, what would you do if I said to you let’s say add 27 to 44?
¶587:
¶588: AB: Right, I see 27 44 kind of like highlighted. Erm, then I jump to 74 then back to 71. So the first 27 stood out then 44 stood, then 74 stood and then 71 stood out. So 27 was here, 44 was there, 74 was over there and 71 over there.
¶589:
¶590: TF: All on the same number line?
¶591:
¶592: AB: Yeah, same number line.
¶593:
¶594: TF: So you could fit all of the different numbers into one. What about all the numbers in between?
¶595:
¶596: AB: Yeah, so I wasn’t really that aware of the numbers in between. I guess I was vaguely aware of the 30’s being in between the 20’s and the 40’s. I wasn’t aware of the 50’s or 60’s at all.
¶597:
¶598: TF: So how did you get from, what was it 27 to was it 77?
¶599:
¶600: AB: 74. So I added 30 onto 44 and then subtracted 3
¶601:
¶602: TF: <laughter> Ok.
¶603:
¶604: AB: Took me a long time to do the addition, like at first I was really like kind of like just focusing on the number line. I was like ok there’s 27, there’s 44, and then I was wondering if some automatic process was going to happen. I had to just consciously say right ok now we can do the sum and like and have a strong like conscious intention to do the sum, it was only then that I formed the sum. So once I decided to do the sum it was 74 that sort of lit up, then I went back to 71.
¶605:
¶606: TF: Er right, the way I would have done it is to add let’s say the unit, say 60 and then do a smaller addition. But you actually added 30 to the first one and then subtracted 3 to get to 27. <AB agrees throughout> Well what if I said to you 27 minus 44?
¶607:
¶608: AB: So, I kind of got to 0 and then I was like what’s left and I realised 17 was left and so I ended up minus 17. So I saw 27, I saw 0 and then I saw -17. I wasn’t really aware of what really happened, but it was all kind of the same number line again.
¶609:
¶610: TF: What was your answer?
¶611:
¶612: AB: So the only point -17.
¶613:
¶614: TF: Am I doing my maths wrong. -17 yeah.
¶615:
¶616: AB: So, I’m a bit less clear how I did that sum. Erm, so it's a 27 lit up then 0, same number line and then I just kind of knew there was like a 17 to subtract. But I didn’t see 44 this time.
¶617:
¶618: TF: Ok, shall we try a division then. Maybe first multiplication, what about 8 times 7?

¶619:
¶620: AB: So, yeah kind of. That one is a bit strange, for some reason I did 8 times 9 first 72 lit up, then I realised you said you said 7 and 56 did. <laughter> So again it kind of seems to be the same number line. I have no idea how I kind of got to it, maybe I memorized the times table, I don’t know.
¶621:
¶622: TF: Shall we try a higher one, what about 12 times 13?
¶623:
¶624: AB: <pauses> Yeah, so I got. I kind of saw 12, saw 13, saw 144 and then I saw 156.
¶625:
¶626: TF: What did you do in order to get to the 144?
¶627:
¶628: AB: So I probably just memorised like 12 times 12, like recalled that from memory and I added on another 12.
¶629:
¶630: TF: I see, I would have done it differently, again it's interesting. I would have just said multiply 1 by the tens and then by the units. And then the two sums together. <AB agrees> But you seem to like doing certain multiplications for certain changes and then subtract something, add something again. Like with the 30 and then taking off 3 later. Ok, in terms of let’s say division, let’s say 110 divided by 5?
¶631:
¶632: AB: Right, so 110 lit up and 5 lit up, then I kind of saw 120, number 100, number 20 and then 22.
¶633:
¶634: TF: So did you have a feeling that all these different numbers lit up on their right spot on the number line?
¶635:
¶636: AB: Yeah.
¶637:
¶638: TF: But you didn’t manipulate the number in your way.
¶639:
¶640: AB: No, it's almost like I kind of do, Unless I really know what’s going on. I saw 110, I saw 5. I couldn’t do 110 divided by 5. How about a rounder number 100, 100 lit up and gave me the answer automatically 20 and then it's kind of like what’s left over there’s another 2 there so 22.
¶641:
¶642: TF: So that’s how I would do it. Except for the visual feedback.
¶643:
¶644: AB: I’m really focusing on the number line, I normally see that on a <laughter>
¶645:
¶646: TF: So let’s see if you were doing calculations on your computer or something like that, you wouldn’t get a visual representation of all the numbers.
¶647:
¶648: AB: Probably as if I was typing it into the calculator. <laughter>
¶649:
¶650: TF: Yeah, that’s true actually, nowadays we do very little calculations by hand or mentally. <AB agrees>
¶651:
¶652: AB: But yeah, certainly I am visualising something like that.
¶653:
¶654: TF: Like you did for the physics equations <questioning tone>
¶655:
¶656: AB: Yeah exactly.

¶657: TF: So when, ok let’s try that again. So E, what do you see for E?
¶658:
¶659: AB: Just see the letter E somewhere between 2 and 3 on the number line.
¶660:
¶661: TF: So, the others numbers are already present? Is there a line below them?
¶662:
¶663: AB: Below the numbers. <questioning tone>
¶664:
¶665: TF: Well where the.
¶666:
¶667: AB: Yes, they’re all just kind of up here from the manipulation we’ve been doing.
¶668:
¶669: TF: What does the E look like?
¶670:
¶671: AB: Just an E
¶672:
¶673: TF: Is it just plain black, normal font, no texture <AB agreeing throughout with these>
¶674:
¶675: AB: Slightly overlapping with the number 3 that’s closer to 2 than 3. And the numbers are quite bunched up because I’ve got about 120 of them. <laughter>
¶676:
¶677: TF: From the previous stuff?
¶678:
¶679: AB: Yeah.
¶680:
¶681: TF: Ok, so when you said E you located it on the number line that was already there. <AB agrees> What about creating a new one?
¶682:
¶683: AB: Yeah, there would be E equals 2.71828
¶684:
¶685: TF: So it's

¶686: AB: It's on a white page.

¶687: TF: So there’s the letter E and the equals sign and then the numbers and then what trailing off?
¶688:
¶689: AB: Yeah, kind of dot dot dot
¶690:
¶691: TF: Do you see anything else, are they bunched up?
¶692:
¶693: AB: There are a whole load of other numbers trying to get in, their decimal places are already succeeding in getting there.
¶694:
¶695: TF: Which one is the last one that got in?
¶696:
¶697: AB: 8
¶698:
¶699: TF: And that’s a pretty solid 8?
¶700:
¶701: AB: Yeah.
¶702:
¶703: TF: And the one after, is anything there?
¶704:
¶705: AB: dot dot dot and a whole scrapheap of numbers kind of sitting there in a pile.
¶706:
¶707: TF: But there wasn’t any say any imagery associated with the letter?
¶708:
¶709: AB: No.
¶710:
¶711: TF: Strange, I remember some of the examples we had before. What about a physical constant like c
¶712:
¶713: AB: c equals 3.0 x 10 to the 8
¶714:
¶715: TF: Is that what you see? Also again written <AB agrees on both>
¶716:
¶717: AB: m s to the minus 1

¶718: TF: m s to the minus 1, also written?
¶719:
¶720: AB: Yeah. See a Plank’s constant h bar kind floating around there as well <laughter and cross talking>
¶721:
¶722: TF: Well the E didn’t associate, because that’s a different E than the physical E, so there’s an ambiguous E somehow.
¶723:
¶724: AB: Erm, which E?
¶725:
¶726: TF: There’s one E for energy, and then there’s the E which is the rational number. Do they look different? I don’t know the other one is just a letter.
¶727:
¶728: AB: <cross talking, unclear> (53:43) That’s the lower case E. Yeah, I get an E=mc popping up over there now as well, now you said that. <laughter>
¶729:
¶730: TF: Well, the capital E says that. So is it also on the white page?
¶731:
¶732: AB: Yeah.
¶733:
¶734: TF: Kind of attached to the numbers somehow, is it in the same space? How is it associated?
¶735:
¶736: AB: Just a white page with the formula.
¶737:
¶738: TF: Erm anyway, I was trying to bring that up as an example of an equation on a page. But if I told you to say 5 times 7, if you try to do it.
¶739:
¶740: AB: Now, it's just a number lights up again, 5 and 7, 35. I got it very briefly 5 times 7 equals 35 over there, but only once you kind of said that. First thing that happened was 5 and 7 on the number line and then 35.
¶741:
¶742: TF: So what did you do to bring up the representation? Did you think of what would it look like on the?
¶743:
¶744: AB: The page. It's only after I gone through the number line to get 35.
¶745:
¶746: TF: Ok, at least it shows that it's a possibility but it's not something that seems to be happening spontaneously.
¶747:
¶748: AB: Yeah, it didn’t interrupt for any of the other ones we did.
¶749:
¶750: TF: I think this is pretty much it for me unless you want to explore something more.
¶751:
¶752: AB: No I think that’s it.

¶753: TF: Ok cool.
¶754:
¶755: **End of the interview.**
